# Exploring the Long-Term Hydrolytic Behavior of Zwitterionic Polymethacrylates and Polymethacrylamides

**DOI:** 10.3390/polym10060639

**Published:** 2018-06-08

**Authors:** Eric Schönemann, André Laschewsky, Axel Rosenhahn

**Affiliations:** 1 Institute of Chemistry, University of Potsdam, Karl-Liebknecht-Str. 24-25, D-14476 Potsdam-Golm, Germany; eschoenemann@uni-potsdam.de; 2 Fraunhofer Institute of Applied Polymer Research IAP, Geiselberg-Str. 69, D-14476 Potsdam-Golm, Germany; 3 Institute of Analytical Chemistry—Biogrenzflächen, Ruhr-Universität Bochum, Universitätsstr. 150, D-44801 Bochum, Germany; axel.rosenhahn@rub.de

**Keywords:** polyzwitterions, stability, polymer degradation, hydrolysis, polysulfobetaine, polysulfabetaine, anti-fouling materials

## Abstract

The hydrolytic stability of polymers to be used for coatings in aqueous environments, for example, to confer anti-fouling properties, is crucial. However, long-term exposure studies on such polymers are virtually missing. In this context, we synthesized a set of nine polymers that are typically used for low-fouling coatings, comprising the well-established poly(oligoethylene glycol methylether methacrylate), poly(3-(*N*-2-methacryloylethyl-*N,N*-dimethyl) ammoniopropanesulfonate) (“sulfobetaine methacrylate”), and poly(3-(*N*-3-methacryamidopropyl-*N,N*-dimethyl)ammoniopropanesulfonate) (“sulfobetaine methacrylamide”) as well as a series of hitherto rarely studied polysulfabetaines, which had been suggested to be particularly hydrolysis-stable. Hydrolysis resistance upon extended storage in aqueous solution is followed by ^1^H NMR at ambient temperature in various pH regimes. Whereas the monomers suffered slow (in PBS) to very fast hydrolysis (in 1 M NaOH), the polymers, including the polymethacrylates, proved to be highly stable. No degradation of the carboxyl ester or amide was observed after one year in PBS, 1 M HCl, or in sodium carbonate buffer of pH 10. This demonstrates their basic suitability for anti-fouling applications. Poly(sulfobetaine methacrylamide) proved even to be stable for one year in 1 M NaOH without any signs of degradation. The stability is ascribed to a steric shielding effect. The hemisulfate group in the polysulfabetaines, however, was found to be partially labile.

## 1. Introduction

Any solid surface exposed to aqueous media is prone to the unwanted adsorption of material, be it low molar mass compounds, macromolecules, or living organisms. This phenomenon is known as (bio)fouling. It is an ubiquitous and persistent challenge for any surface that is exposed to the environment, in particular to sea and fresh water biotopes, as well as to biological fluids or tissue [1,2]. One common strategy to inhibit fouling is based on the surface modification by hydrophilic polymers, which are swollen by water and minimize the unspecific adsorption of material [3,4]. Several systematic studies conclude that best results are achieved when attractive interactions to the surface are minimized, by avoiding electrostatic attraction and strong hydrogen bonding between the foulant and the hydrophilic polymers that protect the underlying surface [5,6,7]. In particular, derivatives of poly(ethylene glycol) and many zwitterionic polymers seem to resist fouling effectively due to their extensive hydration, and thus have been employed widely for the construction of antifouling coatings [8,9,10,11,12]. Obviously, the correct performance of such polymer coatings requires their appropriate stability under the pertinent conditions—first of all against hydrolysis. In fact, aqueous media can be chemically quite aggressive, especially under acidic or basic conditions.

Surprisingly, the stability of antifouling polymer coatings against degradation by water is seldom considered [13,14]. Although occasionally concerns are expressed, they are mostly discussed on the basis of common wisdom rather than of experimental facts. Accordingly, polymers with all-carbon backbones are preferred, avoiding the risk of backbone hydrolysis [15,16,17]. For such vinyl and vinylidene polymers, it is generally assumed that polymers connecting the functional side groups by ester moieties to the backbone (such as poly((meth)acrylates)) are considerably more sensitive to hydrolysis than analogs bearing amide moieties (such as poly((meth)acrylamides)), and much more than derivatives of polystyrene or poly(diallylammonium) compounds [18,19,20,21]. Also, it is generally accepted that hydrolysis is accelerated by increasing temperature, as well as by acidic or basic conditions. Still, particularly for the most widely employed antifouling polymers that bear oligo(ethylene glycol) or zwitterionic moieties in their side chains, experimental studies on their hydrolysis are scarce [22,23,24,25,26]. Moreover, they consider typically short periods only, that is, a few days at most. The majority of the systematic studies seem to have been conducted on the hydrolytic stability of the underlying monomers [27,28,29,30,31], but not on the polymers, assuming tacitly that the difference would be small. Accordingly, the literature assessments of the stability of antifouling polymers often represent mere assumptions, or are based on singular observations for which the precise experimental conditions are often not disclosed. According to the data available, polyacrylates seem to be rather susceptible to hydrolysis, much more than polymethacrylates [32,33,34,35,36,37]. Moreover, for the case of the monomers, methacrylamides were shown to resist hydrolysis better than methacrylates do [28,29]. Still, hydrolysis of both zwitterionic polymethacrylates and polymethacrylamides was noted occasionally [38,39]. However, the pH value of the reaction media remained unclear, and whether the hydrolytic degradation encountered in the final polymers took place at the polymer level, or on the monomer level prior to incorporation into the polymers. Note that, at least under basic conditions, pyridinium and benzylammonium moieties may also suffer degradation in aqueous media [40,41,42]. Further, studies on a set of zwitterionic (meth)acrylic systems revealed that 3-ammoniopropanoate derivatives are prone to degradation [14,29,43], presumably by elimination of a tertiary amine of these Mannich-base type compounds.

**Figure 1:**
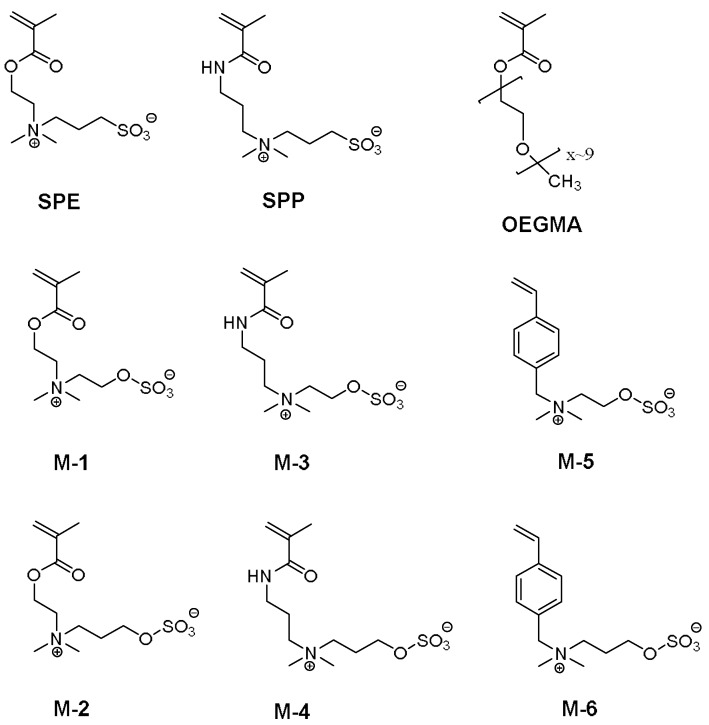
Chemical structures of the zwitterionic sulfobetaine (**SPE**, **SPP**)- and sulfabetaine monomers (**M-1** to **M-6**) used, and of the reference monomer **OEGMA**.

Comprehensive studies on the stability of antifouling polymers are missing, especially those covering long durations. Therefore, we investigated the hydrolytic stability for a set of six sulfabetaine and two sulfobetaine homopolymers, which are typically employed for antifouling studies. The chemical structures of the respective monomers are displayed in Figure 1. Degradation of these polymers in aqueous solution was studied over a period of up to one year at different pH values, and compared with the behavior of their monomers. As reference, we included the non-ionic homopolymer of an (oligoethylene glycol) methacrylate macromonomer, as such polymers have been widely used to implement antifouling behavior [2,10,44,45]. In addition to the most frequently used polysulfobetaines **P-SPE** and **P-SPP**, whose monomers are commercially available, we have also studied a series of polysulfabetaines, which are distinguished from the former polyzwitterions by the hemisulfate group forming the anionic sites instead of the sulfonate group [20]. The particular interest to include the large set of polysulfabetaines in our study arose from recent claims that this previously hardly studied polymer class [39,46,47,48,49,50] excels with respect to their stability against hydrolysis [46,48]. To keep the number of experiments manageable, the study was performed at room temperature, which seemed a reasonable choice in view of most outdoor applications. The stability of the various compounds against hydrolysis was followed by ^1^H NMR spectroscopy of aqueous solutions as a function of their contact time. Solutions were prepared with precise pH values, namely 0, 7.4, 10, and 14. When necessary, a defined amount of NaCl was added to decrease the upper consolute boundary to below 22 °C, as many of the polyzwitterions studied exhibit a high upper critical solution temperature (UCST) in pure water that is effectively reduced by added electrolytes [51,52,53,54].

## 2. Materials and Methods

### 2.1. Materials

Initiator 2,2′-azobisisobutyronitrile “AIBN” (Sigma Aldrich Chemie GmbH, Taufkirchen, Germany, 98%) was crystallized from methanol prior to use. Poly(ethylene glycol) methyl ether methacrylate “**OEGMA**” (*M*_r_ = 500, Sigma Aldrich, containing 100 ppm of monomethyl hydroquinone “MEHQ” and 200 ppm of 2,6-di-*tert*-butyl-4-methylphenol “BHT” as inhibitors) was purified prior to use by filtration through a bed of aluminum oxide (“ALOX” 90 active neutral, 70-230 mesh ASTM 0.063–0.2 mm, Merck, Darmstadt, Germany). Monomers 3-[*N*-2-(methacryloyloxy)ethyl-*N,N*-dimethyl]ammonio propane-1-sulfonate “**SPE**” (Sigma Aldrich, ≥97%) and 3-[*N*-3-(methacrylamido)propyl-*N,N*-dimethyl]ammonio propane-1-sulfonate “**SPP**” (gift from Raschig GmbH Ludwigshafen/Germany) were used as received. Synthesis and purification of monomer **M-2** were described before.^39^ The syntheses of sulfabetaine monomers **M-1** and **M-3** to **M-6** are described below, modifying recipes from the literature [46,47]. Reagents and solvents 2-(dimethylamino)ethyl methacrylate (Aldrich, ≥98%, 2000 ppm MEHQ), *N*-[3-(dimethylamino) propyl] methacrylamide (gift from Evonik Degussa, Hanau/Germany), *N*-(4-vinylbenzyl)-*N,N*-dimethylamine (Acros Organics/Fisher Scientific GmbH, Schwerte, Germany, 90% stabilized with 0.1% 4-*tert*-butylcatechol), 1,3,2-dioxathiolane 2,2-dioxide (“ethylenesulfate”, TCI, Deutschland GmbH, Eschborn, Germany, ≥98.0%), 1,3,2-dioxathian 2,2-dioxide (“propylenesulfate”, TCI ≥ 98.0%), acetonitrile (Carl Roth GmbH, Karlsruhe, Germany, ≥99,9%), acetone (VWR International GmbH, Darmstadt, Germany, 100.0%), trifluoroethanol (Roth, 99.8%), methanol (VWR, ≥98.5%), ethanol (Chemsolute/Th. Geyer, Renningen, Germany, ≥99.5%), sodium chloride (Chemsolute, ≥99.0%), sodium hydroxide (Chemsolute, ≥99.8), deuterium chloride (ARMAR Chemicals, Döttingen, Switzerland 38 wt % in D_2_O ≥99.5 atom % deuterium), deuterium oxide (VWR, ≥99.90 atom % deuterium), sodium carbonate (Acros, purum), sodium hydrogen carbonate (Roth, ≥99%), phosphate buffered saline tablets (“PBS”, Sigma-Aldrich, Lot BCBF6911), 3-(trimethylsilyl) propionic acid-d4 sodium salt (Acros, 98 atom % deuterium), and 1,1,1,3,3,3-hexafluoro-2-propanol “HFIP” (Fluorochem, Lot FCB013827) were used as received if not stated otherwise. Deionized water was further purified by a Millipore Milli-Q Plus water purification system (Merck Millipore, Darmstadt, Germany), resistivity 18 mΩ·cm^−1^. 

### 2.2. Synthetic Methods and Procedures

#### 2.2.1. Synthesis of Sulfabetaine Monomers

*2-(N-(2-(methacryloyloxy)ethyl)-N,N-dimethylammonio) ethyl sulfate (**M-1**):* 2-(dimethylamino)ethyl methacrylate (6.92 g, 44 mmol, 1.1 eq.) and 1,3,2-dioxathiolane 2,2-dioxide (4.96 g, 40 mmol, 1.0 eq.) were dissolved in acetonitrile (60 mL) and stirred at 50 °C for 40 h. The reaction mixture was cooled to −25 °C. The solid formed was filtered off and washed with acetonitrile. After drying in vacuum, **M-1** was obtained as colorless powder (11.25 g, 92%).

^1^H NMR (300 MHz, D_2_O, 298 K) δ (ppm) = 6.20 (s, 1H, CH=C–COO– (cis)), 5.81 (s, 1H, CH=C–COO– (trans)), 4.70 (bs, 2H, –COO–CH_2_–), 4.55 (bs, 2H, –CH_2_–OSO_3_^−^), 4.06–3.80 (m, 4H, –CH_2_–N^+^–CH_2_–), 3.32 (s, 6H, –N^+^–CH_3_), 1.97 (s, 3H, =C–CH_3_). ^13^C NMR (75 MHz, D_2_O, 298 K) δ (ppm) = 169.01 (–COO–), 135.83 (=C–COO–), 128.37 (=CH_2_), 64.34 (–COO–C–C–N^+^–), 64.15 (–N^+^–C–C–OSO_3_^−^), 62.36 (–N^+^–C–C–OSO_3_^−^), 59.05 (–COO–C–C–N^+^–), 52.83 (–N^+^–CH_3_), 17.95 (–C–CH_3_). HR-MS (ESI): calculated: 282.1011 [M + H]^+^; found: 282.0997 [M + H]^+^. Elemental analysis (C_10_H_19_NO_6_S, *M*_r_ = 281.32): calculated: C = 42.69%, H = 6.81%, N = 4.98%, S = 11.40%, O = 34.12%; found: C = 42.30%, H = 6.69%, N = 5.11%, S = 11.60%. FT-IR (selected bands in cm^−1^): 3033 ν(N^+^–CH_3_), 2974 ν(CH_3_), 1736 ν(C=O), 1641 ν(C=C), 1176 ν_as_(SO_3_^−)^, 1035 ν_s_(SO_3_^−^).

*2-(N-(3-methacrylamidopropyl)-N,N-dimethylammonio)ethyl sulfate (**M-3***): *N*-(3-(dimethylamino)propyl)methacrylamide (7.49 g, 44 mmol, 1.1 eq) and 1,3,2-dioxathiolane 2,2-dioxide (4.96 g, 40 mmol, 1.00 eq.) were dissolved in acetonitrile (60 mL) and stirred at 50 °C for 40 h. The reaction mixture was cooled to room temperature and acetone was added to complete precipitation. The formed solid was filtered off, washed with acetone. The solid was dispersed in acetonitrile (100 mL), shortly heated to reflux and allowed to cool to room temperature. The solid again was filtered off, washed with acetone and dried in vacuum. **M-3** was obtained as slightly yellowish powder (6.02 g, 51%).

^1^H NMR (300 MHz, D_2_O, 298 K) δ (ppm) = 5.74 (s, 1H, CH=C–COO– (cis)), 5.49 (s, 1H, CH=C-COO– (trans)), 4.49 (m, 2H, –CH_2_–OSO_3_^−^), 3.76 (m, 2H, –N^+^–CH_2_–C–OSO_3_^−^), 3.60–3.35 (m, 4H, –CNO–CH_2_–C–CH_2_–N^+^–), 3.20 (s, 6H, –N^+^–CH_3_), 2.10 (m, 2H, –CNO–C–CH_2_–C–N^+^–), 1.95 (s, 3H, =C–CH_3_). ^13^C NMR (75 MHz, D_2_O, 298 K) δ (ppm) = 172.63 (–CON–), 139.50 (=C–CON–), 121.97 (=CH_2_), 63.76 (–CON–C–C–C–N^+^–), 63.06 (–N^+^–C–C–OSO_3_^−^), 62.32 (–N^+^–C–C–OSO_3_^−^), 52.23 (–N^+^–CH_3_), 36.90 (–CON–C–C–C–N^+^–), 22.93 (–CON–C–C–C–N^+^–), 18.26 (–C–CH_3_). HR-MS (ESI): calculated: 295.1328 [M + H] ^+^; found: 295.1349 [M + H]^+^. Elemental analysis (C_11_H_22_N_2_O_5_S, *M*_r_ = 294.37): calculated: C = 44.88%, H = 7.53%, N = 9.52%, S = 10.89%, O = 27.18%; found: C = 44.46%, H = 7.57%, N = 9.50%, S = 11.12%. FT-IR (selected bands in cm^−1^): 3042 ν (N^+^–CH_3_), 2966 ν(CH_3_), 1663 ν(C=O_amide_), 1626 ν(C=C), 1212 ν_as_(SO_3_^−^), 1036 ν_s_(SO_3_^−^).

*3-(N-(3-methacrylamidopropyl)-N,N-dimethylammonio)propyl sulfate (**M-4**): N*-(3-(dimethylamino)propyl)methacrylamide (7.49 g, 44 mmol, 1.1 eq) and 1,3,2-dioxathiane 2,2-dioxide (5.53 g, 40 mmol, 1.00 eq.) were dissolved in acetonitrile (60 mL) and stirred at 50 °C for 40 h. The reaction mixture was cooled to room temperature. Acetone was added to complete precipitation. The formed solid was filtered off and washed with acetone. The solid was dispersed in acetonitrile (100 mL), shortly heated to reflux and allowed to cool to room temperature. The solid was filtered off, washed with acetone and dried in vacuum. **M-4** was obtained as weakly yellowish powder (8.52 g, 69%).

^1^H NMR (300 MHz, D_2_O, 298 K) δ (ppm) = 5.74 (s, 1H, CH=C–CON– (cis)), 5.50 (s, 1H, CH=C–N (trans)), 4.18 (t, *J*=5.6, 2H, –CH_2_–OSO_3_^−^), 3.49 (m, 2H, –N^+^–CH_2_–C–C–OSO_3_^−^), 3.44–3.32 (m, 4H, –CNO–CH_2_–C–CH_2_–N^+^–), 3.13 (s, 6H, –N^+^–CH_3_), 2.20 (m, 2H, –N^+^–C–CH_2_–C–OSO_3_^−^), 2.09 (m, 2H, –CNO–C–CH_2_–C–N^+^-), 1.96 (s, 3H, =C–CH_3_). ^13^C NMR (75 MHz, D_2_O) δ = 172.61 (–CON–), 139.48 (=C–CON–), 122.03 (=CH_2_), 65.93 (–N^+^–C–C–C–OSO_3_^−^), 62.44 (–CON–C–C–C–N^+^–), 61.57 (N^+^–C–C–C–OSO_3_^−^), 51.5 (2C, N^+^-CH_3_), 36.84 (CON–C–C–C–N^+^–), 22.95 (–N^+^–C–C–C–OSO_3_^−^) 22.90 (CON–C–C–C–N^+^–), 18.28 (–C–CH_3_). HR-MS (ESI): calculated: 309.1484 [M + H]^+^; found: 309.1487 [M + H]^+^. Elemental analysis (C_12_H_24_N_2_O_5_S, *M*_r_ = 308.39): calculated: C = 46.74%, H = 7.84%, N = 9.08%, S = 10.40%, O = 25.94%; found: C = 45.70%, H = 7.56%, N = 9.08%, S = 10.41%.FT-IR (selected bands in cm^−1^): 3041 ν(N^+^–CH_3_), 2973 ν(CH_3_), 1655ν (C=O_amide_), 1612 ν(C=C), 1215 ν_as_(SO_3_^−^), 1029 ν_s_(SO_3_^−^).

*2-(N,N-dimethyl(-N-4-vinylbenzyl)ammonio)ethyl sulfate (**M-5**)*: *N,N*-dimethylvinylbenzylamine (4.50 g, 28 mmol, 1.1 eq) and 1,3,2-dioxathiolane 2,2-dioxide (3.15 g, 25 mmol, 1.00 eq.) were dissolved in acetonitrile (40 mL) and stirred at 50 °C for 40 h. The reaction mixture was cooled to −25 °C. The formed solid was filtered off, washed with acetone and dried under reduced pressure. **M-5** was obtained as colorless powder (5.35 g, 74%).

^1^H NMR (300 MHz, D_2_O, 298 K) δ (ppm) = 7.60 (d, *J* = 8.1, 1H, =CH– (aryl C2)), 7.50 (d, *J* = 8.1, 1H, =CH– (aryl C3)), 6.81 (dd, *J* = 17.7, 10.9, 1H, =CH–C–), 5.92 (d, *J* = 17.7, 1H, CH=C–C– (cis)), 5.39 (d, *J* = 10.9, 1H, CH=C–C– (trans)), 4.55 (s, 2H, aryl-CH_2_–N^+^–), 4.51 (m, 2H, –CH_2_–OSO_3_^−^), 3.71 (m, 1H, –N^+^–CH_2_–C–OSO_3_^−^), 3.09 (s, 6H, –N^+^–CH_3_). ^13^C NMR (75 MHz, D_2_O, 298 K) δ (ppm) = 140.49 (H_2_C=CH–C–), 136.35 (H_2_C=CH–C–), 134.03 (=CH– (aryl C3)), 127.36 ((=CH– (aryl C2)), 126.78 (C=C–C–N^+^–), 116.96 (=CH_2_), 69.75 (C=C–C–N^+^–), 63.17 (–N^+^–C–C–OSO_3_^−^), 62.41 (–N^+^–C–C–OSO_3_^−^), 51.01 (–N^+^– (CH_3_)_2_). HR-MS (ESI): calculated: 286.1113 [M + H]^+^; found: 286.1112 [M + H]^+^. Elemental analysis (C_13_H_19_NO_4_S, *M*_r_ = 285.36): calculated: C = 54.72%, H = 6.71%, N = 4.91%, S = 11.24%, O = 22.43%; found: C = 54.29%, H = 6.56%, N = 4.89%, S = 11.51%. FT-IR (selected bands in cm^−1^): 3034 ν(N^+^–CH_3_), 2962 ν(CH_3_), 1612 ν(C=C), 1234 ν_as_(SO_3_^−^), 1032 ν_s_(SO_3_^−^).

*3-(N,N-dimethyl(-N-4-vinylbenzyl)ammonio)propyl sulfate (**M-6**)*: *N,N*-dimethylvinylbenzylamine (4.50 g, 28 mmol, 1.1 eq) and 1,3,2-dioxathiane 2,2-dioxide (3.50 g, 25 mmol, 1.00 eq.) were dissolved in acetonitrile (40 mL) and stirred at 50 °C for 40 h. The reaction mixture was cooled to room temperature. Acetone was added to complete precipitation. The formed solid was filtered off and recrystallized from hot water. **M-6** was obtained as colorless crystals (5.11 g, 67%).

^1^H NMR (300 MHz, D_2_O, 298 K) δ (ppm) = 7.64 (d, *J* = 7.8, 2H, =CH- (aryl C2)), 7.54 (d, *J* = 7.9, 1H, =CH– (aryl C3)), 6.86 (dd, *J* = 17.6, 11.0, 1H, =CH–C–), 5.96 (d, *J* = 17.6, 1H, CH=C–C– (cis)), 5.43 (d, *J* = 11.0, 1H, CH=C–C– (trans)), 4.51 (s, 2H, –C–CH_2_–N^+^–), 4.18 (t, *J* = 5.5, 2H, –CH_2_–OSO_3_^−^), 3.51–3.40 (m, 2H, –N^+^–CH_2_–C–C–OSO_3_^−^), 3.06 (s, 6H, –N^+^–CH_3_), 2.32 (m, 2H, –N^+^–C–CH_2_–C-OSO_3_^−^). ^13^C NMR (75 MHz, D_2_O, 298 K) δ (ppm) = 140.43 (H_2_C=CH–C–), 136.37 (H_2_C=CH–C-), 133.88 ((=CH– (aryl C3)), 127.39 ((=CH– (aryl C2)), 126.95 (C=C–C–N^+^–), 116.96 (=CH_2_), 68.23 (C=C–C–N^+^–), 66.02 (–N^+^–C–C–C–OSO_3_^−^), 61.64 (–N^+^–C–C–C–OSO_3_^−^), 50.39 (–N^+^–CH_3_), 23.15 ((–N^+^–C–C–C–OSO_3_^−^). HR-MS (ESI): calculated: 300.1270 [M + H]^+^; found: 300.1266 [M + H]^+^. Elemental analysis (C_14_H_21_NO_4_S, *M*_r_ = 299.39): calculated: C = 56.17%, H = 7.07%, N = 4.68%, S = 10.71%, O = 21.38%; found: C = 55.96%, H = 6.89%, N = 4.70%, S = 11.00%. FT-IR (selected bands in cm^−1^): 3039 ν(N^+^-CH_3_), 2956ν (CH_3_), 1629 ν(C=C), 1229 ν_as_(SO_3_^−^), 1030 ν_s_(SO_3_^−^).

#### 2.2.2. Polymer Synthesis

*Polymerization of OEGMA*: **OEGMA** (5.00 g, 10 mmol) and AIBN (8.2 mg, 0.05 mmol, 0.5 mol %) were dissolved in ethanol (20 g, 80 wt %) and purged with argon for 30 min. The reaction mixture was polymerized at 60 °C for 20 h. The reaction was stopped by allowing air into the vessel and cooling to ambient temperature. After dialysis against water and freeze drying, polymer **P-OEGMA** was obtained as highly viscous gluey syrup (yield 4.65 g, 93%). ^1^H NMR (300 MHz, D_2_O, 298 K) δ (ppm) = 4.47–4.02 (br, 2H, COO–CH_2_–C–O–), 4.02–3.50 (br, 34H, O–CH_2_–CH_2_–O–), 3.50–3.31 (br, 3H, OCH_3_), 2.38–1.57 (br, 2H, C–CH_2_–C), 1.39–0.68 (br, 3H, C–CH_3_). FT-IR (selected bands in cm^−1^): 2870 ν(CH_2_), 1727 ν(C=O). Elemental analysis (C_14_H_21_NO_4_S, *M*_r_ = 279.35): calculated: C = 55.63%, H = 8.93%, N = 0.00%, S = 0.00%, O = 35.44%; found: C = 53.90%, H = 9.12%, N = 0.00%, S = 0.00%. TGA: onset of decomposition 150 °C.

*Polymerization of SPE*: **SPE** (2.78 g, 10 mmol) and AIBN (8.2 mg, 0.05 mmol, 0.5 mol %) were dissolved in TFE (11 g, 80 wt %) and purged with argon for 30 min. The reaction mixture was polymerized at 60 °C for 20 h. The reaction mixture was repeatedly precipitated into methanol, then dialyzed in Milli Q water and freeze dried. Polymer **P-SPE** was obtained as colorless solid (yield 1.86 g, 67%). ^1^H NMR (300 MHz, in dilute aqueous NaCl (9.0 g·L^−1^) in D_2_O, 298 K) δ (ppm) = 4.63–4.27 (br, 1H, –COO–CH_2_–), 4.02–3.72 (br, 2H, –COO–C–CH_2_–N^+^–), 3.72–3.43 (br, 2H, N^+^–CH_2_–C–C–SO_3_^−^), 3.43–3.07 (br, 6H, –N^+^–CH_3_), 3.07–2.79 (br, 2H, –CH_2_–SO_3_^−^), 2.44–2.15 (br, 2H, –CH_2_–C–SO_3_^−^), 2.15–1.58 (br, 2H, –C–CH_2_–C), 1.29–0.56 (br, 3H, –C–CH_3_). FT-IR (selected bands in cm^−1^): 3034 ν(N^+^–CH_3_), 2977 ν(CH_3_), 1723 ν(C=O), 1170 ν_as_(SO_3_^−^), 1035 ν_s_(SO_3_^−^). Elemental analysis (C_11_H_21_NO_5_S, *M*_r_ = 279.35)): calculated: C = 47.30%, H = 7.58%, N = 5.01%, S = 11.48%, O = 28.64%; found: C = 44.88%, H = 8.18%, N = 4.75%, S = 10.72%. TGA: onset of decomposition 260 °C.

*Polymerization of SPP*: **SPP** (2.93 g, 10 mmol) and AIBN (8.2 mg, 0.05 mmol, 0.5 mol %) were dissolved in TFE (10 g, 80 wt %) and purged with argon for 30 min. The reaction mixture was polymerized at 60 °C for 20 h. The reaction mixture was repeatedly precipitated into methanol, then dialyzed in Milli Q water and freeze dried. Polymer **P-SPP** was obtained as colorless solid (yield 1.96 g, 67%).

^1^H NMR (300 MHz, in dilute aqueous NaCl (9.0 g·L^−1^) in D_2_O, 298 K) δ (ppm) = 3.65–3.49 (br, 2H, –CNO–C–C–CH_2_–N^+^–),), 3.49–3.34 (br, 2H, –N^+^–CH_2_–C–C–SO_3_^−^), 3.34–3.11 (br, 8H, –N^+^– (CH_3_)_2_, –CNO–CH_2_–C–C–N^+^–), 3.11–2.97 (br, 2H, –CH_2_–SO_3_^−^), 2.36–2.15 (br, 2H, –CNO–C–CH_2_–C–N^+^–), 2.15–1.96 (br, 2H, –N^+^–C–CH_2_–C–SO_3_^−^), 1.96–1.49 (br, 2H, –C–CH_2_–C), 1.24–0.78 (br, 3H, –C–CH_3_). FT-IR (selected bands in cm^−1^): 3034 ν(N^+^–CH_3_), 2938 ν(CH_3_), 1641 ν(C=O_amide_), 1179 ν_as_(SO_3_^−^), 1035 ν_s_(SO_3_^−^). Elemental analysis (C_12_H_24_N_2_O_4_S, *M*_r_ = 292.39): calculated: C = 49.29%, H = 8.27%, N = 9.58%, S = 10.96%, O = 21.89%; found: C = 45.54%, H = 9.10%, N = 8.86%, S = 10.16%. TGA: onset of decomposition 270 °C.

*Polymerization of M-1*: **M-1** (1.41 g, 5 mmol) and AIBN (4.1 mg, 0.025 mmol, 0.5 mol %) were dissolved in TFE (6 g, 80 wt %) and purged with argon for 30 min. The reaction mixture was polymerized at 60 °C for 20 h. After dialysis in Milli Q water and freeze drying, polymer **P-1** was obtained as colorless solid (yield 1.15 g, 82%).

^1^H NMR (300 MHz, in saturated NaCl solution in D_2_O, 298 K) δ (ppm) = 5.12–4.85 (br, 4H, –CH_2_–OSO_3_^−^, –COO–CH_2_–), 4.50–4.09 (br, 4H, –CH_2_–N^+^–CH_2_–), 3.84–3.51 (br, 6H, –N^+^–CH_3_), 2.83–1.88 (br, 2H, –C–CH_2_–C–), 1.80–0.73 (br, 3H, –C–CH_3_). FT-IR (selected bands in cm^−1^): 3037 ν(N^+^–CH_3_), 2997 ν(CH_3_), 1732 ν(C=O), 1149 ν_as_(SO_3_^−^), 1037 ν_s_(SO_3_^−^). Elemental analysis (C_10_H_19_NO_6_S, *M*_r_ = 281.32): calculated: C = 42.69%, H = 6.81%, N = 4.98%, S = 11.40%, O = 34.12%; found: C = 41.49%, H = 7.61%, N = 4.83%, S = 11.15%. TGA: onset of decomposition 280 °C.

*Polymerization of **M-2***: **M-2** (2.95 g, 10 mmol) and AIBN (8.2 mg, 0.05 mmol, 0.5 mol %) were dissolved in TFE (12 g, 80 wt %) and purged with argon for 30 min. The reaction mixture was polymerized at 60 °C for 20 h. After dialysis in Milli Q water and freeze drying, polymer **P-2** was obtained as colorless solid (yield 2.50 g, 85%).

^1^H NMR (300 MHz, in saturated NaCl solution in D_2_O, 298 K) δ = 5.00–4.67 (br, 2H, COO–CH_2_–), 4.65–4.45 (br, 2H, –CH_2_–OSO_3_^−^), 4.35–4.09 (br, 2H, –COO–C–CH_2_–), 4.09–3.86 (br, 2H, –N^+^–CH_2_–C–C–OSO_3_^−^), 3.76–3.46 (br, 6H, –N^+^– (CH_3_)_2_), 2.73–1.90 (br, 4H, –CH_2_–C–OSO_3_^−^, –C–CH_2_–C–), 1.66–1.15 (br, 3H, –C–CH_3_). FT-IR (selected bands in cm^−1^): 3034 ν(N^+^–CH_3_), 2969 ν(CH_3_), 1723 ν(C=O), 1146 ν_as_(SO_3_^−^), 1018 ν_s_(SO_3_^−^). Elemental analysis (C_11_H_21_NO_6_S, *M*_r_ = 295.35): calculated: C = 44.73%, H = 7.17%, N = 4.74%, S = 10.85%, O = 33.43%; found: C = 40.69%, H = 7.19%, N = 4.29%, S = 9.97%. TGA: onset of decomposition at 280 °C.

*Polymerization of M-3*: **M-3** (2.35 g, 8 mmol) and AIBN (6.6 mg, 0.04 mmol, 0.5 mol %) were dissolved in TFE (9 g, 80 wt %) and purged with argon for 30 min. The reaction mixture was polymerized at 60 °C for 20 h. After dialysis in Milli Q water and freeze drying, polymer **P-3** was obtained as colorless solid (yield 0.85 g, 36%).

^1^H NMR (300 MHz, in saturated NaCl solution in D_2_O, 298 K) δ (ppm) = 5.02–4.85 (br, 2H, –CH_2_–OSO_3_^−^), 4.31–4.10 (br, 2H, –N^+^–CH_2_–C–OSO_3_^−^), 3.97–3.77 (br, 4H, –CNO–CH_2_–C–CH_2_–N^+^–), 3.73–3.31 (br, 6H, –N^+^– (CH_3_)_2_), 2.59–1.90 (br, 4H, –CNO–C–CH_2_–C–N^+^–, –C–CH_2_–C–), 1.65–1.12 (br, 3H, –C–CH_3_). FT-IR (selected bands in cm^−1^): 3040 ν(N^+^–CH_3_), 2971 ν(CH_3_), 1652 ν(C=O_amide_), 1067 ν_s_(SO_3_^−^). Elemental analysis (C_11_H_22_N_2_O_5_S, *M*_r_ = 294.37): calculated: C = 44.88%, H = 7.53%, N = 9.52%, S = 10.89%, O = 27.18%; found: C = 42.37%, H = 8.12%, N = 8.97%, S = 10.46%. TGA: onset of decomposition at 280 °C.

*Polymerization of M-4*: **M-4** (2.47 g, 8 mmol) and AIBN (6.6 mg, 0.04 mmol, 0.5 mol %) were dissolved in TFE (10 g, 80 wt %) and purged with argon for 30 min. The reaction mixture was polymerized at 60 °C for 20 h. After dialysis in Milli Q water and freeze drying, polymer **P-4** was obtained as colorless solid (yield 0.85 g, 34%).

^1^H NMR (300 MHz, in saturated NaCl solution in D_2_O, 298 K) δ (ppm) = δ = 4.69–4.49 (br, 2H, –CH_2_–OSO_3_^−^), 3.99–3.84 (br, 2H, –N^+^–CH_2_–C–C–OSO_3_^−^), 3.84–3.71 (br, 4H, –CNO–CH_2_–C–CH_2_–N^+^–), 3.68–3.39 (br, 6H, –N^+^–CH_3_), 2.77–2.55 (br, 2H, –CH_2_–C–OSO_3_^−^), 2.55–2.08 (br, 4H, –CNO–C–CH_2_–C–N^+^–, –C–CH_2_–C), 1.67–1.07 (br, 3H, –C–CH_3_). FT-IR (selected bands in cm^−1^): 3053 ν(N^+^–CH_3_), 2970 ν(CH_3_), 1652 ν(C=O_amide_), 1066 ν_s_(SO_3_^−^). Elemental analysis (C_12_H_24_N_2_O_5_S, *M*_r_ = 308.39): calculated: C = 46.74%, H = 7.84%, N = 9.08%, S = 10.40%, O = 25.94%; found: C = 43.89%, H = 8.46%, N = 8.54%, S = 9.82%. TGA: onset of decomposition at 280 °C.

*Polymerization of M-5*: **M-5** (1.43 g, 5 mmol) and AIBN (4.1 mg, 0.025 mmol, 0.5 mol %) were dissolved in TFE (6 g, 80 wt %) and purged with argon for 30 min. The reaction mixture was polymerized at 60 °C for 20 h. After dialysis in Milli Q water and freeze drying, polymer **P-5** was obtained as colorless solid (yield 1.43 g, 100%). 

^1^H NMR (300 MHz, in saturated NaCl solution in D_2_O, 298 K) δ (ppm) = polymer did not dissolve in eluent D_2_O saturated with NaCl. FT-IR (selected bands in cm-1): 3035 ν(N^+^–CH_3_), 2925 ν(CH_3_), 1615 ν(C=C), 1227 ν_as_(SO_3_^−^), 1032 ν_s_(SO_3_^−^). Elemental analysis (C_13_H_19_NO_4_S, *M*_r_ =285.36): calculated: C = 54.72%, H = 6.71%, N = 4.91%, S = 11.24%, O = 22.43%; found: C = 51.65%, H = 7.05%, N = 4.67%, S = 10.31%. TGA: onset of decomposition at 280 °C.

*Polymerization of M-6*: **M-6** (1.50 g, 5 mmol) and AIBN (4.1 mg, 0.025 mmol, 0.5 mol %) were dissolved in TFE (6 g, 80 wt %) and purged with argon for 30 min. The reaction mixture was polymerized at 60 °C for 20 h. After dialysis in Milli Q water and freeze drying, polymer **P-6** was obtained as colorless solid (yield 1.42 g, 95%).

^1^H NMR (300 MHz, in saturated NaCl solution in D_2_O, 298 K) δ (ppm) = 8.71–6.46 (br, 4H, =CH–), 4.71–4.32 (br, 2H, –C–CH_2_–N^+^–) 4.18–2.92 (br, 10H, –CH_2_–OSO_3_^−^, –N^+^–CH_2_–C–C–OSO_3_^−^, –N^+^–CH_3_), 2.92–2.45 (br, 2H, –N^+^–C–CH_2_–C–OSO_3_^−^). FT-IR (selected bands in cm^−1^): 3037 ν(N^+^–CH_3_), 2997 ν(CH_3_), 1732 ν(C=O), 1149 ν_as_(SO_3_^−^), 1037ν_s_ (SO_3_^−^). Elemental analysis (C_14_H_21_NO_4_S, *M*_r_ = 299.39): calculated: C = 56.17%, H = 7.07%, N = 4.68%, S = 10.71%, O = 21.38%; found: C = 52.75%, H = 7.41%, N = 4.39%, S = 10.24%. TGA: onset of decomposition at 280 °C.

### 2.3. Instrumentation and Methods

^1^H and ^13^C NMR spectra, ^1^H-^1^H-Correlation Spectra (COSY) and ^1^H-^13^C-Hetero-nuclear Multiple Quantum Coherence spectra (HMQC) were recorded with an Avance 300 spectrometer (Bruker, Billerica, MA, USA, 300 and 75 MHz, respectively) at ambient temperature in deuterated solvents. ^13^C spectra were recorded in ^1^H-broad band decoupling mode and Attached Proton Test (ATP) mode respectively. Solvent signals or 3-(trimethylsilyl) propionic-2,2,3,3-d_4_ acid sodium salt were used as internal shift references. High resolution mass spectra (HR-MS) were recorded with a Thermo Scientific ESI-Q-TOF micro (quadrupol—time of flight) (Thermo Fisher Scientific, Waltham, MA, USA) with electrospray ionization (ESI) using water as solvent. Element analysis was conducted by using a Vario ELII microanalyzer (Elentar Analysensysteme, Hanau, Germany). FT-IR spectra were recorded in a N_2_ purged atmosphere with a Nicolet Nexus FT-IR spectrometer (Thermo Fisher Scientific) equipped with an attenuated total reflection (ATR) smart endurance element. Thermogravimetric analysis (TGA) was conducted under N_2_ purged atmosphere using a TG 209 F1 apparatus (Netzsch Gerätebau GmbH, Selb, Germany), in the temperature range from 25 to 900 °C with a heating rate of 10 K·min^−1^. Size exclusion chromatography (SEC) for polyzwitterions was run with an apparatus SEC3010 (WGE—Dr. Bures, Dallgow-Döberitz, Germany) equipped with a refractive index detector and PL-HFIP gel columns (Agilent Technologies, Santa Clara, CA, USA), using hexafluoroisopropanol (HFIP) containing 50 mM of sodium trifluoroacetate as eluent (flow rate 0.8 mL·min^−1^), and poly(methylmethacrylate) “PMMA” standards (500 to 520,000 Da, narrowly distributed, PSS Polymer Standard Service, Mainz, Germany) for calibration. Size exclusion chromatography (SEC) for **P-OEGMA** was run with an apparatus Thermo Finnigan Spectra System 1000 (Thermo Fisher Scientific) equipped with a refractive index detector Wyatt Optilab DSAP and GRAL/GRAM columns (Polymer Standard Service), using NMP containing 5 g·L^−1^ LiBr as eluent (flow rate 0.5 mL·min^−1^), and linear polystyrene standards (682 to 2,520,000 Da, narrowly distributed, PSS Polymer Standard Service) for calibration.

For investigating the stability in aqueous media, the compounds were recorded in given time intervals by NMR spectra in D_2_O at different pH values at ambient temperature (22 ± 1 °C). The concentration of the monomers was 0.1 mol·L^−1^ throughout the experiments, except for **M-5** and **M-6**, which have a lower solubility in the buffer solutions used, and thus were dissolved only until saturation. The aqueous media employed are namely phosphate buffered saline (PBS, pH = 7.4), 1 M sodium carbonate—sodium bicarbonate buffer (pH = 10), 1 M aqueous sodium hydroxide (pH = 14) and 1 M deuterium chloride solution (pH = 0). Analogously, the concentration of the polymers was set to 0.1 mol/L with respect to the constitutional repeat unit (CRU). As however only polymers **P-OEGMA** and (barely) **P-SPP** were soluble at room temperature in pure water, specific amounts of NaCl were added to the other polymer systems to enable their dissolution. Notably in the case of the polysulfabetaines, solubility was so low that half saturated or even saturated aqueous NaCl was needed to obtain homogenous polymer solutions at 22 °C.

## 3. Results

### 3.1. Synthesis of the Monomer and Polymers, and Their General Aqueous Solution Behavior

As sulfabetaine monomers are not commercially available, monomers **M**-**1**–**M**-**6** were prepared by a standard strategy, namely by ring opening alkylation of commercially available tertiary amine precursor monomers with the cyclic ethylene or propylene sulfate diesters [20,55], adapting published procedures [46,47]. In particular, the reaction conditions were slightly modified by using a 10% molar excess of the tertiary amine precursors, and by prolonging reaction times in comparison to the literature, in order to assure full consumption of the cancerogenic cyclic sulfates. When the reaction is carried out in acetonitrile, the zwitterionic products precipitate readily, and their separation and purification is straightforward. Analytical data (from elemental analysis, mass spectrometry, and ^1^H-NMR spectroscopy) agreed well with the literature data available [46,47], and were complemented by their ^13^C-NMR and infrared spectra (see also Electronic Supporting Information).

The various zwitterionic monomers could be smoothly converted into their homopolymers via free radical polymerization in a homogeneous solution in trifluoroethanol (TFE), which is one of the few effective solvents for most zwitterionic monomers and their polymers [20,51]. Key analytical data of the polymers are summarized in Table 1. The apparent molar masses were in the range of 130 to 420 kg·mol^−1^, and dispersities Ð were in the range of ~2 to ~4, in agreement with a standard free radical polymerization process conducted up to high yields. The rather high molar masses of the samples aggravated the problem of the generally low solubility of most polyzwitterions in pure water (Table 1). In fact, only the polysulfobetaines **P**-**SPE** and **P**-**SPP** were soluble in pure water at all, and even they show a miscibility gap at low temperatures, that is, UCST behavior. The measured cloud points (CP) agree well with recently reported data [54,56]. In marked contrast, the hypothetical CPs of all polysulfabetaines studied are above 100 °C in pure water.

Polymers **P-1**–**P-6** require high concentrations of added salt to form aqueous solutions, normal saline solution not being sufficient, in agreement with the few literature reports existing [39,46,47]. The reduced water-solubility of polysulfabetaines compared to polysulfobetaines is in agreement with molecular modelling studies [57].

Ranking qualitatively the solubility in aqueous media via the minimum amount of NaCl necessary for dissolution, we find that the polysulfabetaines with poly(methacrylic) backbones **P-1**–**P-4** dissolve more easily than the analogous poly(vinylbenzylammonium) derived polyzwitterions **P-5** and **P-6**. In fact, **P-5** is insoluble even in saturated brine. This order might have been anticipated by common chemical wisdom. Still, it implies that the presumably particularly hydrolytically stable polyzwitterions **P-5** and **P-6** disposing of a polystyrene backbone [46,48] may be of little use in practice when water-soluble systems are needed. Moreover, we corroborate that the ammoniopropylsulfates, such as **P-2**, **P-4**, and **P-6**, dissolve more easily than the analogous ammonioethylsulfates, such as **P-1**, **P-3**, and **P-5** [46,58]. The finding that the zwitterions, in which the ammonium nitrogen and the anionic moieties are separated by a trimethylene spacer, are more soluble than the ones separated by dimethylene spacer is not self-evident, but seems to parallel the higher water-solubility of ammoniopropanesulfonates over their ammonioethylsulfonate analogs [59]. Due to the virtual insolubility of **P-5** in any aqueous medium studied at ambient temperature, even at maximum sodium chloride concentrations, this polymer was excluded from the further investigations.

In contrast to the polyzwitterions, the non-ionic reference **P-OEGMA** is soluble in all aqueous media studied here at room temperature, but shows a rather high cloud point of the lower critical solution temperature (LCST) type. The CP value of 85 °C in pure water agrees well previous reports [60], and decreases increasingly when substantial amounts of salt are added so that the polymer becomes insoluble in saturated brine (Table 1).

### 3.2. Investigation of Polymer Degradation in Aqueous Solution

The stability of the zwitterionic monomers and polymers in aqueous solution at various pH values was followed at room temperature (22 ± 1 °C) via ^1^H NMR spectroscopy. Beyond tracking and potentially quantifying ongoing degradation, NMR spectroscopy also offers the chance to identify the degradation products and, therefore, possibly to learn not only about the kinetics but also about the mechanism on the reaction(s) occurring. Whereas for low molar mass compounds, such as the monomers, signal resolution—and therefore the amount of information that can be derived from the spectra—is typically high, polymers exhibit inherently broad NMR signals and reduced resolution. On the one hand, this handicaps the identification and quantification of polymeric degradation products. On the other hand, low molar mass molecules that are released give sharp signals, which are detected already for trace amounts.

**Figure 2:**
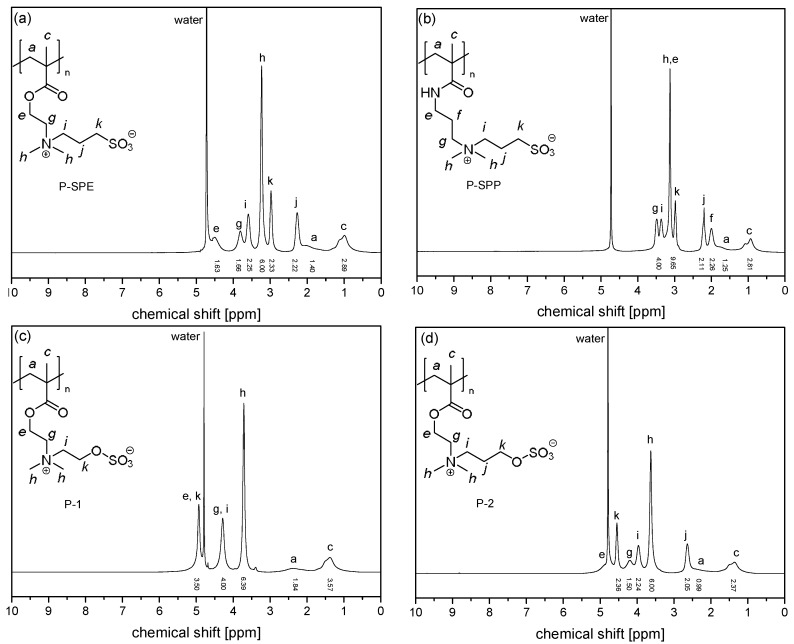
^1^H NMR spectra in D_2_O containing NaCl of (**a**) **P-SPE** (9.0 g·L^−1^ NaCl), (**b**) **P-SPP** (9.0 g·L^−1^ NaCl), (**c**) **P-1** (saturated solution), and (**d**) **P-2** (saturated solution).

**Figure 3:**
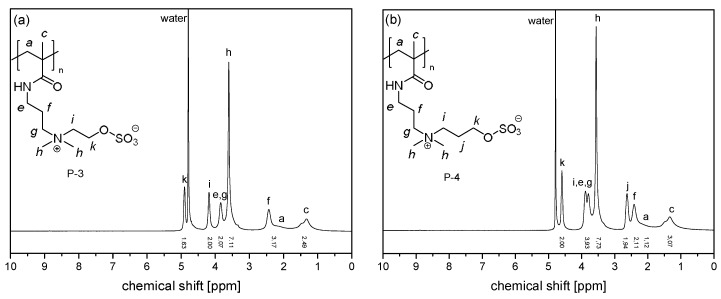

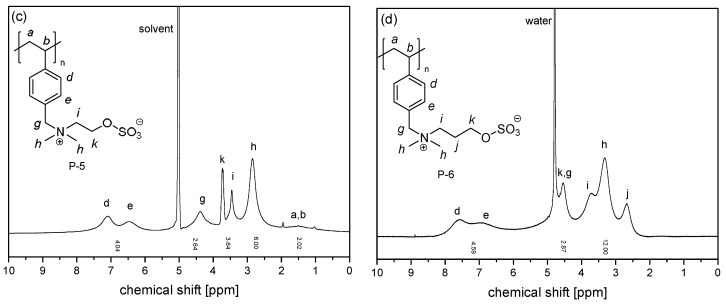
^1^H NMR spectra of (**a**) **P-3** in D_2_O saturated with NaCl, (**b**) **P-4** in D_2_O saturated with NaCl, (**c**) **P-5** in TFE-d_3_, (**d**) **P-6** in D_2_O saturated with NaCl.

Initial spectra of the intact monomers and polymers were recorded in D_2_O (pH~5.8) prior to the ageing experiments, to which the necessary amounts of NaCl were added to achieve solubility at 22 °C (Figure 2 and Figure 3). Only in the case of polysulfabetaine **P-5** that was insoluble even in saturated brine, the reference spectrum was recorded in deuterated TFE (Figure 3c). Despite the broad peaks and the limited resolution, all signals can be easily attributed to specific protons by comparison with the monomer spectra as well as with literature reports [47,54,56,60]. Note that for the methacrylic polymers **P-OEGMA**, **P-SPE**, **P-SPP**, and **P-1** to **P-4**, the shape of the signal group between 1.5 and 0.7 ppm—that is characteristic for the methyl group on the polymer backbone—informs about the polymers’ tacticity [61], indicating that syndiotactic triades prevail in the samples, while the amount of isotactic triades is very small. This distribution of triades is typical for such polymers when made by standard radical polymerization in solution [54,56].

**Figure 4:**
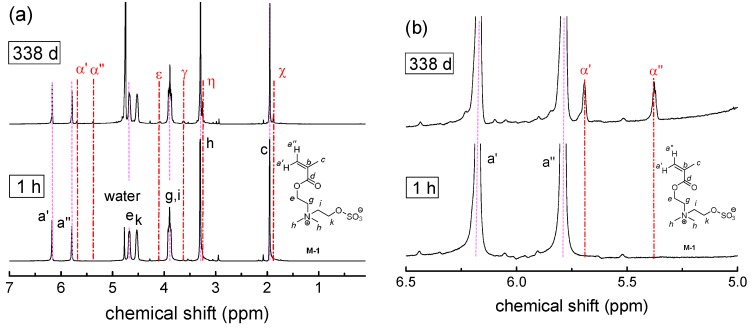

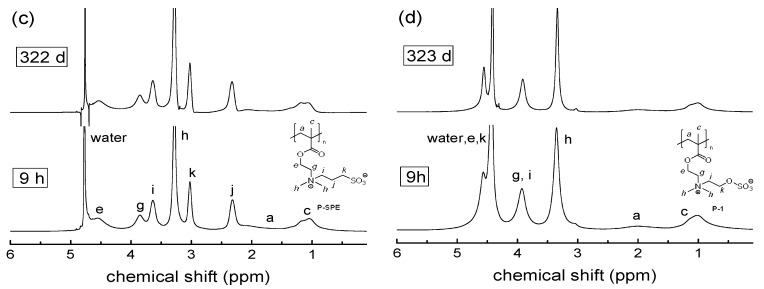
Evolution of the ^1^H-NMR spectra over time illustrating the degradation behavior in phosphate buffered saline (PBS) in D_2_O (pH = 7.4) at room temperature for: (**a**) **M-1**; (**b**) Inset **M-1**, (**c**) **P-SPE**; (**d**) **P-1**. Signal attributions of the starting compounds are labeled by roman letters, signal attributions of the degradation product by corresponding Greek letters (α, β, χ, δ, ε, φ, γ, η, ι, ϕ, and κ corresponding to a, b, c, d, e, f, g, h, i, j, and k, respectively). Broken vertical lines are meant as guide to the eye.

Then, long-term stability of the polymers in aqueous solution at the physiologically important pH value of 7.4 was studied for a period of up to one year. The underlying monomers were also investigated for comparison. Under these conditions, we found degradation to be very slow, if occurring at all (Figure 4). For the methacrylate monomers **SPE**, **M-1**, **M-2**, and **OEGMA,** the most prominent changes are on the one hand the small, gradual intensity loss of the signals at about 4.6, 5.8, and 6.2 ppm, which are characteristic for the –COO–CH_2_– group and for the two vinylidene protons, respectively. On the other hand, small new signals gradually evolve at about 3.9, 5.4, and 5.7 ppm. All the changes in the spectra are indicative for the minor hydrolysis of the ester moiety yielding the methacrylate anion and the zwitterionic alcohol (or oligoethylene glycol in the case of **OEGMA**). In contrast, for the methacrylamide monomers **SPP**, **M-3**, and **M-4**, as well as for the styrene derivatives **M-5** and **M-6**, no indication for degradation is found after one year. Importantly, none of the polymers showed any indication for degradation, irrespective of the presence or absence of carboxylic ester bonds in their structure. Whereas small amounts of polymer bound degradation products may be missed in the NMR spectra, any low molar mass product split from the polymer, such as an alcohol or an amine, can be sensitively detected. As no traces of sharp peaks arise in the spectra superposing the broad polymer signals, we state that all polymers are fully stable even for the longest ageing time. Figure 4 illustrates the behavior for selected samples.

**Figure 5:**
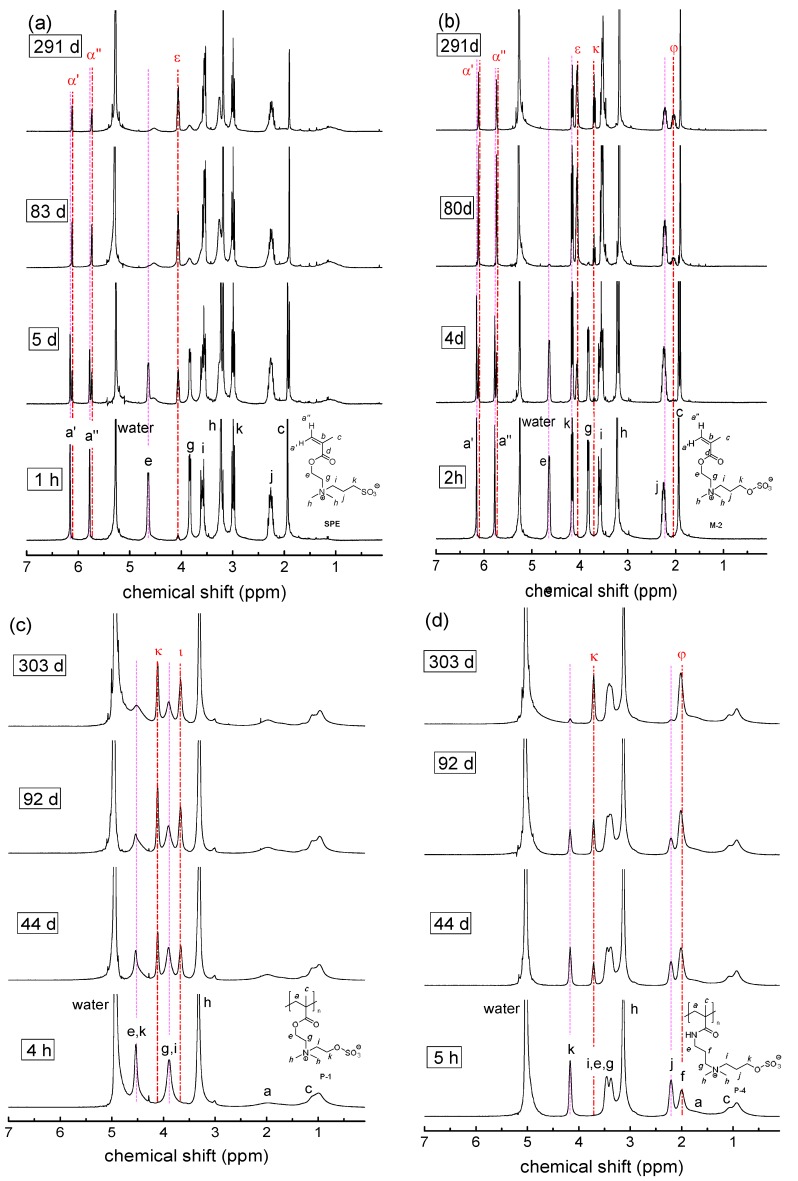
Evolution of the ^1^H-NMR spectra over time illustrating the degradation behavior in 1 M hydrochloric acid (DCl in D_2_O, pH = 0) at room temperature for: (**a**) **SPE**; (**b**) **M-2**, (**c**) **P-1**; (**d**) **P-4**. Signal attributions of the starting compounds are labeled by roman letters, signal attributions of the degradation product by corresponding Greek letters (*cf.* Figure 4). Broken vertical lines are meant as guide to the eye.

**Figure 6:**
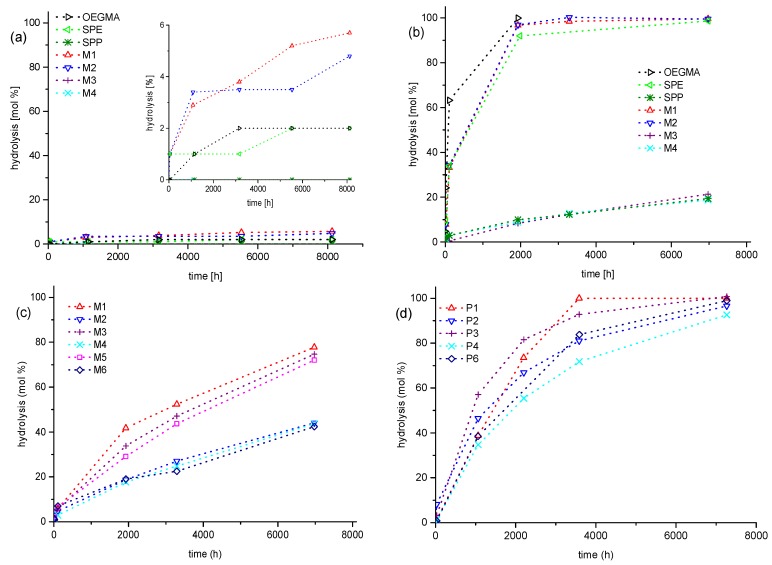
Kinetics of ester and amide hydrolysis for monomers **SPE** (◁), **SPP** (✴), **M-1** (△), **M-2** (▽), **M-3** (+), **M-4** (✕), and **OEGMA** (**▷**): (**a**) phosphate buffered saline (PBS) in D_2_O (pH = 7.4) and (**b**) in 1 M DCl in D_2_O (pH = 0); kinetics of sulfate hydrolysis for sulfabetaines in 1 M DCl in D_2_O (pH = 0): (**c**) monomers **M-1** (△), **M-2** (▽), **M-3** (+), **M-4** (✕), **M-5** (☐), **M-6** (**◇**) and (**d**) polymers **P-1** (△), **P-2** (▽), **P-3** (+), **P-4** (✕), **P-5** (☐), **P-6** (**◇**).

Having revealed the long-term inertness of the polymers in PBS, that is, under mild conditions, we investigated next the stability under strongly acidic conditions that are known to catalyze the hydrolysis of ester and amides groups. Figure 5 exemplifies the observed degradation behaviors in 1 M hydrochloric acid (pH = 0) for selected samples. Qualitatively, we observe the same changes in the spectra as found in PBS for the methacrylate monomers, but the evolution is much faster. The acid catalyzed hydrolysis of the carboxylate ester groups of the zwitterionic monomers reaches virtually completion after about 2000 h, that is, after about 12 weeks (Figure 6). Under the strongly acidic conditions, the amide groups of the monomers are also attacked, albeit hydrolysis is considerably slower than for the esters (Figure 6b). Note that the changes in the NMR spectra of the methacrylamides are visually less prominent than in the case of the methacrylate esters, as certain signals of functional protons are superimposed. Nevertheless, characteristic changes in the spectra are the gradually attenuated signals at about 3.4 and 5.45 which are characteristic for the –C(=O)N–CH_2_– group and for the *trans*-vinylidene proton of the methacrylamide moiety, respectively (see Supporting information). In parallel, new signals evolve at about 3.2 and 6.2 ppm, while the intensity of the vinylidene proton signal at 5.75 remains constant. This latter, a surprising finding at a first view, is due to the superposition of the signals of the *cis*-vinylidene proton of the methacrylamide moiety and the *trans*-vinylidene proton of methacrylic acid. Again, the various changes observed in the spectra are indicative for the hydrolysis of the amide moiety into methacrylic acid and the protonated zwitterionic amine. Amide hydrolysis amounts to about 20% after one year, whereby no difference is noted between the sulfobetaine methacrylamide **SPP** and the sulfabetaine ones **M-3** and **M-4** (Figure 6b).

In flagrant contrast to the behavior of the monomers, we do not detect any degradation of the amide and even of the carboxylate ester bonds for the polymers even after one year at pH = 0. However, we see for both the monomers and polymers which bear sulfabetaine moieties, that the signal at about 4.2 ppm indicative of the –CH_2_–O–SO_3_^−^ group is gradually attenuated, while a new signal at 3.7 ppm rises in parallel that is indicative of a primary alcohol moiety (Figure 5c,d). This indicates that the sulfate ester of the sulfabetaine moiety is hydrolyzed. Whereas after one year, the degree of hydrolysis attains 40% for the ammoniopropylsulfate monomers **M-2, M-4**, and **M-6**, hydrolysis reaches 75% the analogous ammonioethylsulfate monomers **M-1**, **M-3**, and **M-5** (Figure 6c). Interestingly, the hydrolysis of the hemisulfate groups in the sulfabetaine polymers is somewhat faster than in their monomers, and reaches full degradation within one year. The ammonioethylsulfates degrade again faster than their ammoniopropylsulfate analogues (Figure 6d), but the difference is less pronounced than for the monomers.

The various findings summarized in Figure 6 provide a comprehensive picture of the hydrolytic stability of the compounds under neutral and acidic conditions. On the one hand, the carboxylate ester and amide moieties of the polymethacrylates and -methacrylamides are extremely stable, outperforming the stability of the underlying monomers by far. Furthermore, the polymeric carboxylate ester and amide moieties are considerably more stable than the hemisulfate moiety, independent of whether the latter is attached to monomers or polymers. Clearly, the stability of the ester moiety of the polymethacrylates, which is attached directly to the backbone, is much higher than the one reported for ester moieties placed in the side chains of methacrylic polymers well separated from the backbone [62,63,64]. This remarkable finding goes along with previous reports that polymethacrylates are much less sensitive to hydrolysis than their polyacrylate analogs [32,33,34,35,37]. This may be explained by steric effects. When attached to the polymer backbone, the carboxyl functions are effectively shielded against attack by water molecules due to the neighboring methyl groups, which create a highly crowded local environment. Possibly, the low polarity of this environment also restrains protonation of the carboxyl groups, thus limiting acid catalysis and enhancing the stability. In contrast, the hemisulfate moieties are localized well apart from the polymer backbone, and thus, cannot profit from a protective steric effect, leaving them exposed to acid catalyzed hydrolytic attack.

**Figure 7:**
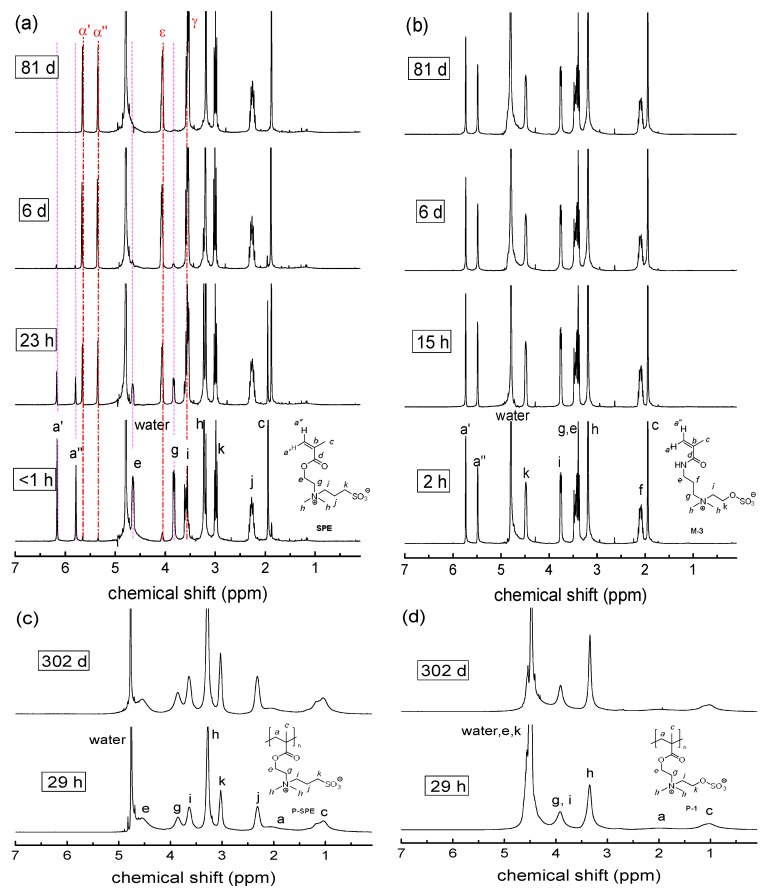
Evolution of the ^1^H-NMR spectra over time illustrating the degradation behavior in carbonate buffer in D_2_O (pH = 10) at room temperature for: (**a**) **SPE**; (**b**) **M-3**, (**c**) **P-SPE**; (**d**) **P-1**. Signal attributions of the starting compounds are labeled by roman letters, signal attributions of the degradation product by corresponding Greek letters. Broken vertical lines are meant as guide to the eye.

**Figure 8:**
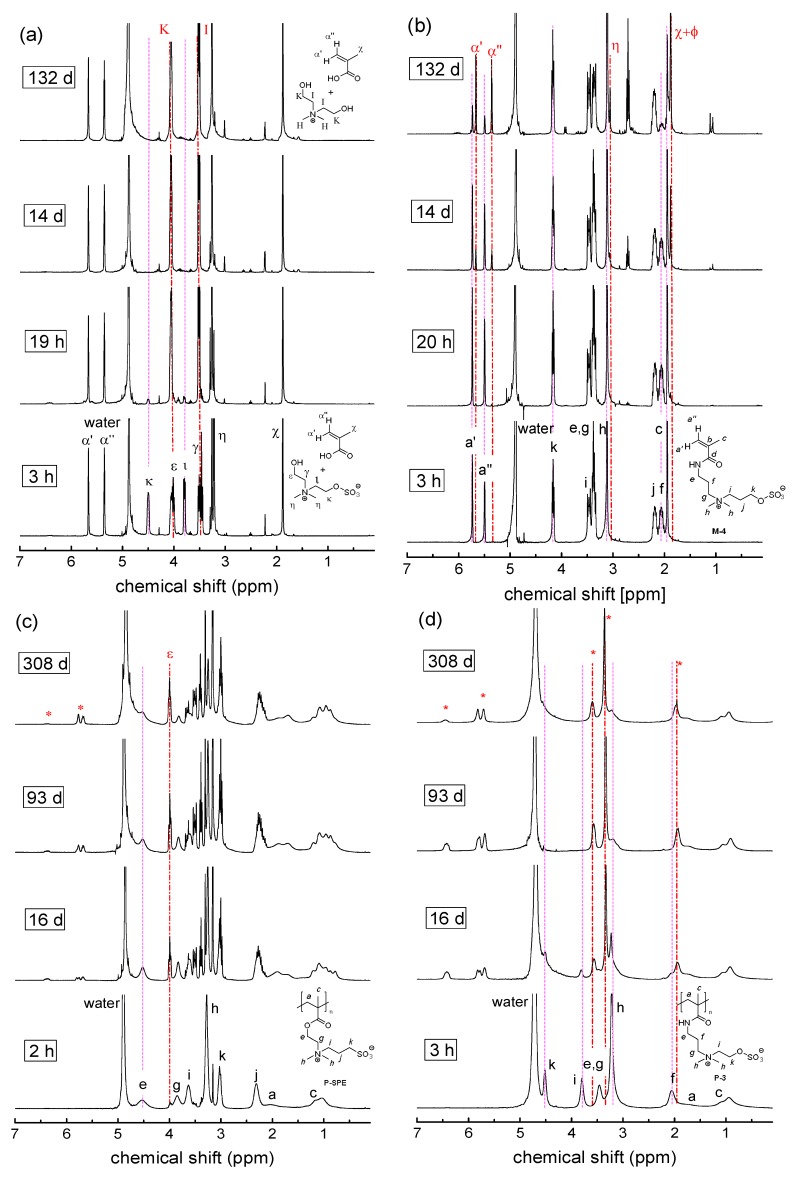
Evolution of the ^1^H-NMR spectra over time illustrating the degradation behavior in 1 M sodium hydroxide in D_2_O (pH = 14) at room temperature for: (**a**) **M-1**; (**b**) **M-4**, (**c**) **P-SPE**; (**d**) **P-3**. Signal attributions of the starting compounds are labeled by roman letters, signal attributions of the degradation product by corresponding Greek letters. Broken vertical lines are meant as guide to the eye.

**Figure 9:**
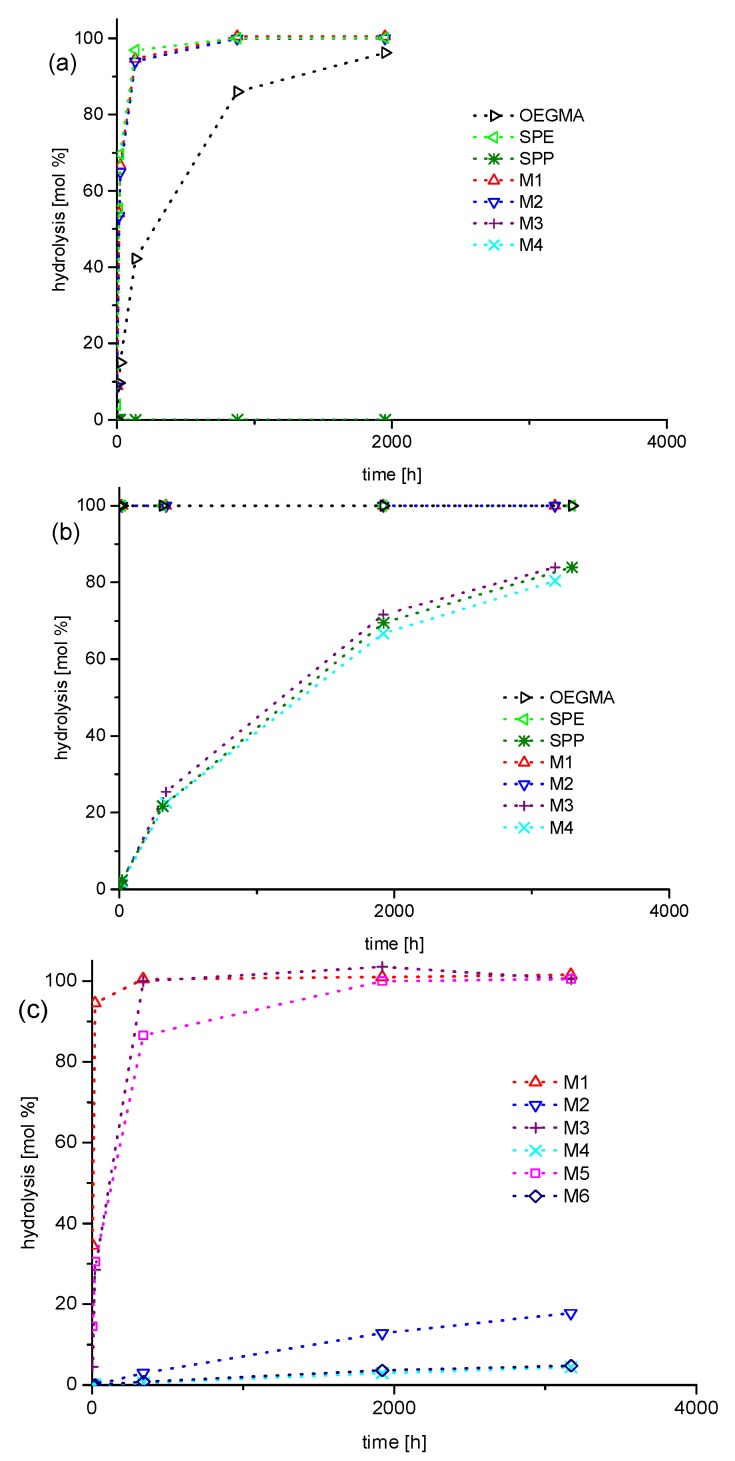
Kinetics of ester and amide hydrolysis for monomers **SPE** (◁), **SPP** (✴), **M-1** (△), **M-2** (▽), **M-3** (+), **M-4** (✕), and **OEGMA** (**▷**): (**a**) in carbonate buffer in D_2_O (pH = 10) and (**b**) in 1 M sodium hydroxide in D_2_O (pH = 14); (**c**) kinetics of sulfate hydrolysis for sulfabetaine monomers **M-1** (△), **M-2** (▽), **M-3** (+), **M-4** (✕), **M-5** (☐), and **M-6** (**◇**) in 1 M sodium hydroxide in D_2_O (pH = 14).

Finally, the stability of the monomers and polymers against degradation under basic conditions was investigated (Figure 7, Figure 8 and Figure 9). As ester and amide groups are generally known to be more sensitive to the hydrolysis under the action of base than of acid, we studied two levels of basic conditions, established by a bicarbonate/carbonate buffer with pH = 10 (Figure 7), and by 1 M sodium hydroxide solution with pH = 14 (Figure 8).

In carbonate buffer with pH = 10, the general picture of relative stabilities resembles qualitatively the findings in PBS, while degradation effects occur markedly faster (compare Figure 6a with Figure 9a). For the monomers, we observe the accelerated hydrolysis of the carboxylate ester group (Figure 7a), which is virtually complete after 1000 h for the zwitterionic methacrylates, and after 2000 h for the nonionic **OEGMA**. In contrast, we did not detect degradation of the methacrylamide moieties even after one year (Figure 7b). Also, the sulfate esters of the sulfabetaine monomers stayed intact over this period. Furthermore, all the polymers, the polymethacrylates included, did not show any sign of degradation (Figure 7c,d), demonstrating once more the high stability of poly(methacrylic) systems against hydrolysis.

In highly alkaline solution however, at pH = 14, degradation became visible for most compounds studied (Figure 8), but the stabilities of the various systems diverge strongly. The ester groups of the methacrylate monomers are completely hydrolyzed in less than 3 h (Figure 8a and Figure 9b). Also the methacrylamide monomers suffer hydrolysis (Figure 8b), but much more slowly, reaching about 80% degradation after four months (Figure 9b). Concerning the polymerizable groups, only the styrene moiety of monomers **M-5** and **M-6** survived one year of storage at pH = 14 unharmed. However, the hemisulfate moiety of the sulfabetaine monomers proved to be vulnerable to strong base (Figure 8b,d), with its stability depending sensitively on the distance between the ammonium and the sulfate groups (Figure 9c). The ammoniopropylsulfates showed only slow degradation of the hemisulfate moiety via hydrolysis (<50% after one year) leaving the ammonium moiety apparently untouched. In contrast, the ammonioethylsulfates decomposed readily within about 20 days by seemingly a number of different reactions in addition to plain hydrolysis. The new signals evolving nearby the original signal of the dimethylammonium group at about 3.1 ppm (Figure 8b,d), indicate that degradation involves at least partially also the vicinity of the ammonium nitrogen. Together with the new signals between 5.2 and 6.5 ppm, a region characteristic for olefinic protons, this degradation pathway seems to proceed via elimination of the hemisulfate, producing the vinylammonium moiety. In fact, inorganic esters of choline were reported to eliminate under basic conditions to neurine [65], and the elimination of hemisulfate in β-position to other strongly electron withdrawing groups, such as sulfones, is well established to yield vinylsulfones [66].

Also, most of the polymers show some degradation under such harsh conditions. Only for the polysulfobetaine **P-SPP**, the ammoniopropylsulfates **P-4** and **P-6**, as well as reference **P-OEGMA**, no indication for hydrolysis was noted (see Supporting Information). In the case of the zwitterionic polymethacrylates however, the carboxylate esters degrade, though more slowly than their monomers, and low molar mass degradation products become visible already after 2 h (Figure 8c). In contrast, we do not detect any similar low molar mass degradation products for the polymethacrylamides, and in particular, the amide bonds remain still untouched even after one year. Interestingly, the spectra show that the action of the strong base on the polymethacrylates does not only induce degradation by standard ester hydrolysis, which is indicated by the sharp triplet signal evolving continuously at about 3.9 ppm that is indicative for the formation of the low molar mass zwitterionic primary alcohol cleaved from the polymer. Apart from a plethora of sharp signals in the range of 3.8–2.9 ppm, also broadened and rather weak new signals evolve in the region of 5.5–6.6 ppm. This region is characteristic for olefinic protons, while the marked signal broadening suggests that these new groups are polymer bound. We therefore presume, in analogy to the behavior of the ammonioethylsulfates discussed above, that these weak signals derive in the case of **P-SPE** from a Hofmann elimination that produces vinyl esters, which slowly degrade further. Unfortunately, the multitude of new signals and the superposition of many signal groups of the polymers as well as of the various degradation products, made it difficult to quantify their amounts for establishing precise degradation kinetics.

As found for their monomers, the stability at high pH of the sulfate esters of the various sulfabetaine polymers **P-1**–**P-4** and **P-6** depended sensitively on the distance between the ammonium and the hemisulfate groups. The ammonioethylsulfate moieties decomposed rapidly. Already after 24 h, new broad signals in the region of 5.5–6.8 ppm are observed, which for instance, together with the new signal at about 3.0 ppm suggest the formation of vinylammonium moieties, as discussed above for the corresponding monomers. In contrast, we did not observe noticeable degradation of the ammoniopropylsulfate moiety after 1 year at pH 14 in the case of the polymethacrylamide **P-4** and the polystyrene derivative **P-6**. Nevertheless, it must be kept in mind that the degradation of the hemisulfate moiety (e.g., by conventional hydrolysis) does not result necessarily in low molar mass degradation products visible in the ^1^H NMR spectra, diminishing the sensitivity of the analysis. Very weak new signals in the region of 5.5 to 6.4 ppm in the case of **P-4** point to traces of olefinic degradation products formed. Polymethacrylate **P-2** bearing ammoniopropylsulfate groups showed however clear signs of degradation, evidenced by the gradual rise of new signals in the region of 5.5–6.8 ppm. Due to the complexity of the spectra, it could not be determined to which extent this was the consequence of degradation of the carboxylate or of the sulfate ester groups, respectively. This may be an interesting point to elucidate in the future, because if the former process dominates, the stability against strong base of poly(sulfobetaine methacrylate)s such as **P-SPE** or **P-2** might be improved by simple means. If replacing the parent 2-dimethylaminoethyl methacrylate building block by longer dimethylaminoalkyl homologues [52,54,67,68], the presumed facilitated Hofmann elimination of the 2-ammonioethylester moiety should be overcome. In any case, no indication for degradation of the ammoniopropanesulfonate moiety of the sulfobetaines is found.

Summarizing the findings for the stability of the polymers under basic conditions, we state the general weakness of the ammonioethylsulfate moiety at high pH, whereas the ammoniopropylsulfonate and largely also the ammoniopropanesulfate moieties are stable. Further, we observe again a strongly enhanced stability of the methacrylic polymers compared to their monomers. In particular, not only the zwitterionic polystyrene derivative **P-6**, but also both the sulfobetaine polymethacrylamide **P-SPP** and the sulfabetaine polymethacrylamide **P-4** were found to be extremely stable. Even the polymethacrylate **P-OEGMA** turned out to be stable under strongly alkaline conditions. This is in striking contrast to the notable degradation of the various zwitterionic polymethacrylates, which does not follow a pure basic ester cleavage mechanism. We may speculate that the difference may on the one hand be due to the particularly high steric crowding of the carboxyl group in the case of the polymerized macromonomer **OEGMA** that may be already considered as a molecular bottle brush [69], which protects the carboxylate esters. On the other hand, electrostatic interaction with the cationic ammonium sites might increase the local concentration of the hydroxide anions, and favor their attack of the ester bonds. Alternatively, a specific interaction of the ammonium group with the carbonyl group in quaternized derivatives of 2-dimethyaminoethyl methacrylate via a loop-like conformation has been discussed [70,71], which also might facilitate the attack of the ester bond in the polyzwitterions by hydroxide ions.

## 4. Conclusions

In contrast to their monomers, zwitterionic methacrylic polymers as well as their oligoethylene glycol analog, which are largely used for antifouling purposes, appear stable against hydrolytic degradation at ambient temperature for extended periods for as long as one year, minimum. This remarkable stability is shown not only for neutral, but also for strongly acidic and moderately basic pH values, and applies equally to zwitterionic polymethacrylic esters and amides. According to our findings, the occasional reports about partial hydrolysis of related polymers are due to degradation of the monomers prior to or during polymerization, but not due to hydrolysis of the final polymers. Putatively, the high hydrolytic stability of the methacrylic polymers is due to the protection of the carboxyl groups against attack by water molecules by virtue of the high local steric crowding. Only at strongly alkaline conditions, at the pH of 14, the zwitterionic polymethacrylates start to degrade after a few hours, while the polymethacrylamides and also poly(oligoethylene glycol methacrylate) remain intact. Such strongly basic- or acidic-conditions seem unrealistic for most antifouling applications, but might occur in certain cleaning or disinfection protocols. According to our findings, acidic conditions should be preferred over alkaline ones, if such extreme pH values are necessary.

Moreover, the recently proposed need of particular, more hydrolysis resistant polymer backbones, such as polystyrenes, for anti-fouling purposes seems unnecessary and might be even be detrimental, in particular when experiencing the very poor water-solubility of many such polyzwitterions. Concerning the stability of the zwitterionic moieties, sulfobetaine moieties were corroborated to be fully resistant even to extreme pH scenarios. In contrast, the hemisulfate groups of the recently proposed sulfabetaine alternatives are potentially weak spots. While being stable at moderate pH values, they decompose markedly in acid at ambient temperature. In contrast, in strong base as 1 M NaOH, the poly(ammoniopropylsulfate)s proved to be stable, whereas the poly(ammonioethylsulfate)s degraded rapidly. The difference is attributed to a second degradation pathway instead of conventional sulfate ester hydrolysis, namely the facilitated base-catalyzed elimination of sulfate thus producing neurine derivatives, when a dimethylene spacer separates the ammonium and the sulfate groups.

Altogether, our findings demonstrate the basic suitability of zwitterionic polymetharylates and -methacrylamides, in particular of polysulfobetaines, for anti-fouling applications even for extended periods of use.

## Figures and Tables

**Table 1:** Molar masses of the polymers prepared, and their solubility (3 wt %) in various aqueous solvents.

Polymer	*M*_n_^app^ (kg·mol^−1^) ^a^	Dispersity Ð ^a^	CP in water (°C)	CP in normal saline (°C) ^b^	CP in PBS (°C) ^c^	CP in brine (°C) ^d^
**P-OEGMA**	130 ^e^	3.8 ^(e)^	85 ^f^	80 ^f^	79 ^f^	^j^
**P-SPE**	210	2.2	55 ^g^	<0	<0	<0
**P-SPP**	180	2.2	21 ^g^	<0	<0	<0
**P-1**	400	1.8	^h^	^h^	^h^	<0
**P-2**	420	1.6	^h^	^h^	^h^	<0
**P-3**	^j^	^j^	^h^	^h^	^h^	<0
**P-4**	220	2.3	^h^	^h^	^h^	<0
**P-5**	^j^	^j^	^h^	^h^	^h^	^j^
**P-6**	^j^	^j^	^h^	^h^	^h^	<0

^a^ apparent number average molar mass *M*_n_^app^ and dispersity Ð (*M*_w_^app^/*M*_n_^app^) by SEC, eluent HFIP, calibration with linear poly(methylmethacrylate) standards; ^b^ 9.0 g/L NaCl in water; ^c^ phosphate buffered saline; ^d^ saturated aqueous NaCl solution; ^e^ eluent NMP, calibration with linear polystyrene standards; ^f^ cloud point CP with lower critical solution temperature (LCST); ^g^ cloud point CP with upper critical solution temperature (UCST); ^h^ not soluble; ^j^ swells only in solvent/eluent.

## Supplementary Materials

The following are available online at http://www.mdpi.com/2073-4360/10/6/639/s1: procedures for preparing buffer solutions; detailed ^1^H- and ^13^C-NMR spectroscopic characterization of the monomers (Figures S1–S13), and of the polymers, (Figures S14–S20) prior to storage; full set of ^1^H-NMR spectra of the monomers and polymers taken after precise intervals of storage for specific pH conditions (Figures S21–S102).
