# Supplementary material for: Exploring the Long-Term Hydrolytic Behavior of Zwitterionic Polymethacrylates and Polymethacrylamides
Source: Polymers (Basel). 2018 Jun 8;10(6):639. doi: 10.3390/polym10060639 (PMC6403559; doi:10.3390/polym10060639)
Supplement: Supplementary file 1 [file polymers-10-00639-s001.pdf]

# Supporting Information

## Exploring the Long-Term Hydrolytic Behavior of Zwitterionic Polymethacrylates and Polymethacrylamides

Eric Schönemann,[a] André Laschewsky,[a,b,*] Axel Rosenhahn [c,#]

[a] *Institut für Chemie, Universität Potsdam, Karl-Liebknechtstr. 24-25, 14476 Potsdam-Golm, Germany. E-mail: laschews@uni-potsdam.de*

[b] *Fraunhofer Institute for Applied Polymer Research IAP, Geiselbergstr. 69, 14476 Potsdam-Golm, Germany*

[c] *Analytische Chemie - Biogrenzflächen, Ruhr-Universität Bochum, Universitätsstr. 150, 44801 Bochum, Germany*

*Corresponding Author: E-mail: laschews@uni-potsdam.de, phone +49 331 997 5225, Fax +49 331 997 5036. ORCID number 0000-0003-2443-886X

[#] ORCID number 0000-0001-9393-7190

## Contents

1. Detailed 1H- and 13C-NMR spectroscopic characterization of the monomers ............3
2. Detailed 1H- and 13C-NMR spectroscopic characterization of the polymers ............15
3. Preparation of buffer solution........................................................................23
4. Evolution of the monomer and polymer 1H-NMR spectra upon storage in aqueous media at 22 °C at various pH values. .............................................................25
    4.1. Monomer hydrolysis in phosphate buffered saline (pH = 7.4) .......................25
    4.2. Monomer hydrolysis in 1 M hydrochloric acid pH=0 ....................................36
    4.3. Monomer hydrolysis hydrogen carbonate buffer (pH=10) .............................53
    4.4. Monomer hydrolysis in 1 M sodium hydroxide solution (pH=14) .................64
    4.5. Polymer hydrolysis in phosphate buffered saline (pH = 7.4) .........................81
    4.6. Polymer hydrolysis in 1 M hydrochloric acid pH=0......................................85
    4.7. Polymer hydrolysis hydrogen carbonate buffer (pH=10) ...............................95
    4.8. Polymer hydrolysis in 1 M sodium hydroxide solution (pH=14) ...................99

**1. Detailed 1H- and 13C-NMR spectroscopic characterization of the monomers**

*2-(N-(2-(methacryloyloxy)ethyl)-N,N-dimethylammonio)ethyl sulfate (**M-1**)*

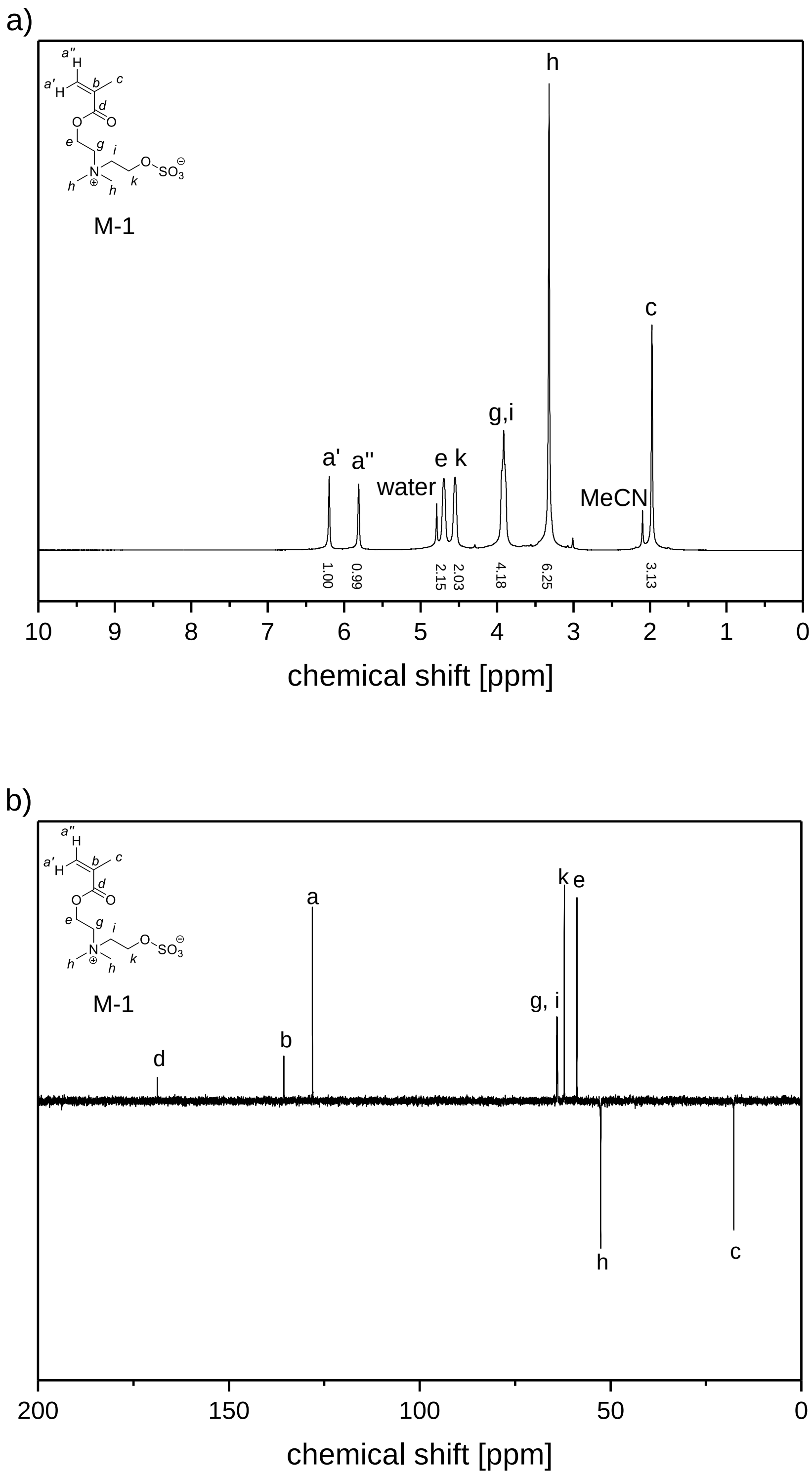


Figure S 1 a) $^{1}H$ NMR (in $D_2O$) and b) $^{13}C$ (APT) NMR spectra (in $D_2O$) of **M-1**.

a)

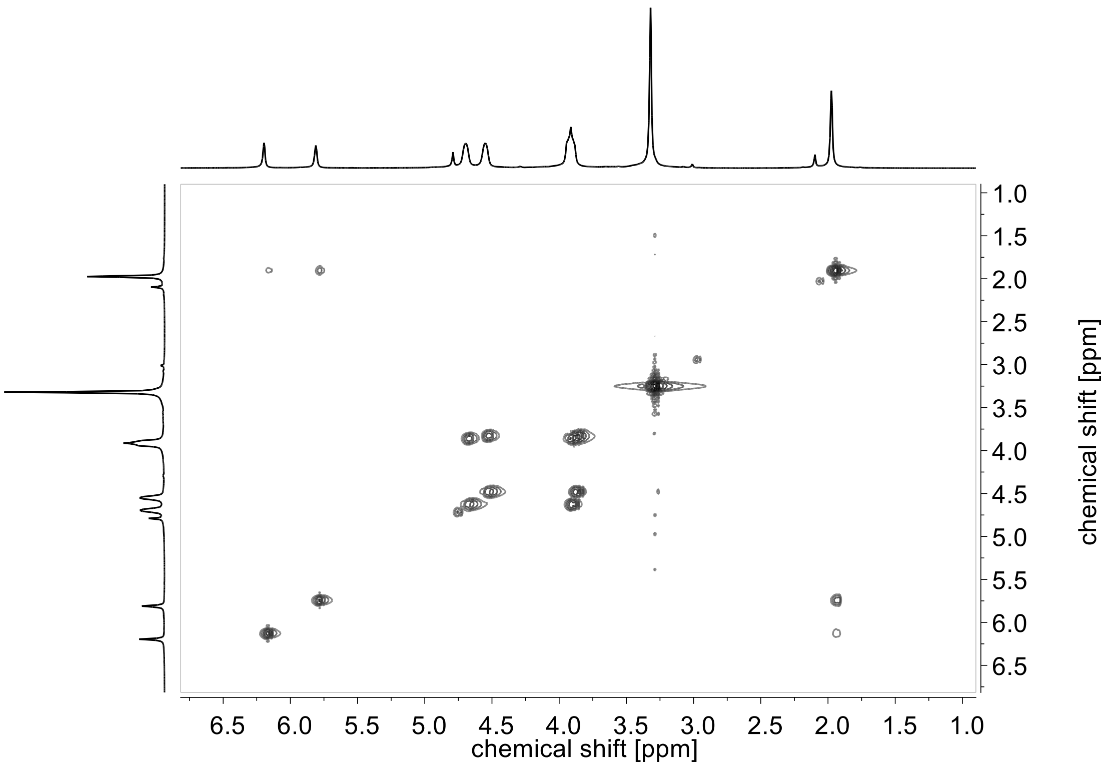


b)

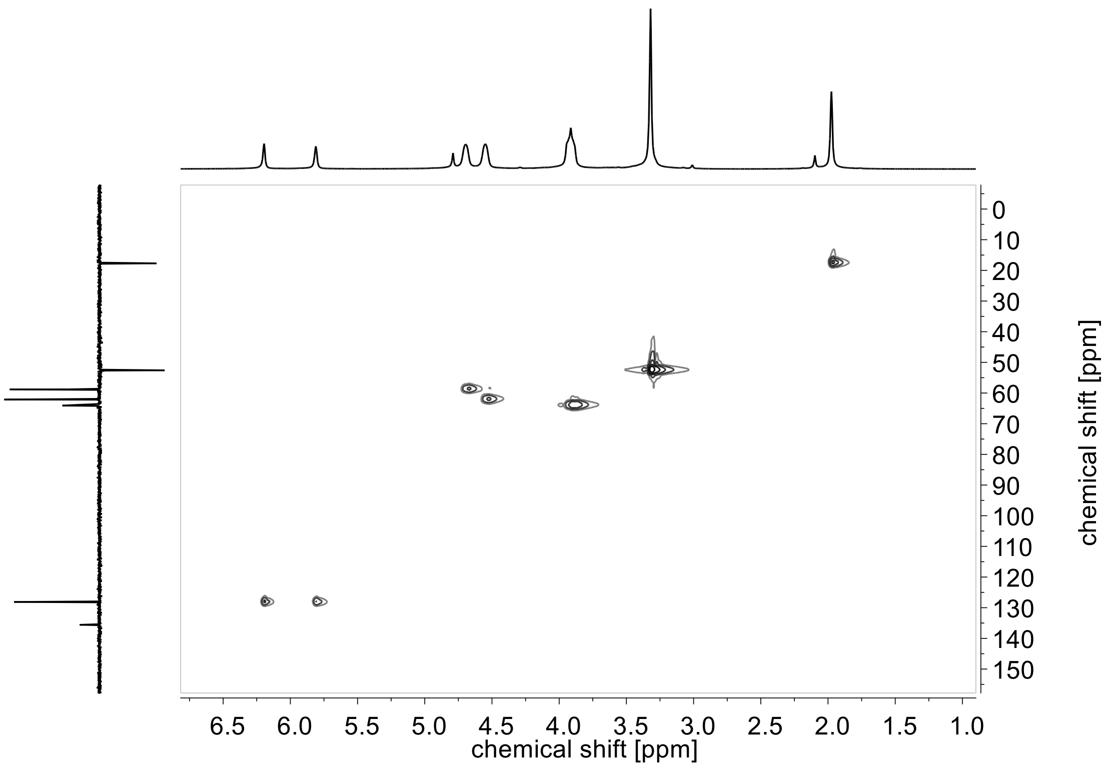


Figure S 2 a) $^1$H-$^1$H-COSY and b) $^1$H-$^{13}$C-HMQC NMR spectra (in $D_2O$) of **M-1**.

*3-(N-(2-(methacryloyloxy)ethyl)-N,N-dimethylammonio)propyl sulfate* ***(M-2)***

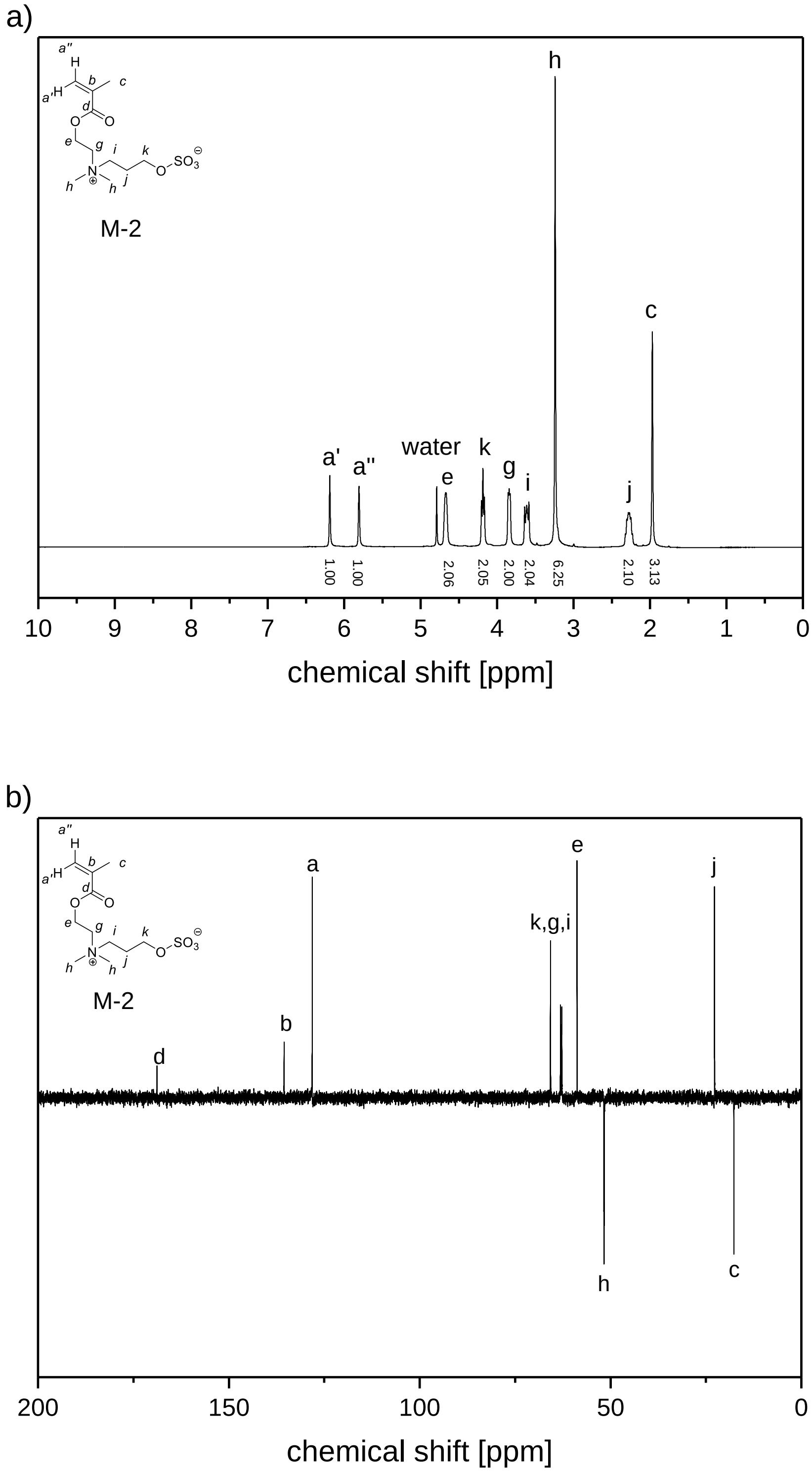


Figure S 3 a) $^{1}H$ NMR (in $D_2O$) and b) $^{13}C$ (APT) NMR spectra (in $D_2O$) of **M-2**.

a)

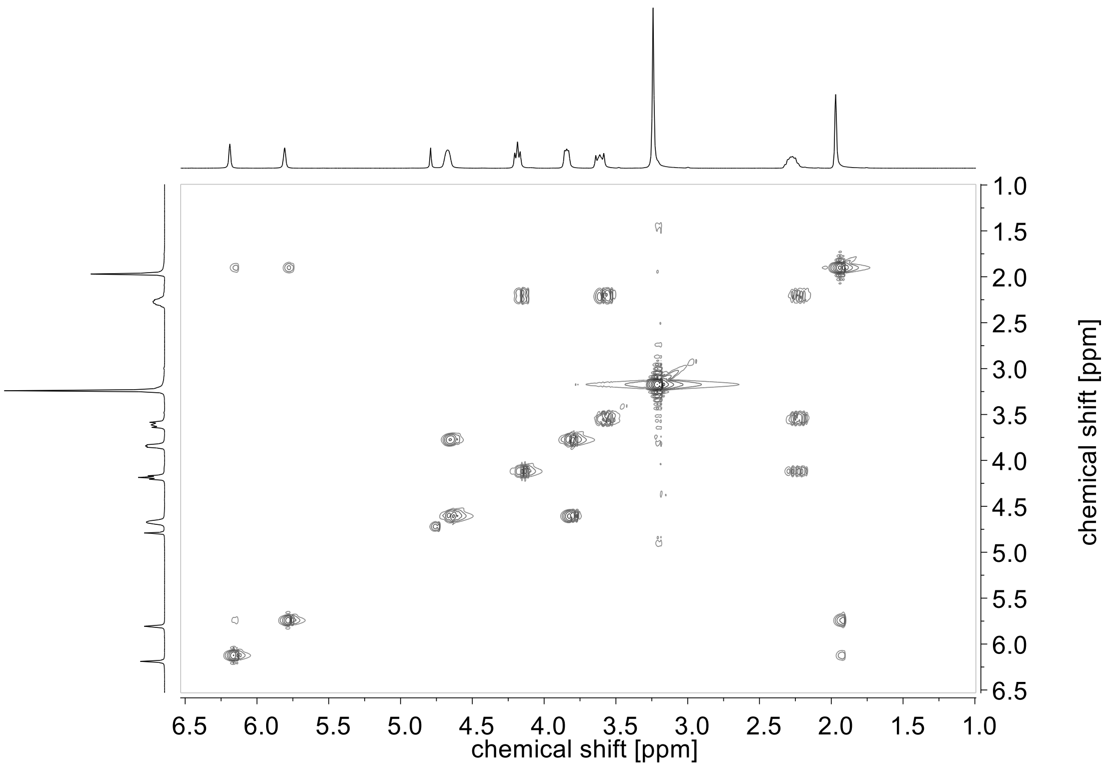


b)

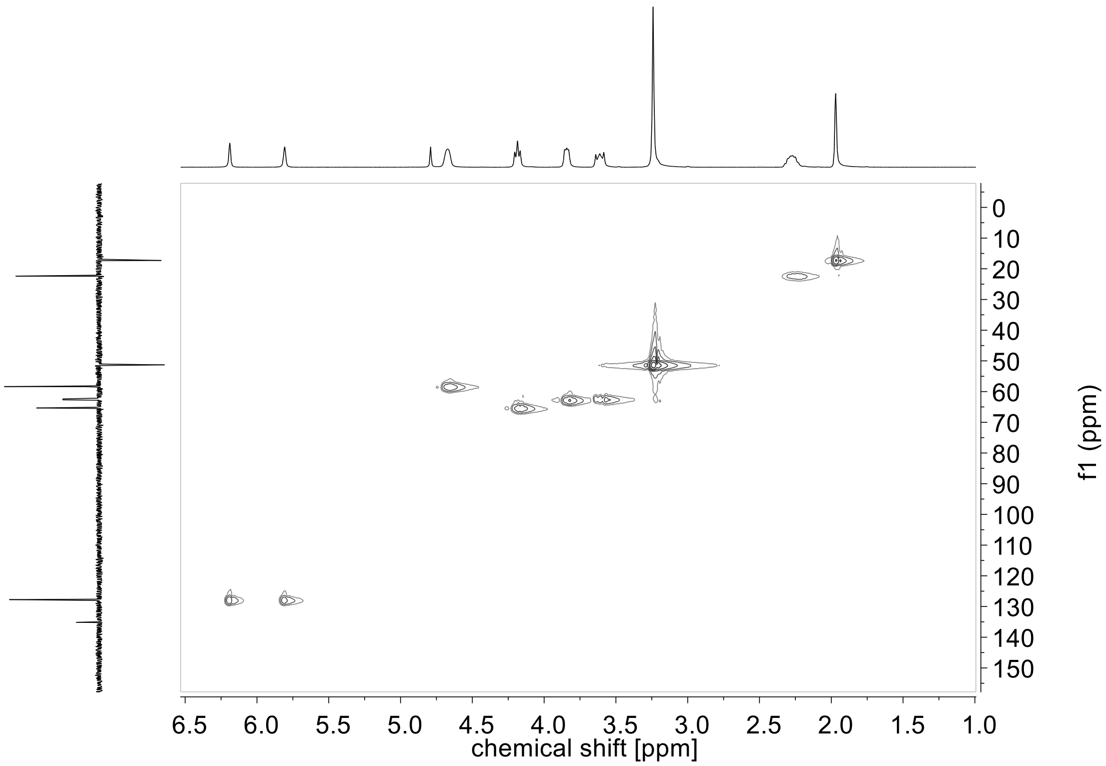


Figure S 4 a) $^{1}H$-$^{1}H$-COSY and b) $^{1}H$-$^{13}C$-HMQC NMR spectra (in $D_2O$) of **M-2**.

*2-(N-(3-methacrylamidopropyl)-N,N-dimethylammonio)ethyl sulfate (**M-3**)*

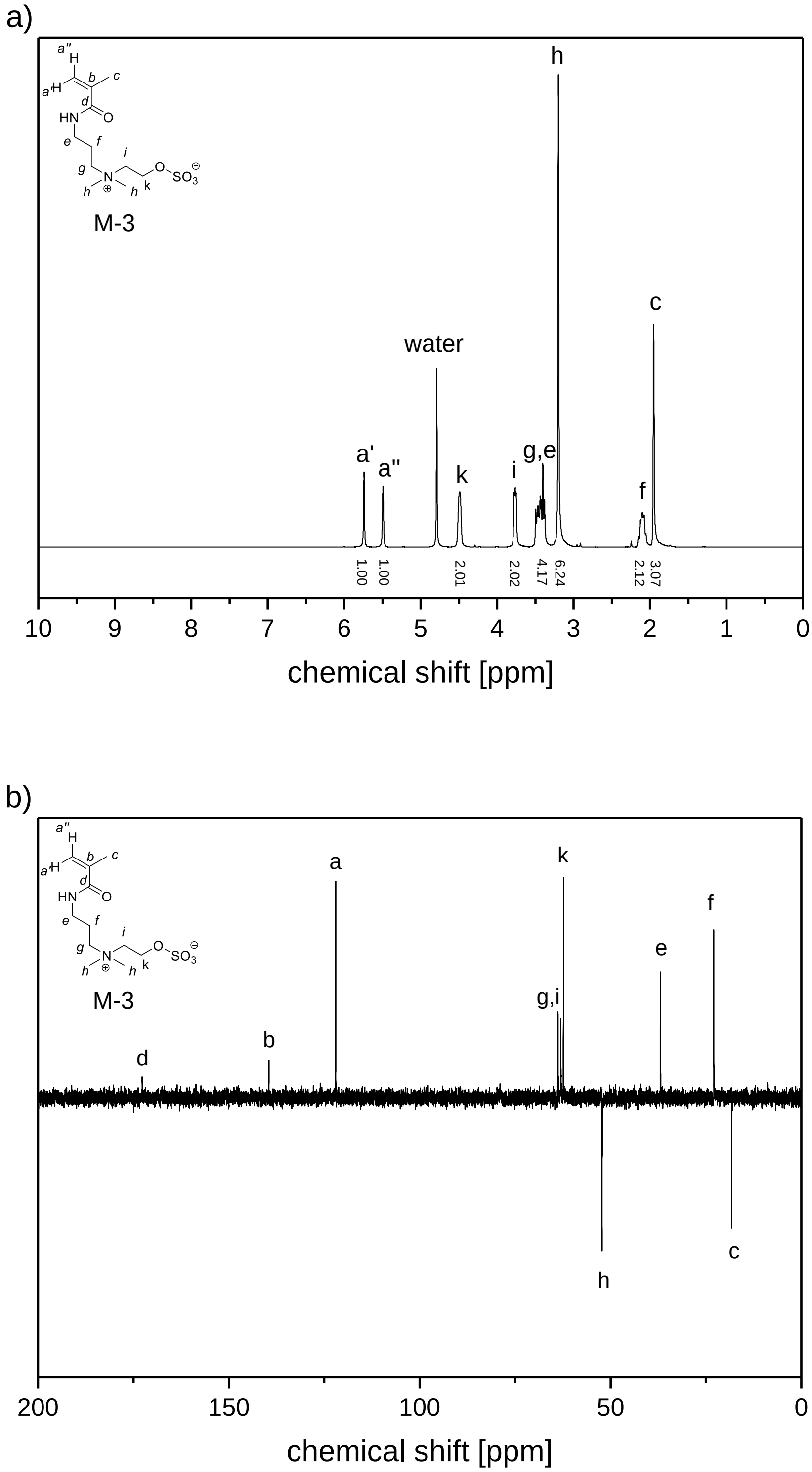


Figure S 5  a) $^{1}$H NMR (in $D_2O$) and b) $^{13}$C (APT) NMR spectra (in $D_2O$) of **M-3**.

a)

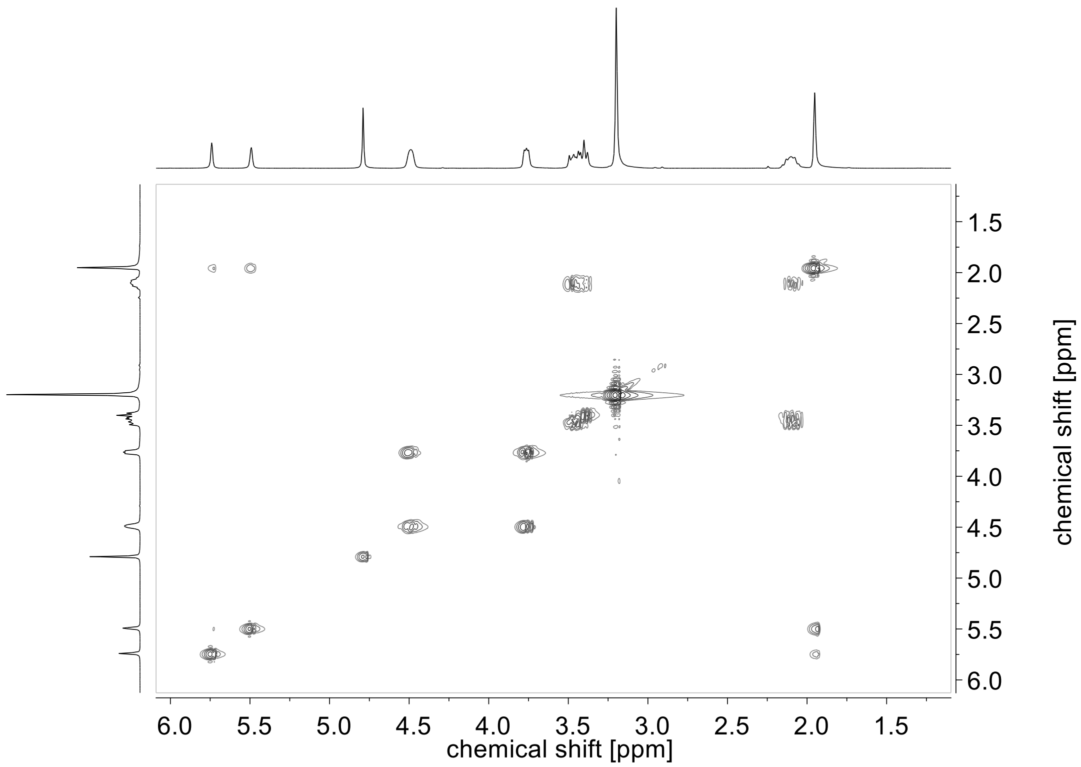


b)

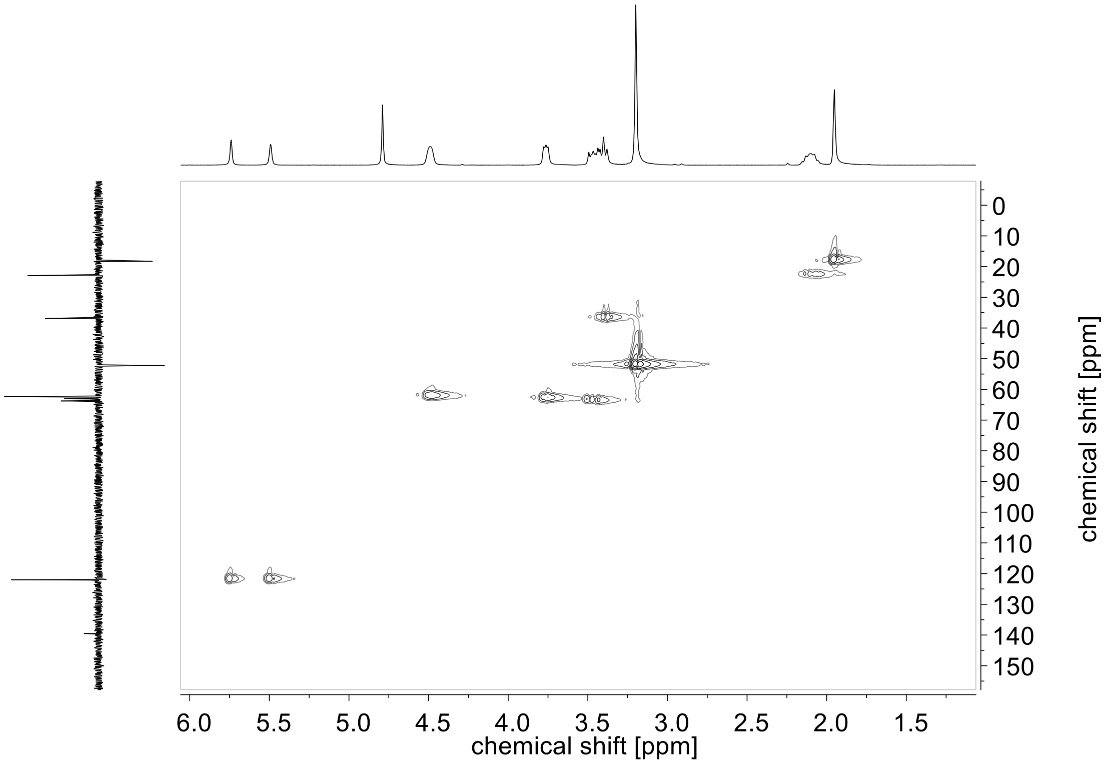


Figure S 6 a) $^{1}H$-$^{1}H$-COSY and b) $^{1}H$-$^{13}C$-HMQC NMR spectra (in $D_2O$) of **M-3**.

*3-(N-(3-methacrylamidopropyl)-N,N-dimethylammonio)propyl sulfate (**M-4**)*

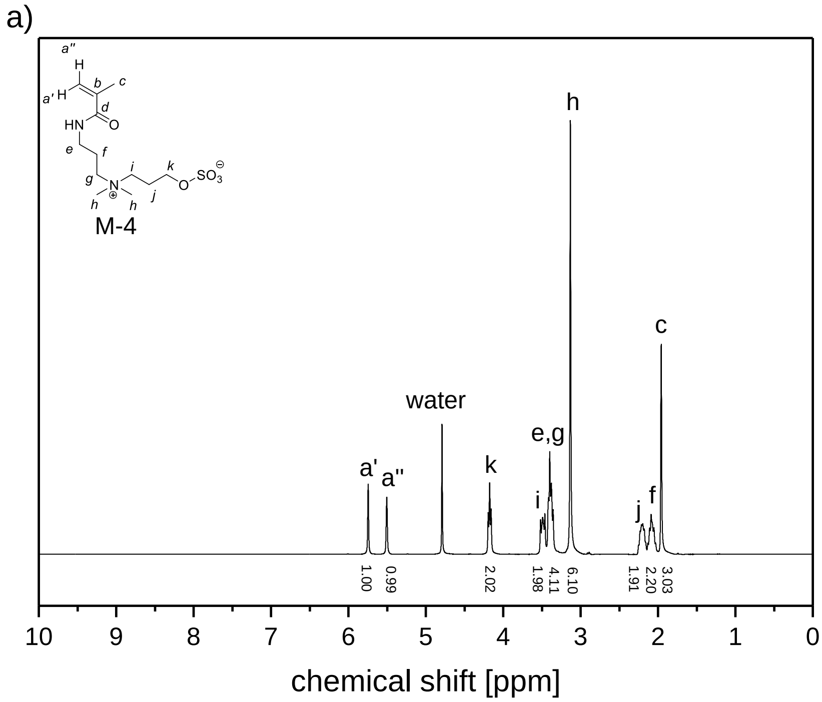


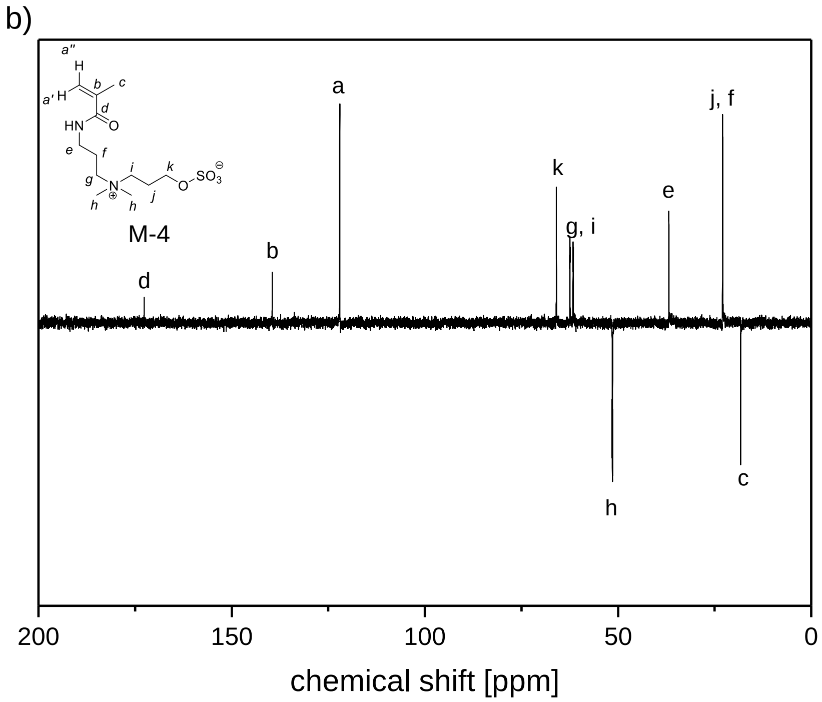


Figure S 7 a) $^{1}$H NMR (in $D_2O$) and b) $^{13}$C (APT) NMR spectra (in $D_2O$) of **M-4**.

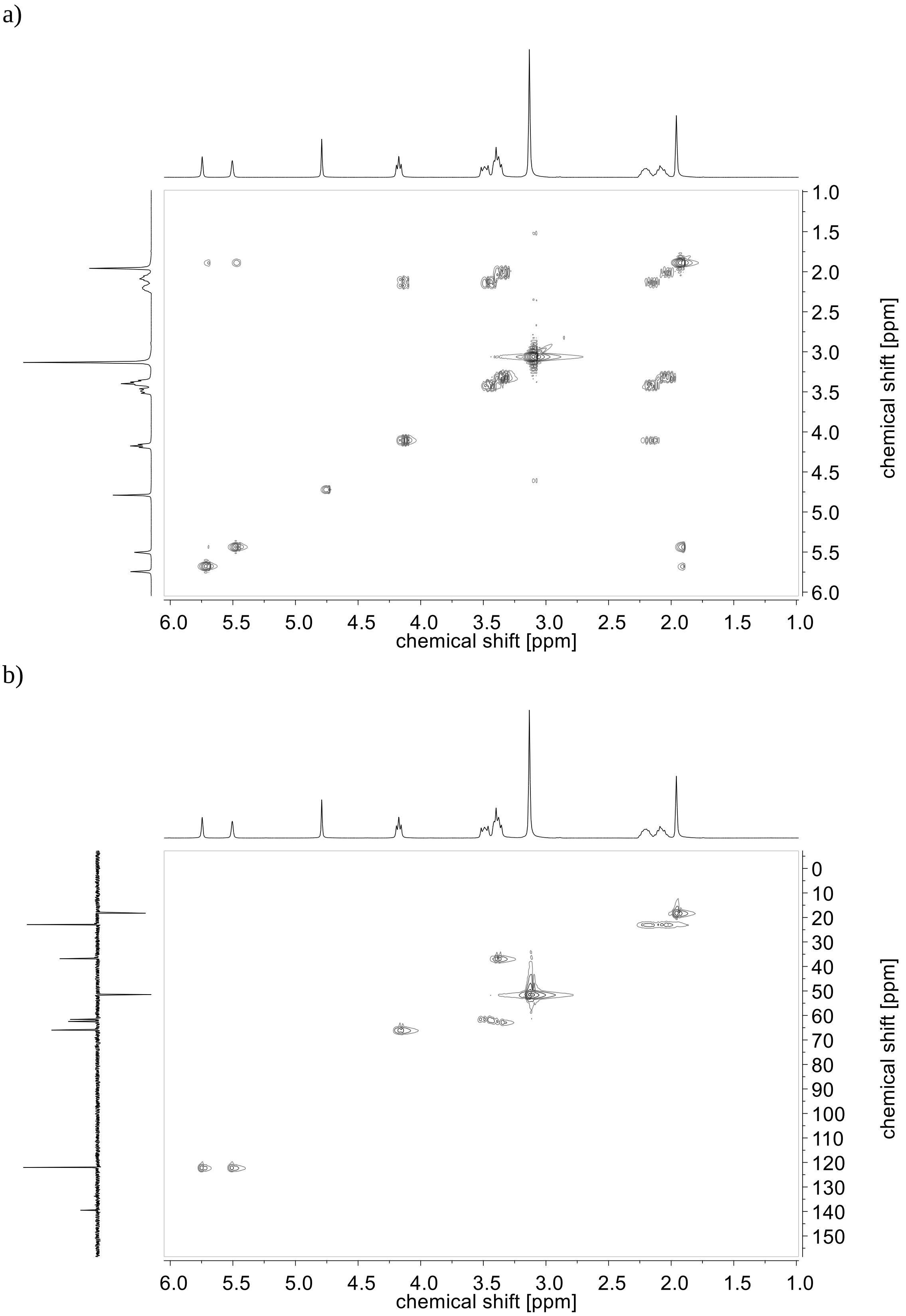


Figure S 8 a) $^{1}H$-$^{1}H$-COSY and b) $^{1}H$-$^{13}C$-HMQC NMR spectra (in $D_2O$) of **M-4**.

*2-(N,N-dimethyl-N-(4-vinylbenzyl)ammonio)ethyl sulfate (**M-5**)*

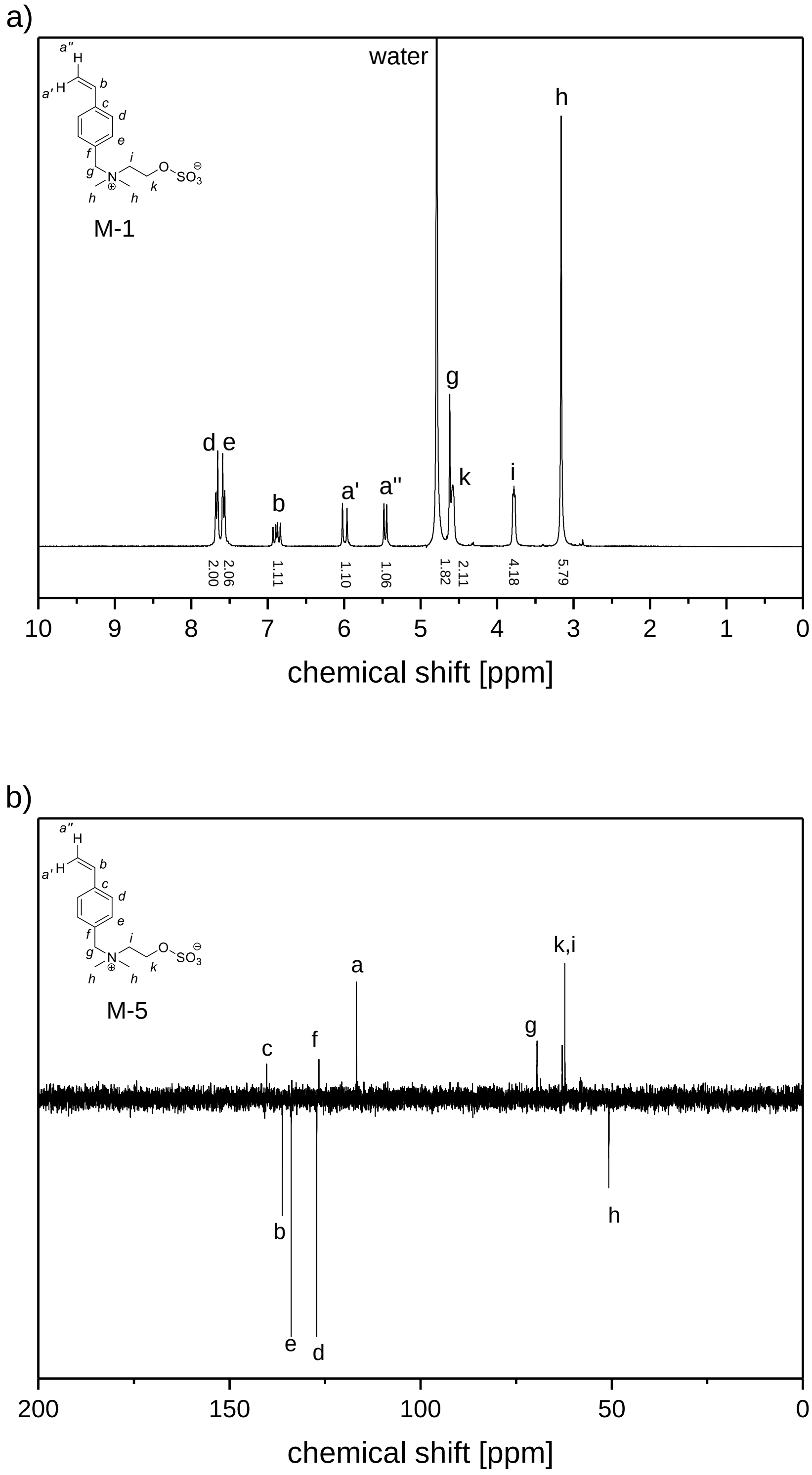


Figure S 9 a) $^{1}H$ NMR (in $D_2O$) and b) $^{13}C$ (APT) NMR spectra (in $D_2O$) of **M-5**

a)

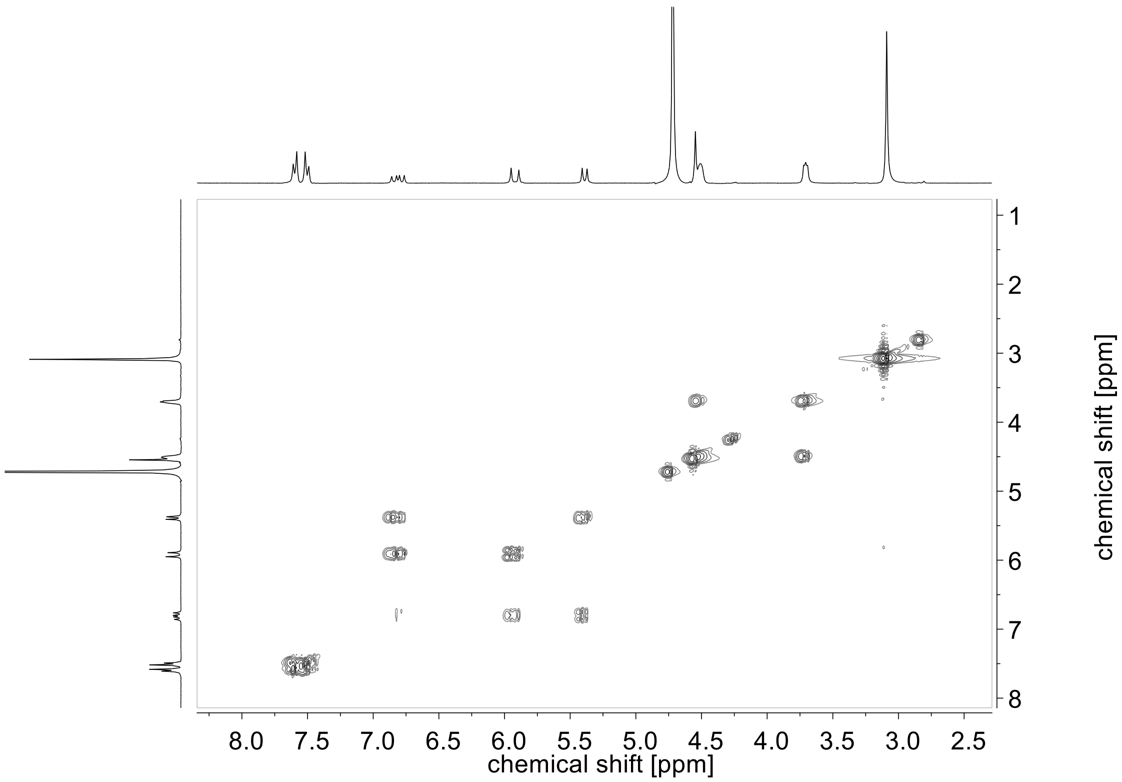


b)

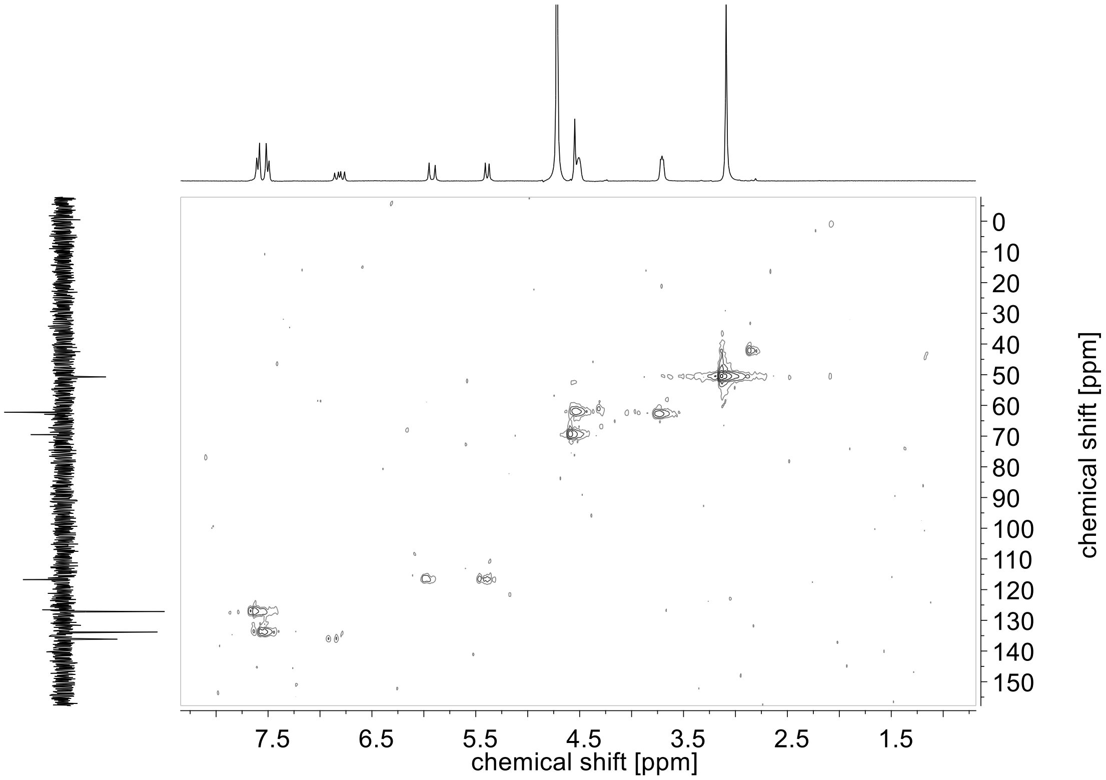


Figure S 10 a) $^{1}H$-$^{1}H$-COSY and b) $^{1}H$-$^{13}C$-HMQC NMR spectra (in $D_2O$) of **M-5**.

*3-N,N-(dimethyl-N-(4-vinylbenzyl)ammonio)propyl sulfate (**M-6**)*

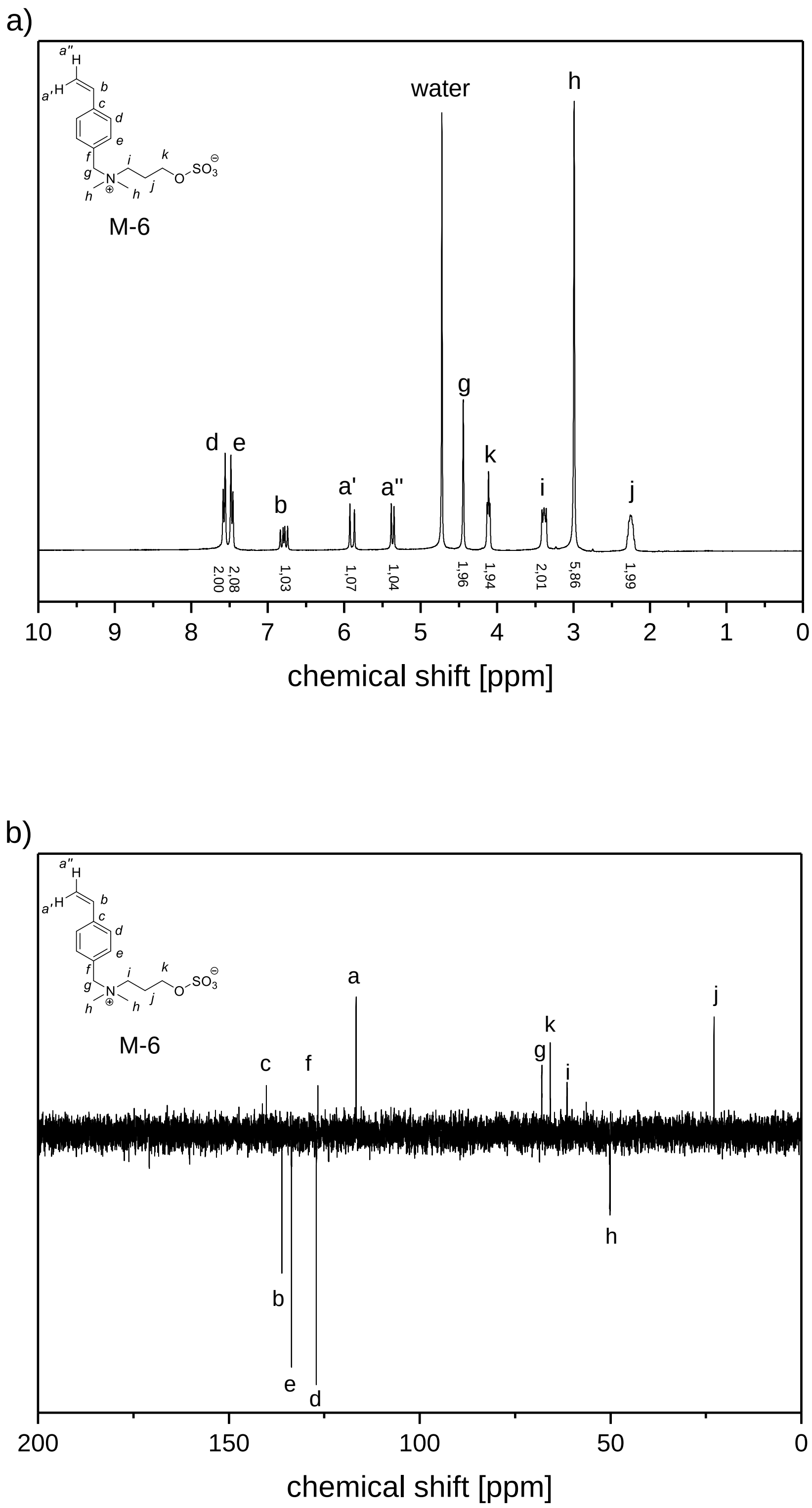


Figure S 11 a) $^{1}$H NMR (in $D_2O$) and b) $^{13}$C (APT) NMR spectra (in $D_2O$) of **M-6**

a)

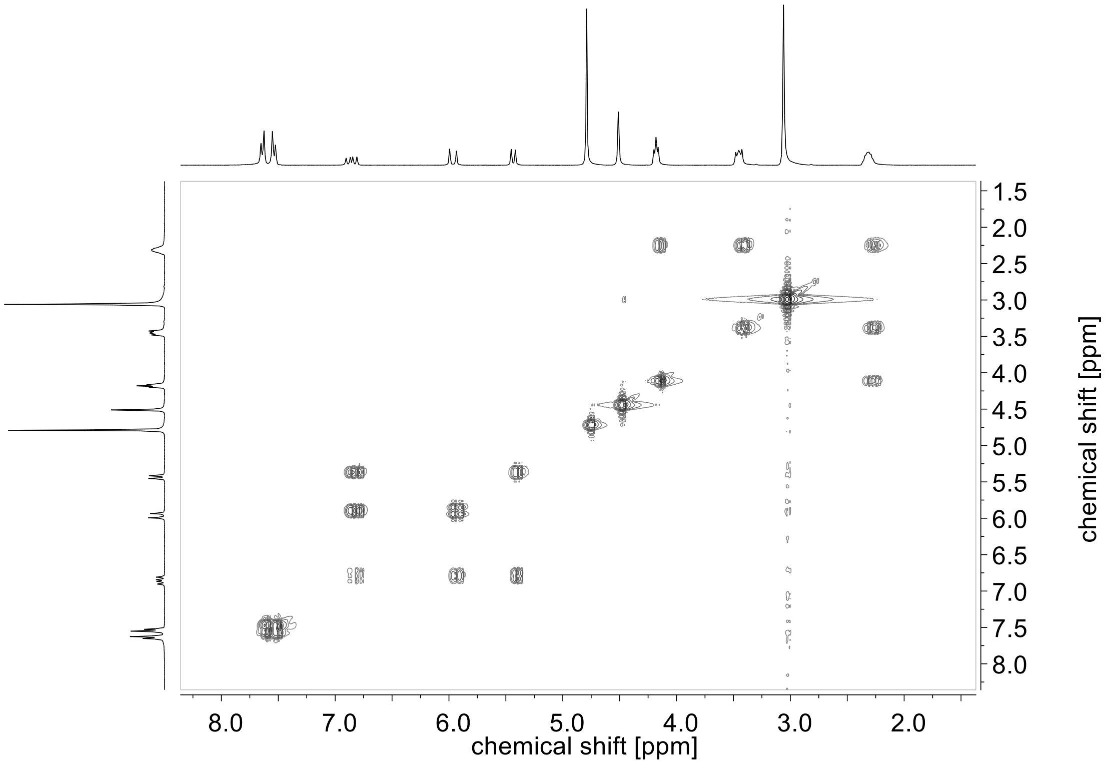


b)

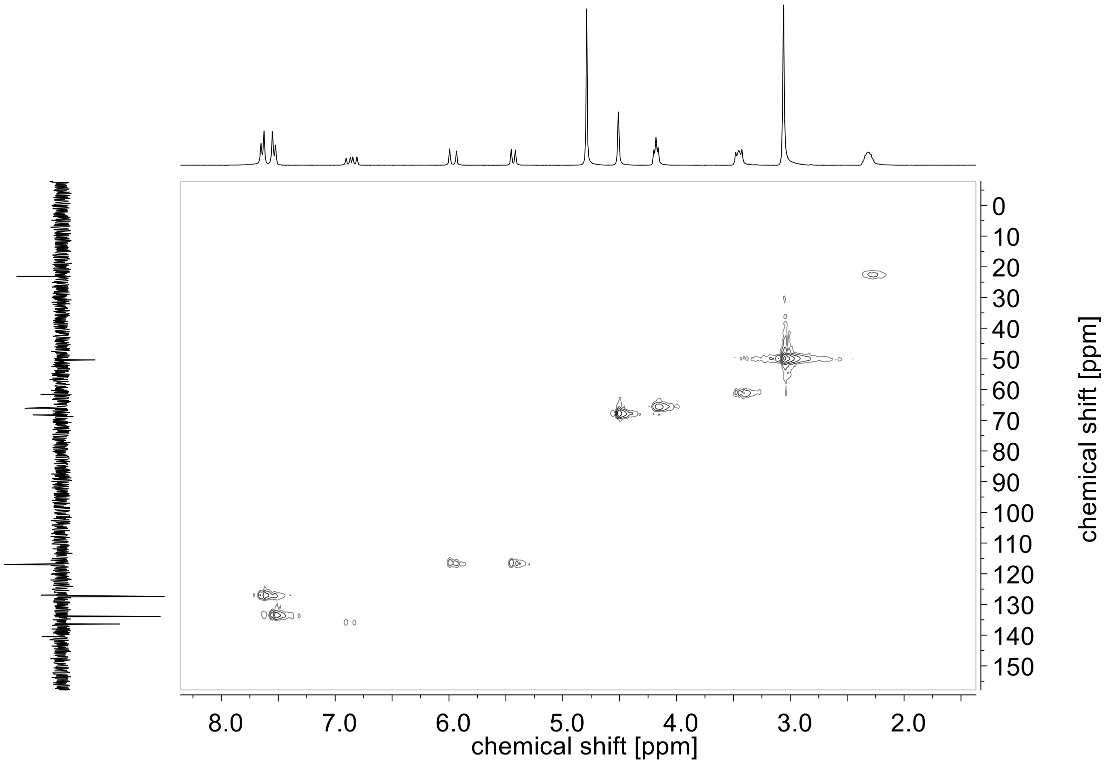


Figure S 12 a) $^{1}H$-$^{1}H$-COSY and b) $^{1}H$-$^{13}C$-HMQC NMR spectra (in $D_2O$) of **M-6**.

## 2. Detailed 1H- and 13C-NMR spectroscopic characterization of the polymers

*Polymer* ***P-OEGMA***

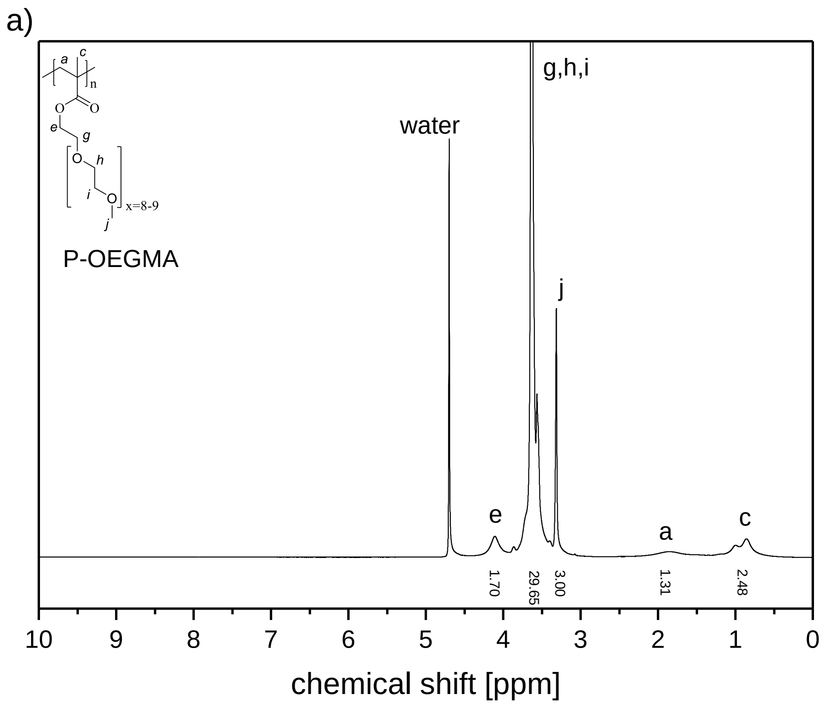


Figure S 13 a) $^{1}$H NMR (in $D_2O$) of **P-OEGMA**.

*Polymer P-SPE*

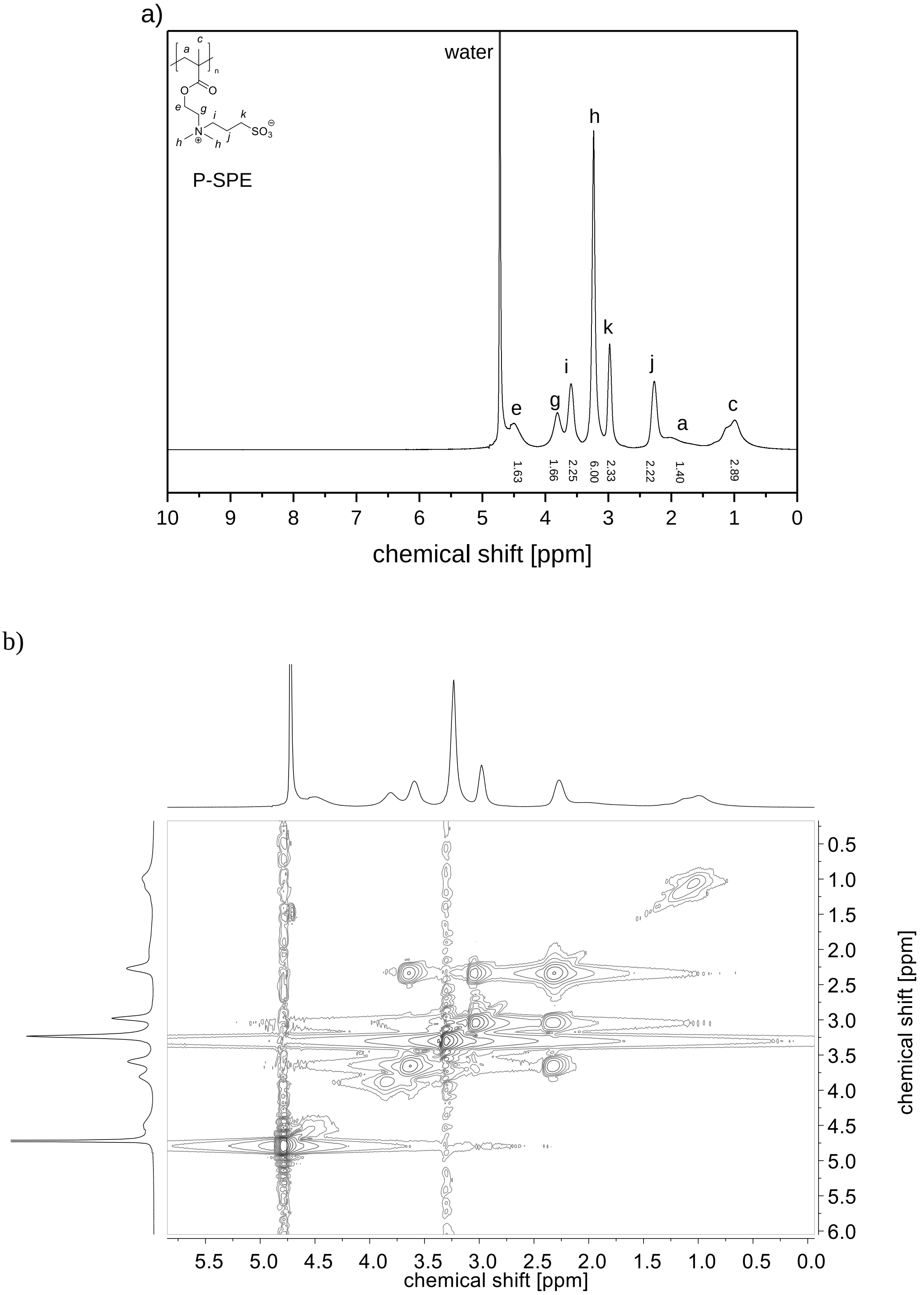


Figure S 14  a) $^{1}H$ NMR (in dilute aqueous NaCl (9.0 $g \cdot L^{-1}$) in $D_2O$) and b) $^{1}H$-$^{1}H$-COSY (in dilute aqueous NaCl (9.0 $g \cdot L^{-1}$) in $D_2O$) of **P-SPE**.

*Polymer **P-SPP***

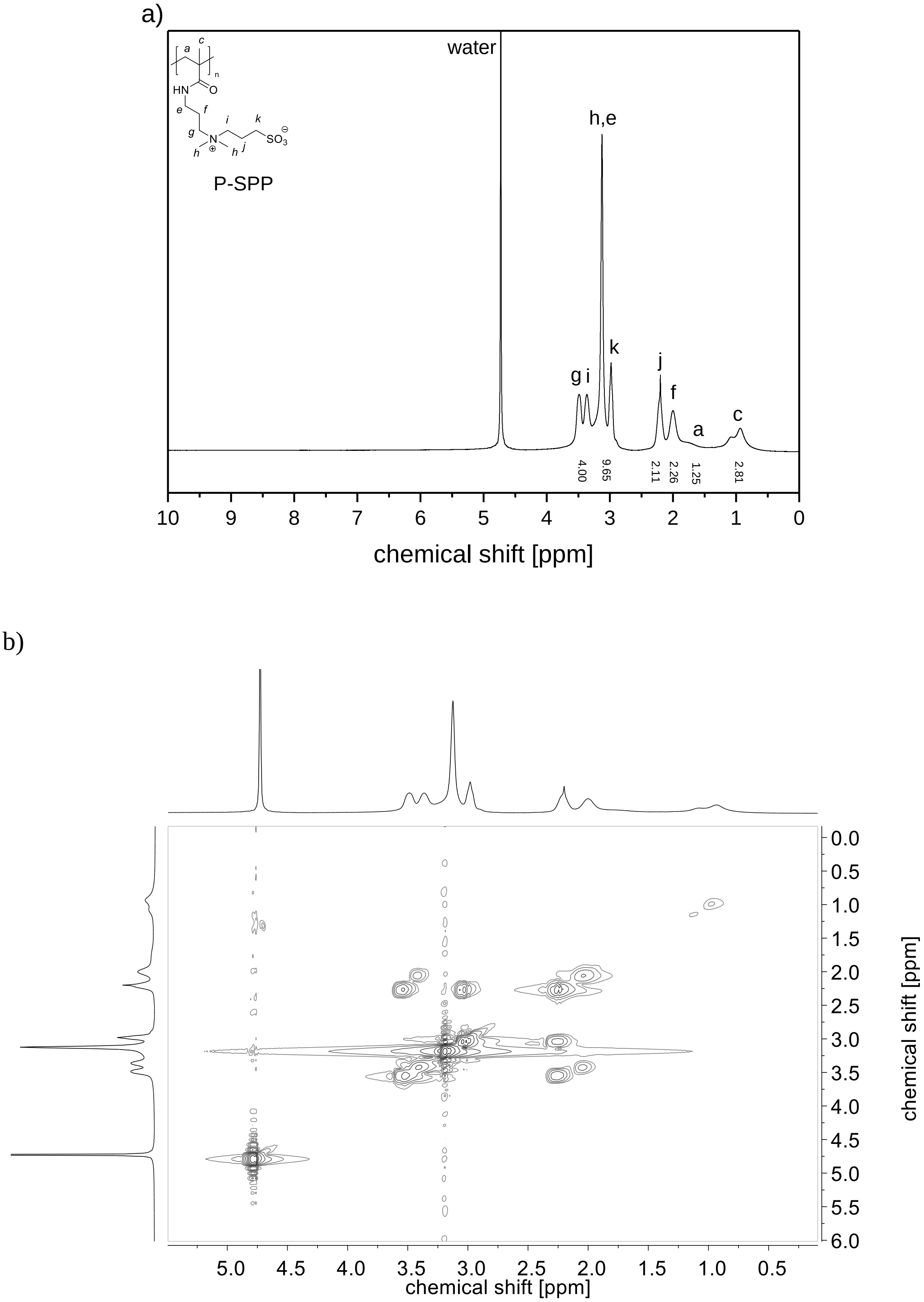


Figure S 15 a) $^{1}H$ NMR (in dilute aqueous NaCl (9.0 g·L$^{-1}$) in $D_2O$) and b) $^{1}H$-$^{1}H$-COSY (in dilute aqueous NaCl (9.0 g·L$^{-1}$) in $D_2O$) of **P-SPP**.

*Polymer* ***P-1***

a)

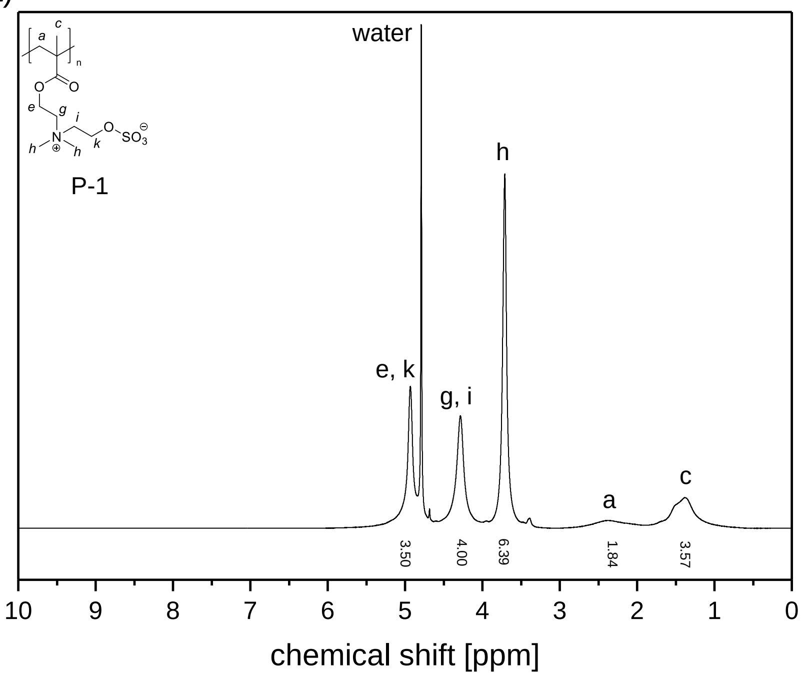


b)

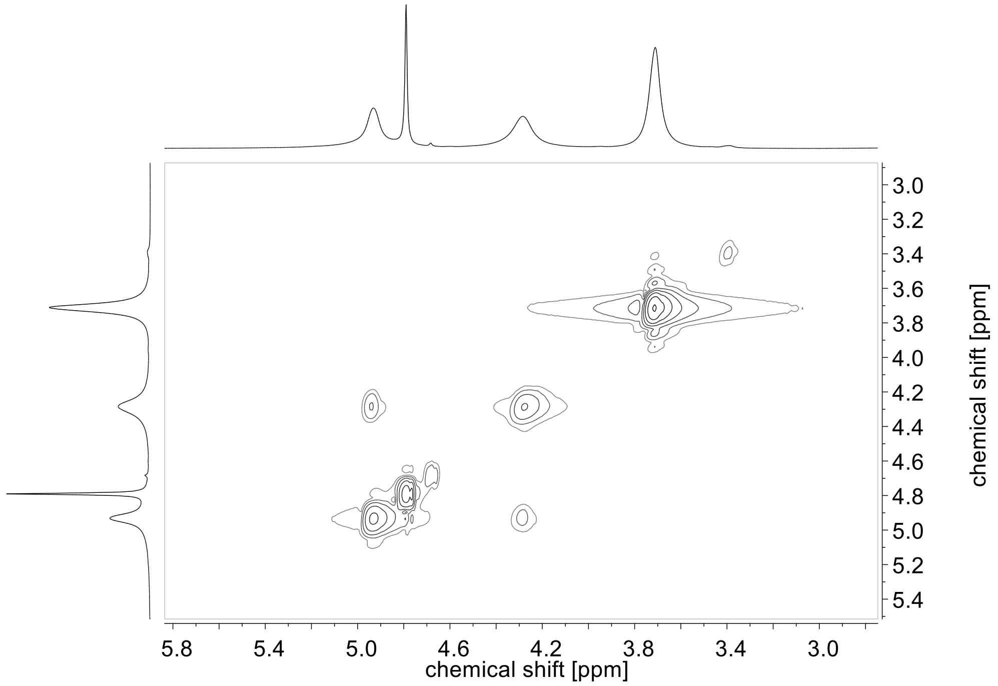


Figure S 16 a) $^{1}H$ NMR (in saturated NaCl solution in $D_2O$) and b) $^{1}H$-$^{1}H$-COSY (in saturated NaCl solution in $D_2O$) of **P-1**.

*Polymer* ***P-2***

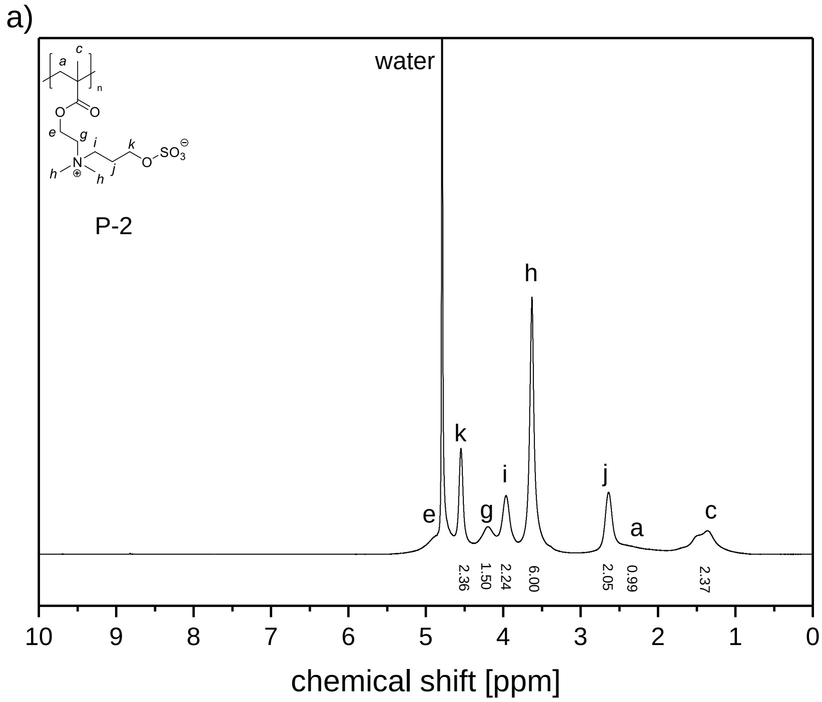


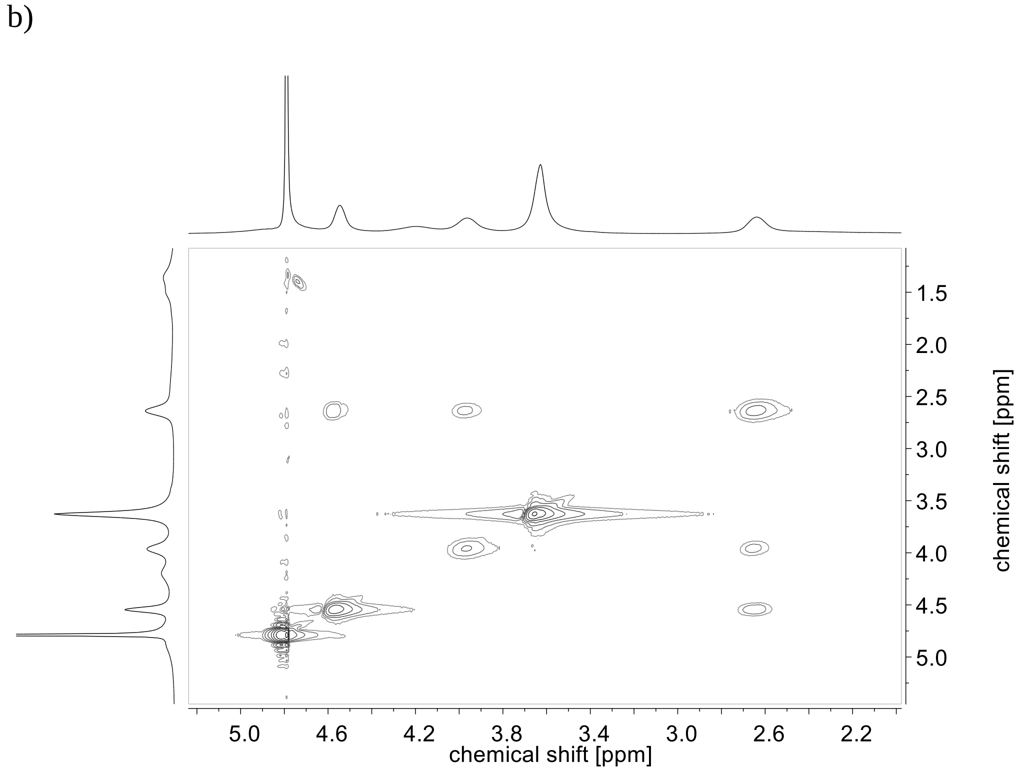


Figure S 17 a) $^{1}H$ NMR (in saturated NaCl solution in $D_2O$) and b) $^{1}H$-$^{1}H$-COSY (in saturated NaCl solution in $D_2O$ of **P-2**.

*Polymer **P-3***

a)

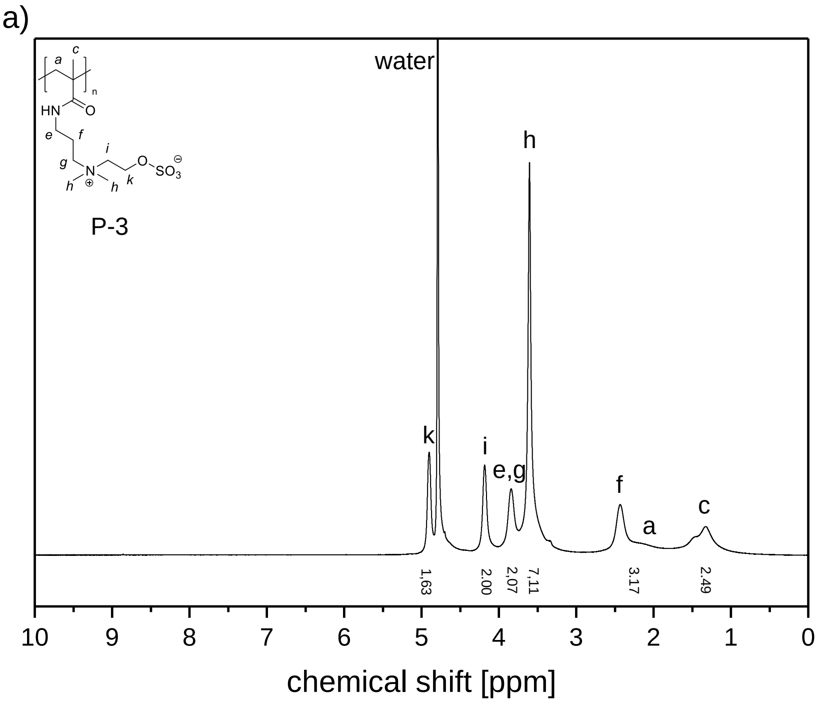


b)

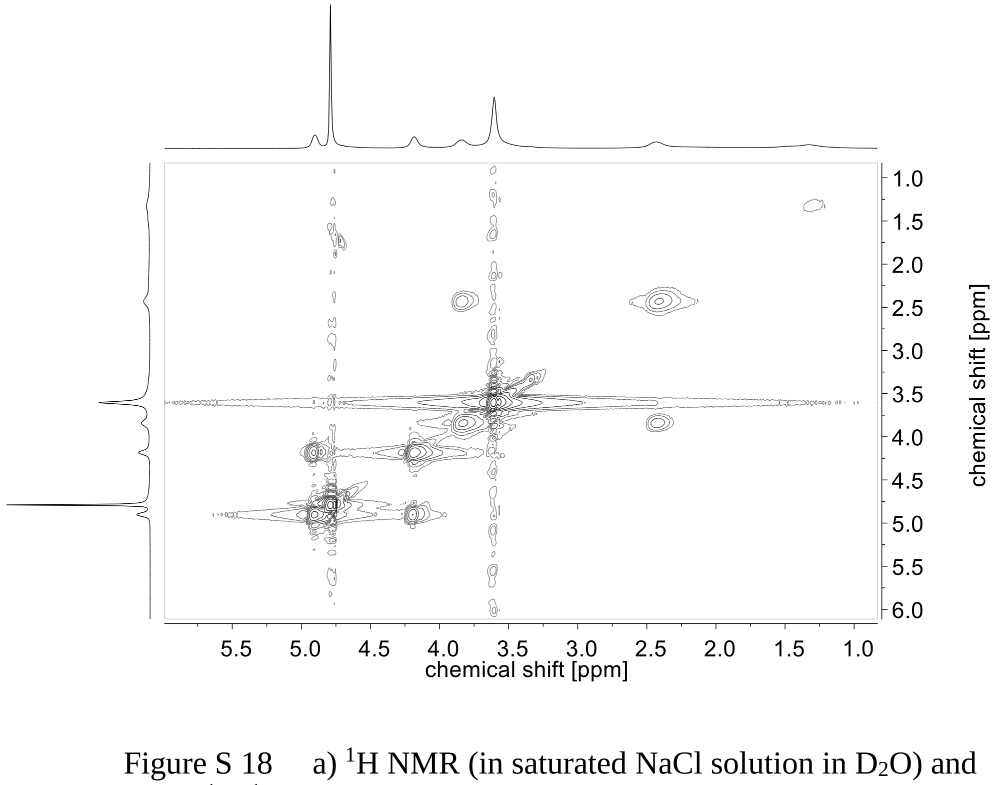


Figure S 18 a) $^{1}H$ NMR (in saturated NaCl solution in $D_2O$) and b) $^{1}H$-$^{1}H$-COSY (in saturated NaCl solution in $D_2O$ of **P-3**.

*Polymer **P-4***

a)

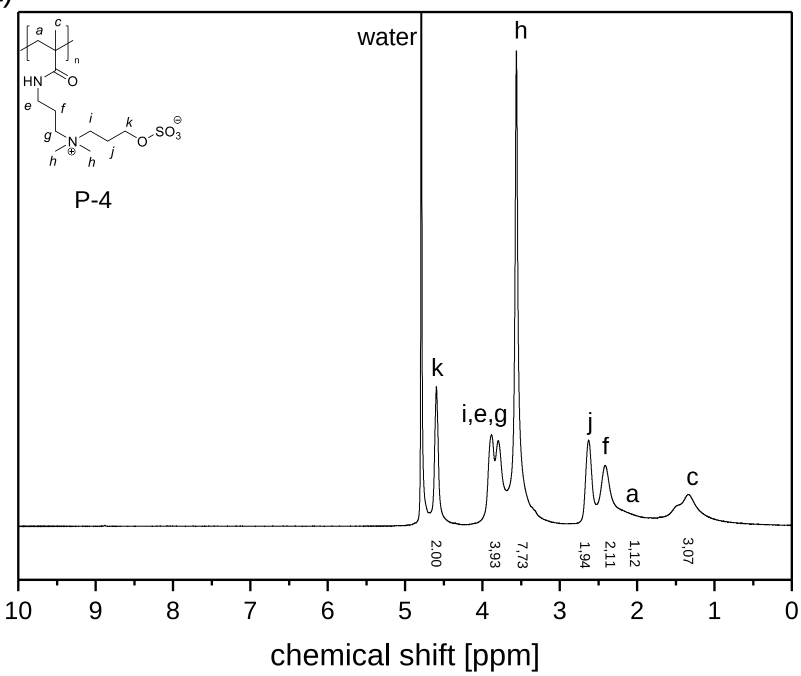


b)

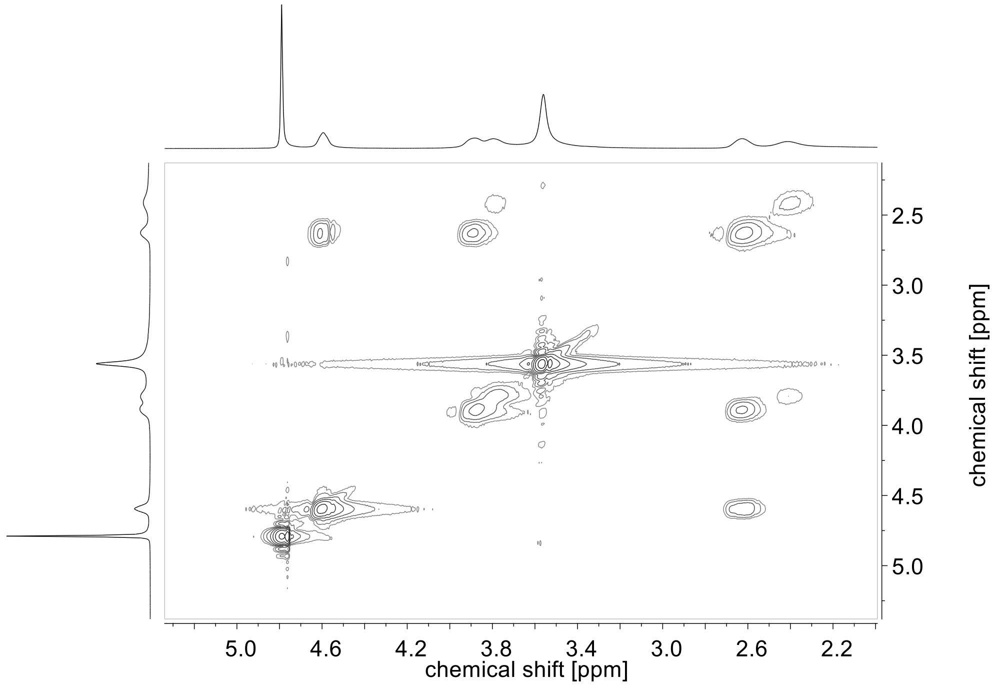


Figure S 19 a) $^{1}H$ NMR (in saturated NaCl solution in $D_2O$) and b) $^{1}H$-$^{1}H$-COSY (in saturated NaCl solution in $D_2O$ of **P-4**.

*Polymer **P-6***

a)

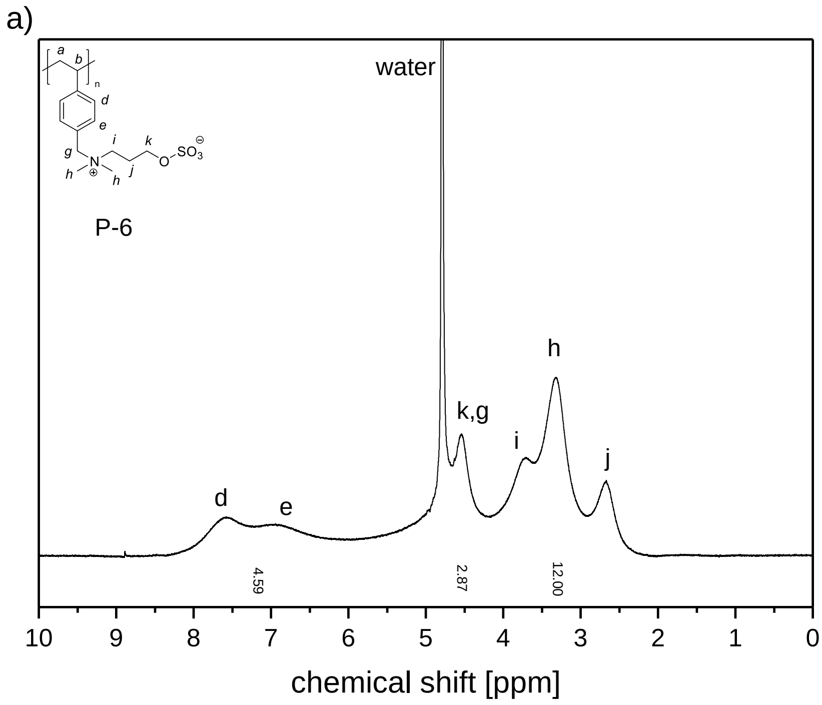


b)

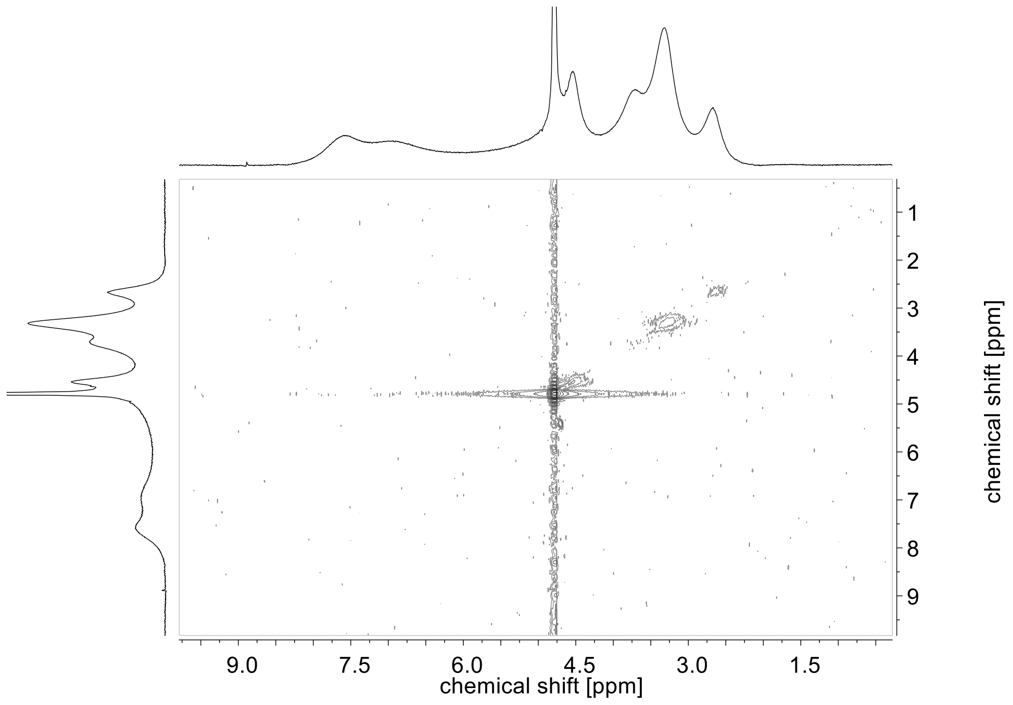


Figure S 20 a) $^{1}H$ NMR (in saturated NaCl solution in $D_2O$) and b) $^{1}H$-$^{1}H$-COSY (in saturated NaCl solution in $D_2O$ of **P-6**.

## 3. Preparation of buffer solution

*1. Monomer/phosphate buffered saline (PBS) pH=7.4*

48 mg of a PBS tablet (provider Sigma Aldrich) were dissolved in 5.0 mL of $D_2O$, resulting in a 0.01 M phosphate buffer, 0.0027 M KCl and 0.137 M NaCl solution with a pH value of 7.4 at 25 °C. 0.6 mL of the prepared buffer solution was added to 0.06 mmol monomer shortly before the first NMR measurement. 3-(Trimethylsilyl)propionic-2,2,3,3-$d_4$ acid sodium salt was added as inner standard. Monomers **M-5** and **M-6** dissolved only partially in the buffer solution.

*2. Monomer/deuterium chloride pH=0*

0.8 mL of deuterium chloride (38 wt% in $D_2O$) and 12.5 of 3-(trimethylsilyl)propionic-2,2,3,3-$d_4$ acid sodium salt (12,5 mM) were dissolved in 4.2 mL of $D_2O$, resulting in a deuterium chloride solution with a pH-value of 0. 0.3 mL of the so prepared solution was added to a 0.3 mL/0.06 mmol of monomer solution shortly before the first NMR measurement. Monomers **M-5** and **M-6** dissolved only partially in the buffer solution.

*3. Monomer/carbonate buffer pH=10*

302.4 mg of $NaHCO_3$, 148.4 mg of $Na_2CO_3$ and 12.5 mg of 3-(trimethylsilyl)propionic-2,2,3,3-$d_4$ acid sodium salt (12,5 mM) were dissolved in 5.0 mL of $D_2O$, resulting in a 1 molar carbonate buffer solution with a pH-value of 10. 0.6 mL of the prepared buffer solution was added to 0.06 mmol of monomer shortly before the first NMR measurement. Monomers **M-5** and **M-6** dissolved only partially in the buffer solution.

*4. Monomer/sodium hydroxide pH=14*

400 mg of NaOH and 12.5 of 3-(trimethylsilyl)propionic-2,2,3,3-$d_4$ acid sodium salt (12,5 mM) were dissolved in 5.0 mL of $D_2O$, resulting in a sodium hydroxide solution with a pH-value of 14. 0.3 mL of the prepared solution was added to 0.06 mmol monomer in 0.3 mL $D_2O$ shortly before the first NMR measurement. Monomers **M-5** and **M-6** dissolved only partially in the buffer solution.

*5. Polymer/phosphate buffered saline (PBS) pH=7.4*

48 mg of a PBS pill (provider Sigma Aldrich) and 12.5 mg of 3-(trimethylsilyl)propionic-2,2,3,3-$d_4$ acid sodium salt (12,5 mM) were dissolved in 5.0 mL of $D_2O$, resulting in a 0.01 M phosphate buffer, 0.0027 M KCl and 0.137 M NaCl solution with a pH value of 7.4 at 25 °C. The prepared buffer solution was added to the equivalent weight of 0.06 mmol repeating units of the polymer before the first NMR measurement. In case of **P-1** to **P-6** the prepared phosphate buffer solution was additionally saturated with sodium chloride. Polymer **P-5** did not dissolve in the sodium chloride saturated phosphate buffer solution.

*6. Monomer/deuterium chloride pH=0*

0.8 mL of deuterium chloride (38 wt% in $D_2O$) and 12.5 of 3-(trimethylsilyl)propionic-2,2,3,3-$d_4$ acid sodium salt (12,5 mM) were dissolved in 4.2 mL of $D_2O$, resulting in a deuterium chloride solution with a pH-value of 0. 0.3 mL of the prepared solution was added to a polymer solution in pure $D_2O$ (in case of **P-OEGMA**, **P-SPE** and **P-SPP**), or in a saturated NaCl in $D_2O$ (in case of **P-1** to **P-6**) before the first NMR measurement. For **P-1** to **P-6**, the DCl solution was additionally saturated with sodium chloride, before

added to the polymer solution. **P-5** did not dissolve in the sodium chloride saturated deuterium chloride solution.

*7. Polymer/carbonate buffer pH=10*

302.4 mg of $NaHCO_3$, 148.4 mg of $Na_2CO_3$ and 12.5 mg of 3-(trimethylsilyl)propionic-2,2,3,3-$d_4$ acid sodium salt (12,5 mM) were dissolved in 5 mL of $D_2O$, resulting in a carbonate buffer solution with a pH-value of 10. 0.3 mL of the prepared buffer solution was added in a polymer solution in pure $D_2O$ (in case of P-OEGMA, P-SPE and P-SPP), or in saturated NaCl in $D_2O$ (in case of **P-1** to **P-6**) before the first NMR measurement. **P-5** did not dissolve in the sodium chloride saturated carbonate buffer solution.

*8. Polymer/sodium hydroxide pH=14*

400 mg of NaOH and 12.5 of 3-(trimethylsilyl)propionic-2,2,3,3-$d_4$ acid sodium salt (12,5 mM) were dissolved in 5.0 mL of $D_2O$, resulting in a sodium hydroxide solution with a pH-value of 14. 0.3 mL of the prepared solution was added to a polymer solution in pure $D_2O$ (in case of **P-OEGMA**, **P-SPE** and **P-SPP**), or in a saturated NaCl in $D_2O$ (in case of **P-1** to **P-6**) before the first NMR measurement. For **P-1** and **P-6**, the NaOH solution was additionally saturated with sodium chloride, before added to the polymer solution. **P-5** did not dissolve in the sodium chloride saturated sodium hydroxide solution.

**4. Evolution of the monomer and polymer 1H-NMR spectra upon storage in aqueous media at 22 °C at various pH values.**

4.1. Monomer hydrolysis in phosphate buffered saline (pH = 7.4)

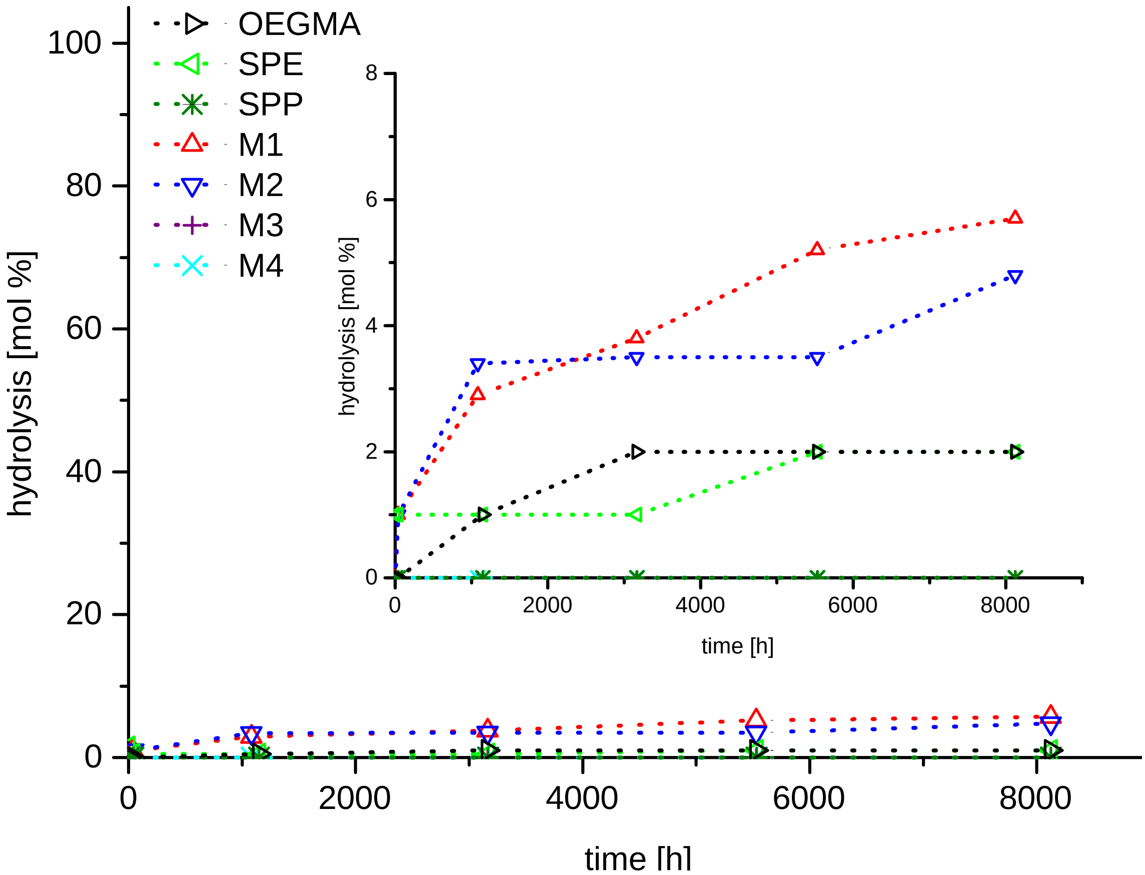


Figure S 21 Evolution of ester and amid hydrolysis of monomers in phosphate buffered saline (PBS) in $D_2O$ (undiluted PBS contains yields in 0.137 mol*$L^{-1}$ of NaCl, 0.0027 mol*$L^{-1}$ of KCl and 0.01 mol*$L^{-1}$phosphate buffer, pH 7.4 at 25 °C): (□) = **OEGMA**, (□) = **SPE**, (*) = **SPP**, (□) = **M-1**, (□) = **M-2**, (+) = **M-3**, (X) = **M-4**.

Calculation of hydrolysis in mol %:

$$Hydrolyse_{\text{M-1}}\ [\text{mol }\%] = \left(\frac{I_{a_2'}*100}{I_{a'}+I_{a_2'}} + \frac{I_{a_2''}*100}{I_{a''}+I_{a_2''}}\right)/2$$

$$Hydrolyse_{M-2}\ [\text{mol }\%] = \left(\frac{I_{a_2'}*100}{I_{a'}+I_{a_2'}} + \frac{I_{a_2''}*100}{I_{a''}+I_{a_2''}}\right)/2$$

$$Hydrolyse_{M-3}\ [\text{mol }\%] = \left(\frac{I_{a_2'}*100}{I_{a'}+I_{a_2'}} + \frac{I_{a_2''}*100}{I_{a''}+I_{a_2''}}\right)/2$$

$$Hydrolyse_{M-4}\ [\text{mol }\%] = \left(\frac{I_{a_2'}*100}{I_{a'}+I_{a_2'}} + \frac{I_{a_2''}*100}{I_{a''}+I_{a_2''}}\right)/2$$

$$Hydrolyse_{OEGMA}\text{ [mol \%]} = (\frac{I_{a_2''}*100}{I_{a'}+I_{a_2''}} + \frac{I_{a_2''}*100}{I_{a''}+I_{a_2''}})/2$$

$$Hydrolyse_{SPE}\text{ [mol \%]} = (\frac{I_{a_2''}*100}{I_{a'}+I_{a_2''}} + \frac{I_{a_2''}*100}{I_{a''}+I_{a_2''}})/2$$

$$Hydrolyse_{SPP}\text{ [mol \%]} = (\frac{I_{a_2'}*100}{I_{a'}+I_{a_2'}} + \frac{I_{a_2''}*100}{I_{a''}+I_{a_2''}})/2$$

The Index 2 in e.g. $I_{e_2}$indicates the hydrolysis product of the ester/amid product, while no index e.g. $I_{a''}$ determines the unchanged molecule without hydrolysis.

$I_{a'}(M\text{-}1,\ range\ in\ ppm)$ = 6.3-6.1
$I_{a_2'}(M\text{-}1,\ range\ in\ ppm)$ = 5.7-5.6
$I_{a''}(M\text{-}1,\ range\ in\ ppm)$ = 5.9-5.7
$I_{a_2''}(M\text{-}1,\ range\ in\ ppm)$ = 5.4-5.3
$I_{a'}(M\text{-}2,\ range\ in\ ppm)$ = 6.3-6.1
$I_{a_2'}(M\text{-}2,\ range\ in\ ppm)$ = 5.7-5.6
$I_{a''}(M\text{-}2,\ range\ in\ ppm)$ = 5.9-5.7
$I_{a_2''}(M\text{-}2,\ range\ in\ ppm)$ = 5.4-5.3
$I_{a'}(M\text{-}3,\ range\ in\ ppm)$ = 5.9-5.6
$I_{a_2'}(M\text{-}3,\ range\ in\ ppm)$ = no signal
$I_{a''}(M\text{-}3,\ range\ in\ ppm)$ = 5.6-5.4
$I_{a_2''}(M\text{-}3,\ range\ in\ ppm)$ = no signal
$I_{a'}(M\text{-}4,\ range\ in\ ppm)$ = 5.9-5.6
$I_{a_2'}(M\text{-}4,\ range\ in\ ppm)$ = no signal
$I_{a''}(M\text{-}4,\ range\ in\ ppm)$ = 5.6-5.4
$I_{a_2''}(M\text{-}4,\ range\ in\ ppm)$ = no signal
$I_{a'}(OEGMA,\ range\ in\ ppm)$ = 6.3-6.0
$I_{a''}(OEGMA,\ range\ in\ ppm)$ = 5.8-5.5
$I_{a_2''}(OEGMA,\ range\ in\ ppm)$ = 5.4-5.3
$I_{a'}(SPE,\ range\ in\ ppm)$ = 6.4-6.1
$I_{a''}(SPE,\ range\ in\ ppm)$ = 5.9-5.7
$I_{a_2''}(SPE,\ range\ in\ ppm)$ = 5.5-5.3
$I_{a'}(SPP,\ range\ in\ ppm)$ = 5.9-5.6
$I_{a_2'}(SPP,\ range\ in\ ppm)$ = no signal
$I_{a''}(SPP,\ range\ in\ ppm)$ = 5.6-5.4
$I_{a_2''}(SPP,\ range\ in\ ppm)$ = no signal

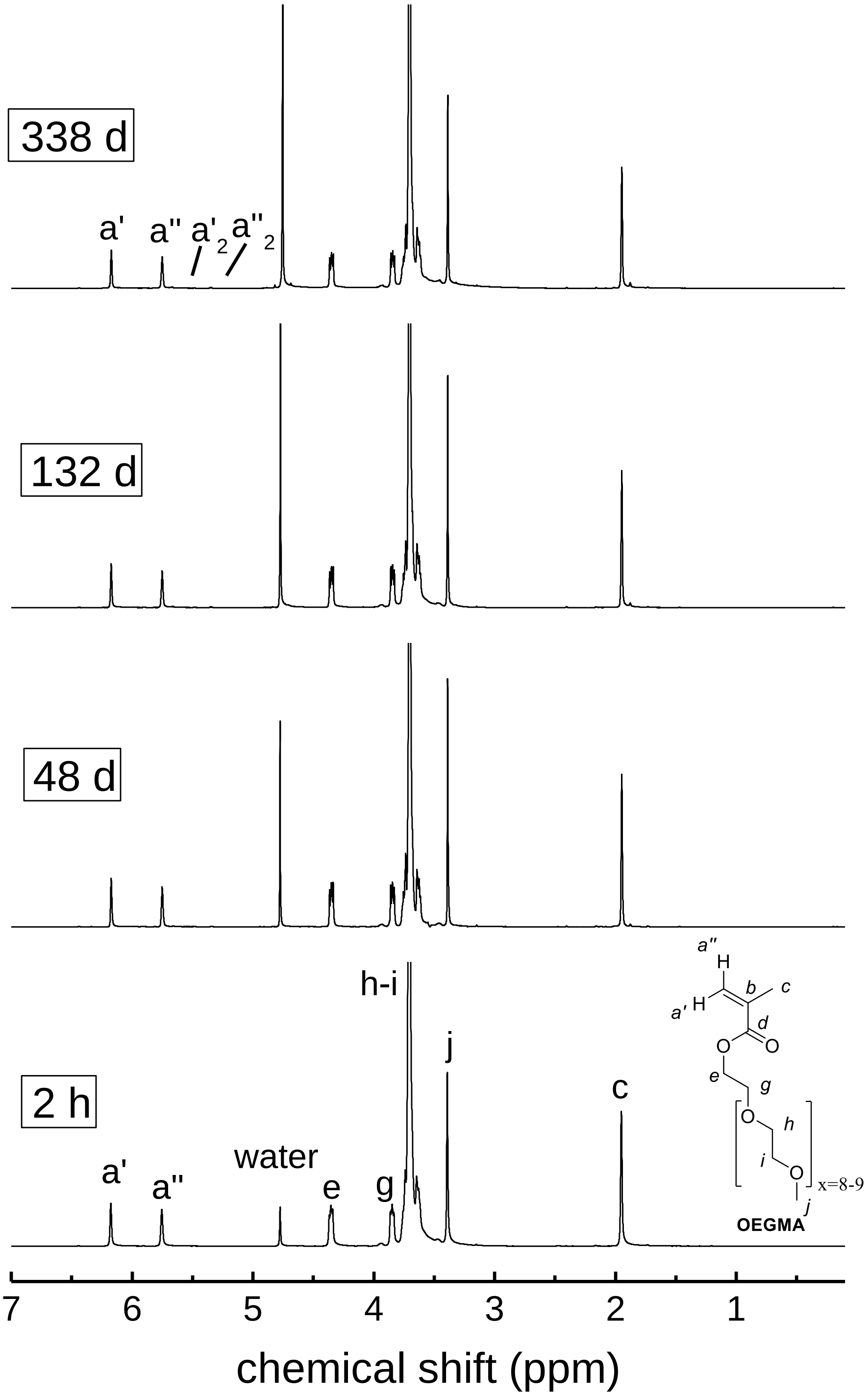


Figure S 22 $^{1}$H-NMR spectrum showing the degradation of 0.1 M solution of **OEGMA** in phosphate buffered saline (PBS) in $D_2O$ (pH = 7.4) at room temperature over time.

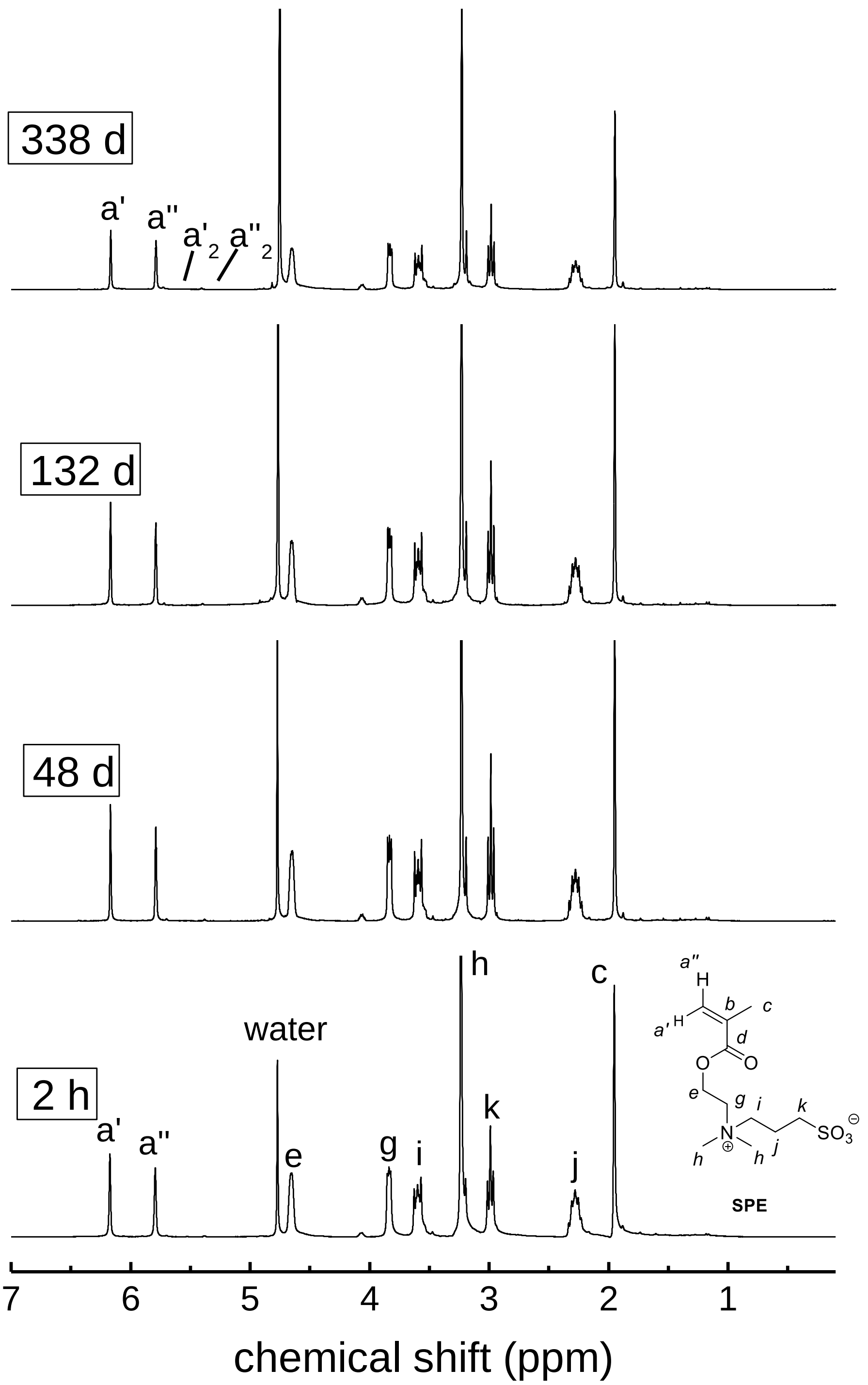


Figure S 23 $^{1}$H-NMR spectrum showing the degradation of 0.1 M solution of **SPE** in phosphate buffered saline (PBS) in $D_2O$ (pH = 7.4) at room temperature over time.

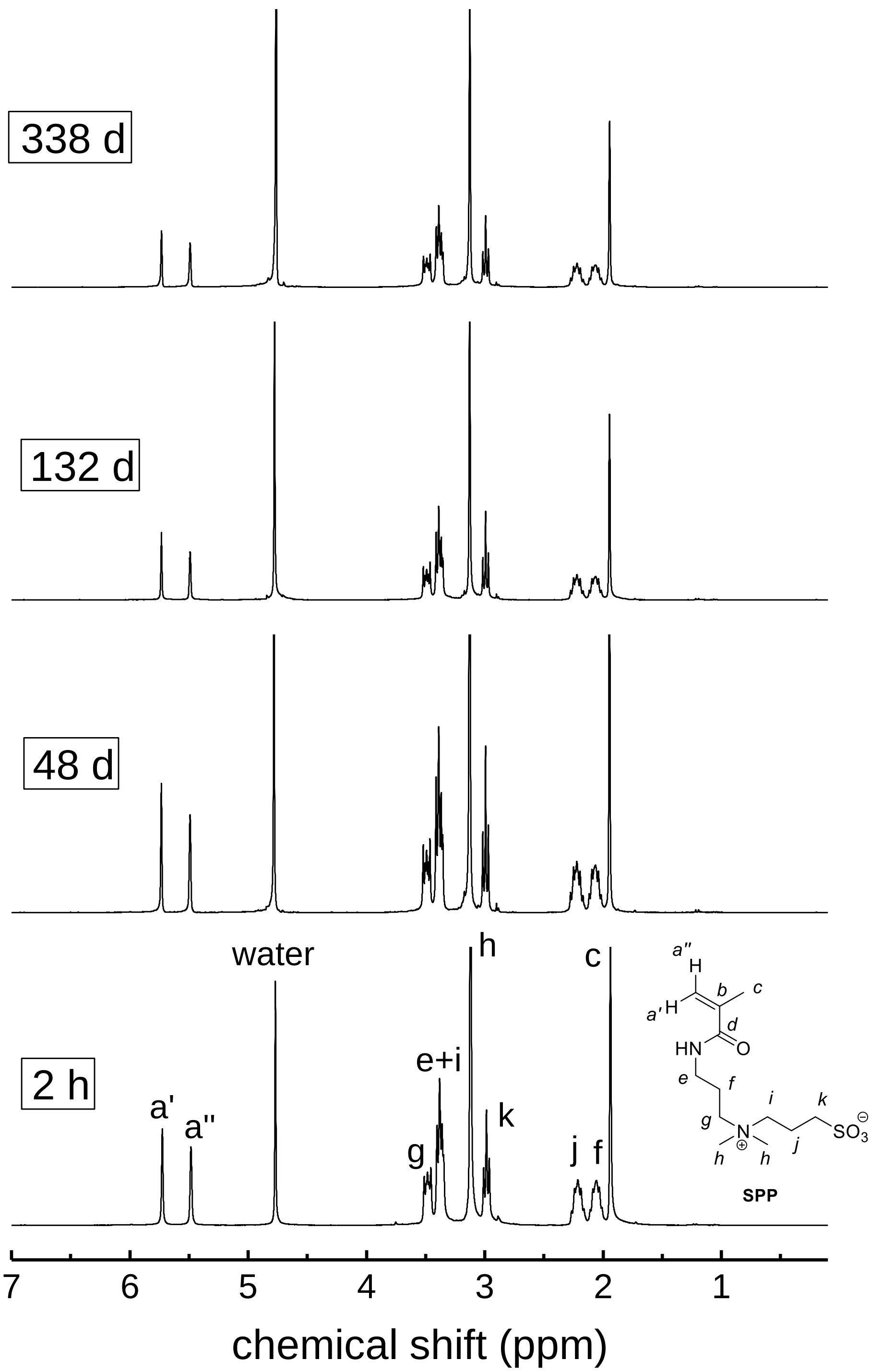


Figure S 24 $^{1}$H-NMR spectrum showing the degradation of 0.1 M solution of **SPP** in phosphate buffered saline (PBS) in $D_2O$ (pH = 7.4) at room temperature over time.

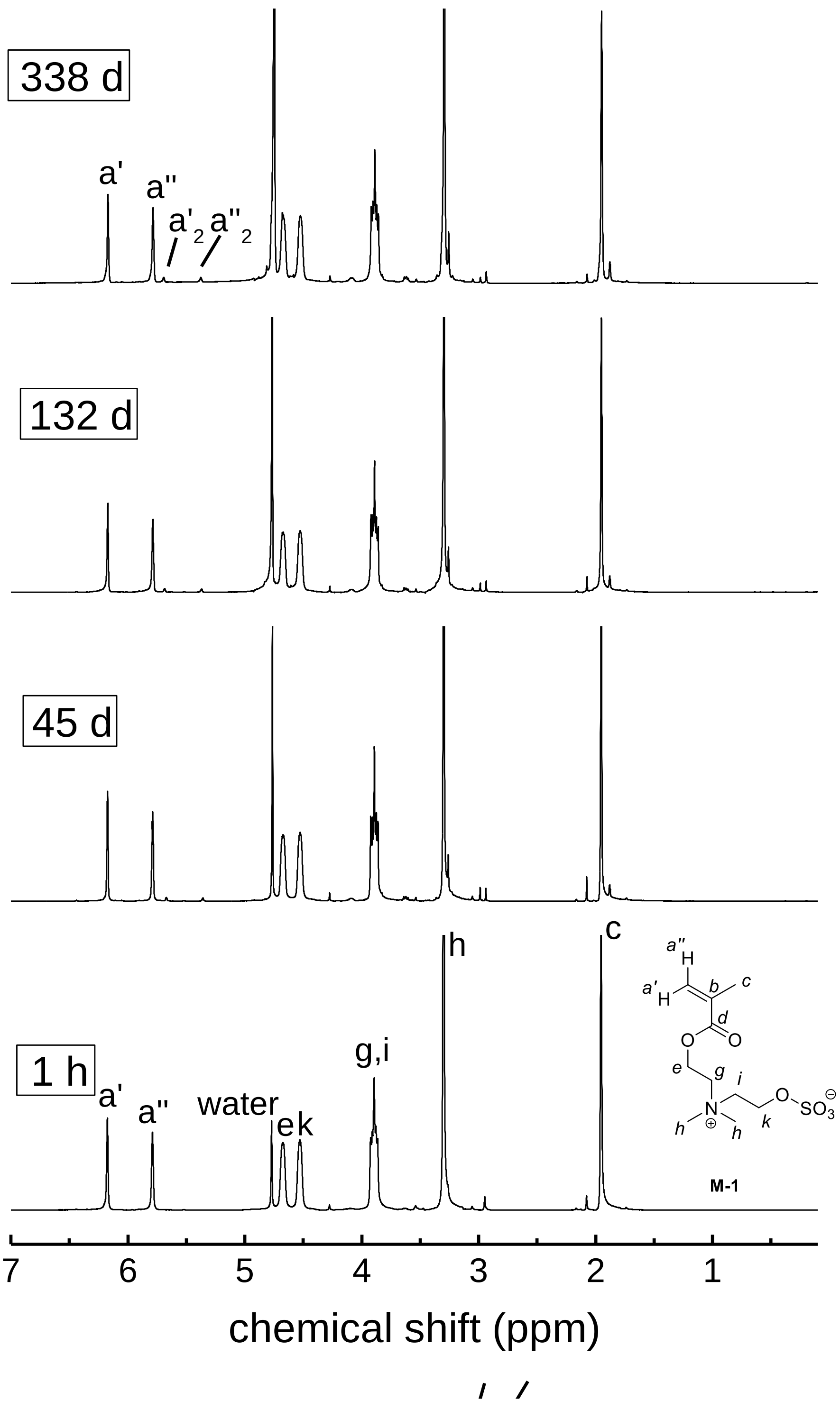


Figure S 25 $^{1}$H-NMR spectrum showing the degradation of 0.1 M solution of **M-1** in phosphate buffered saline (PBS) in $D_2O$ (pH = 7.4) at room temperature over time.

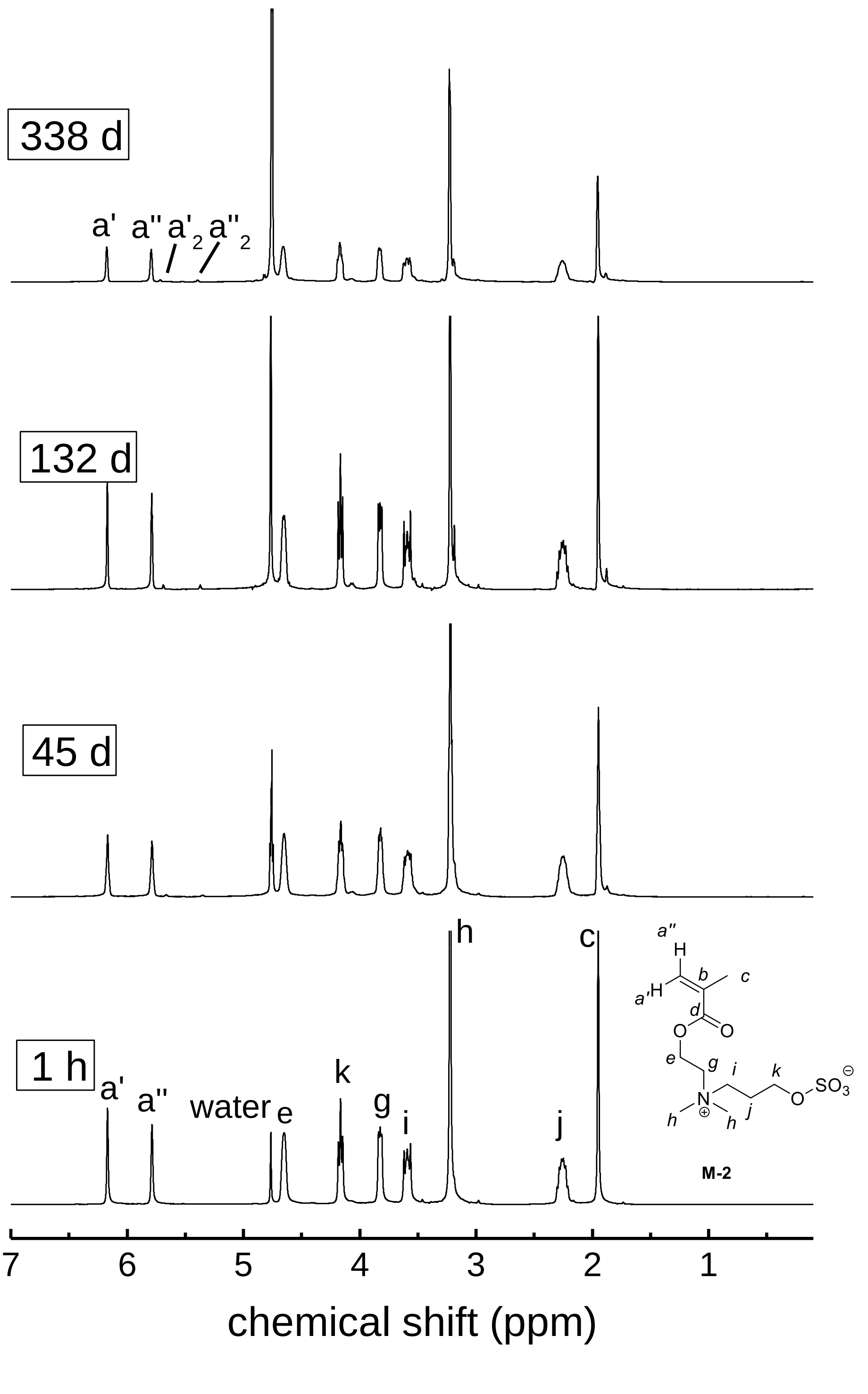


Figure S 26 $^{1}$H-NMR spectrum showing the degradation of 0.1 M solution of **M-2** in phosphate buffered saline (PBS) in $D_2O$ (pH = 7.4) at room temperature over time.

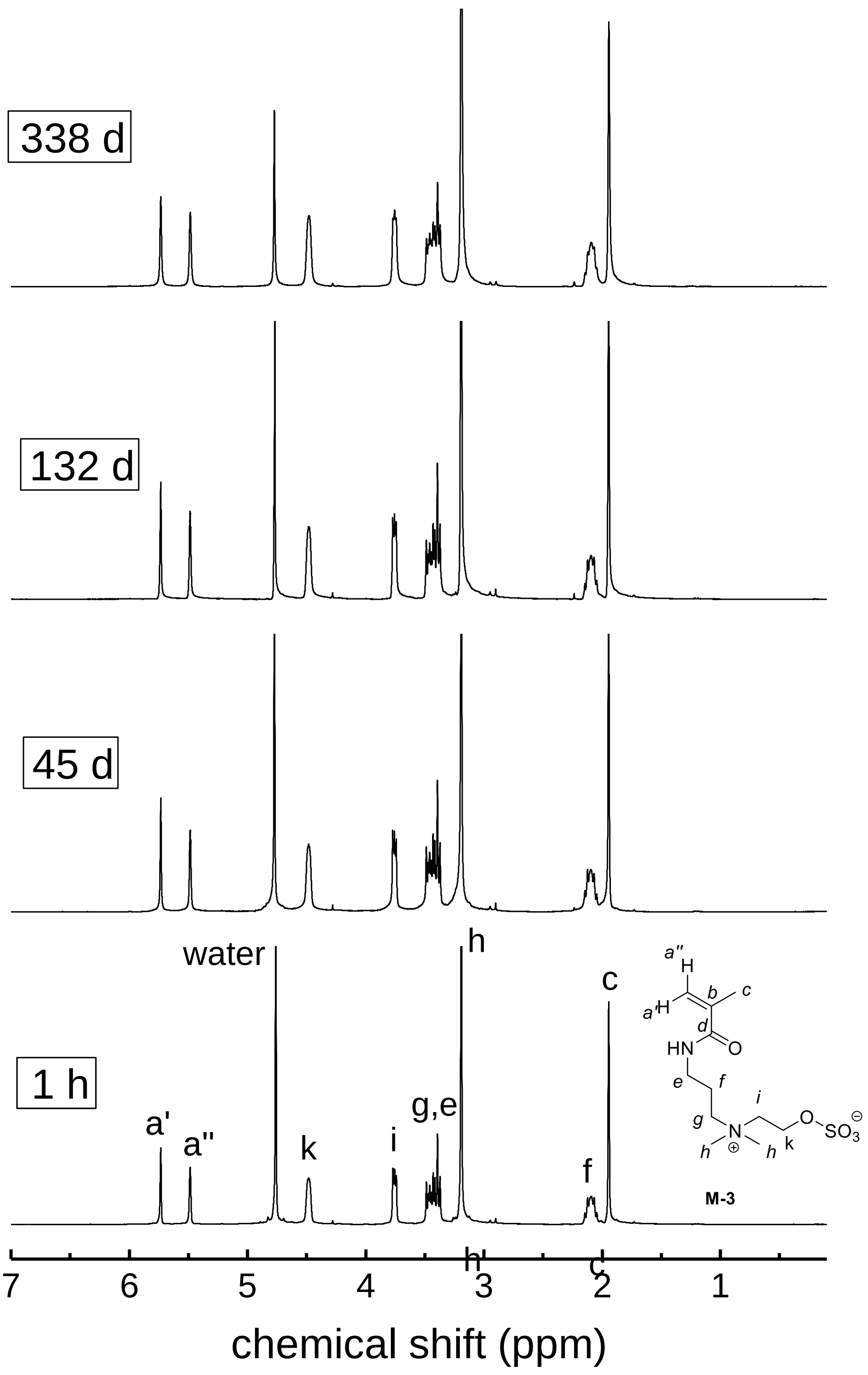


Figure S 27 $^{1}$H-NMR spectrum showing the degradation of 0.1 M solution of **M-3** in phosphate buffered saline (PBS) in $D_2O$ (pH = 7.4) at room temperature over time.

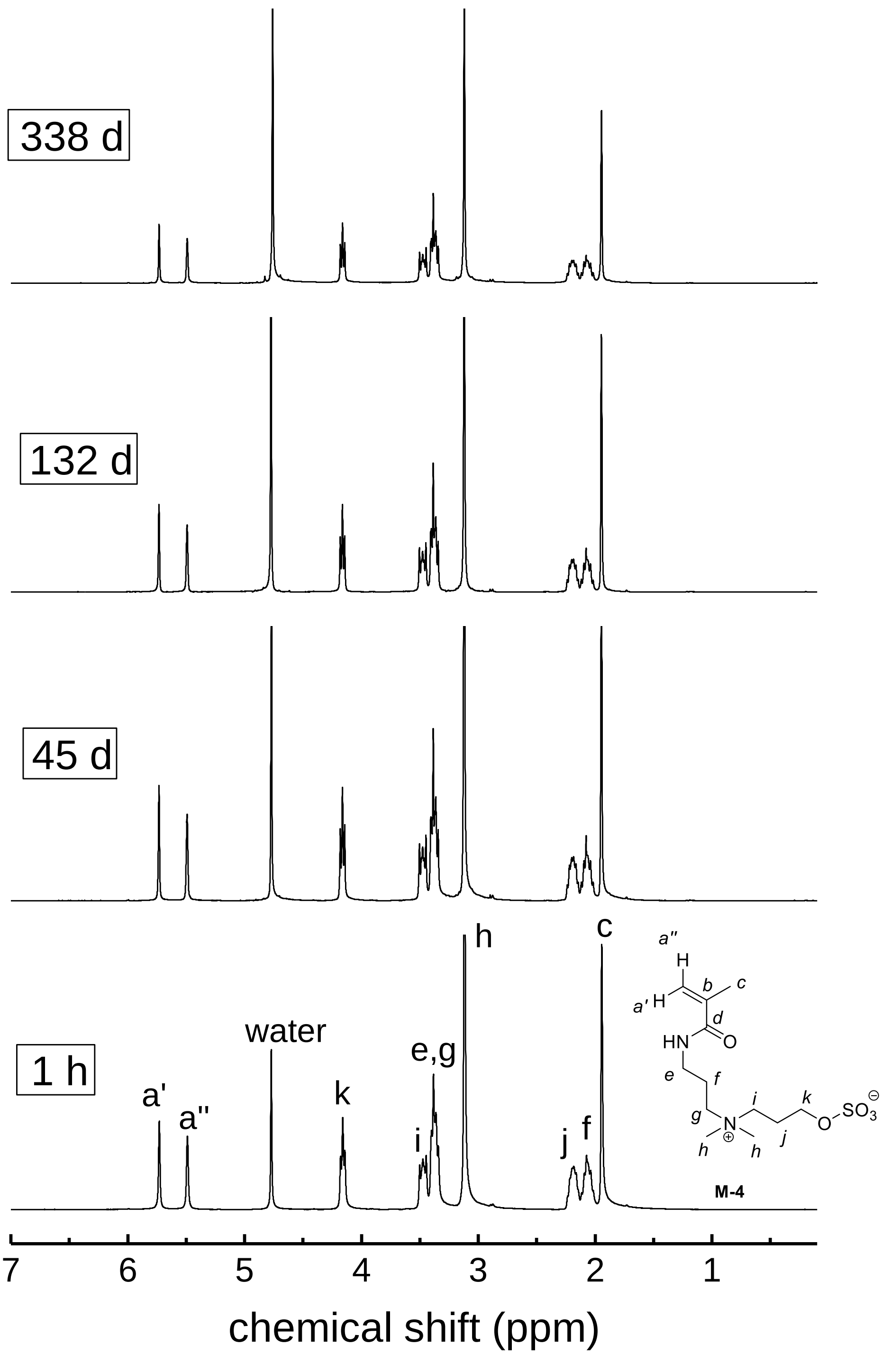


Figure S 28 $^{1}$H-NMR spectrum showing the degradation of 0.1 M solution of **M-4** in phosphate buffered saline (PBS) in $D_2O$ (pH = 7.4) at room temperature over time.

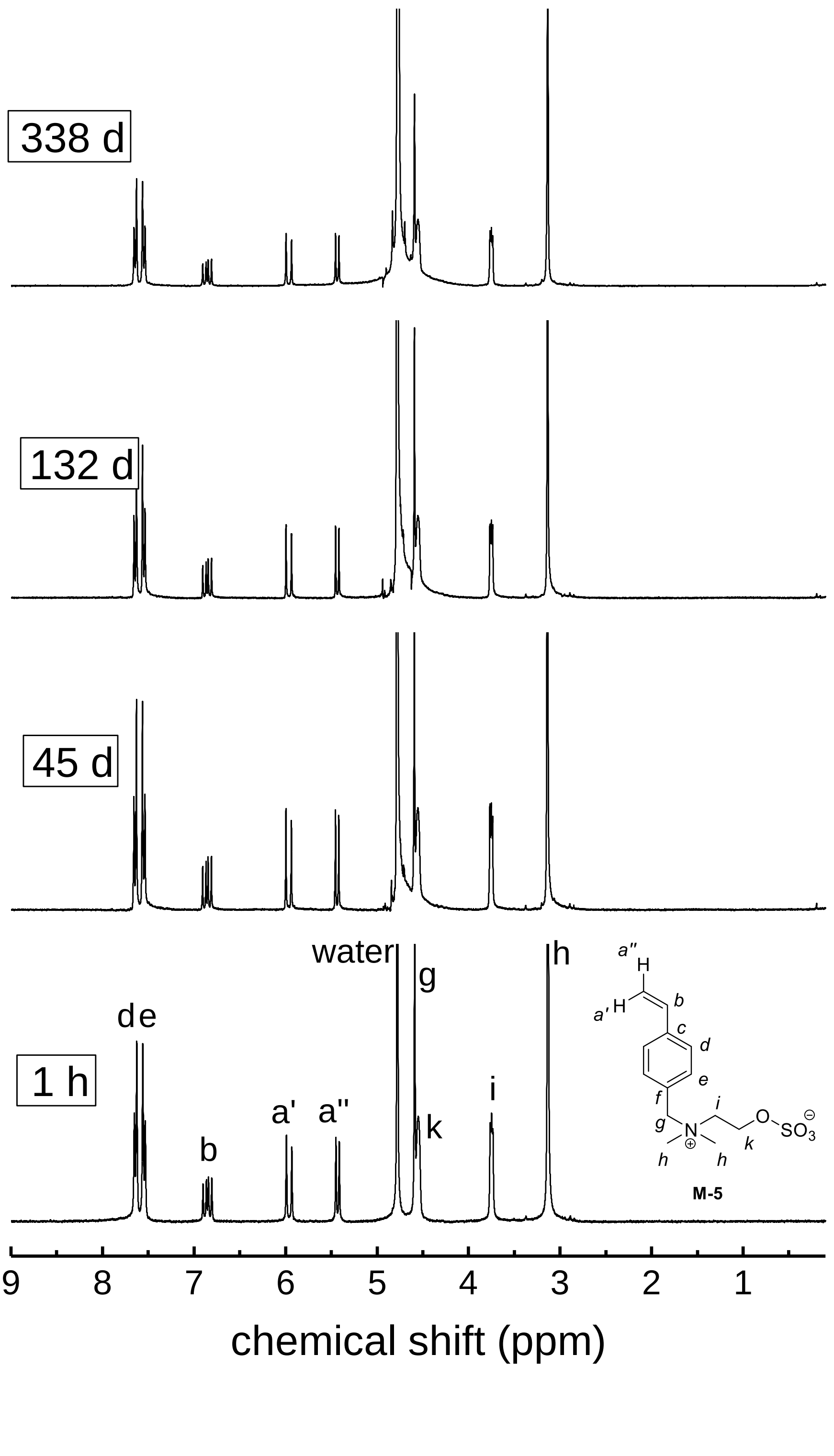


Figure S 29 $^{1}$H-NMR spectrum showing the degradation of 0.1 M solution of **M-5** in phosphate buffered saline (PBS) in $D_2O$ (pH = 7.4) at room temperature over time.

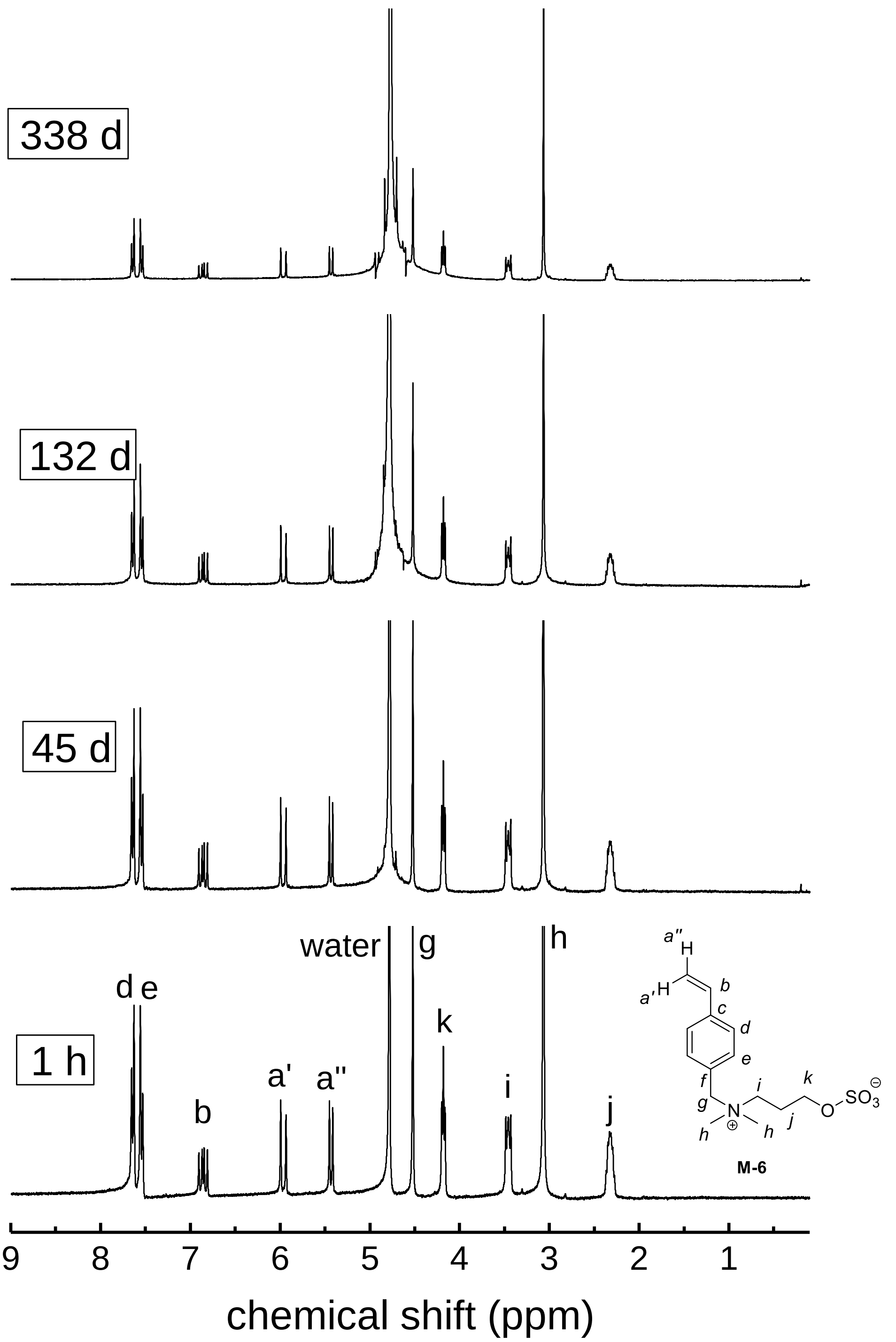


Figure S 30 $^{1}$H-NMR spectrum showing the degradation of 0.1 M solution of **M-6** in phosphate buffered saline (PBS) in $D_2O$ (pH = 7.4) at room temperature over time.

## 4.2. Monomer hydrolysis in 1 M hydrochloric acid pH=0

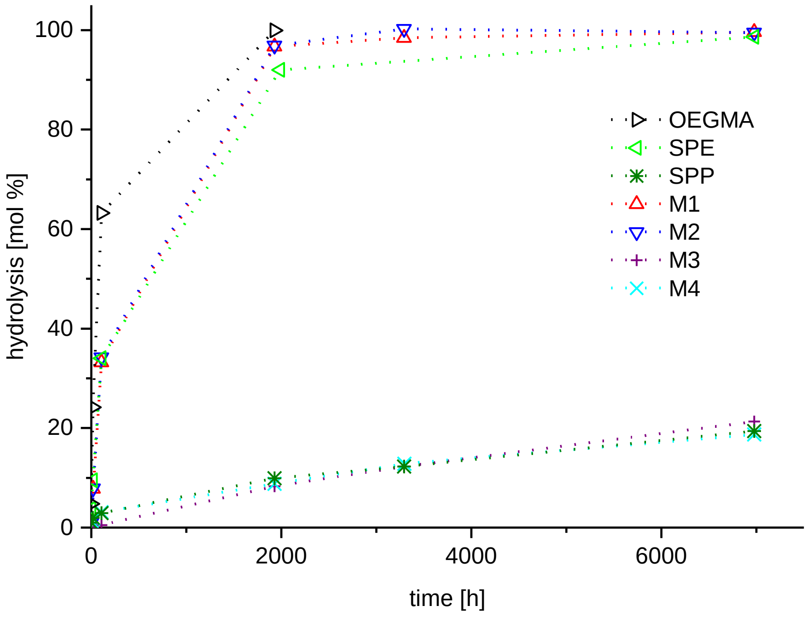


Figure S 31. Evolution of ester and amid hydrolysis of monomers in 1 M hydrochloric acid in $D_2O$ (pH=0): (□) = **OEGMA**, (□) = **SPE**, (*) = **SPP**, (□) = **M-1**, (□) = **M-2**, (+) = **M-3**, (X) = **M-4**.

Calculation of hydrolysis in mol %:

$$Hydrolyse_{\text{M-1}}\ [\text{mol}\ \%] = \left(\frac{I_{a'_2}*100}{I_{a'}+I_{a'_2}} + \frac{I_{a''_2}*100}{I_{a''}+I_{a''_2}}\right)/2$$

$$Hydrolyse_{M-2}\ [\text{mol}\ \%] = \left(\frac{I_{a'_2}*100}{I_{a'}+I_{a'_2}} + \frac{I_{a''_2}*100}{I_{a''}+I_{a''_2}}\right)/2$$

$$Hydrolyse_{M-3}\ [\text{mol}\ \%] = \left(\frac{I_{f_2}*100}{I_{f+f_3}+I_{f_2}}\right)$$

$$Hydrolyse_{M-4}\ [\text{mol}\ \%] = \left(\frac{I_{c_2}*100}{I_{c+c_3}+I_{c_2}}\right)$$

$$Hydrolyse_{OEGMA}\ [\text{mol}\ \%] = \left(\frac{I_{c_2}*100}{I_{c+c_3}+I_{c_2}}\right)$$

$$Hydrolyse_{SPE}\ [\text{mol}\ \%] = \left(\frac{I_{a''_2}*100}{I_{a'}+I_{a''_2}} + \frac{I_{a''_2}*100}{I_{a''}+I_{a''_2}}\right)/2$$

$$Hydrolyse_{SPP}\ [\text{mol}\ \%] = \left(\frac{I_{c_2}*100}{I_{c+c_3}+I_{c_2}}\right)$$

The Index 2 in e.g. $I_{e_2}$ indicates the hydrolysis product of the ester/amid product, while no index e.g. $I_{a''}$ determines the unchanged molecule without hydrolysis.

$I_{a'}(M\text{-}1,\ range\ in\ ppm)$ = 6.2-6.1
$I_{a_2'}(M\text{-}1,\ range\ in\ ppm)$ = 6.1-6.0
$I_{a''}(M\text{-}1,\ range\ in\ ppm)$ = 5.9-5.8
$I_{a_2''}(M\text{-}1,\ range\ in\ ppm)$ = 5.8-5.7
$I_{a'}(M\text{-}2,\ range\ in\ ppm)$ = 6.4-6.2
$I_{a_2'}(M\text{-}2,\ range\ in\ ppm)$ = 6.2-6.0
$I_{a''}(M\text{-}2,\ range\ in\ ppm)$ = 6.0-5.9
$I_{a_2''}(M\text{-}2,\ range\ in\ ppm)$ = 5.9-5.6
$I_{f+f_3}(M\text{-}3,\ range\ in\ ppm)$ = 2.4-2.2
$I_{f_2}(M\text{-}3,\ range\ in\ ppm)$ = 2.2-2.0
$I_{c+c_3}(M\text{-}4,\ range\ in\ ppm)$ = 2.0-1.9
$I_{c_2}(M\text{-}4,\ range\ in\ ppm)$ = 1.9-1.8
$I_{c+c_3}(OEGMA,\ range\ in\ ppm)$ = 2.1-2.0
$I_{c_2}(OEGMA,\ range\ in\ ppm)$ = 2.1-1.9
$I_{a'}(SPE,\ range\ in\ ppm)$ = 6.3-6.1
$I_{a_2'}(SPE,\ range\ in\ ppm)$ = 6.1-6.0
$I_{a''}(SPE,\ range\ in\ ppm)$ = 5.8-5.7
$I_{a_2''}(SPE,\ range\ in\ ppm)$ = 5.7.5.6
$I_{c+c_3}(SPP,\ range\ in\ ppm)$ = 2.0-1.9
$I_{c_2}(SPP,\ range\ in\ ppm)$ = 1.9-1.8

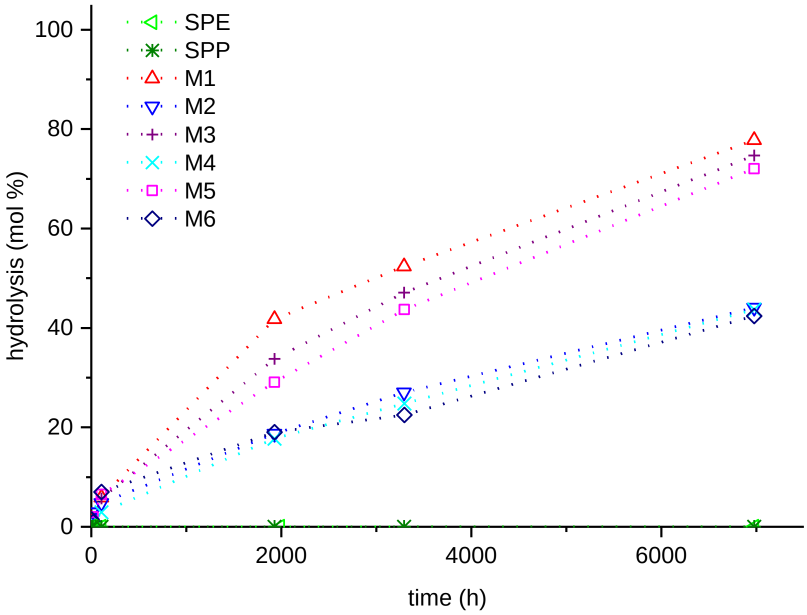


Figure S 32. Evolution of sulfate and sulfonate hydrolysis of monomers in 1 M hydrochloric acid in $D_2O$ (pH=0): (□) = SPE, (*) = SPP, (□) = M1, (□) = M2, (+) = M3, (X) = M4, (□) = M5, (□) = M6.

<u>Calculation of hydrolysis in mol %:</u>

$$Hydrolyse_{\text{M-1}}\ [\text{mol }\%] = \left(\frac{[\mathrm{I}_{k_3+e_3} - 2 * \mathrm{I}_{a_2'}]/2}{[\mathrm{I}_{g+i} - 2 * \ \mathrm{I}_{a_2'}]/2 + [\mathrm{I}_{k_3+e_3} - 2 * \mathrm{I}_{a_2'}]/2} * 100\right)$$

$$Hydrolyse_{M-2}\ [\text{mol }\%] = \left(\frac{\mathrm{I}_{j_3} * 100}{\mathrm{I}_{j_2} + \mathrm{I}_{j_3}}\right)$$

$$Hydrolyse_{M-3}\ [\text{mol }\%] = \left(\frac{\mathrm{I}_{k_3} * 100}{\mathrm{I}_{k} + \mathrm{I}_{k_3}}\right)$$

$$Hydrolyse_{M-4}\ [\text{mol }\%] = \left(\frac{\mathrm{I}_{k_3} * 100}{\mathrm{I}_{k} + \mathrm{I}_{k_3}}\right)$$

$$Hydrolyse_{M-5}\ [\text{mol }\%] = \left(\frac{\mathrm{I}_{i_3} * 100}{\mathrm{I}_{i} + \mathrm{I}_{i_3}}\right)$$

$$Hydrolyse_{M-6}\ [\text{mol }\%] = \left(\frac{\mathrm{I}_{j_3} * 100}{\mathrm{I}_{j_2} + \mathrm{I}_{j_3}}\right)$$

$$Hydrolyse_{SPE}\ [\text{mol }\%] = \left(\frac{\mathrm{I}_{k_3} * 100}{\mathrm{I}_{k+k_2} + \mathrm{I}_{k_3}}\right)$$

$$Hydrolyse_{SPP}\ [\text{mol }\%] = \left(\frac{\mathrm{I}_{k_3} * 100}{\mathrm{I}_{k} + \mathrm{I}_{k_3}}\right)$$

The Index 2 in e.g. $\mathrm{I}_{e_2}$ indicates the hydrolysis product of the ester/amid product, while no index e.g. $\mathrm{I}_{a''}$ determines the unchanged molecule without hydrolysis

$\mathrm{I}_{k_3+e_3}(M\text{-}1,\ range\ in\ ppm)$ = 4.6-4.4
$\mathrm{I}_{a_2'}(M\text{-}1,\ range\ in\ ppm)$ = 6.1-6.2
$\mathrm{I}_{g+i}(M\text{-}1,\ range\ in\ ppm)$ = 4.2-4.0
$\mathrm{I}_{j_3}(M\text{-}2,\ range\ in\ ppm)$ = 2.2-2.0
$\mathrm{I}_{j_2}(M\text{-}2,\ range\ in\ ppm)$ = 2.4-2.2
$\mathrm{I}_{k_3}(M\text{-}3,\ range\ in\ ppm)$ = 4.2-3.9
$\mathrm{I}_{k}(M\text{-}3,\ range\ in\ ppm)$ = 4.6-4.3
$\mathrm{I}_{k_3}(M\text{-}4,\ range\ in\ ppm)$ = 3.8-3.6
$\mathrm{I}_{k}(M\text{-}4,\ range\ in\ ppm)$ = 4.3-4.0
$\mathrm{I}_{i_3}(M\text{-}5,\ range\ in\ ppm)$ = 3.6-3.4
$\mathrm{I}_{i}(M\text{-}5,\ range\ in\ ppm)$ = 3.9-3.6
$\mathrm{I}_{j_3}(M\text{-}6,\ range\ in\ ppm)$ = 2.2-2.0
$\mathrm{I}_{j}(M\text{-}6,\ range\ in\ ppm)$ = 2.4-2.2
$\mathrm{I}_{k_3}(SPE,\ range\ in\ ppm)$ = no signal
$\mathrm{I}_{k+k_2}(SPE,\ range\ in\ ppm)$ = 3.1-2.9
$\mathrm{I}_{k_3}(SPP,\ range\ in\ ppm)$ = no signal
$\mathrm{I}_{k}(SPP,\ range\ in\ ppm)$ = 3.1-2.9

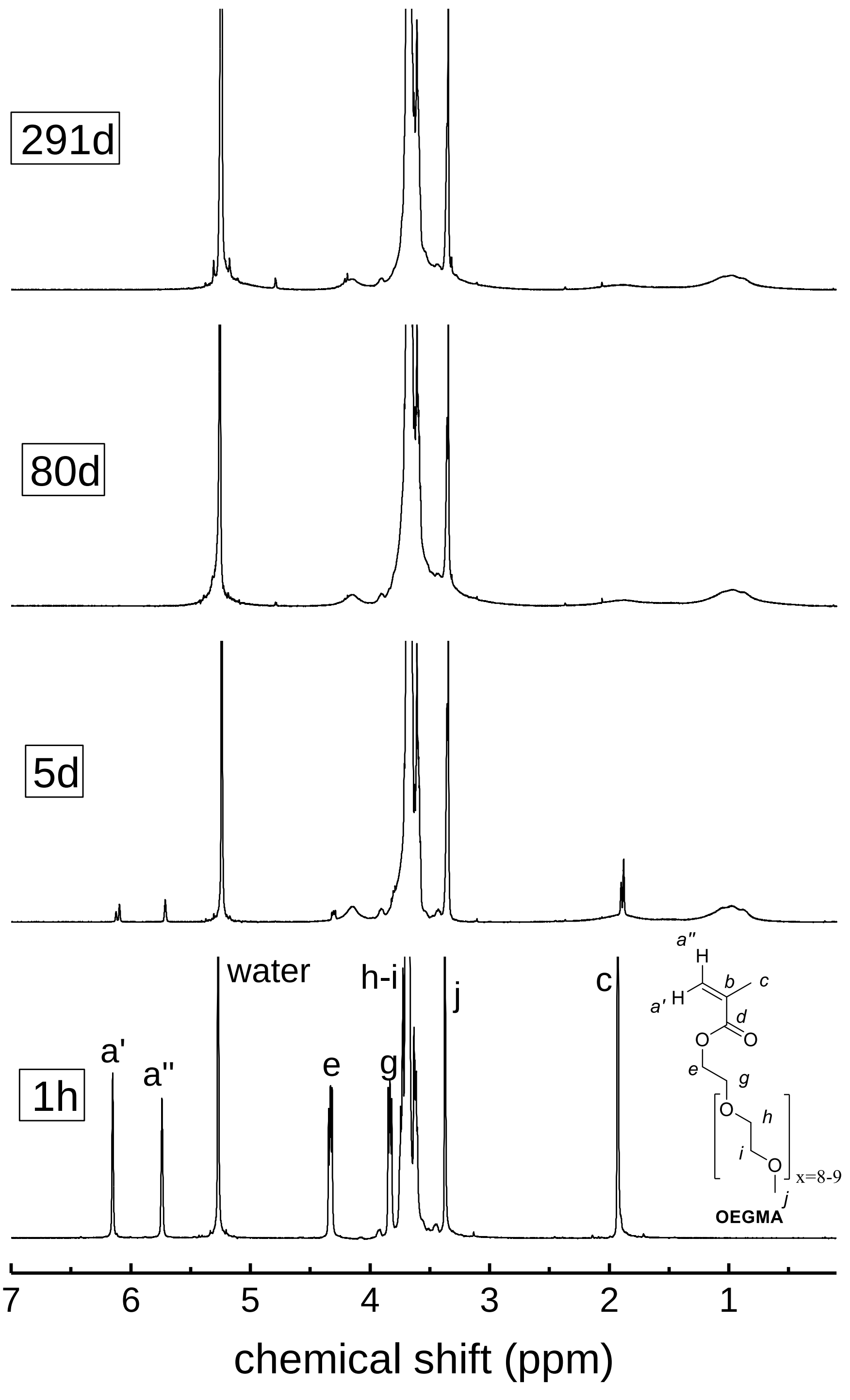


Figure S 33 $^{1}$H-NMR spectrum showing the degradation of 0.1 M solution of **OEGMA** in hydrochloric acid in $D_2O$ (pH = 0) at room temperature over time.

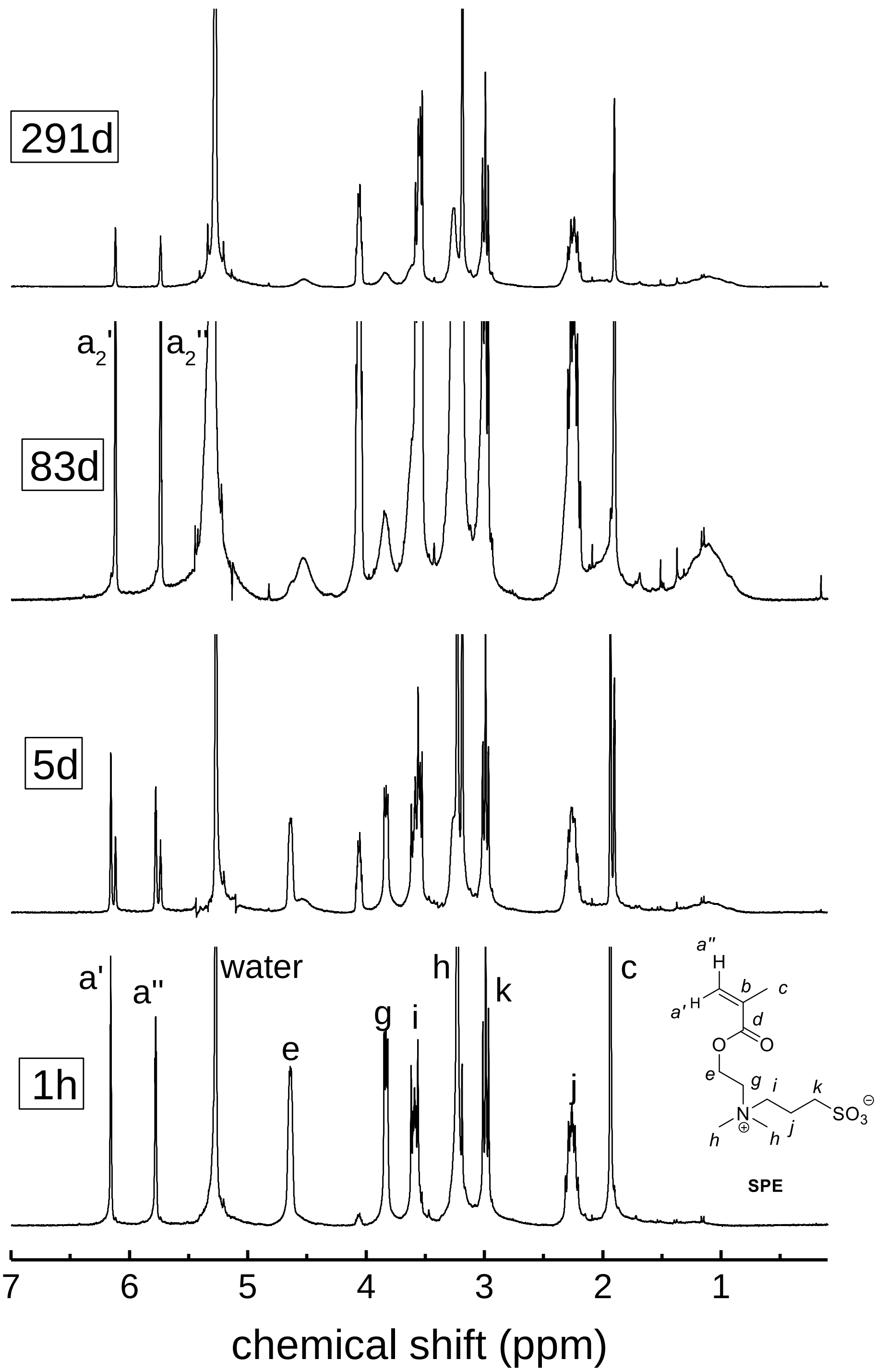


Figure S 34 $^{1}$H-NMR spectrum showing the degradation of 0.1 M solution of **SPE** in hydrochloric acid in $D_2O$ (pH = 0) at room temperature over time.

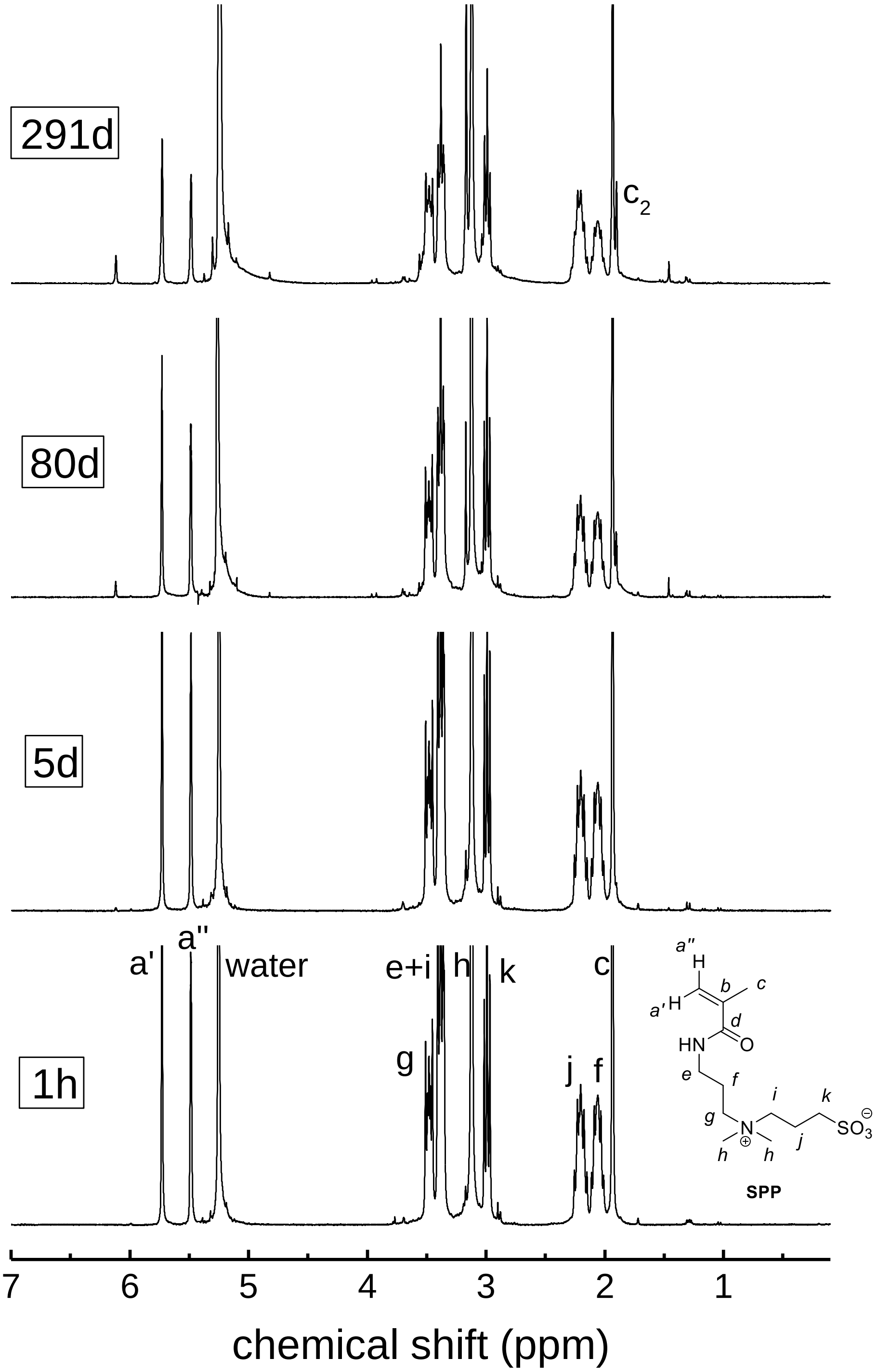


Figure S 35 $^{1}$H-NMR spectrum showing the degradation of 0.1 M solution of **SPP** in hydrochloric acid in $D_2O$ (pH = 0) at room temperature over time.

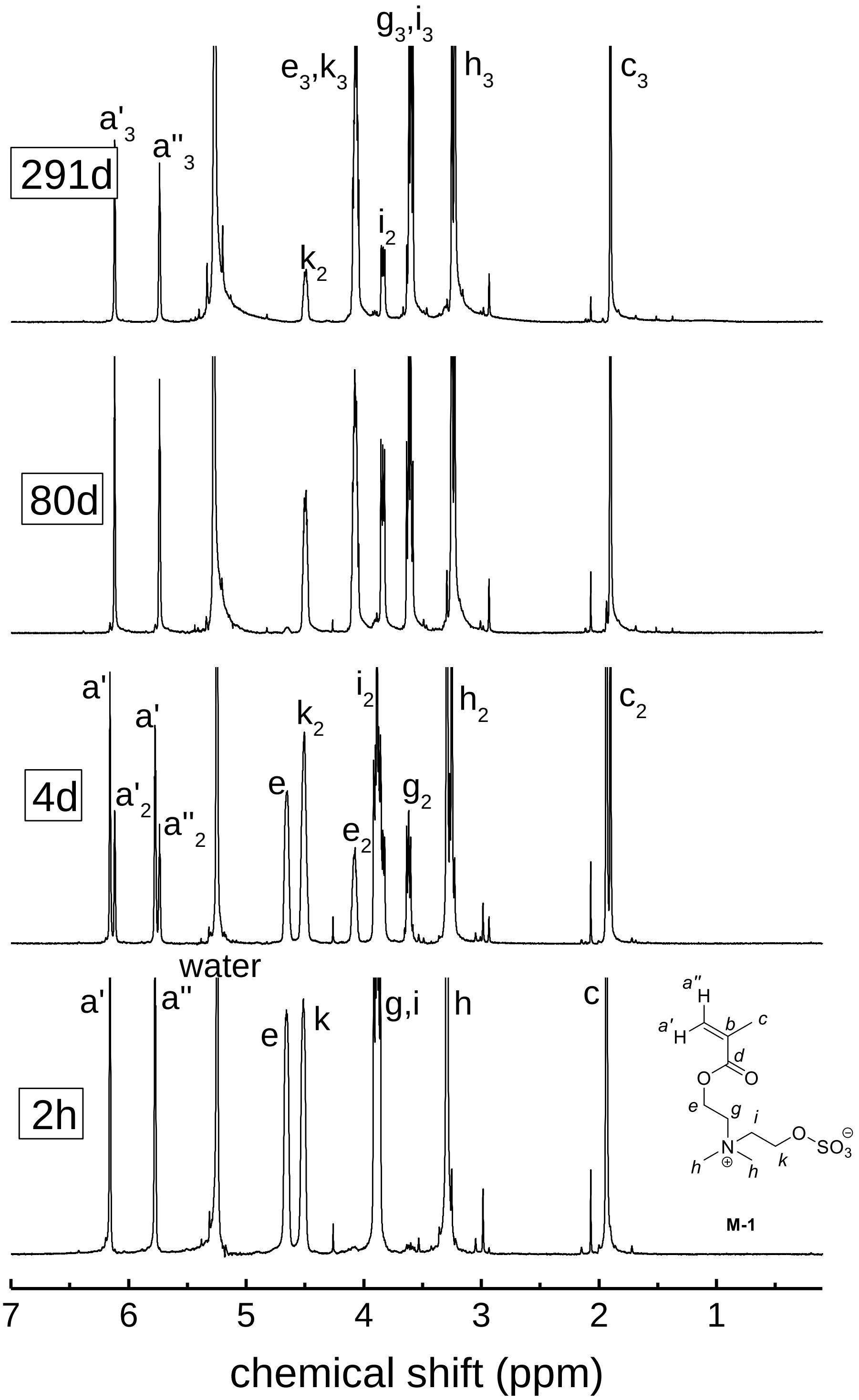


Figure S 36 $^{1}$H-NMR spectrum showing the degradation of 0.1 M solution of **M-1** in hydrochloric acid in $D_2O$ (pH = 0) at room temperature over time.

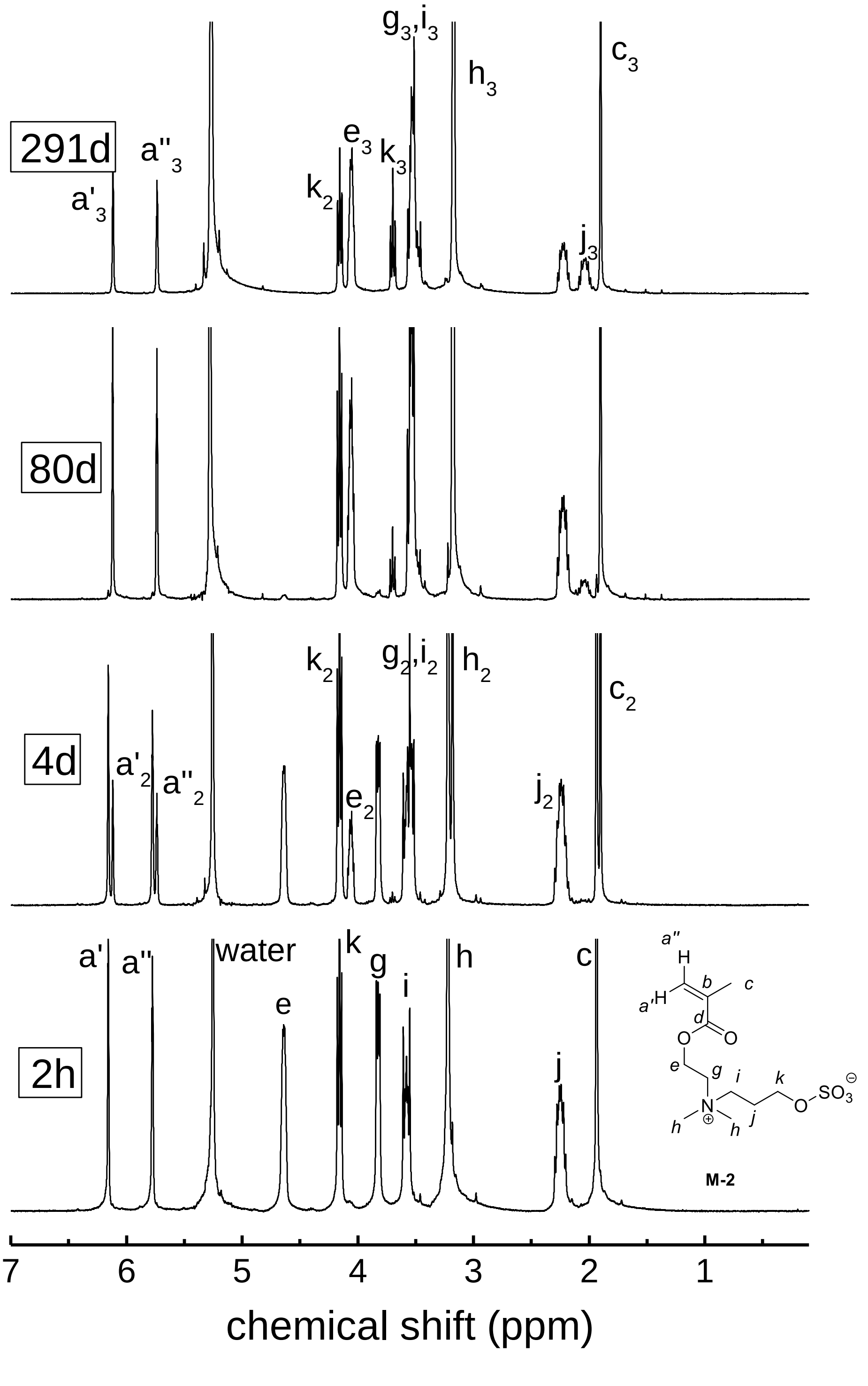


Figure S 37 $^{1}$H-NMR spectrum showing the degradation of 0.1 M solution of **M-2** in hydrochloric acid in $D_2O$ (pH = 0) at room temperature over time.

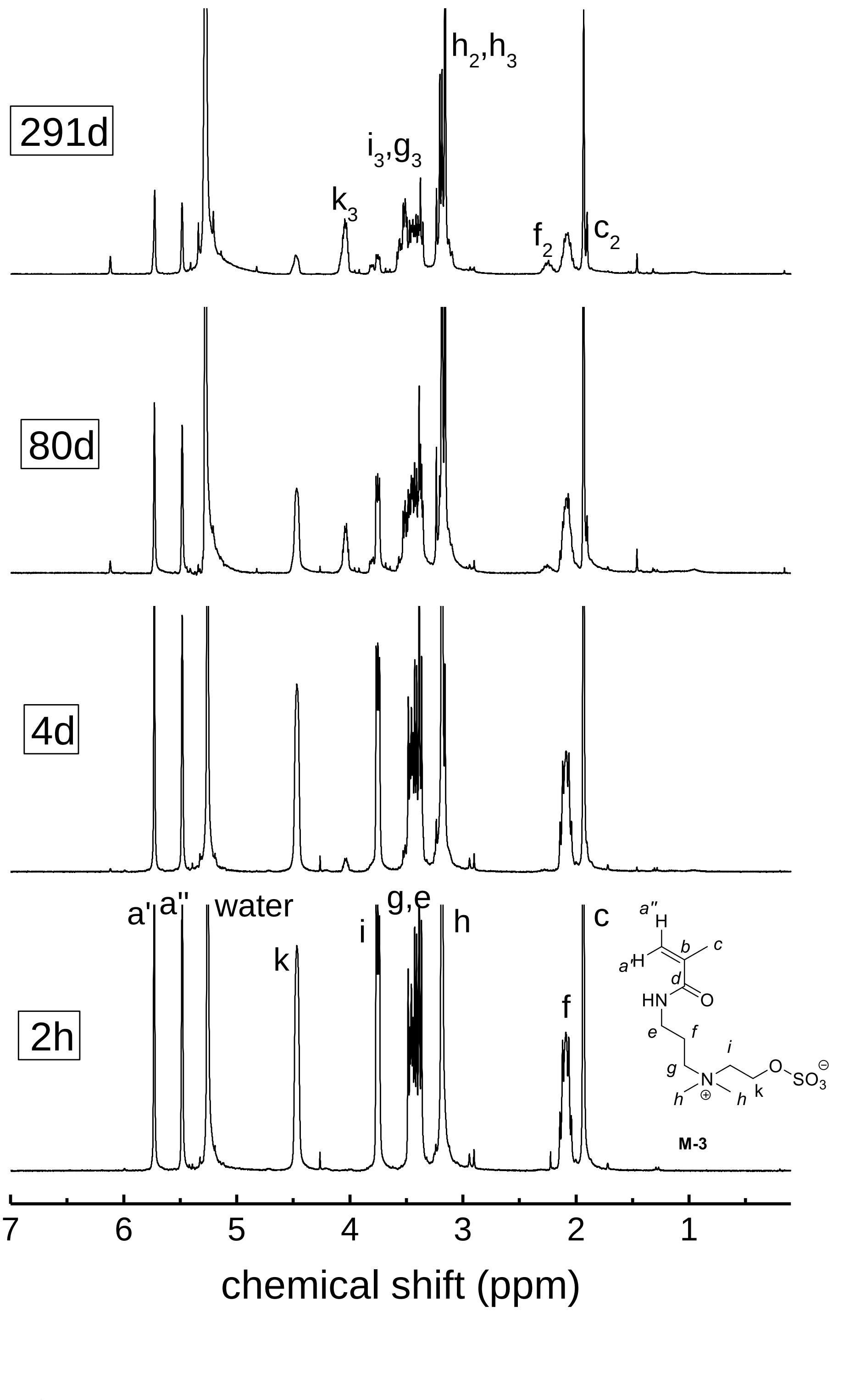


Figure S 38 $^{1}$H-NMR spectrum showing the degradation of 0.1 M solution of **M-3** in hydrochloric acid in $D_2O$ (pH = 0) at room temperature over time.

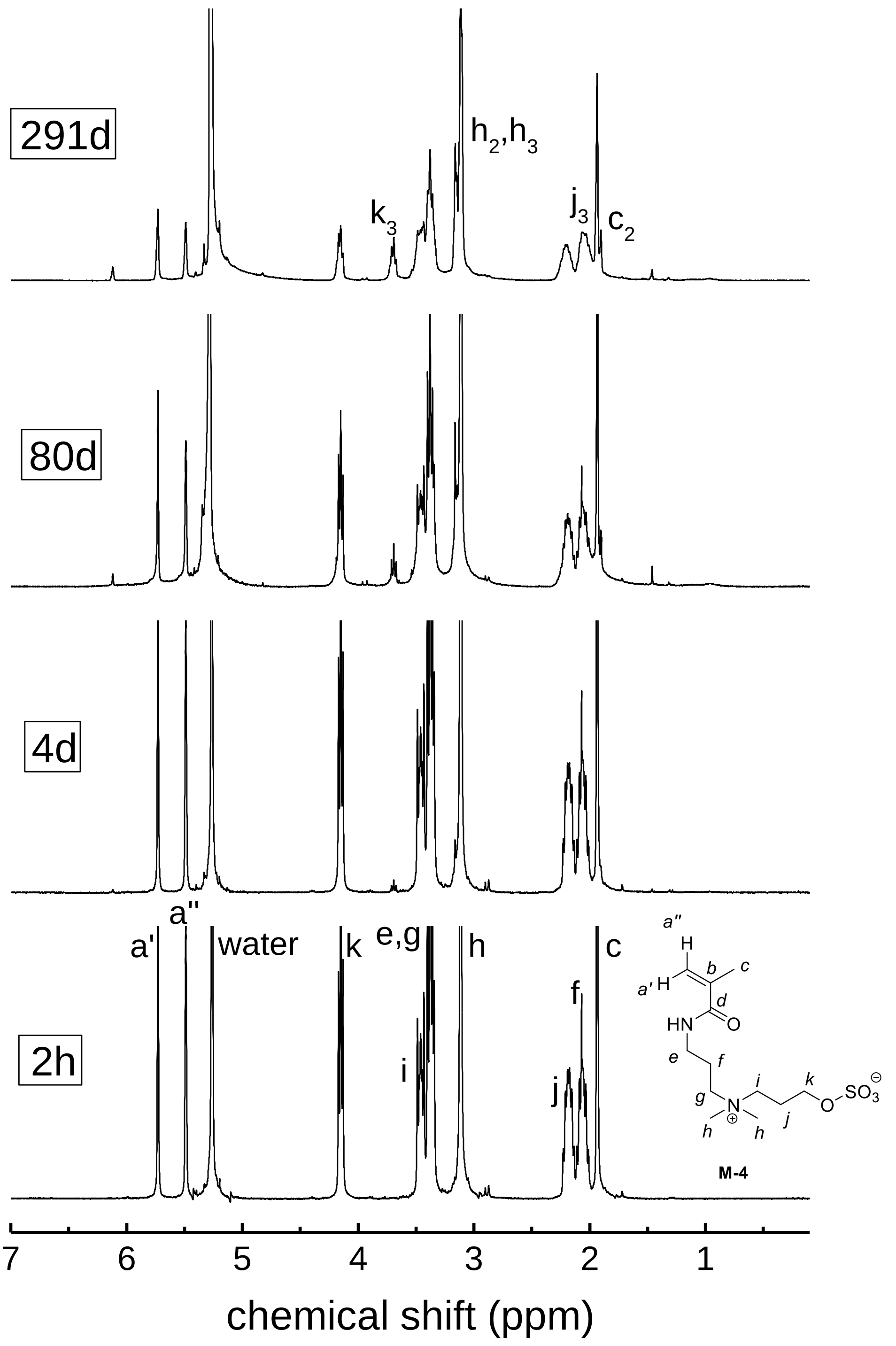


Figure S 39 $^{1}$H-NMR spectrum showing the degradation of 0.1 M solution of **M-4** in hydrochloric acid in $D_2O$ (pH = 0) at room temperature over time.

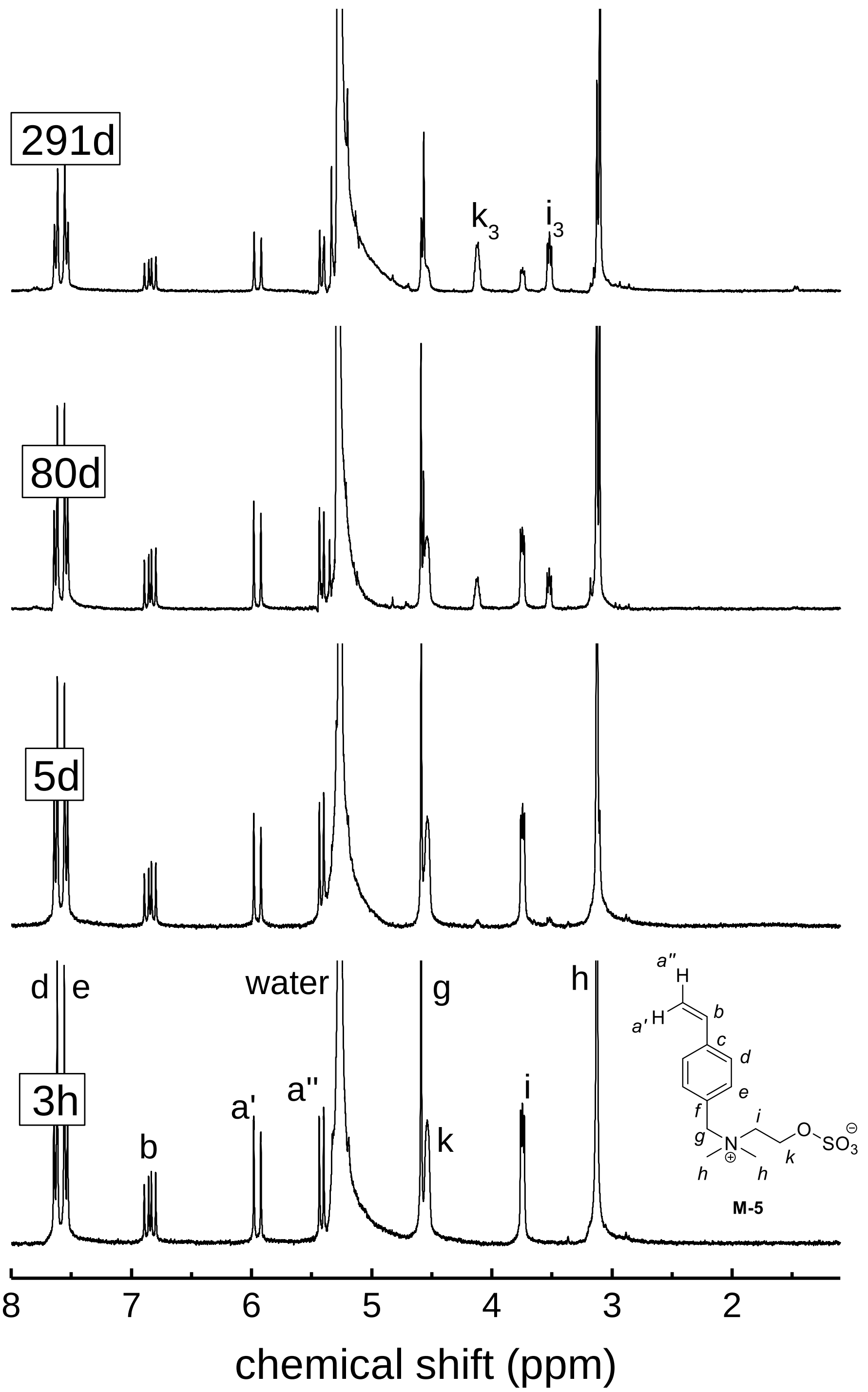


Figure S 40 $^{1}$H-NMR spectrum showing the degradation of 0.1 M solution of **M-5** in hydrochloric acid in $D_2O$ (pH = 0) at room temperature over time.

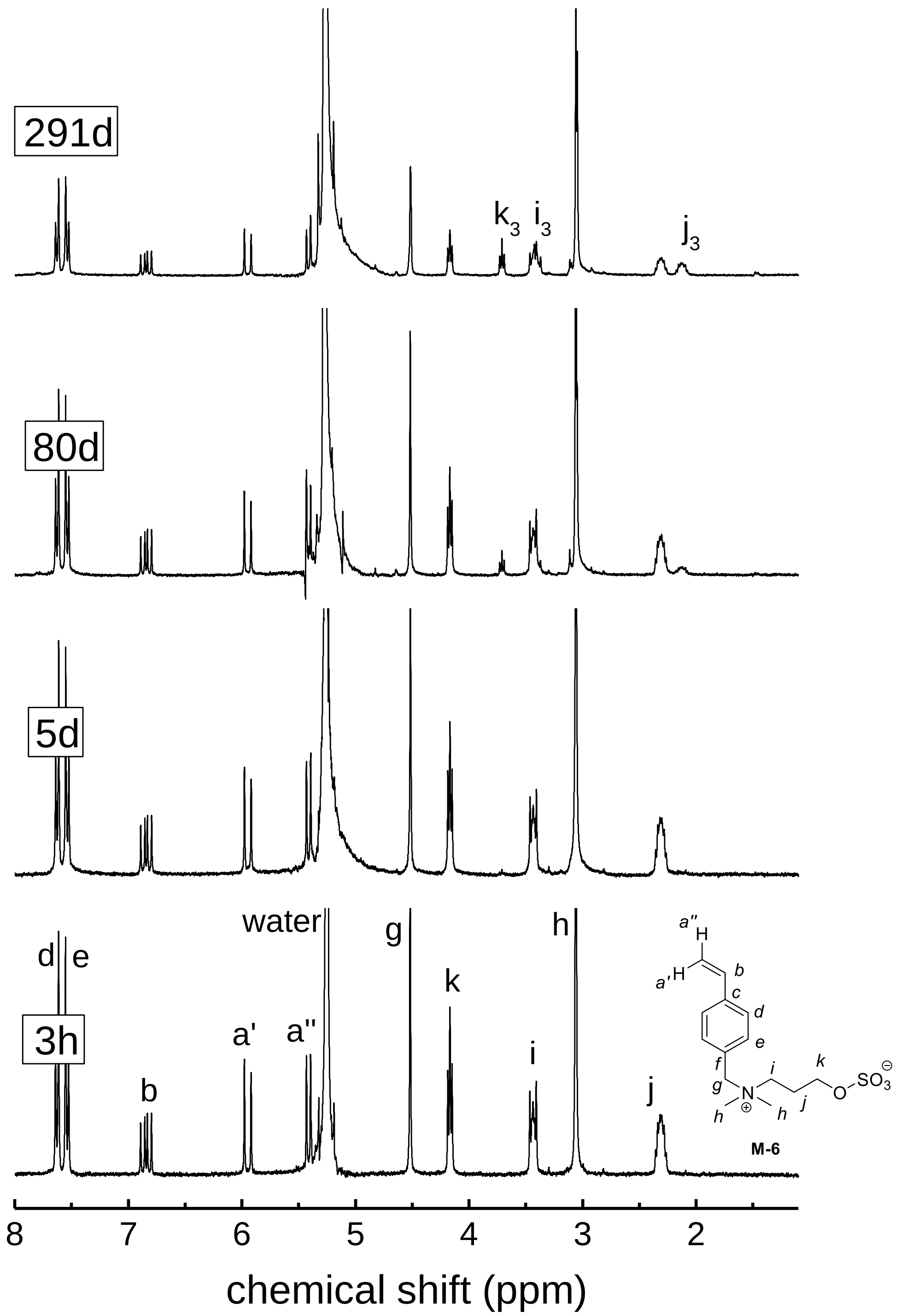


Figure S 41 $^{1}$H-NMR spectrum showing the degradation of 0.1 M solution of **M-6** in hydrochloric acid in $D_2O$ (pH = 0) at room temperature over time.

*4.1. 2D-Spektra ($^{1}$H-$^{1}$H-COSY) - Monomer hydrolysis pH=0*

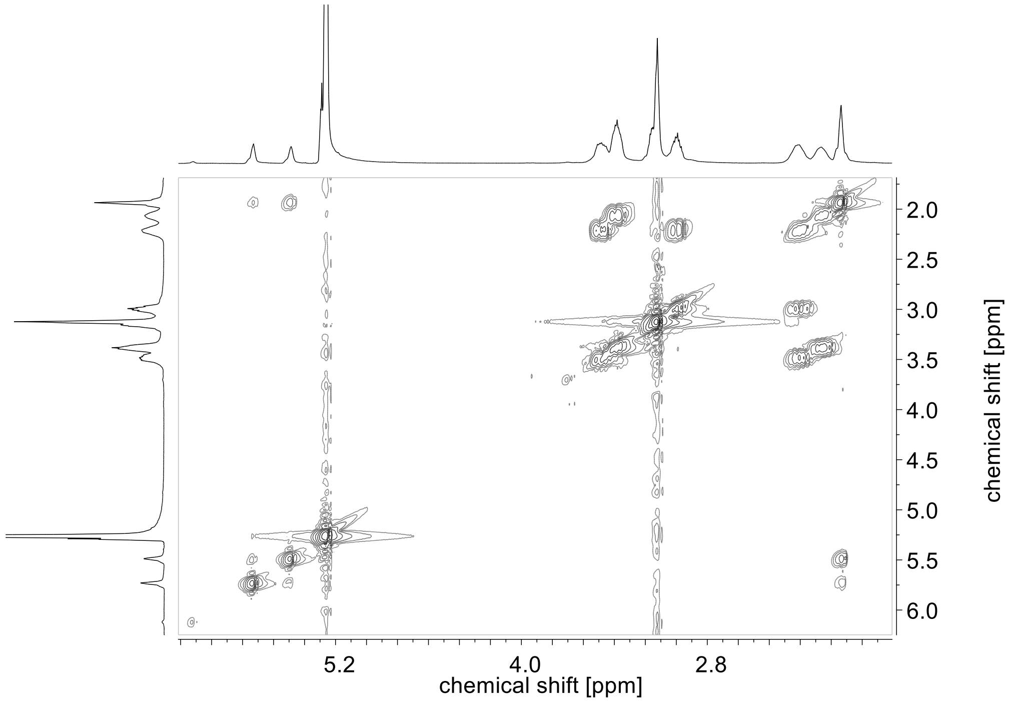


Figure S 42 $^{1}$H-$^{1}$H-COSY NMR spectra of 0.1 M solution of **SPP** in hydrochloric acid in $D_2O$, after 291 days.

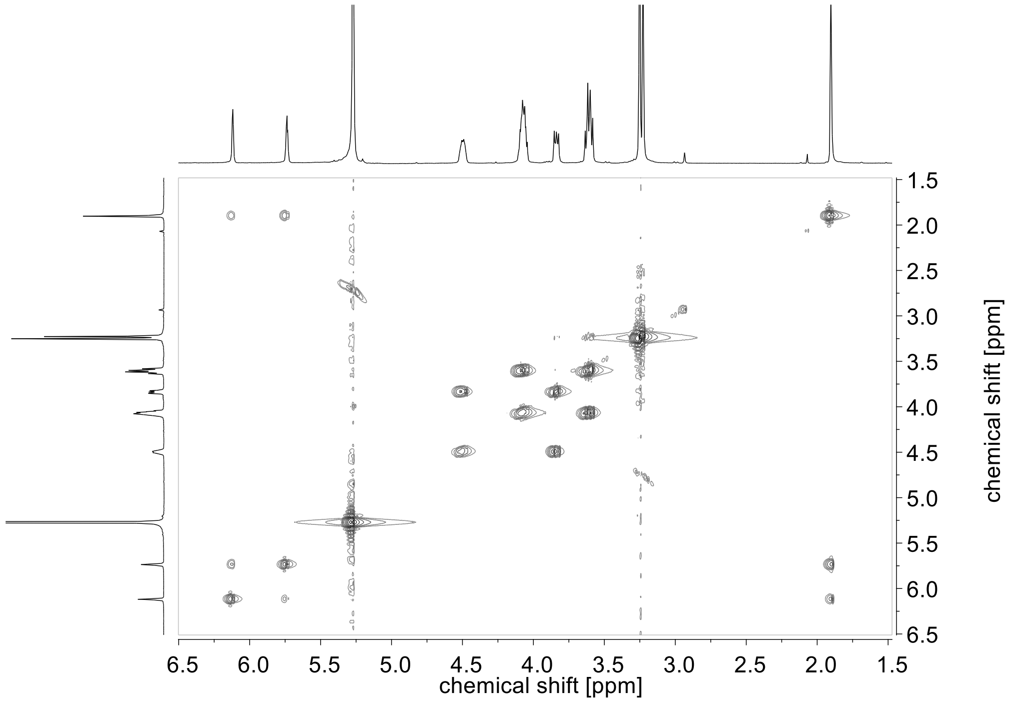


Figure S 43 $^{1}$H-$^{1}$H-COSY NMR spectra of 0.1 M solution of **M-1** in hydrochloric acid in $D_2O$, after 291 days.

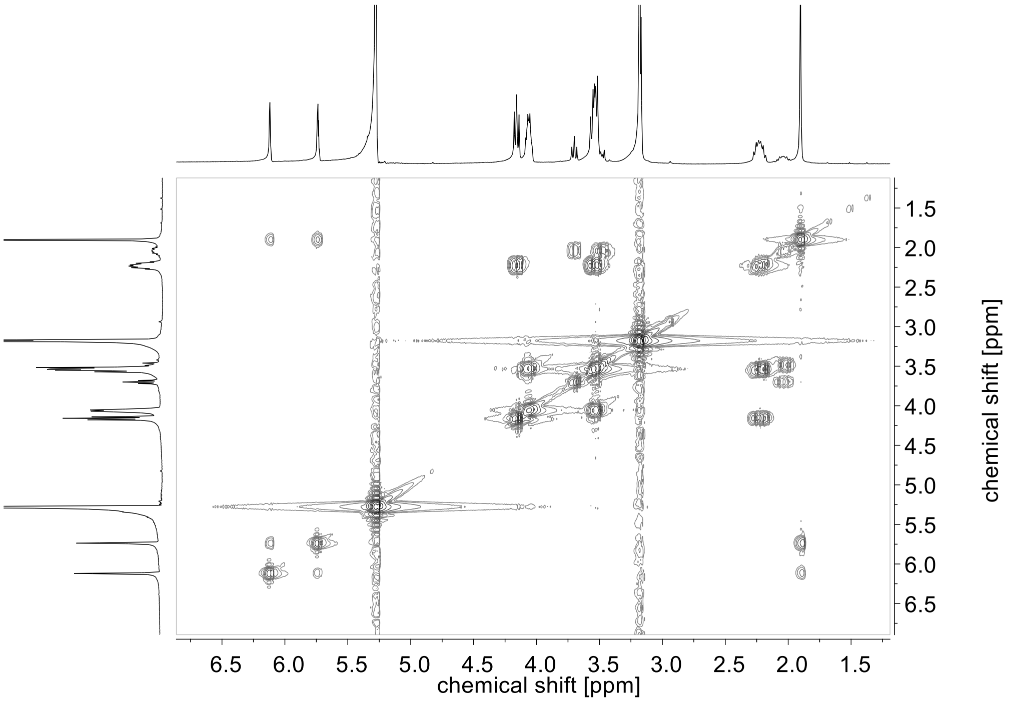


Figure S 44 $^{1}$H-$^{1}$H-COSY NMR spectra of 0.1 M solution of **M-2** in hydrochloric acid in $D_2O$, after 291 days.

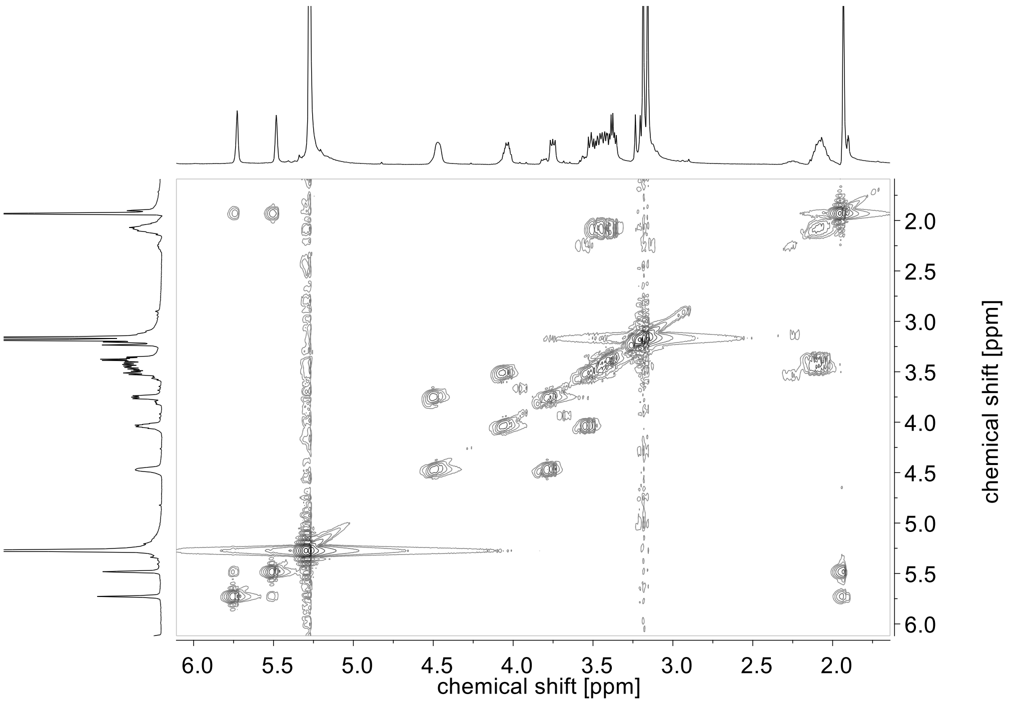


Figure S 45 $^{1}$H-$^{1}$H-COSY NMR spectra of 0.1 M solution of **M-3** in hydrochloric acid in $D_2O$, after 291 days.

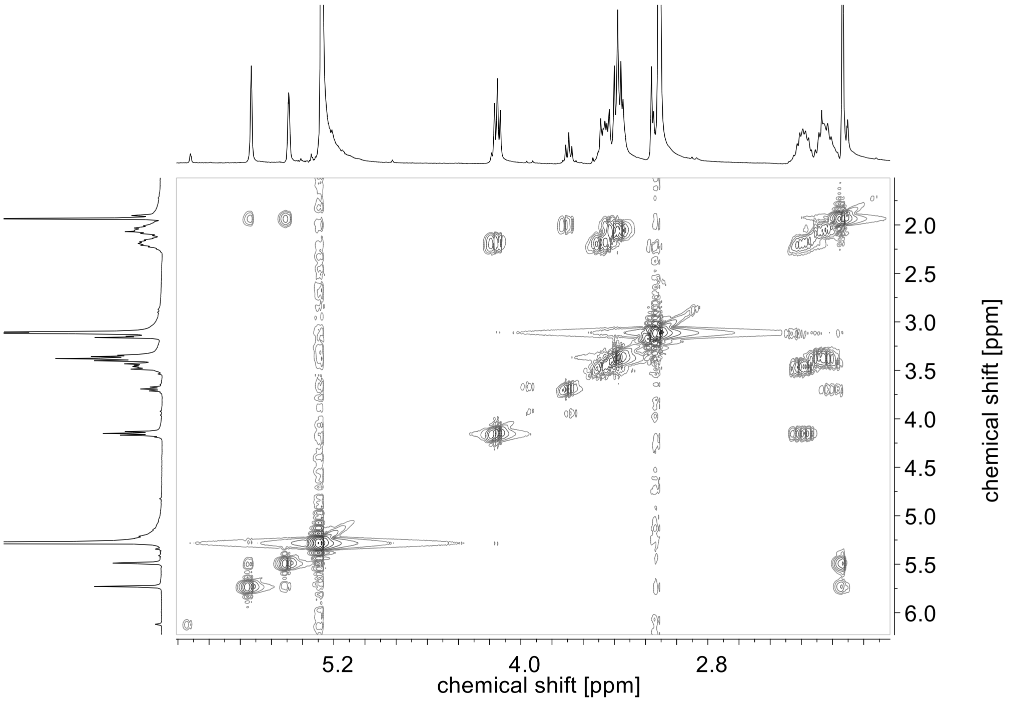


Figure S 46 $^1H$-$^1H$-COSY NMR spectra of 0.1 M solution of **M-4** in hydrochloric acid in $D_2O$, after 291 days.

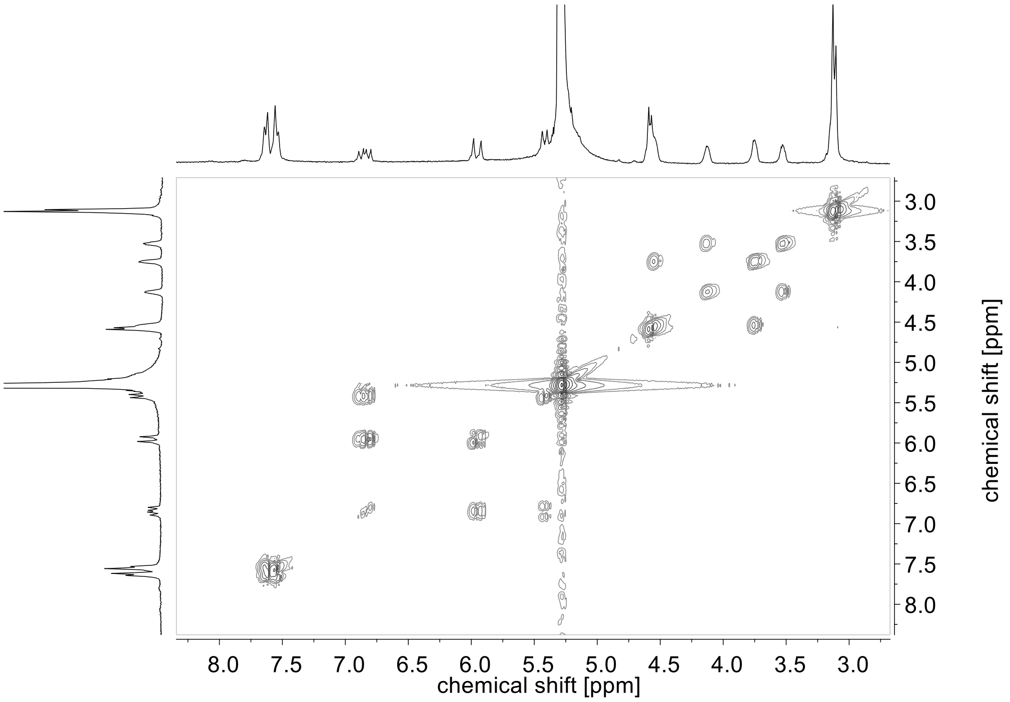


Figure S 47 $^1H$-$^1H$-COSY NMR spectra of 0.1 M solution of **M-5** in hydrochloric acid in $D_2O$, after 291 days.

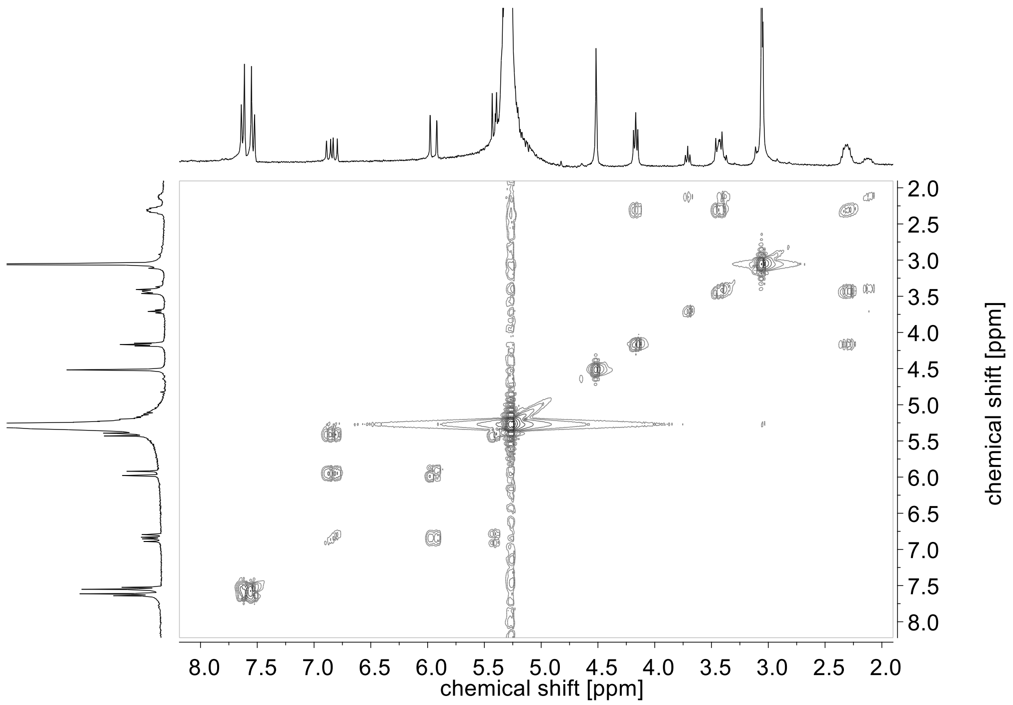


Figure S 48 $^{1}H$-$^{1}H$-COSY NMR spectra of 0.1 M solution of **M-6** in hydrochloric acid in $D_2O$, after 291 days.

## 4.3. Monomer hydrolysis hydrogen carbonate buffer (pH=10)

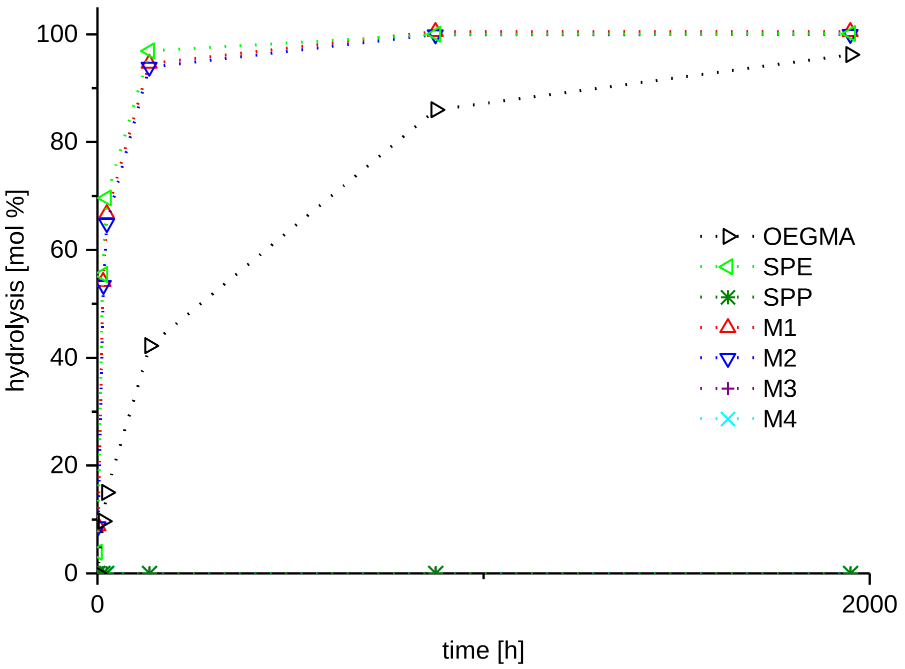


Figure S 49 Evolution of ester and amid hydrolysis of monomers in pH=10 carbonate buffer in $D_2O$: (□) = **OEGMA**, (□) = **SPE**, (*) = **SPP**, (□) = **M-1**, (□) = **M-2**, (+) = **M-3**, (X) = **M-4**.

Calculation of hydrolysis in mol %:

$$Hydrolyse_{\text{M-1}}\ [\text{mol }\%] = \left(\frac{I_{a_2'}*100}{I_{a'}+I_{a_2'}} + \frac{I_{a_2''}*100}{I_{a''}+I_{a_2''}}\right)/2$$

$$Hydrolyse_{M-2}\ [\text{mol }\%] = \left(\frac{I_{a_2'}*100}{I_{a'}+I_{a_2'}} + \frac{I_{a_2''}*100}{I_{a''}+I_{a_2''}}\right)/2$$

$$Hydrolyse_{M-3}\ [\text{mol }\%] = \left(\frac{I_{a_2'}*100}{I_{a'}+I_{a_2'}} + \frac{I_{a_2''}*100}{I_{a''}+I_{a_2''}}\right)/2$$

$$Hydrolyse_{M-4}\ [\text{mol }\%] = \left(\frac{I_{a_2'}*100}{I_{a'}+I_{a_2'}} + \frac{I_{a_2''}*100}{I_{a''}+I_{a_2''}}\right)/2$$

$$Hydrolyse_{OEGMA}\ [\text{mol }\%] = \left(\frac{I_{a_2'}*100}{I_{a'}+I_{a_2'}} + \frac{I_{a_2''}*100}{I_{a''}+I_{a_2''}}\right)/2$$

$$Hydrolyse_{SPE}\ [\text{mol }\%] = \left(\frac{I_{a_2'}*100}{I_{a'}+I_{a_2'}} + \frac{I_{a_2''}*100}{I_{a''}+I_{a_2''}}\right)/2$$

$$Hydrolyse_{SPP}\ [\text{mol }\%] = \left(\frac{I_{a_2'}*100}{I_{a'}+I_{a_2'}} + \frac{I_{a_2''}*100}{I_{a''}+I_{a_2''}}\right)/2$$

The Index 2 in e.g. $I_{e_2}$indicates the hydrolysis product of the ester/amid product, while no index e.g. $I_{a''}$ determines the unchanged molecule without hydrolysis.

$\mathrm{I}_{a'}(M\text{-}1,\ range\ in\ ppm)$ = 6.3-6.1
$\mathrm{I}_{a'_2}(M\text{-}1,\ range\ in\ ppm)$ = 5.7-5.6
$\mathrm{I}_{a''}(M\text{-}1,\ range\ in\ ppm)$ = 5.9-5.7
$\mathrm{I}_{a''_2}(M\text{-}1,\ range\ in\ ppm)$ = 5.4-5.3
$\mathrm{I}_{a'}(M\text{-}2,\ range\ in\ ppm)$ = 6.3-6.1
$\mathrm{I}_{a'_2}(M\text{-}2,\ range\ in\ ppm)$ = 5.7-5.6
$\mathrm{I}_{a''}(M\text{-}2,\ range\ in\ ppm)$ = 5.9-5.7
$\mathrm{I}_{a''_2}(M\text{-}2,\ range\ in\ ppm)$ = 5.4-5.3
$\mathrm{I}_{a'}(M\text{-}3,\ range\ in\ ppm)$ = 5.9-5.6
$\mathrm{I}_{a'_2}(M\text{-}3,\ range\ in\ ppm)$ = no signal
$\mathrm{I}_{a''}(M\text{-}3,\ range\ in\ ppm)$ = 5.6-5.4
$\mathrm{I}_{a''_2}(M\text{-}3,\ range\ in\ ppm)$ = no signal
$\mathrm{I}_{a'}(M\text{-}4,\ range\ in\ ppm)$ = 5.8-5.6
$\mathrm{I}_{a'_2}(M\text{-}4,\ range\ in\ ppm)$ = no signal
$\mathrm{I}_{a''}(M\text{-}4,\ range\ in\ ppm)$ = 5.6-5.4
$\mathrm{I}_{a''_2}(M\text{-}4,\ range\ in\ ppm)$ = no signal
$\mathrm{I}_{a'}(OEGMA,\ range\ in\ ppm)$ = 6.3-6.1
$\mathrm{I}_{a'_2}(OEGMA,\ range\ in\ ppm)$ = 5.7-5.6
$\mathrm{I}_{a''}(OEGMA,\ range\ in\ ppm)$ = 5.8-5.7
$\mathrm{I}_{a''_2}(OEGMA,\ range\ in\ ppm)$ = 5.4-5.3
$\mathrm{I}_{a'}(SPE,\ range\ in\ ppm)$ = 6.3-6.1
$\mathrm{I}_{a'_2}(SPE,\ range\ in\ ppm)$ = 5.7-5.6
$\mathrm{I}_{a''}(SPE,\ range\ in\ ppm)$ = 5.9-5.7
$\mathrm{I}_{a''_2}(SPE,\ range\ in\ ppm)$ = 5.5-5.3
$\mathrm{I}_{a'}(SPP,\ range\ in\ ppm)$ = 5.9-5.6
$\mathrm{I}_{a'_2}(SPP,\ range\ in\ ppm)$ = no signal
$\mathrm{I}_{a''}(SPP,\ range\ in\ ppm)$ = 5.6-5.4
$\mathrm{I}_{a''_2}(SPP,\ range\ in\ ppm)$ = no signal

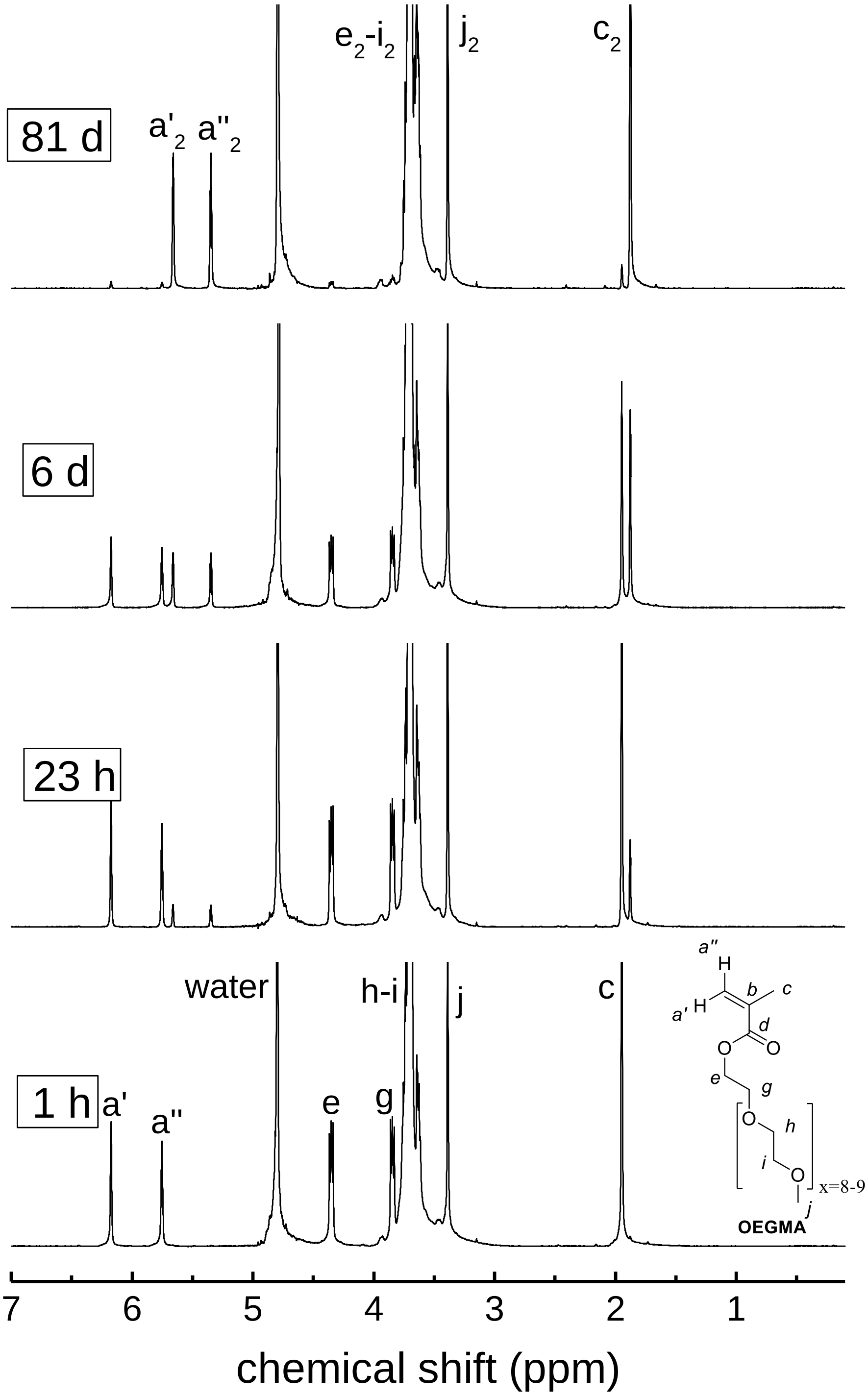


Figure S 50 $^{1}$H-NMR spectrum showing the degradation of 0.1 M solution of **OEGMA** in carbonate buffer in $D_2O$ (pH = 10) at room temperature over time.

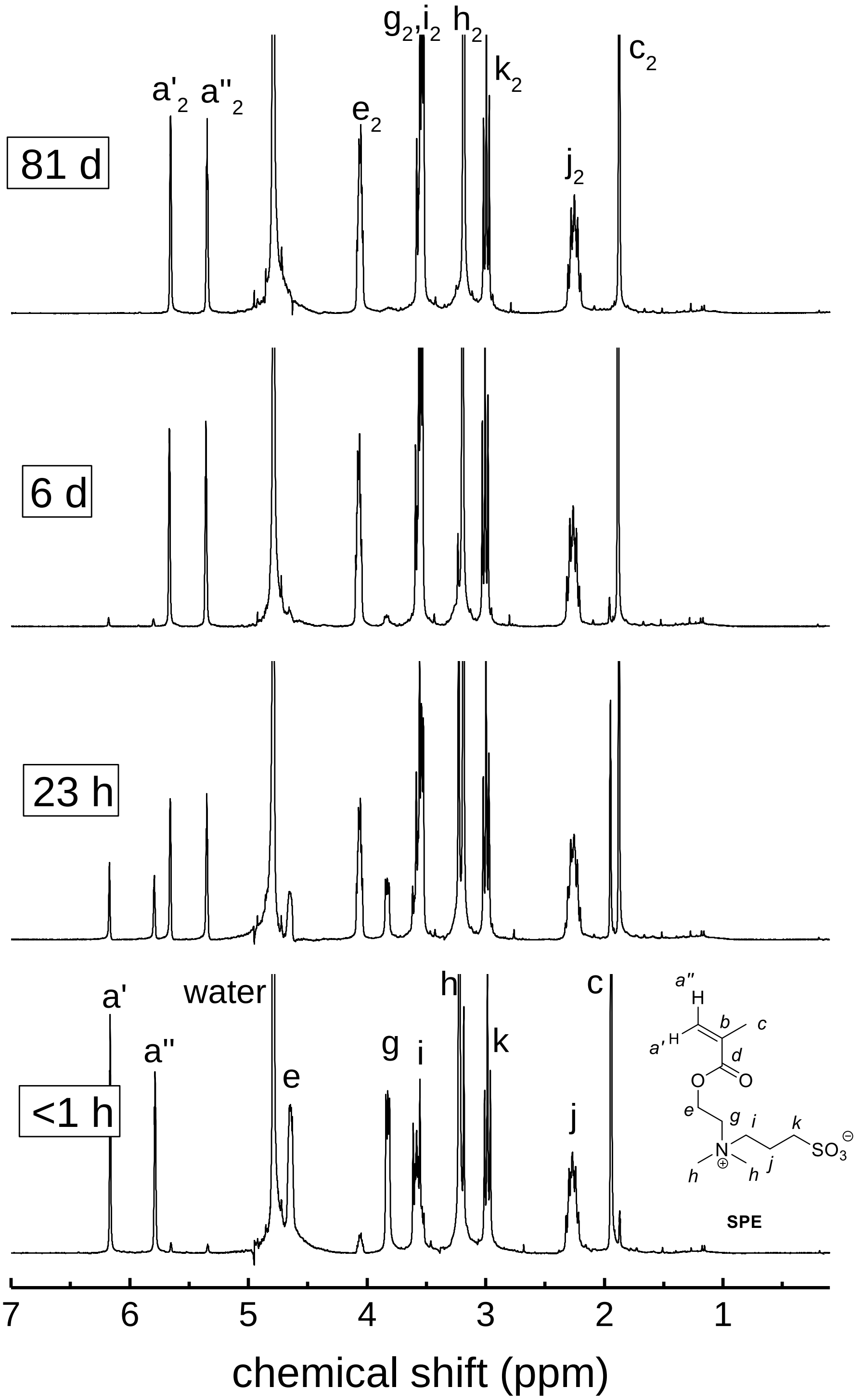


Figure S 51 $^{1}$H-NMR spectrum showing the degradation of 0.1 M solution of **SPE** in carbonate buffer in $D_2O$ (pH = 10) at room temperature over time.

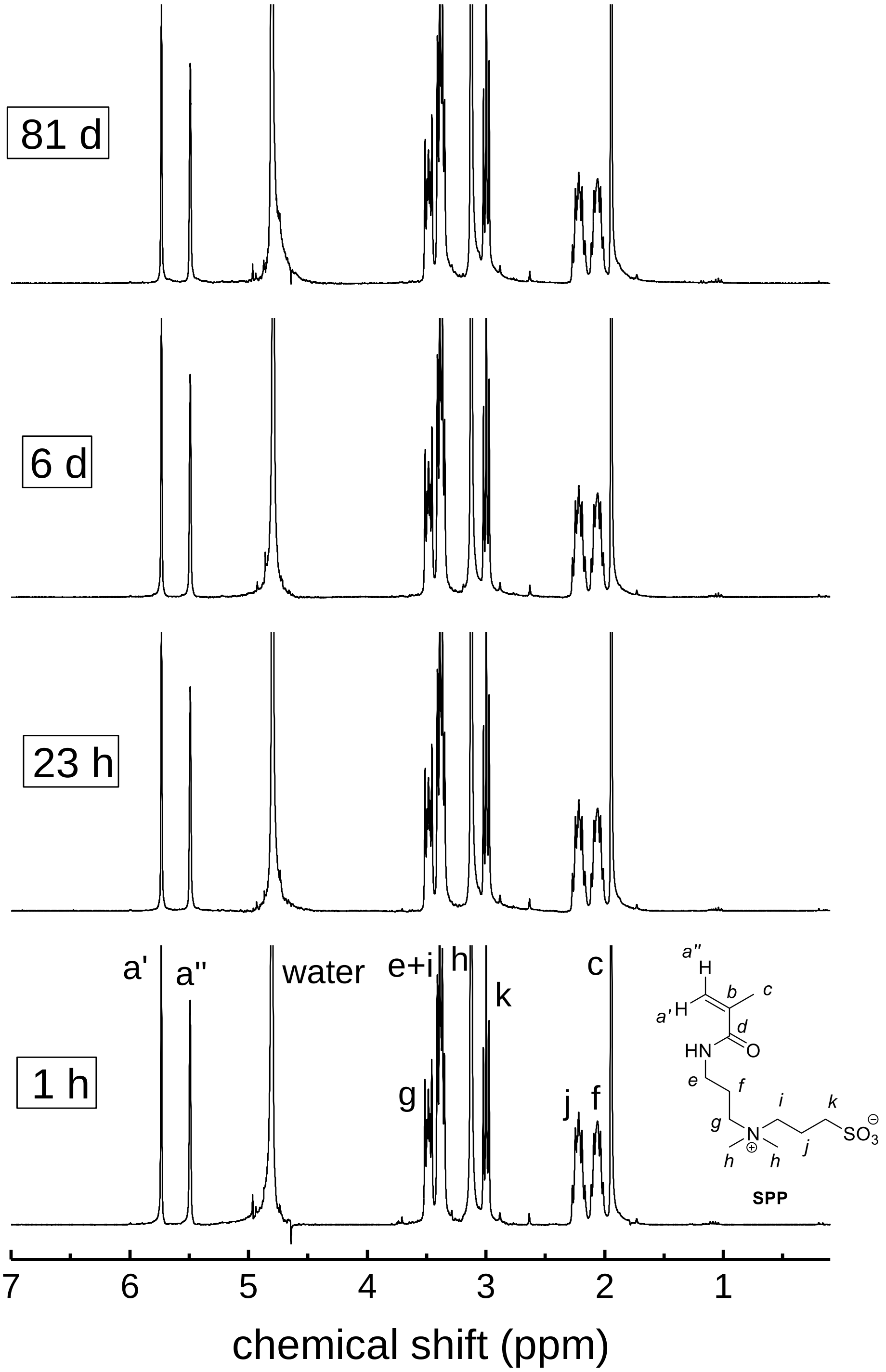


Figure S 52 $^{1}$H-NMR spectrum showing the degradation of 0.1 M solution of **SPP** in carbonate buffer in $D_2O$ (pH = 10) at room temperature over time.

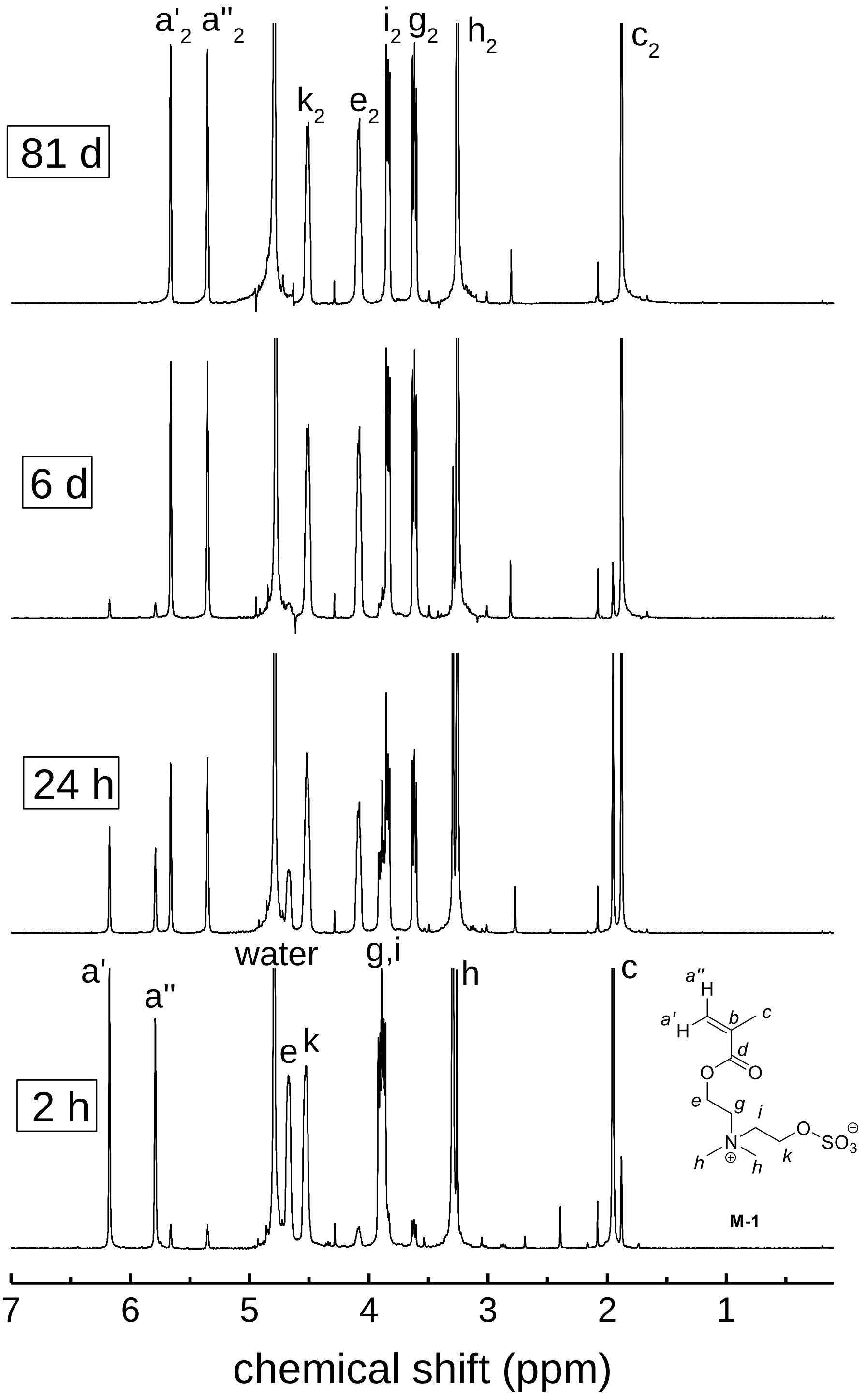


Figure S 53 $^{1}$H-NMR spectrum showing the degradation of 0.1 M solution of **M-1** in carbonate buffer in $D_2O$ (pH = 10) at room temperature over time.

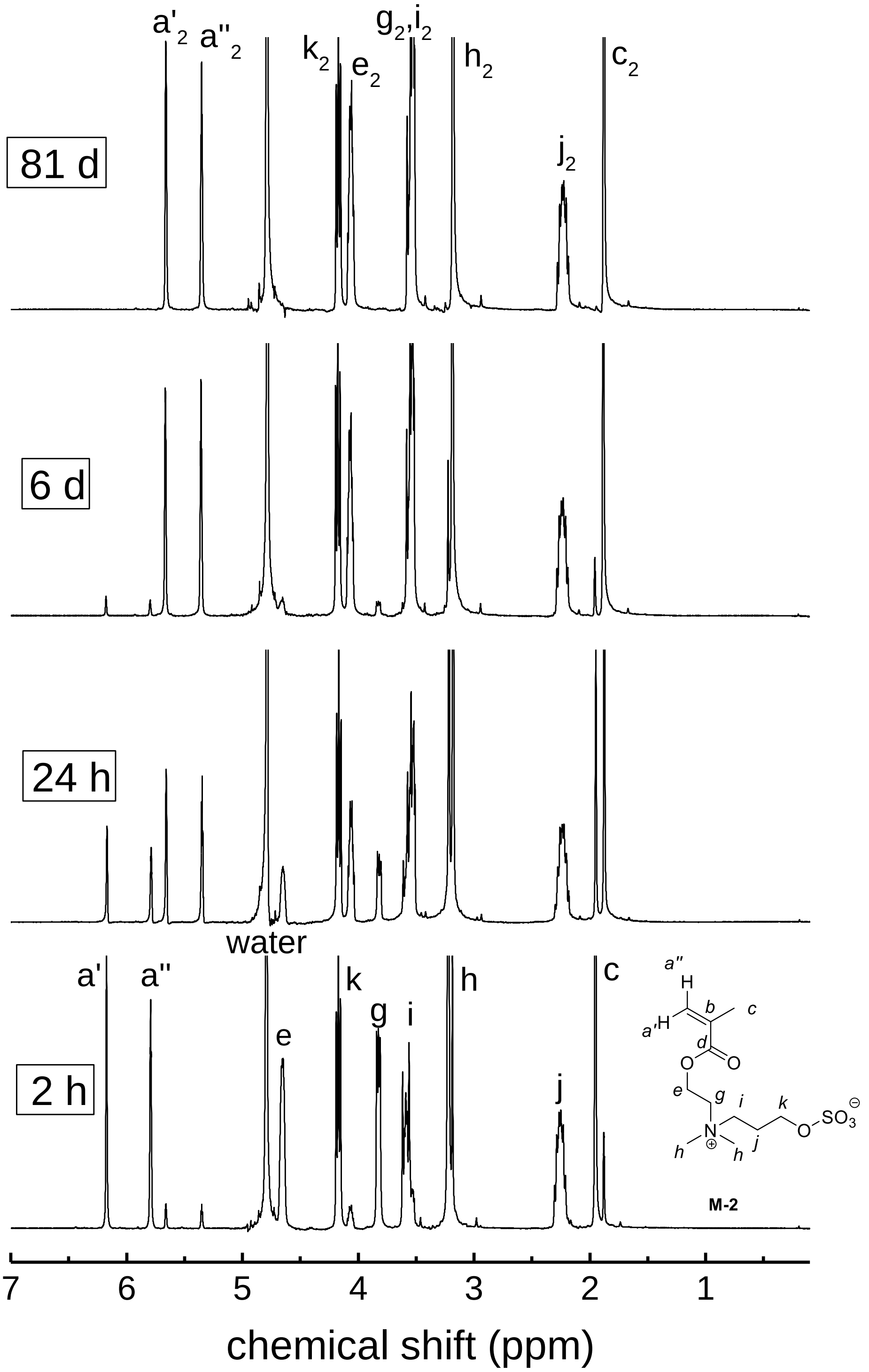


Figure S 54 $^{1}$H-NMR spectrum showing the degradation of 0.1 M solution of **M-2** in carbonate buffer in $D_2O$ (pH = 10) at room temperature over time.

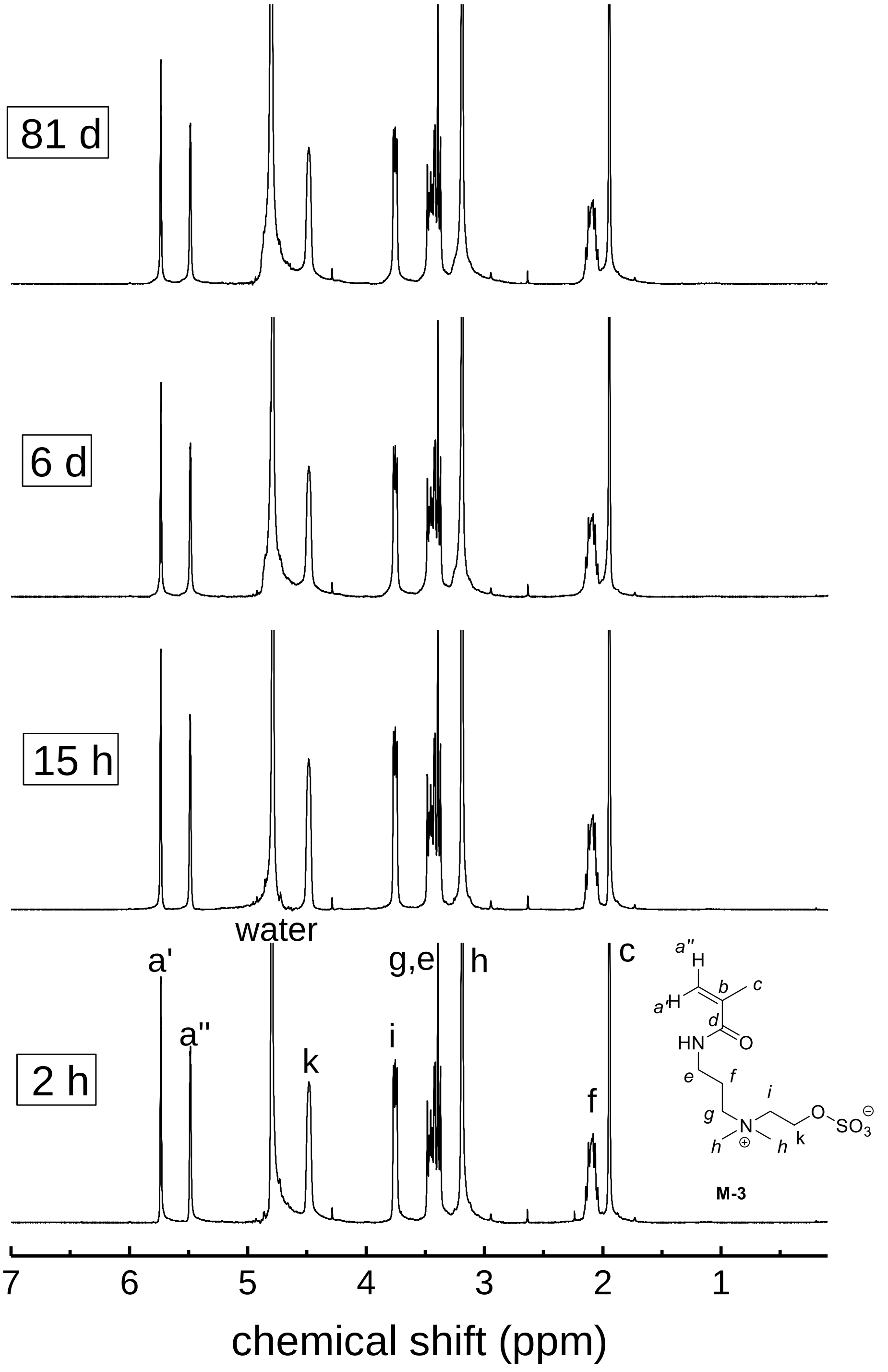


Figure S 55 $^{1}$H-NMR spectrum showing the degradation of 0.1 M solution of **M-3** in carbonate buffer in $D_2O$ (pH = 10) at room temperature over time.

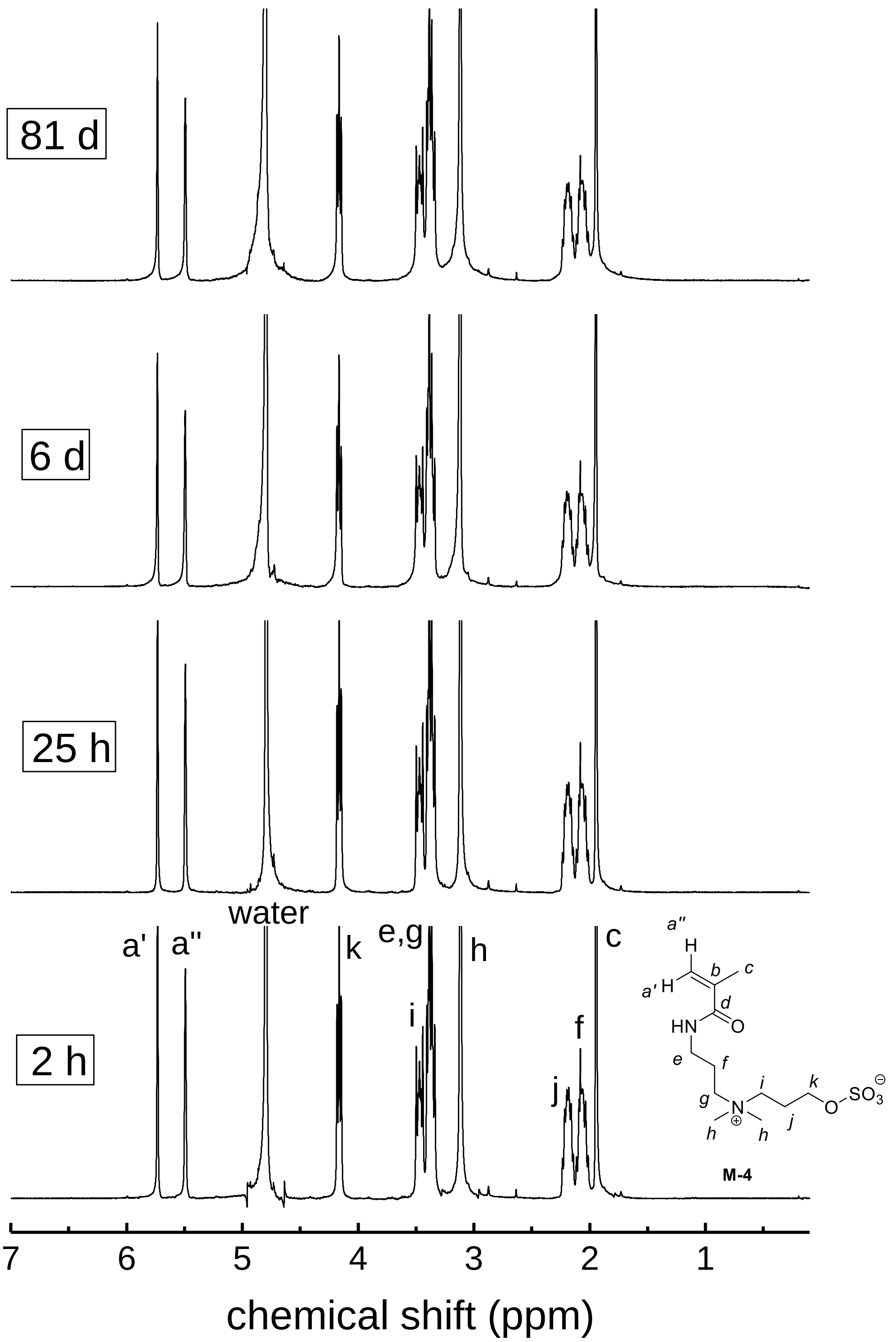


Figure S 56 $^{1}$H-NMR spectrum showing the degradation of 0.1 M solution of **M-4** in carbonate buffer in $D_2O$ (pH = 10) at room temperature over time.

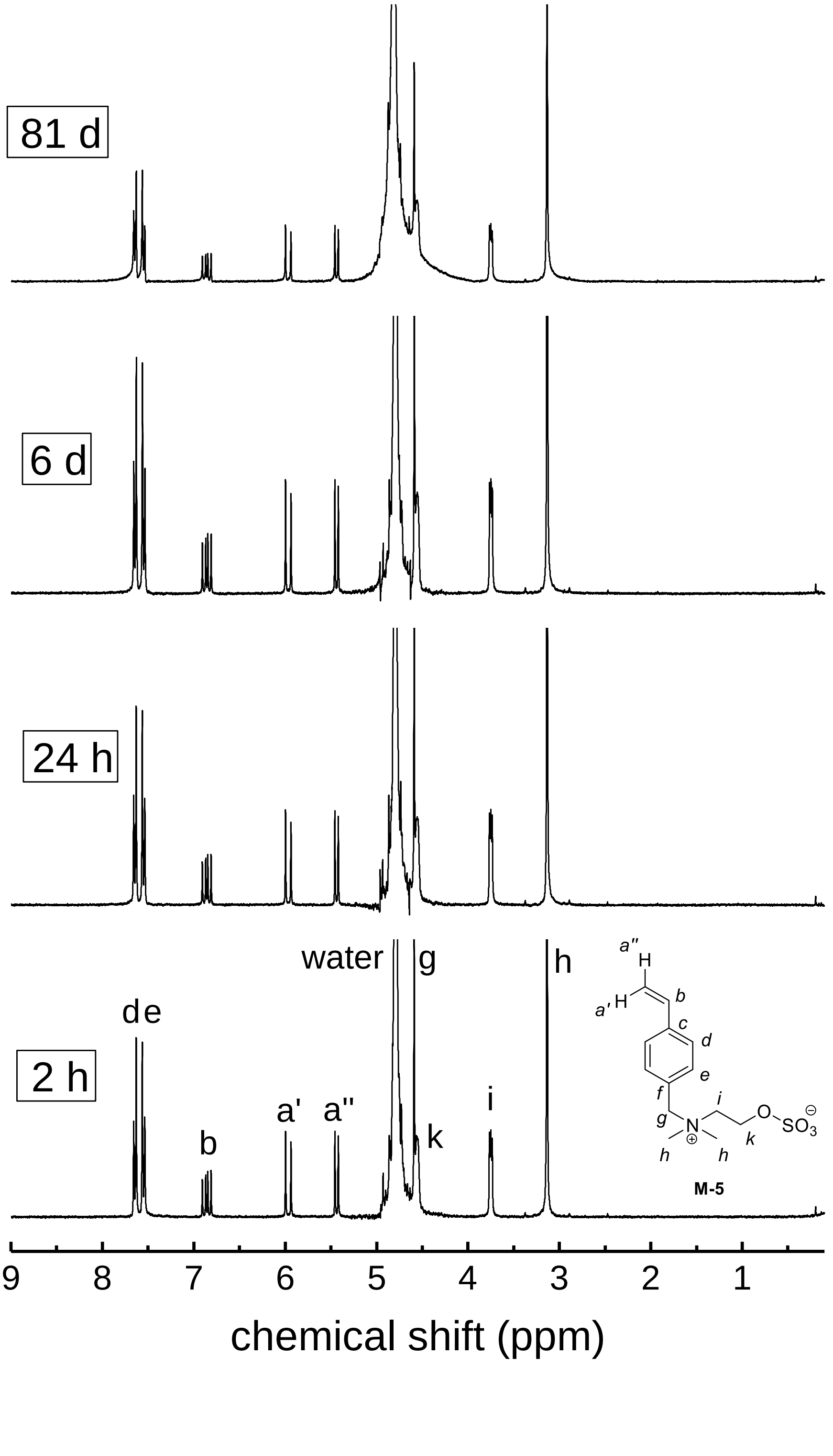


Figure S 57 $^{1}$H-NMR spectrum showing the degradation of 0.1 M solution of **M-5** in carbonate buffer in $D_2O$ (pH = 10) at room temperature over time.

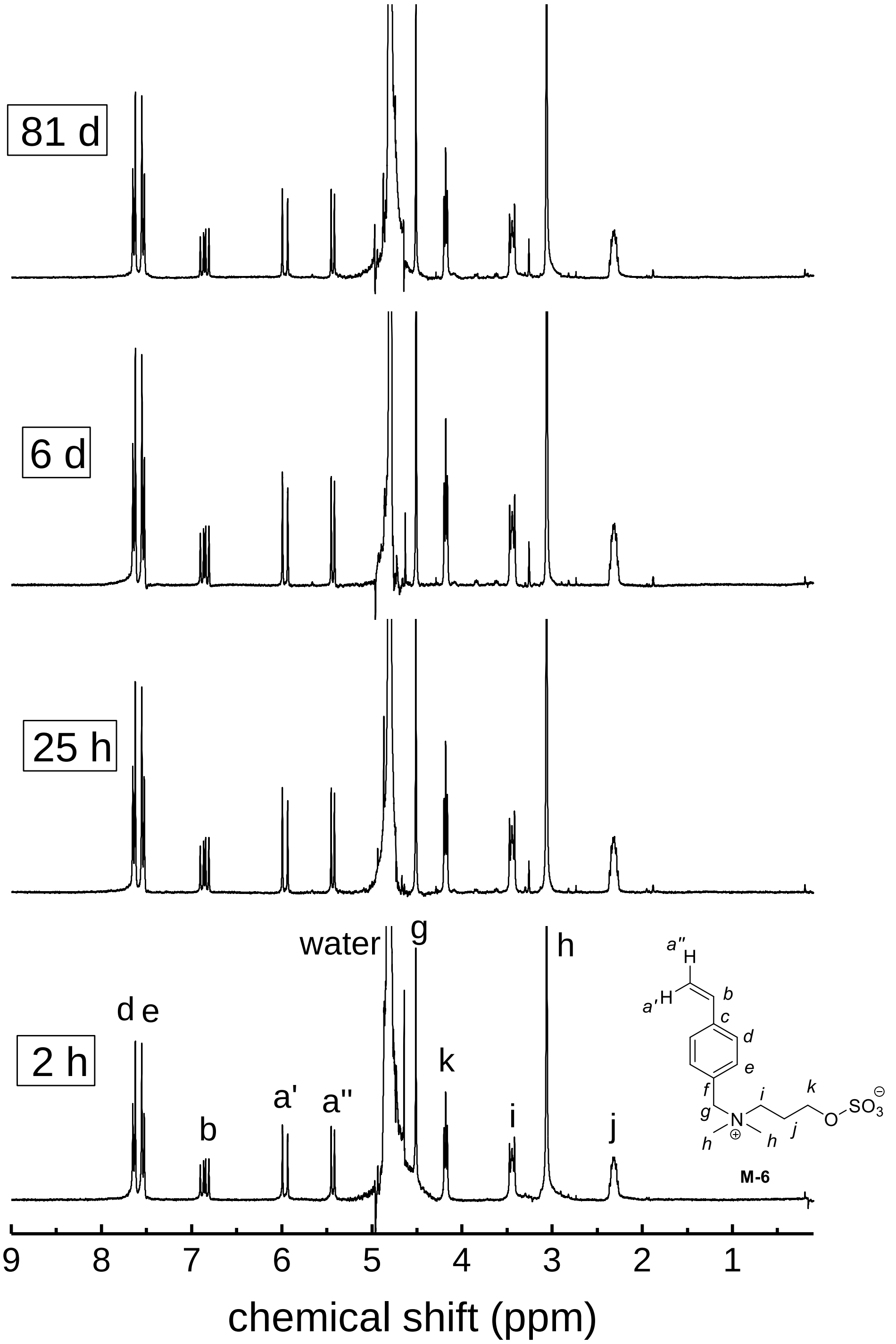


Figure S 58 $^{1}$H-NMR spectrum showing the degradation of 0.1 M solution of **M-6** in carbonate buffer in $D_2O$ (pH = 10) at room temperature over time.

4.4. Monomer hydrolysis in 1 M sodium hydroxide solution (pH=14)

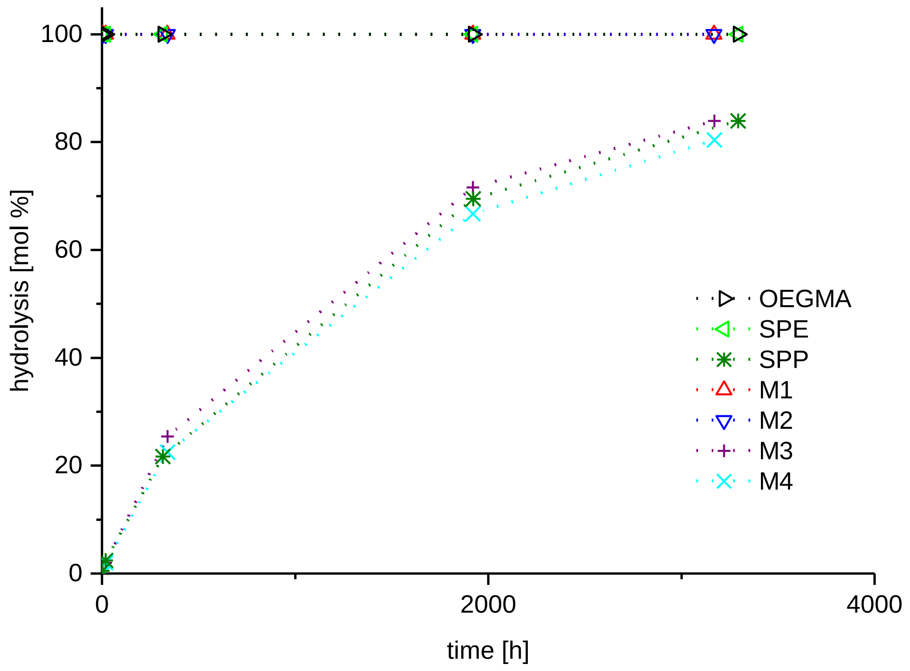


Figure S 59 Evolution of ester and amid hydrolysis of monomers in sodium hydroxide in $D_2O$ (pH=14): (□) = **OEGMA**, (□) = **SPE**, (*) = **SPP**, (□) = **M-1**, (□) = **M-2**, (+) = **M-3**, (X) = **M-4**.

Calculation of hydrolysis in mol %:

$$Hydrolyse_{\text{M-1}}\ [\text{mol }\%] = \left(\frac{I_{a'_2}*100}{I_{a'}+I_{a'_2}} + \frac{I_{a''_2}*100}{I_{a''}+I_{a''_2}}\right)/2$$

$$Hydrolyse_{\text{M-2}}\ [\text{mol }\%] = \left(\frac{I_{a'_2}*100}{I_{a'}+I_{a'_2}} + \frac{I_{a''_2}*100}{I_{a''}+I_{a''_2}}\right)/2$$

$$Hydrolyse_{\text{M-3}}\ [\text{mol }\%] = \left(\frac{I_{a''_2}*100}{I_{a''}+I_{a''_2}}\right)$$

$$Hydrolyse_{\text{M-4}}\ [\text{mol }\%] = \left(\frac{I_{a''_2}*100}{I_{a''}+I_{a''_2}}\right)$$

$$Hydrolyse_{\text{OEGMA}}\ [\text{mol }\%] = \left(\frac{I_{a'}*100}{I_{a'}+I_{a'_2}} + \frac{I_{a''}*100}{I_{a''}+I_{a''_2}}\right)/2$$

$$Hydrolyse_{SPE}\ [\text{mol }\%] = \left(\frac{I_{a'}*100}{I_{a'}+I_{a'_2}} + \frac{I_{a''}*100}{I_{a''}+I_{a''_2}}\right)/2$$

$$Hydrolyse_{SPP}\ [\text{mol }\%] = \left(\frac{I_{a'_2}*100}{I_{a'}+I_{a'_2}} + \frac{I_{a''_2}*100}{I_{a''}+I_{a''_2}}\right)/2$$

The Index 2 in e.g. $I_{e_2}$ indicates the hydrolysis product of the ester/amid product, while no index e.g. $I_{a''}$ determines the unchanged molecule without hydrolysis.

$I_{a'}(M\text{-}1,\ range\ in\ ppm)$ = no signal
$I_{a_2'}(M\text{-}1,\ range\ in\ ppm)$ = 5.8-5.6
$I_{a''}(M\text{-}1,\ range\ in\ ppm)$ = no signal
$I_{a_2''}(M\text{-}1,\ range\ in\ ppm)$ = 5.5-5.3
$I_{a'}(M\text{-}2,\ range\ in\ ppm)$ = no signal
$I_{a_2'}(M\text{-}2,\ range\ in\ ppm)$ = 5.7-5.6
$I_{a''}(M\text{-}2,\ range\ in\ ppm)$ = no signal
$I_{a_2''}(M\text{-}2,\ range\ in\ ppm)$ = 5.4-5.3
$I_{a''}(M\text{-}3,\ range\ in\ ppm)$ = 5.6-5.4
$I_{a_2''}(M\text{-}3,\ range\ in\ ppm)$ = 5.4-5.3
$I_{a''}(M\text{-}4,\ range\ in\ ppm)$ = 5.4-5.3
$I_{a_2''}(M\text{-}4,\ range\ in\ ppm)$ = 5.3-5.2
$I_{a'}(OEGMA,\ range\ in\ ppm)$ = no signal
$I_{a_2'}(OEGMA,\ range\ in\ ppm)$ = 5.8-5.6
$I_{a''}(OEGMA,\ range\ in\ ppm)$ = no signal
$I_{a_2''}(OEGMA,\ range\ in\ ppm)$ = 5.4-5.3
$I_{a'}(SPE,\ range\ in\ ppm)$ = no signal
$I_{a_2'}(SPE,\ range\ in\ ppm)$ = 5.8-5.6
$I_{a''}(SPE,\ range\ in\ ppm)$ = no signal
$I_{a_2''}(SPE,\ range\ in\ ppm)$ = 5.4-5.3
$I_{a'}(SPP,\ range\ in\ ppm)$ = 5.8-5.7
$I_{a_2'}(SPP,\ range\ in\ ppm)$ = 5.7-5.6
$I_{a''}(SPP,\ range\ in\ ppm)$ = 5.5-5.4
$I_{a_2''}(SPP,\ range\ in\ ppm)$ = 5.4-5.3

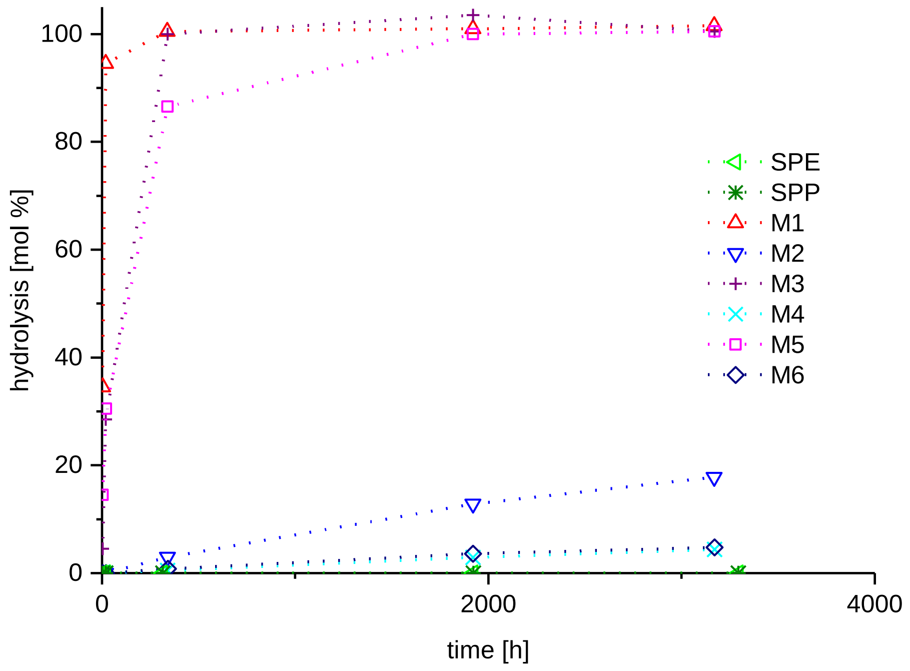


Figure S 60 Evolution of sulfate and sulfonate hydrolysis of monomers in sodium hydroxide in $D_2O$ (pH=14): (□) = **SPE**, (*) = **SPP**, (□) = **M-1**, (□) = **M-2**, (+) = **M-3**, (X) = **M-4**, (□) = **M-5**, (□) = **M-6**.

<u>Calculation of hydrolysis in mol %:</u>

General (without use of product integral):

$$Hyd._{\text{M-X}}\ [\text{mol\%}] = \left(\frac{I_{k_3}}{I_{k_3} + I_{k_2}}\right) * 100 = \frac{2 * I_{a_2''} - I_{k_2}}{2 * I_{a_2''} - I_{k_2} + I_{k_2}} * 100 = \frac{2 * I_{a_2''} - I_{k_2}}{2 * I_{a_2''}} * 100$$

$$Hydrolyse_{\text{M-1}}\ [\text{mol \%}] = \left(\frac{2 * I_{a_2''} - I_{k_2}}{2 * I_{a_2''}}\right) * 100)$$

$$Hydrolyse_{M-2}\ [\text{mol \%}] = \left(\frac{2 * I_{a_2''} - I_{k_2}}{2 * I_{a_2''}}\right) * 100)$$

$$Hydrolyse_{M-3}\ [\text{mol \%}] = \left(\frac{2 * I_{a_2''+a''} - I_{k_2}}{2 * I_{a_2''a''}}\right) * 100)$$

$$Hydrolyse_{M-4}\ [\text{mol \%}] = \left(\frac{2 * I_{a_2''+a''} - I_{k_2}}{2 * I_{a_2''+a''}}\right) * 100)$$

$$Hydrolyse_{M-5}\ [\text{mol \%}] = \left(\frac{2 * I_{a_2'} - I_{i_2}}{2 * I_{a_2'}}\right) * 100)$$

$$Hydrolyse_{M-6}\ [\text{mol \%}] = \left(\frac{2 * I_{a_2'} - I_{k_2}}{2 * I_{a_2'}}\right) * 100)$$

$$Hydrolyse_{SPE}\ [\text{mol \%}] = \left(\frac{2 * I_{a_2''} - I_{k_2}}{2 * I_{a_2''}}\right) * 100$$

$$Hydrolyse_{SPP}\ [\text{mol}\ \%] = (\frac{2 * I_{a_2''} - I_{k_2}}{2 * I_{a_2''}}) * 100$$

The Index 2 in e.g. $I_{e_2}$indicates the hydrolysis product of the ester/amid product, while no index e.g. $I_{a''}$ determines the unchanged molecule without hydrolysis. Index 3 in e.g. $I_{e_3}$indicates the sulfate hydrolysis product.

$I_{k_2}(M\text{-}1,\ range\ in\ ppm)$ = 4.5-4.35
$I_{I_{a_2''}}(M\text{-}1,\ range\ in\ ppm)$ = 5.4-5.1
$I_{k_2}(M\text{-}2,\ range\ in\ ppm)$ = 4.3-4.1
$I_{I_{a_2''}}(M\text{-}2,\ range\ in\ ppm)$ = 5.4-5.3
$I_{k_2}(M\text{-}3,\ range\ in\ ppm)$ = 4.6-4.4
$I_{I_{a_2''}}(M\text{-}3,\ range\ in\ ppm)$ = 5.6-5.4
$I_{k_2}(M\text{-}4,\ range\ in\ ppm)$ = 4.3-4.0
$I_{I_{a_2''}}(M\text{-}4,\ range\ in\ ppm)$ = 5.6-5.3
$I_{i_2}(M\text{-}5,\ range\ in\ ppm)$ = 3.9-3.6
$I_{I_{a_2''}}(M\text{-}5,\ range\ in\ ppm)$ = 6.1-5.8
$I_{I_{a_2''}}(M\text{-}6,\ range\ in\ ppm)$ = 6.1-5.8
$I_{k_2}(M\text{-}6,\ range\ in\ ppm)$ = no decomposition
$I_{I_{a_2''}}(SPE,\ range\ in\ ppm)$ = 5.4-5.3
$I_{k_2}(SPE,\ range\ in\ ppm)$ = no decomposition
$I_{I_{a_2''}}(SPP,\ range\ in\ ppm)$ = 5.5-5.3
$I_{k_2}(SPP,\ range\ in\ ppm)$ = no decomposition

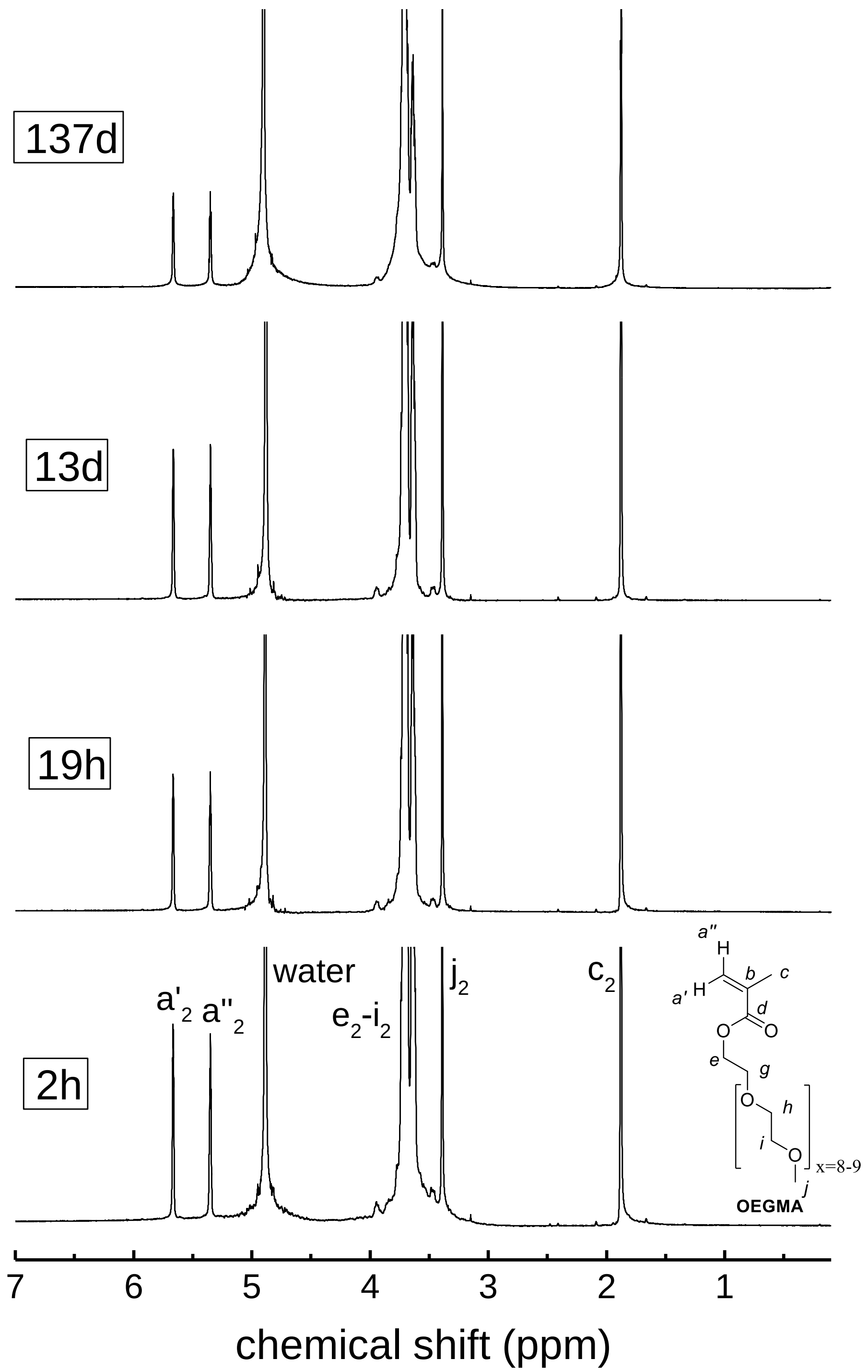


Figure S 61 $^{1}$H-NMR spectrum showing the degradation of 0.1 M solution of **OEGMA** in sodium hydroxid in $D_2O$ (pH = 14) at room temperature over time.

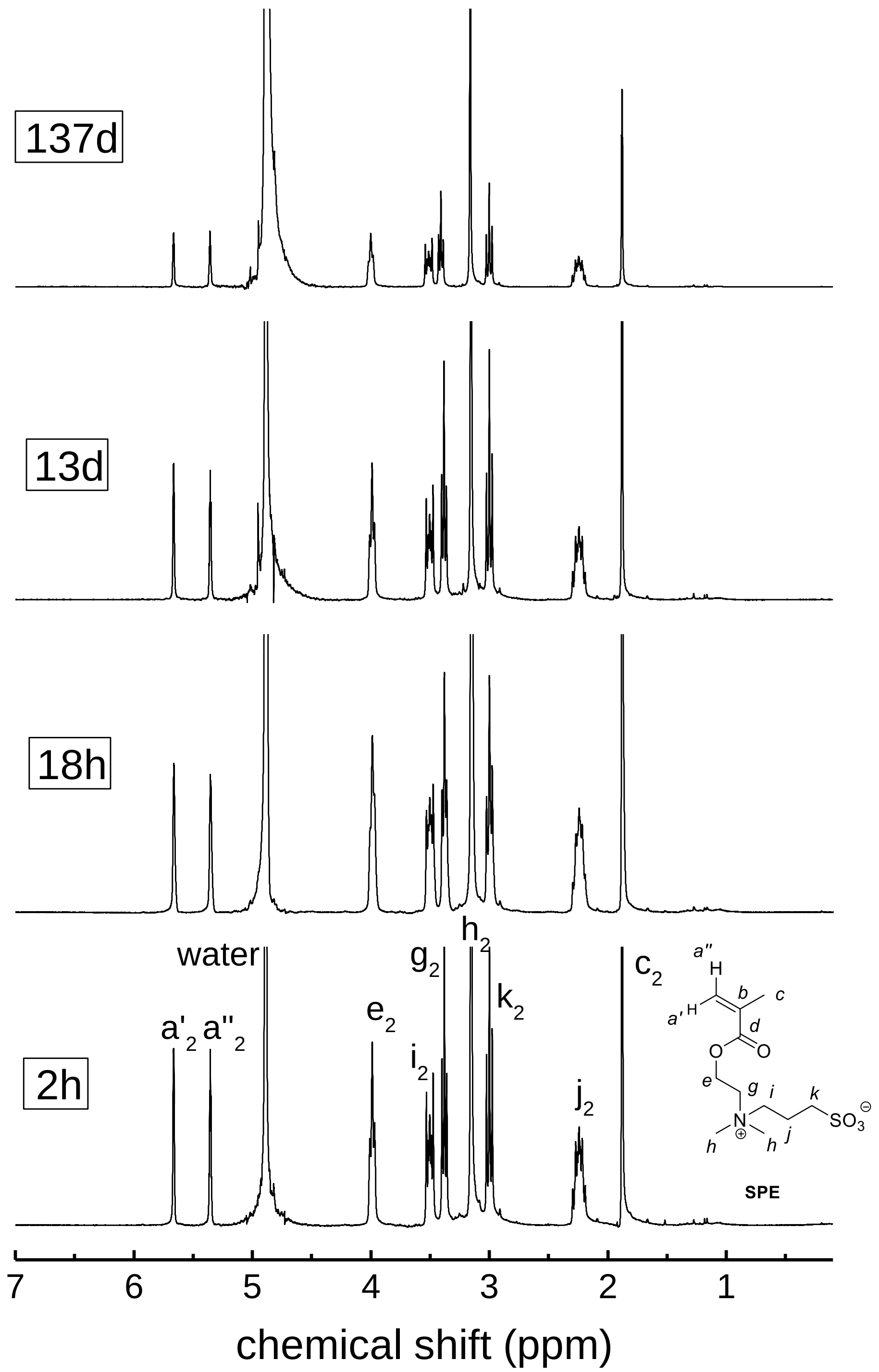


Figure S 62 $^{1}$H-NMR spectrum showing the degradation of 0.1 M solution of **SPE** in sodium hydroxid in $D_2O$ (pH = 14) at room temperature over time.

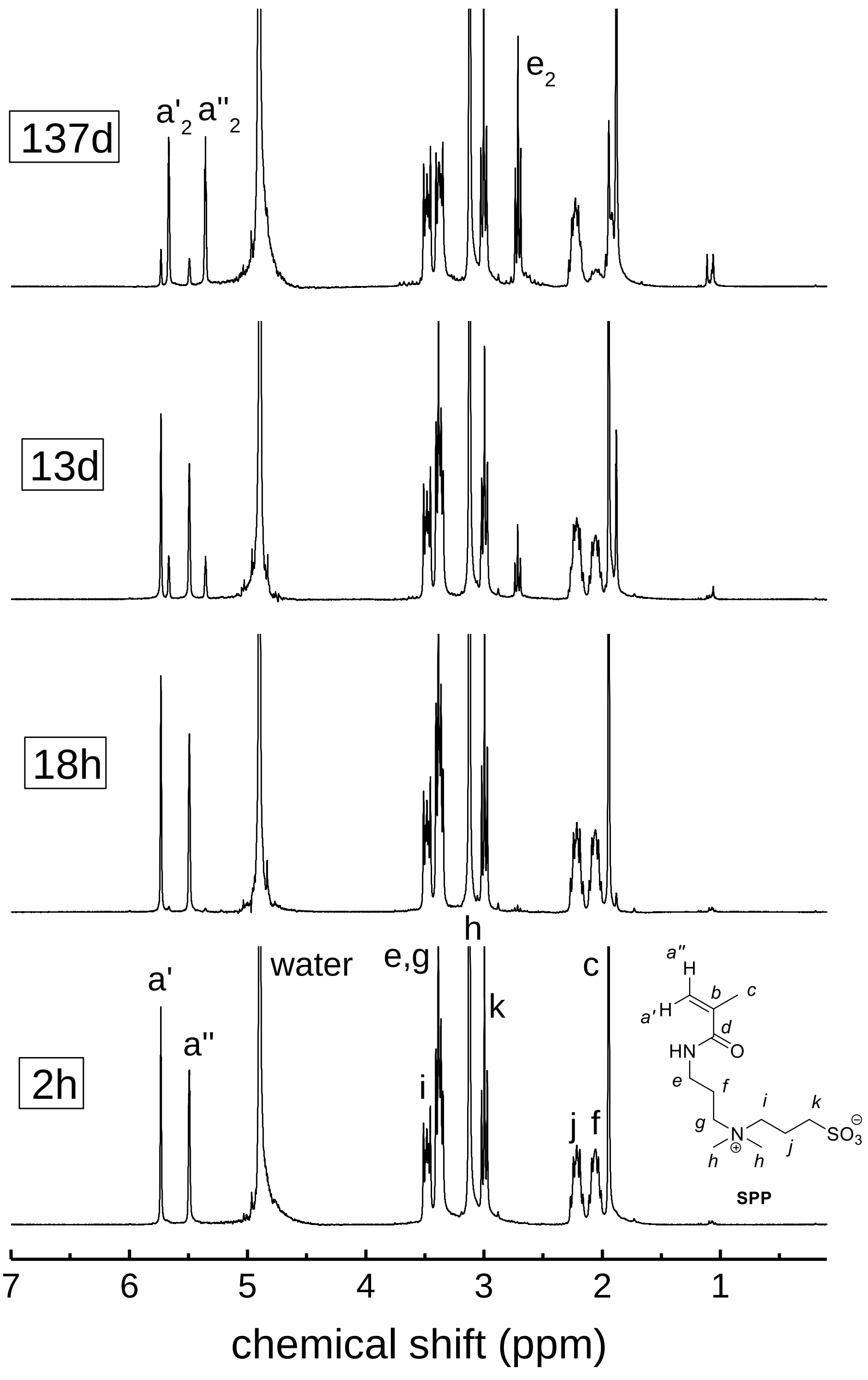


Figure S 63 $^{1}$H-NMR spectrum showing the degradation of 0.1 M solution of **SPP** in sodium hydroxid in $D_2O$ (pH = 14) at room temperature over time.

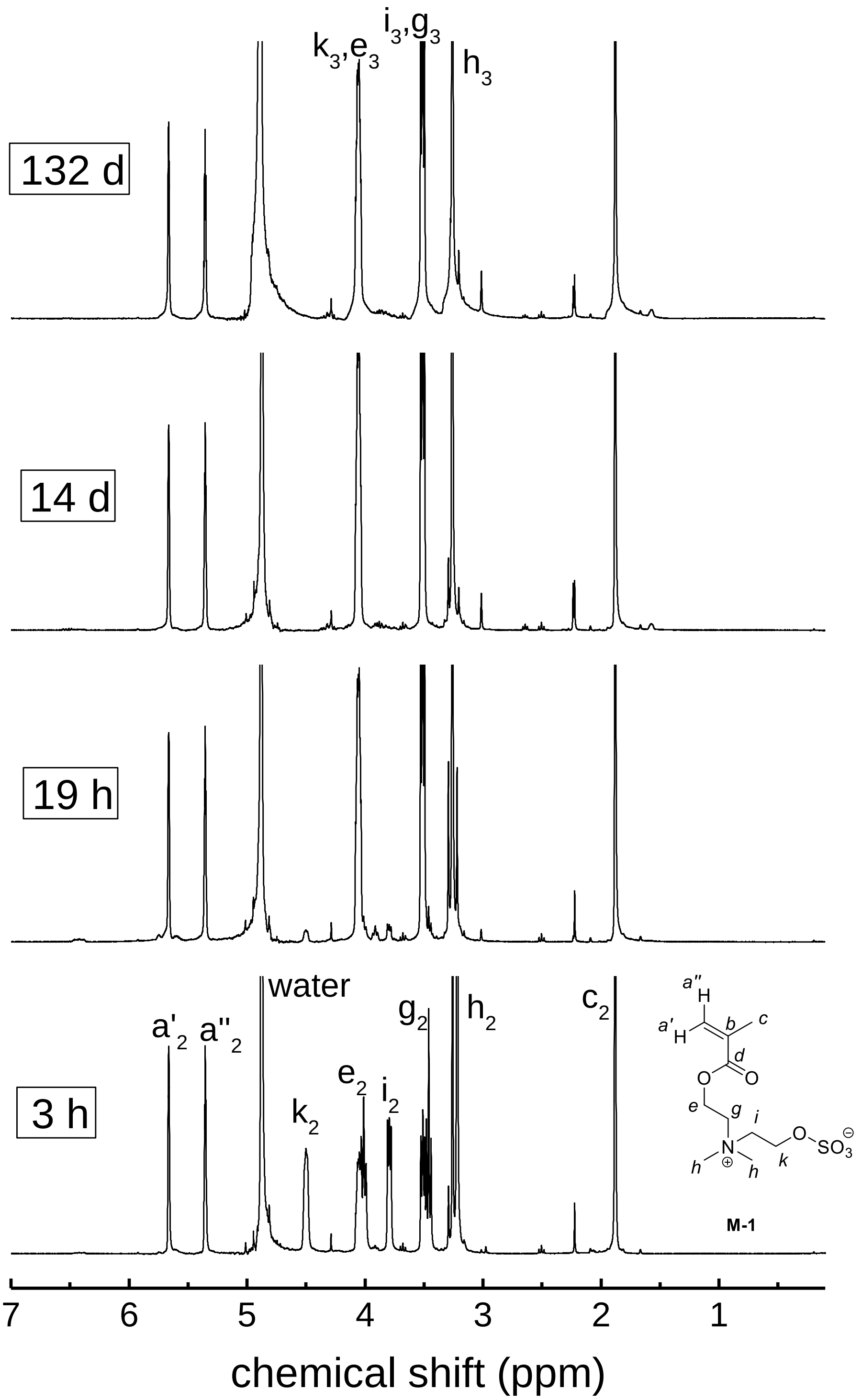


Figure S 64 $^{1}$H-NMR spectrum showing the degradation of 0.1 M solution of **M-1** in sodium hydroxid in $D_2O$ (pH = 14) at room temperature over time.

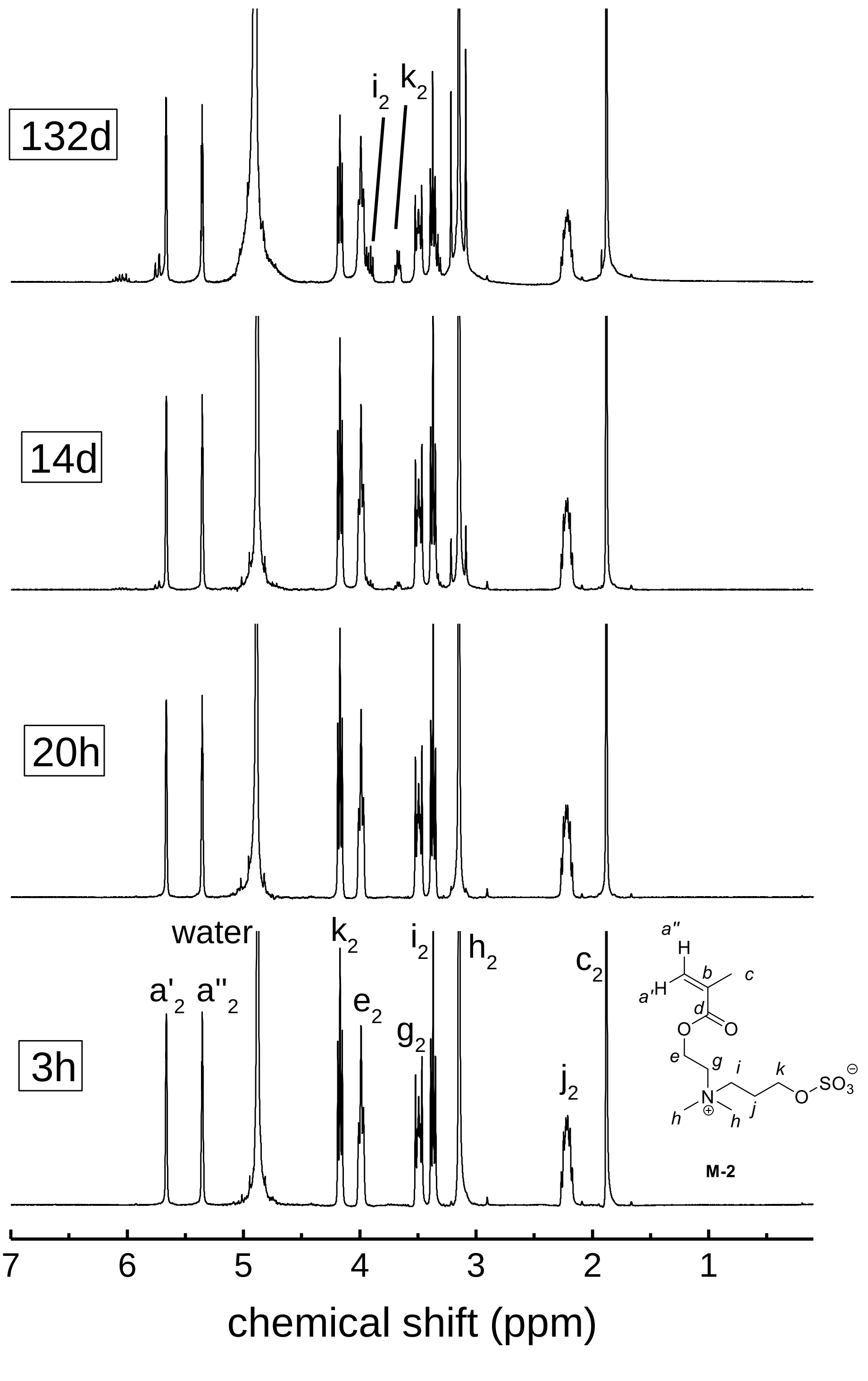


Figure S 65 $^{1}$H-NMR spectrum showing the degradation of 0.1 M solution of **M-2** in sodium hydroxid in $D_2O$ (pH = 14) at room temperature over time.

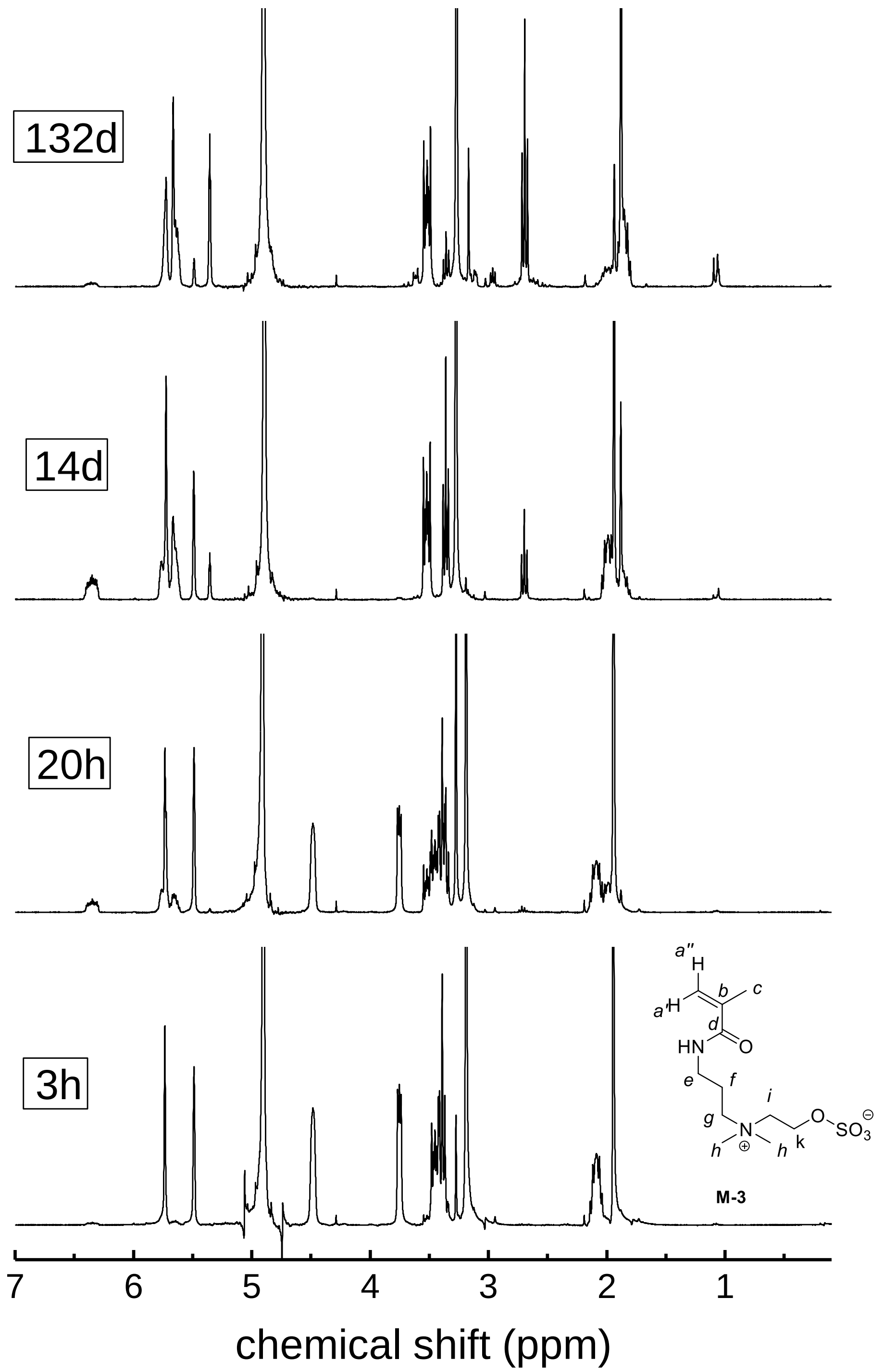


Figure S 66 $^{1}$H-NMR spectrum showing the degradation of 0.1 M solution of **M-3** in sodium hydroxid in $D_2O$ (pH = 14) at room temperature over time.

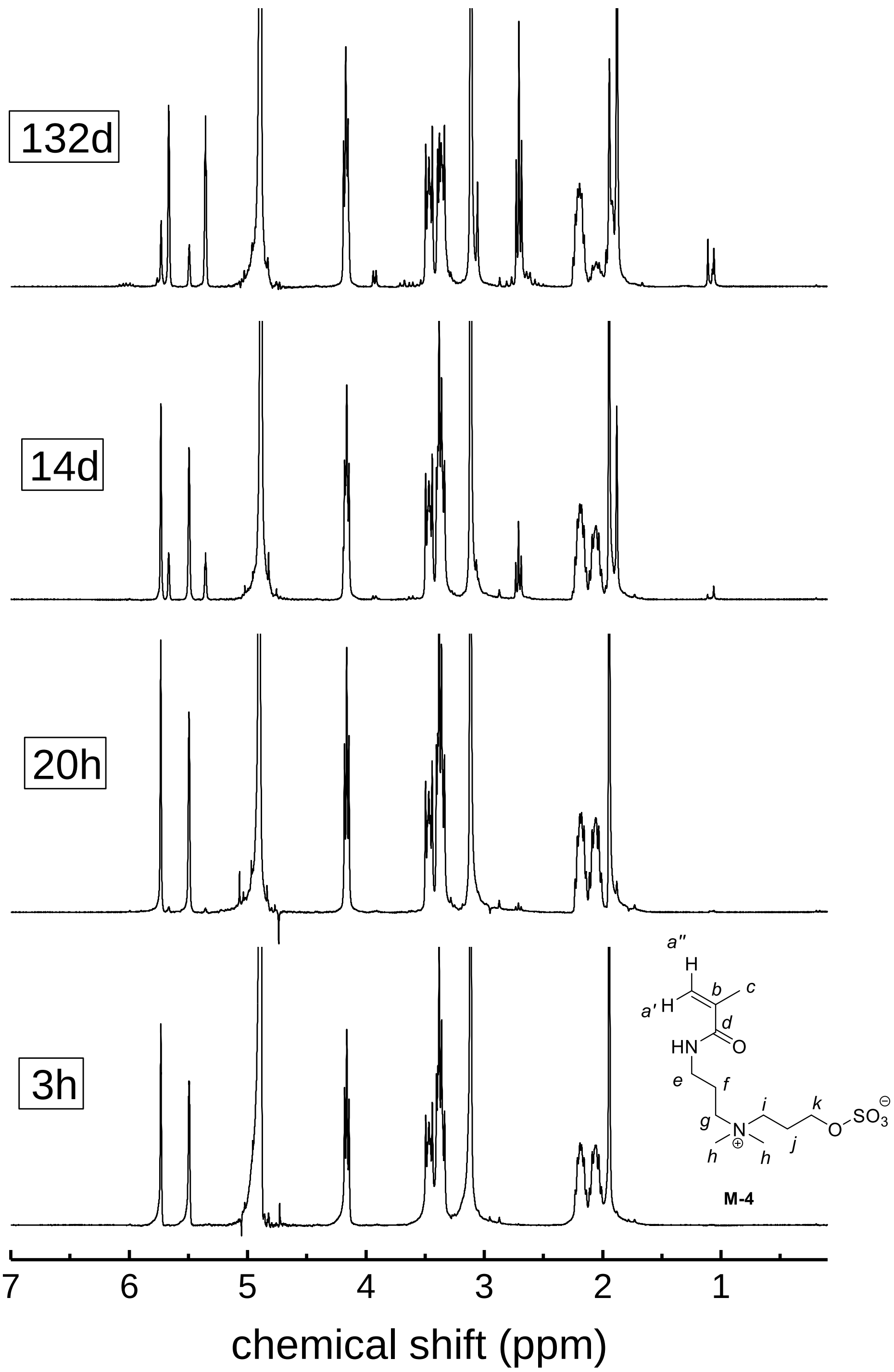


Figure S 67 $^{1}$H-NMR spectrum showing the degradation of 0.1 M solution of **M-4** in sodium hydroxid in $D_2O$ (pH = 14) at room temperature over time.

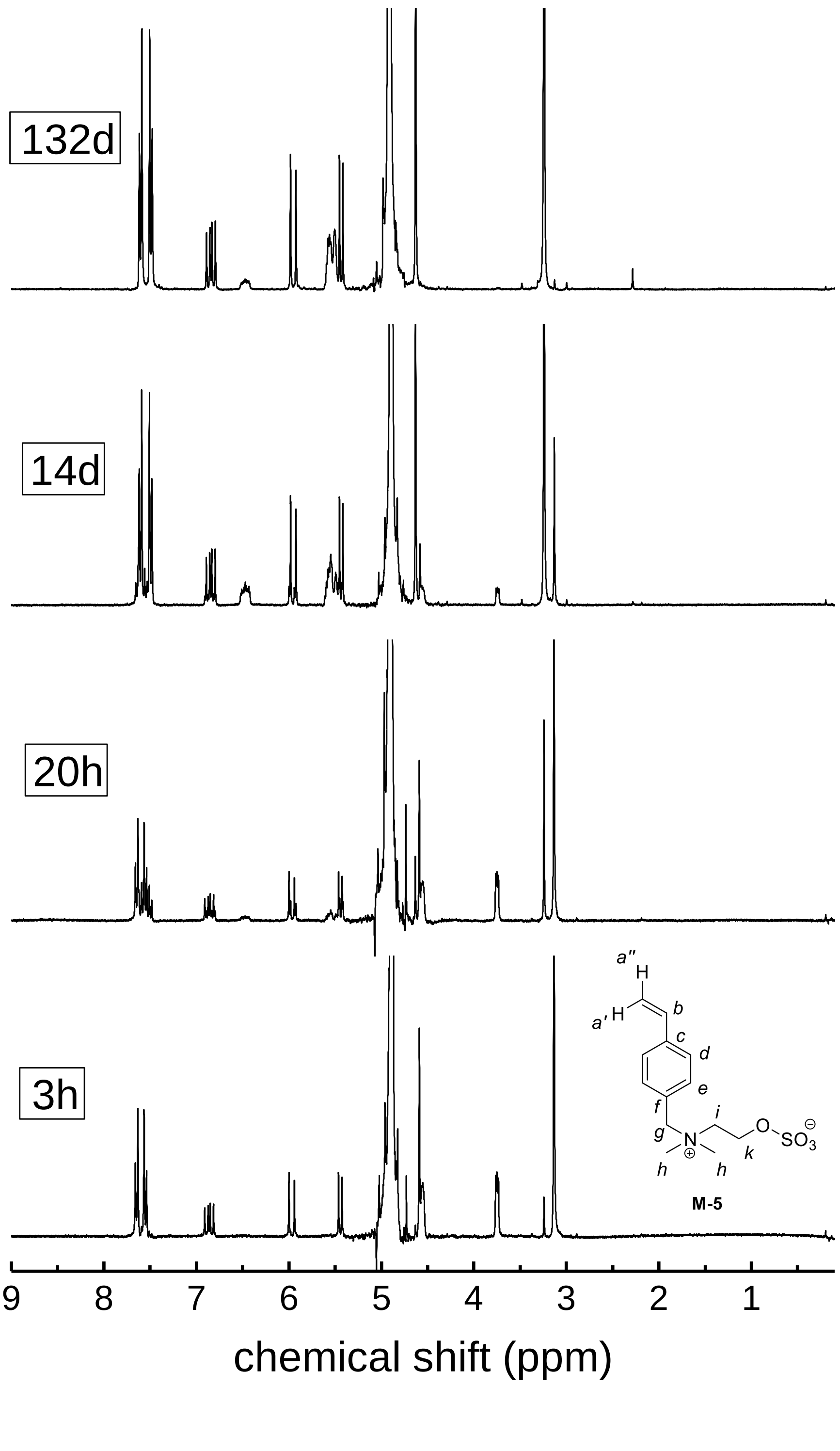


Figure S 68 $^{1}$H-NMR spectrum showing the degradation of 0.1 M solution of **M-5** in sodium hydroxid in $D_2O$ (pH = 14) at room temperature over time.

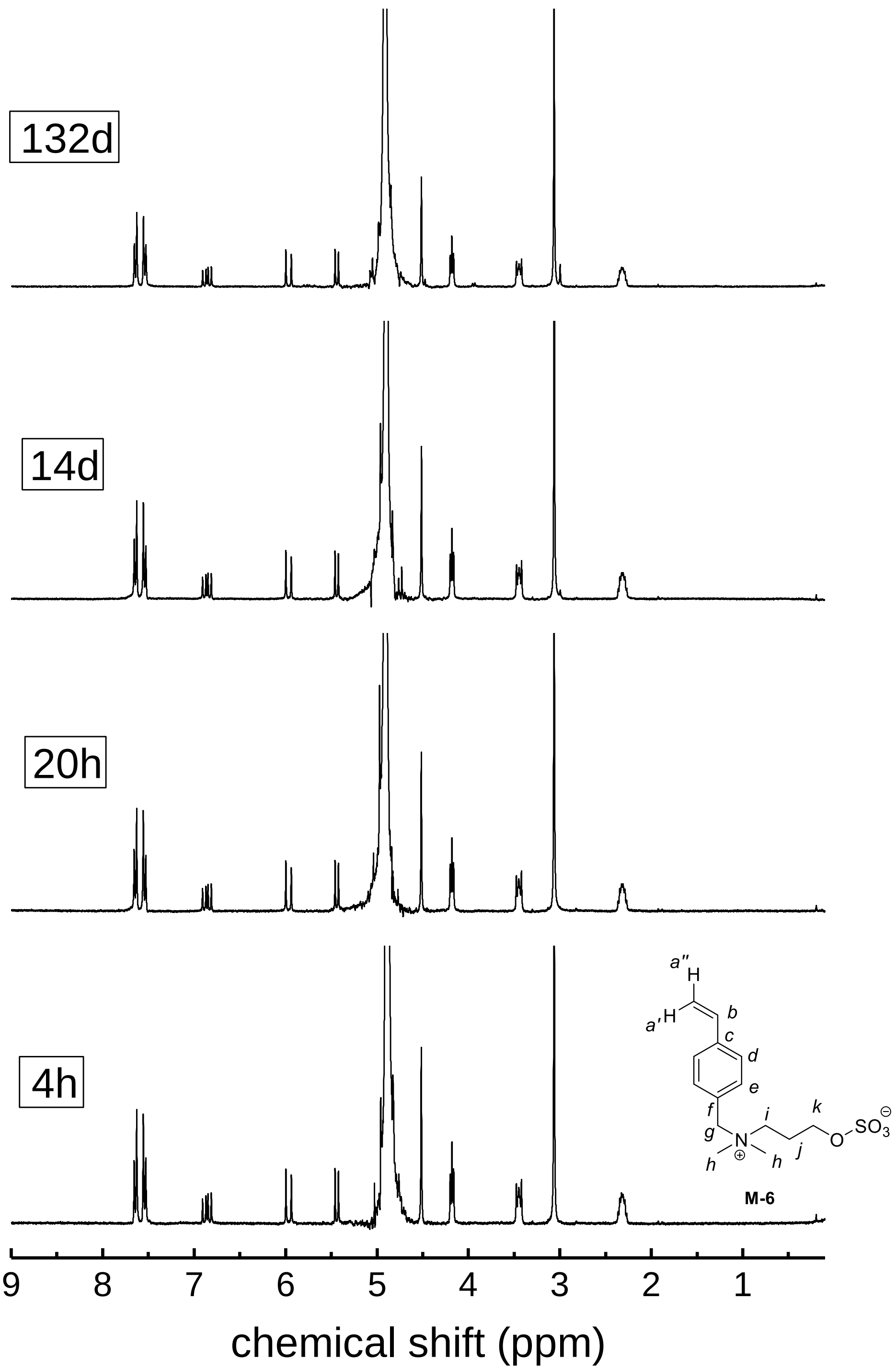


Figure S 69 $^{1}$H-NMR spectrum showing the degradation of 0.1 M solution of **M-6** in sodium hydroxid in $D_2O$ (pH = 14) at room temperature over time.

*3.1. 2D-Spektra ($^1H$-$^1H$-COSY) - Monomer hydrolysis pH=14*

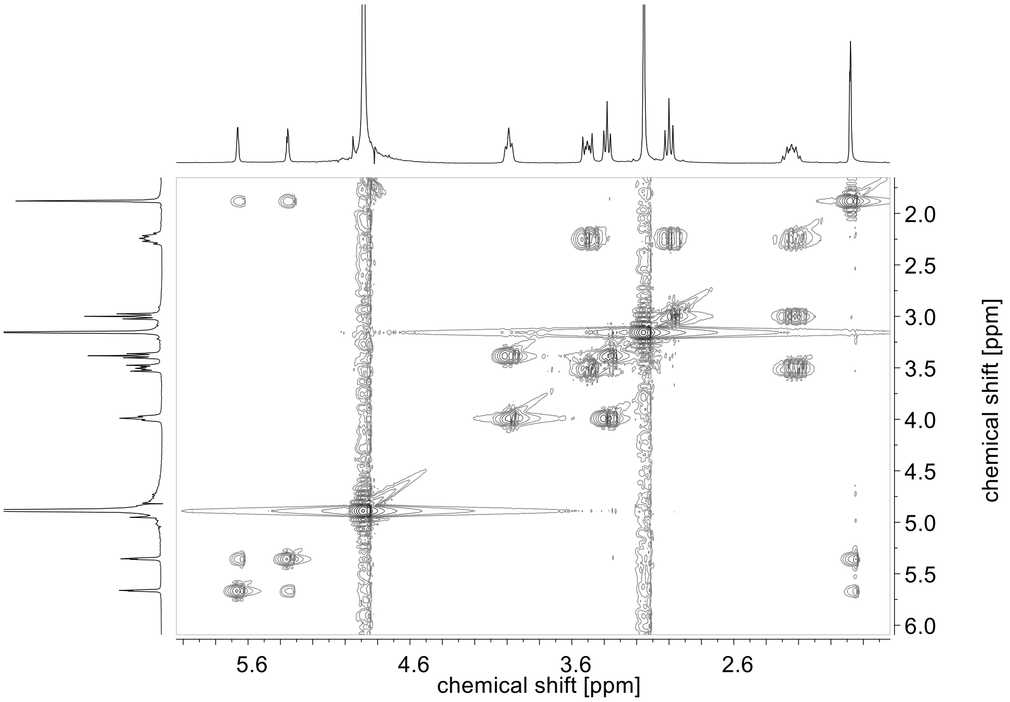


Figure S 70 $^1H$-$^1H$-COSY NMR spectra of 0.1 M solution of **SPE** sodium hydroxide in $D_2O$ (pH = 14), after 124 days.

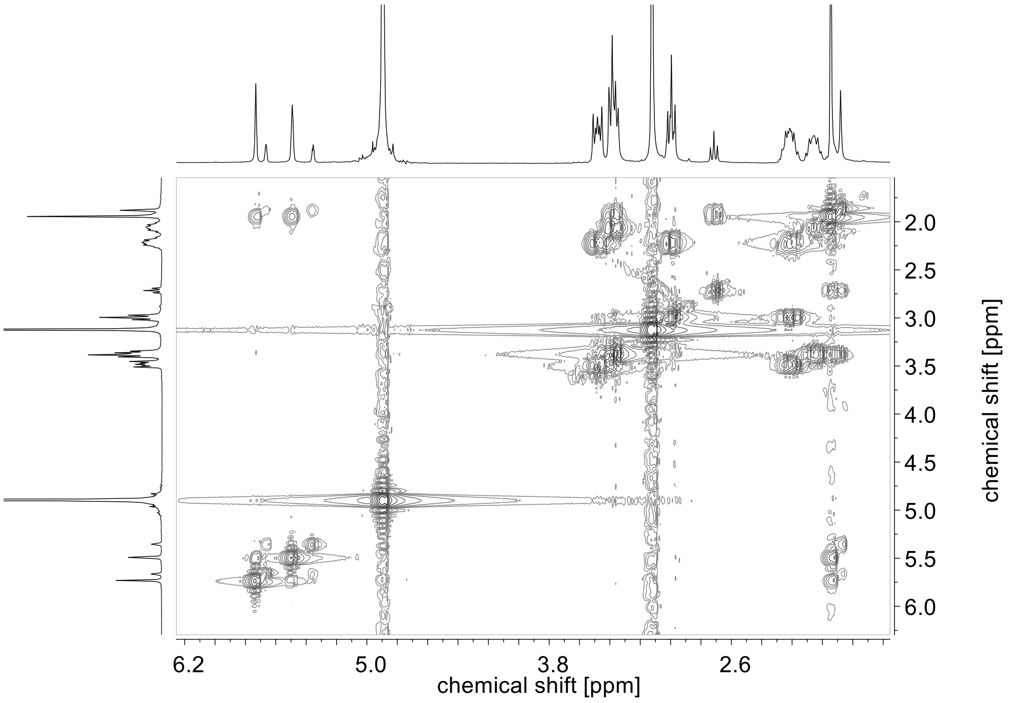


Figure S 71 $^1H$-$^1H$-COSY NMR spectra of 0.1 M solution of **SPP** sodium hydroxide in $D_2O$ (pH = 14), after 124 days.

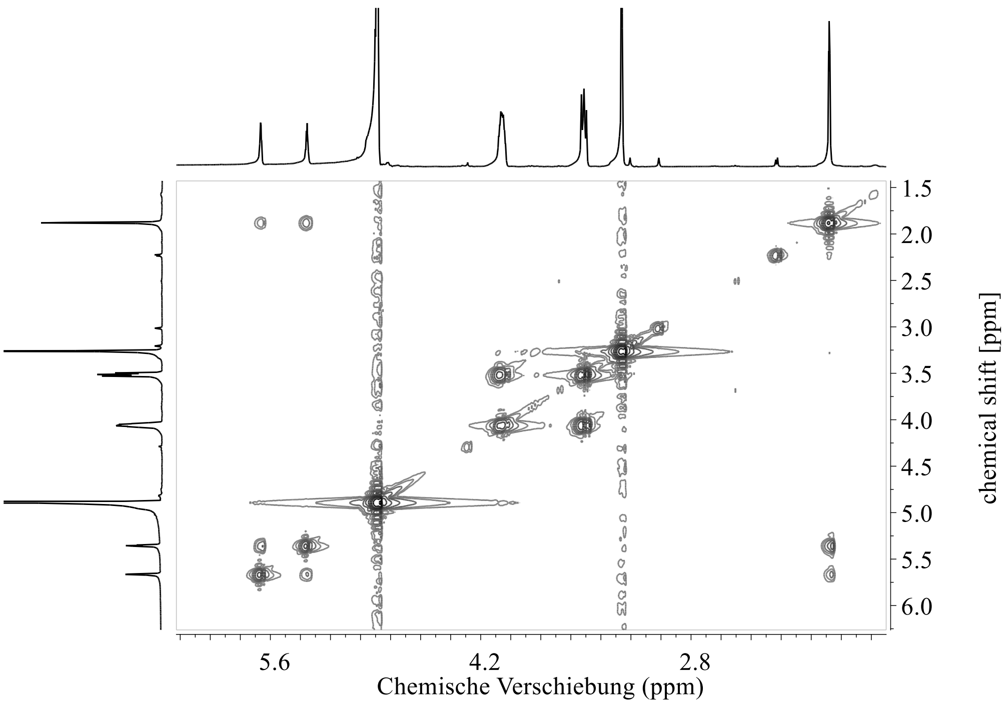


Figure S 72 $^{1}H$-$^{1}H$-COSY NMR spectra of 0.1 M solution of **M-1** sodium hydroxide in $D_2O$ (pH = 14), after 124 days.

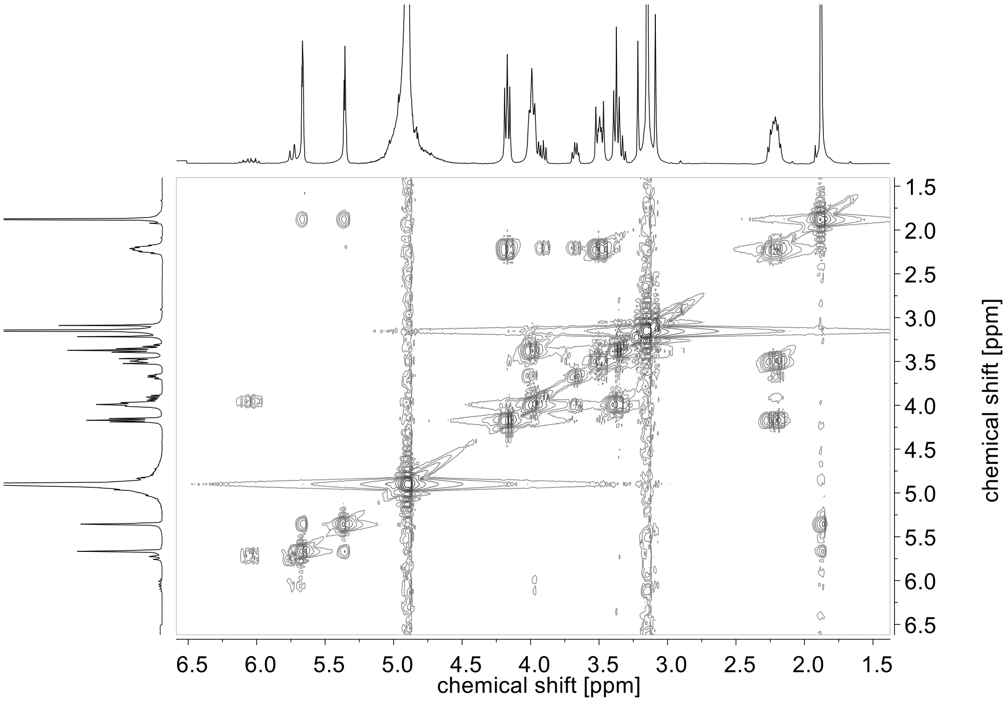


Figure S 73 $^{1}H$-$^{1}H$-COSY NMR spectra of 0.1 M solution of **M-2** sodium hydroxide in $D_2O$ (pH = 14), after 124 days.

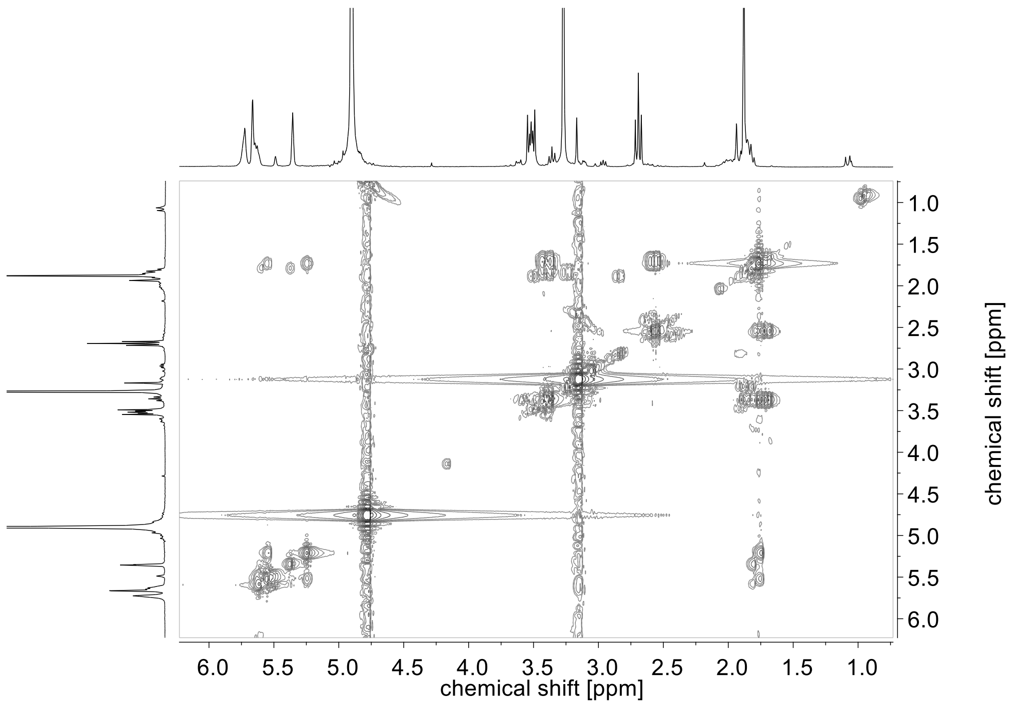


Figure S 74 $^{1}H$-$^{1}H$-COSY NMR spectra of 0.1 M solution of **M-3** sodium hydroxide in $D_2O$ (pH = 14), after 124 days.

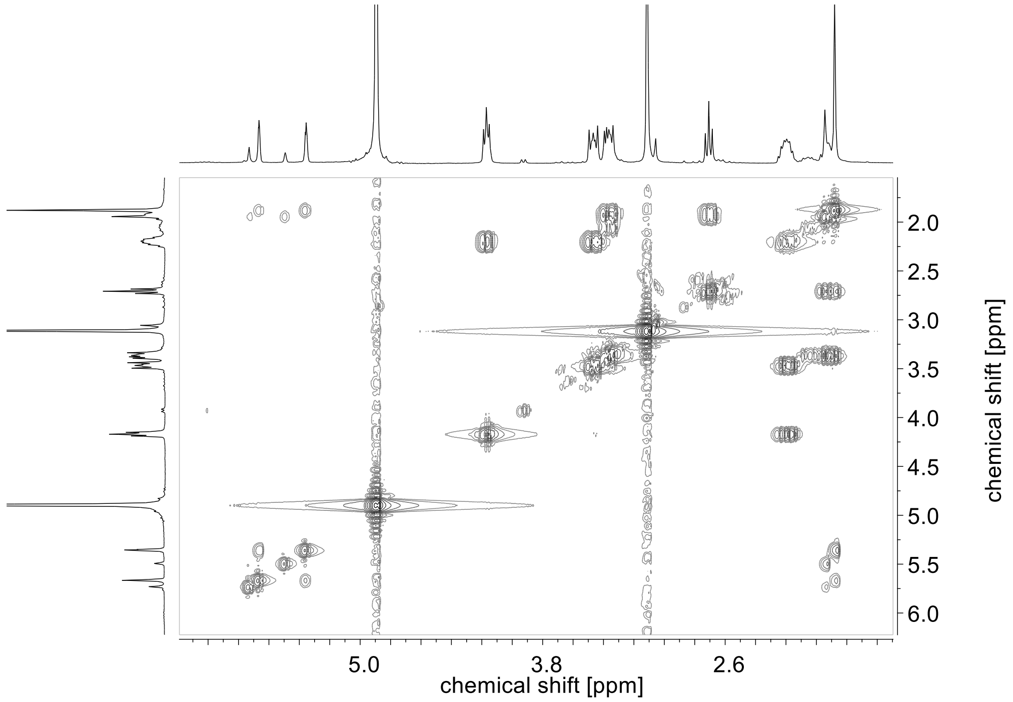


Figure S 75 $^{1}H$-$^{1}H$-COSY NMR spectra of 0.1 M solution of **M-4** sodium hydroxide in $D_2O$ (pH = 14), after 124 days.

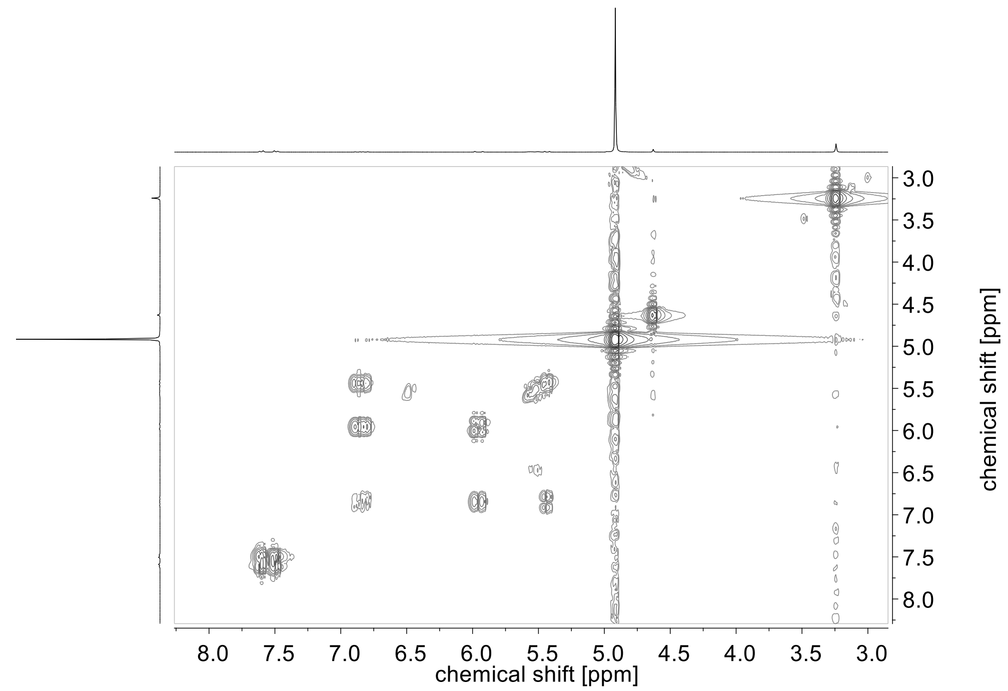


Figure S 76 $^{1}H$-$^{1}H$-COSY NMR spectra of 0.1 M solution of **M-5** sodium hydroxide in $D_2O$ (pH = 14), after 124 days.

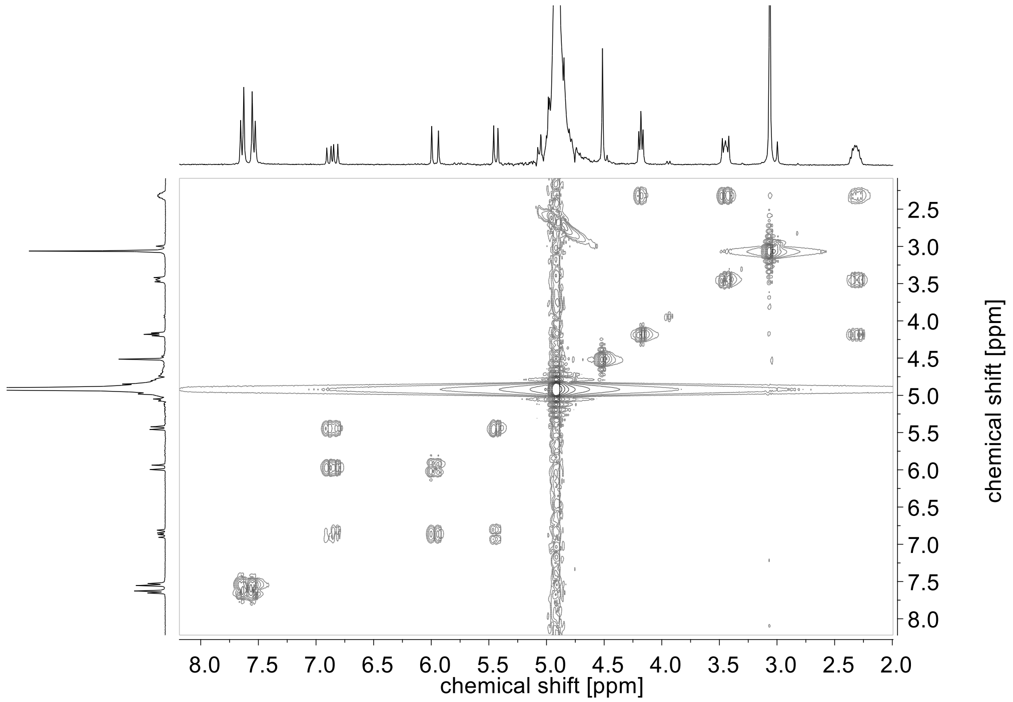


Figure S 77 $^{1}H$-$^{1}H$-COSY NMR spectra of 0.1 M solution of **M-6** sodium hydroxide in $D_2O$ (pH = 14), after 124 days.

4.5. Polymer hydrolysis in phosphate buffered saline (pH = 7.4)

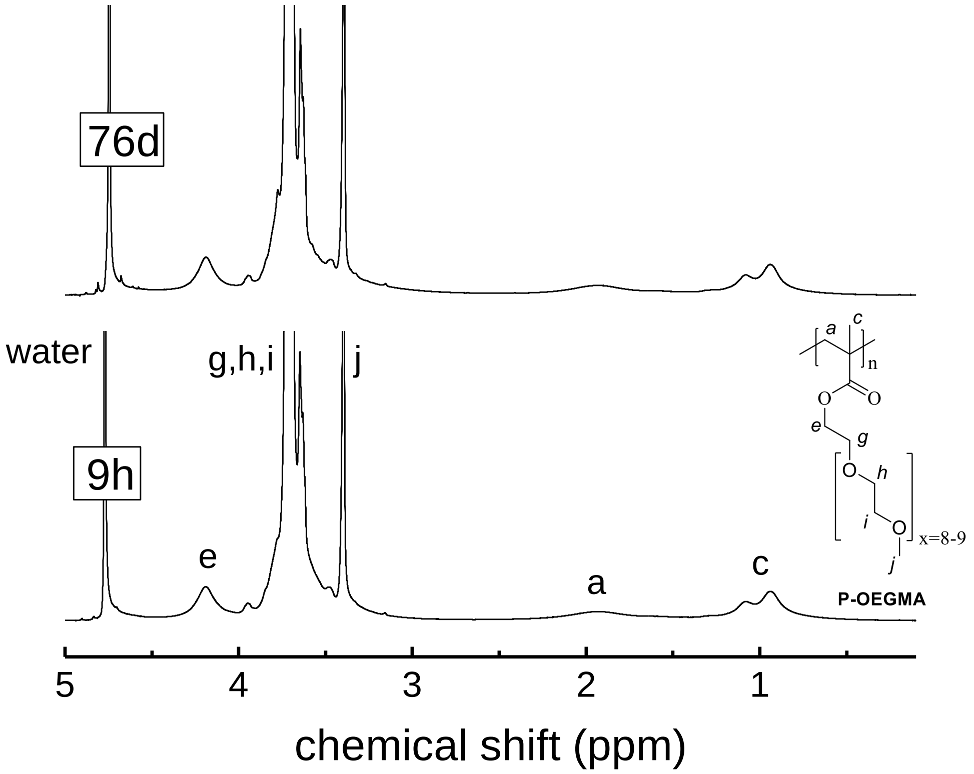


Figure S 78 $^{1}$H-NMR spectrum showing the degradation of **P-OEGMA** in phosphate buffered saline (PBS) in $D_2O$ (pH = 7.4) at room temperature over time.

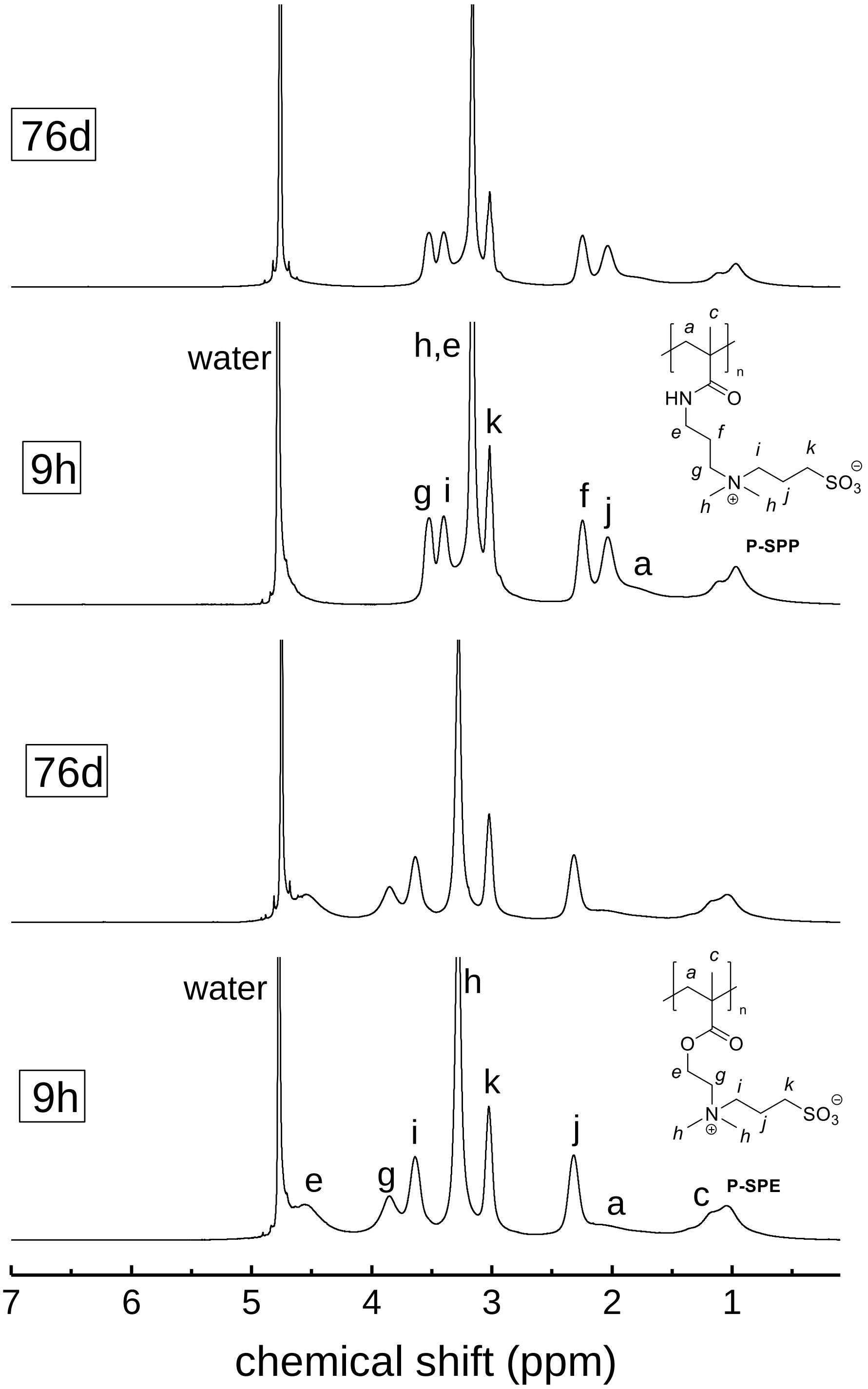


Figure S 79 $^{1}$H-NMR spectrum showing the degradation of **P-SPE** and **P-SPP** in phosphate buffered saline (PBS) in $D_2O$ (pH = 7.4) at room temperature over time.

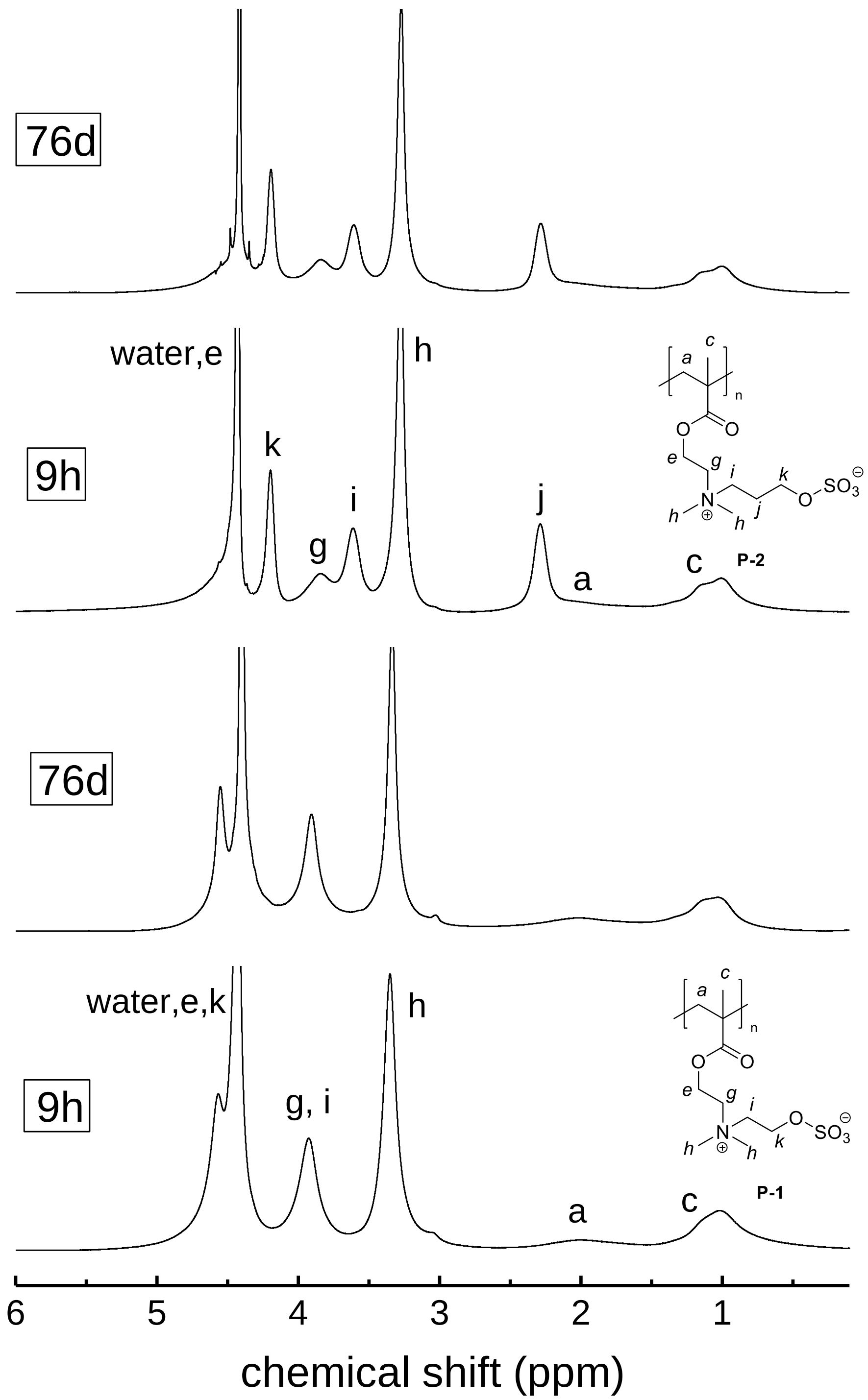


Figure S 80 $^{1}$H-NMR spectrum showing the degradation of **P-1** and **P-2** in phosphate buffered saline (PBS) in $D_2O$ saturated with sodium chloride (pH = 7.4) at room temperature over time.

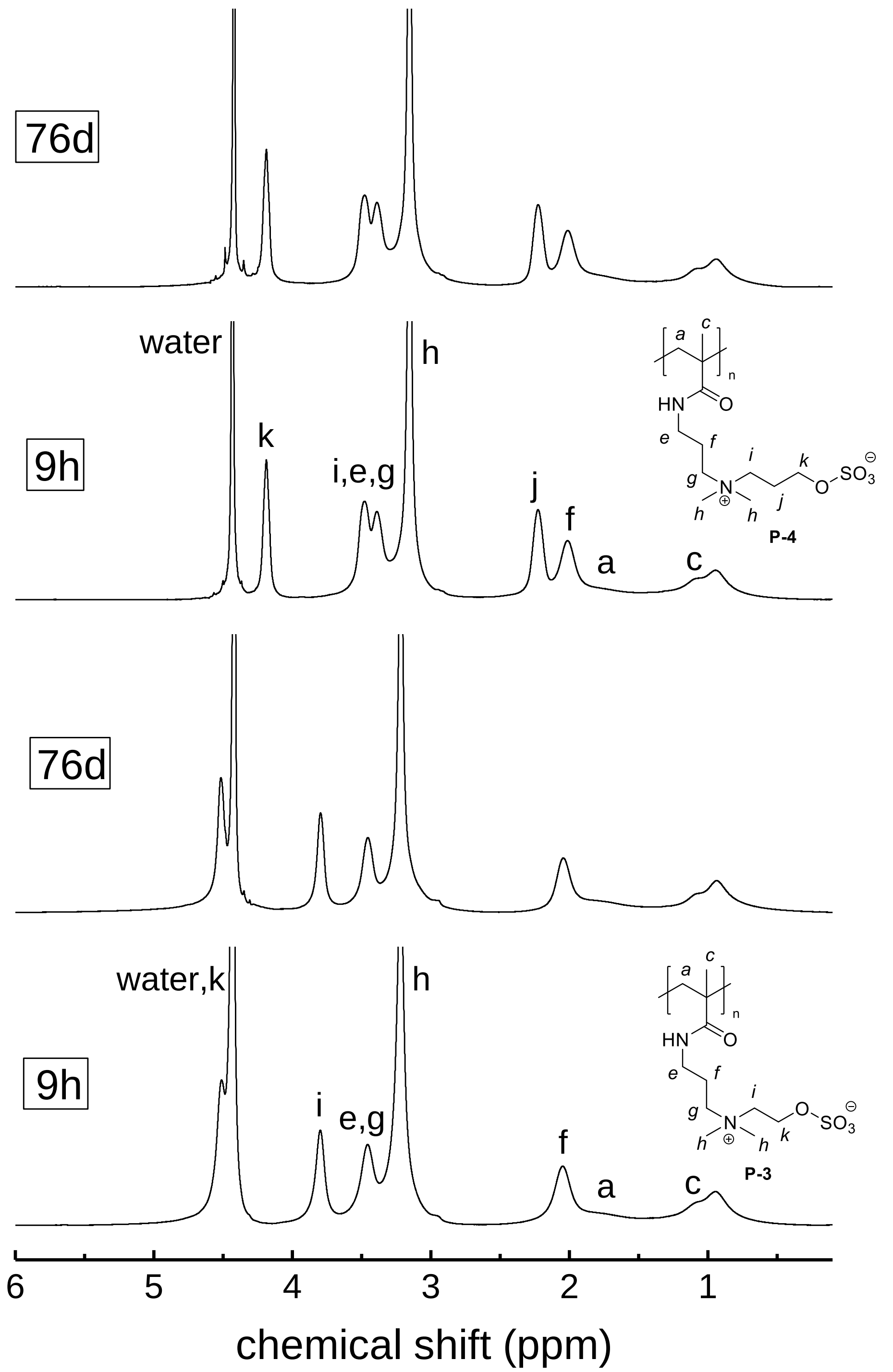


Figure S 81 $^{1}$H-NMR spectrum showing the degradation of **P-3** and **P-4** in phosphate buffered saline (PBS) in $D_2O$ saturated with sodium chloride (pH = 7.4) at room temperature over time.

4.6. Polymer hydrolysis in 1 M hydrochloric acid pH=0

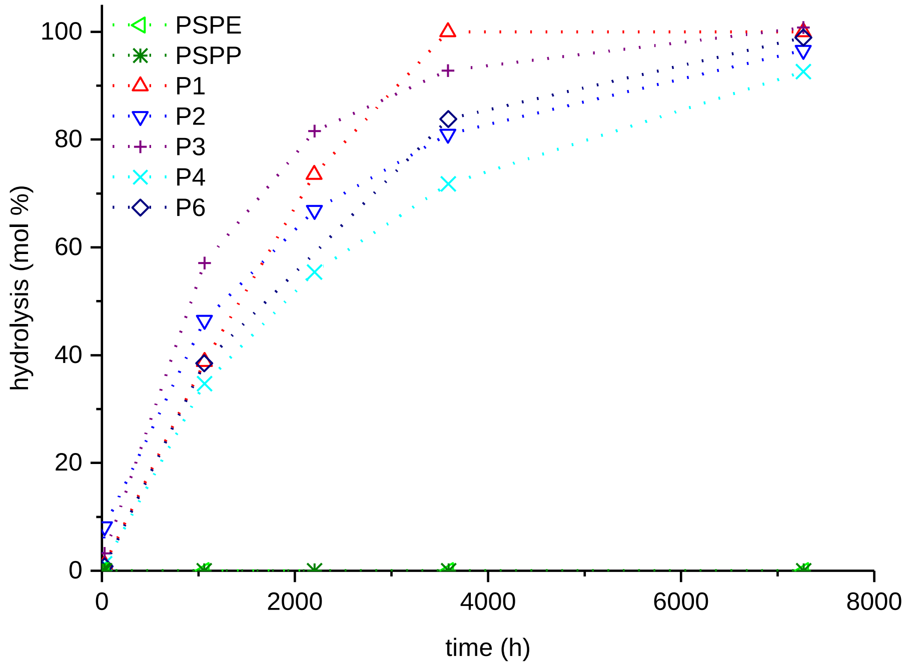


Figure S 82 Evolution of sulfate and sulfonate hydrolysis of polymers in 1 M hydrochloric acid in $D_2O$ (pH=0): (□) = SPE, (*) = SPP, (□) = M1, (□) = M2, (+) = M3, (X) = M4, (□) = M5, (□) = M6.

Calculation of hydrolysis in mol %:

$$Hydrolyse_{\text{M-1}}\ [\text{mol }\%] = \left(\frac{[I_{k_3+e_3} - 2 * I_{a_2'}]/2}{[I_{g+i} - 2 * I_{a_2'}]/2 + [I_{k_3+e_3} - 2 * I_{a_2'}]/2} * 100\right)$$

$$Hydrolyse_{M-2}\ [\text{mol }\%] = \left(\frac{I_{j_3}*100}{I_{j_2}+I_{j_3}}\right)$$

$$Hydrolyse_{M-3}\ [\text{mol }\%] = \left(\frac{I_{k_3}*100}{I_{k}+I_{k_3}}\right)$$

$$Hydrolyse_{M-4}\ [\text{mol }\%] = \left(\frac{I_{k_3}*100}{I_{k}+I_{k_3}}\right)$$

$$Hydrolyse_{M-5}\ [\text{mol }\%] = \left(\frac{I_{i_3}*100}{I_{i}+I_{i_3}}\right)$$

$$Hydrolyse_{M-6}\ [\text{mol }\%] = \left(\frac{I_{j_3}*100}{I_{j_2}+I_{j_3}}\right)$$

$$Hydrolyse_{SPE}\ [\text{mol }\%] = \left(\frac{I_{k_3}*100}{I_{k+k_2}+I_{k_3}}\right)$$

$$Hydrolyse_{SPP}\ [\text{mol }\%] = \left(\frac{I_{k_3}*100}{I_{k}+I_{k_3}}\right)$$

The Index 2 in e.g. $I_{e_2}$ indicates the hydrolysis product of the ester/amid product, while no index e.g. $I_{a''}$ determines the unchanged molecule without hydrolysis

$I_{k_3+e_3}(M\text{-}1,\ range\ in\ ppm)$ = 4.6-4.4
$I_{a_2'}(M\text{-}1,\ range\ in\ ppm)$ = 6.1-6.2
$I_{g+i}(M\text{-}1,\ range\ in\ ppm)$ = 4.2-4.0
$I_{j_3}(M\text{-}2,\ range\ in\ ppm)$ = 2.2-2.0
$I_{j_2}(M\text{-}2,\ range\ in\ ppm)$ = 2.4-2.2
$I_{k_3}(M\text{-}3,\ range\ in\ ppm)$ = 4.2-3.9
$I_{k}(M\text{-}3,\ range\ in\ ppm)$ = 4.6-4.3
$I_{k_3}(M\text{-}4,\ range\ in\ ppm)$ = 3.8-3.6
$I_{k}(M\text{-}4,\ range\ in\ ppm)$ = 4.3-4.0
$I_{i_3}(M\text{-}5,\ range\ in\ ppm)$ = 3.6-3.4
$I_{i}(M\text{-}5,\ range\ in\ ppm)$ = 3.9-3.6
$I_{j_3}(M\text{-}6,\ range\ in\ ppm)$ = 2.2-2.0
$I_{j}(M\text{-}6,\ range\ in\ ppm)$ = 2.4-2.2
$I_{k_3}(SPE,\ range\ in\ ppm)$ = no signal
$I_{k+k_2}(SPE,\ range\ in\ ppm)$ = 3.1-2.9
$I_{k_3}(SPP,\ range\ in\ ppm)$ = no signal
$I_{k}(SPP,\ range\ in\ ppm)$ = 3.1-2.9

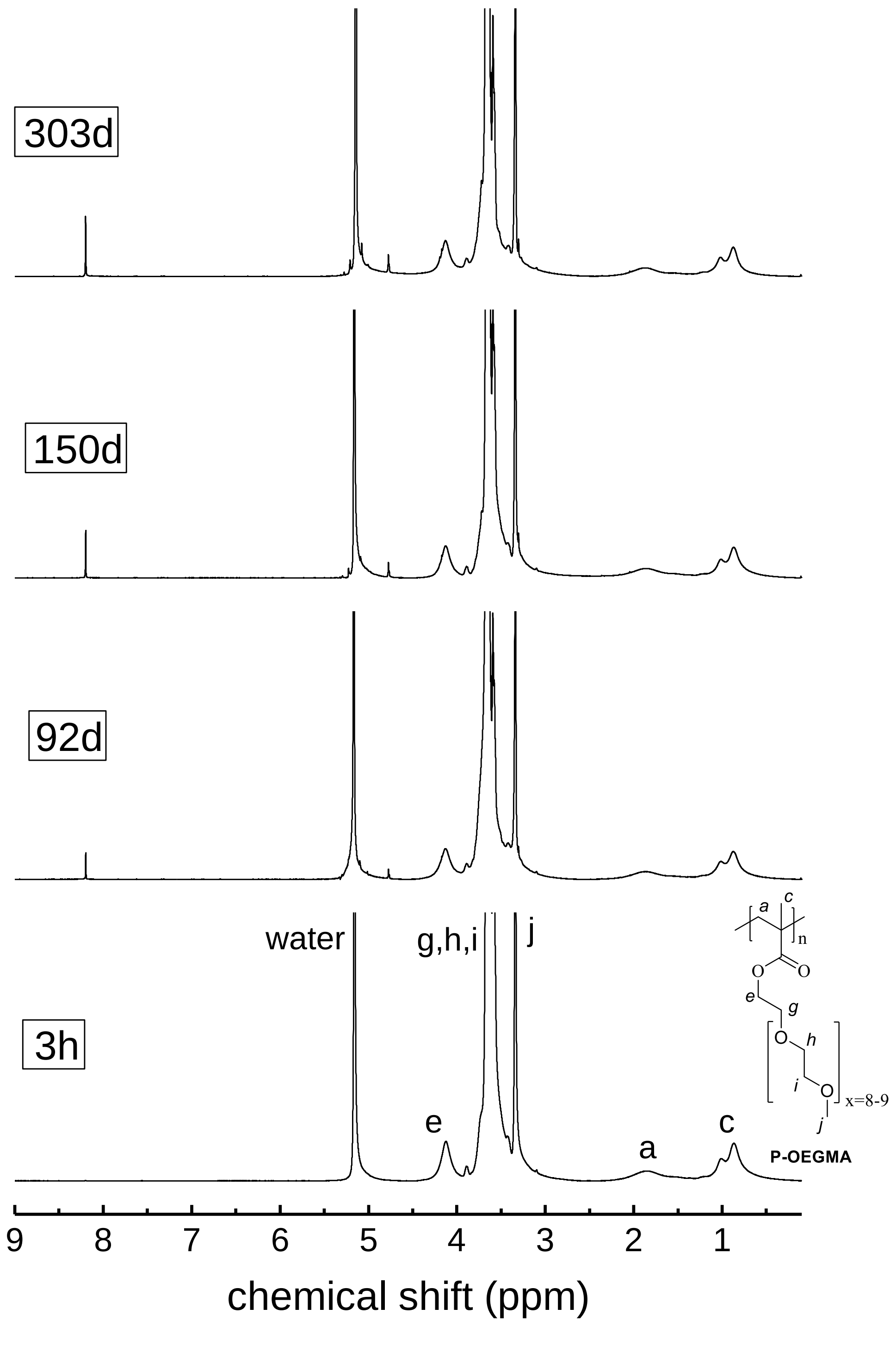


Figure S 83 $^{1}$H-NMR spectrum showing the degradation of **P-OEGMA** in 1 M hydrochloric acid in $D_2O$ (pH = 0) at room temperature over time.

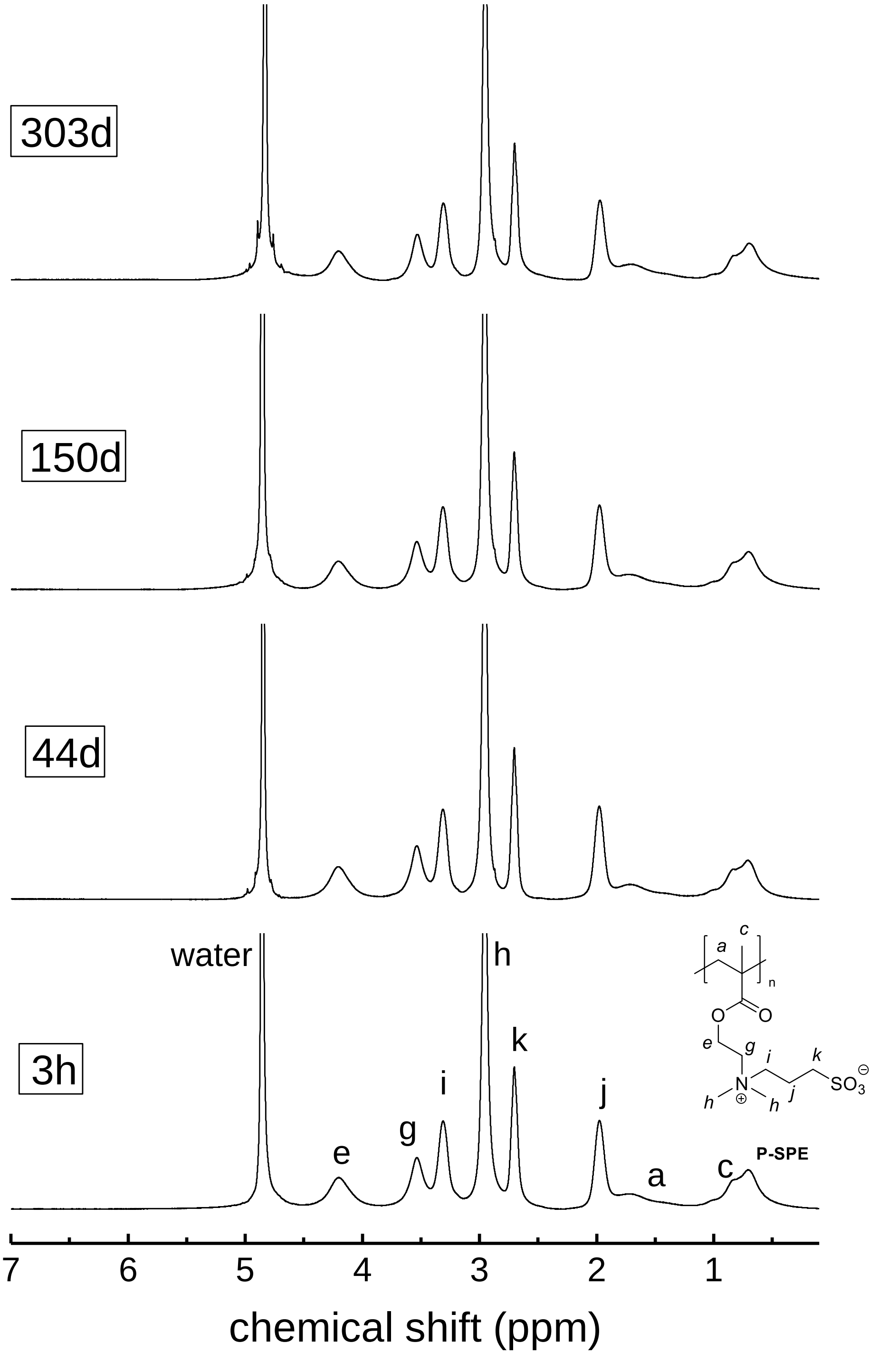


Figure S 84 $^{1}$H-NMR spectrum showing the degradation of **P-SPE** in 1 M hydrochloric acid in $D_2O$ (pH = 0) at room temperature over time.

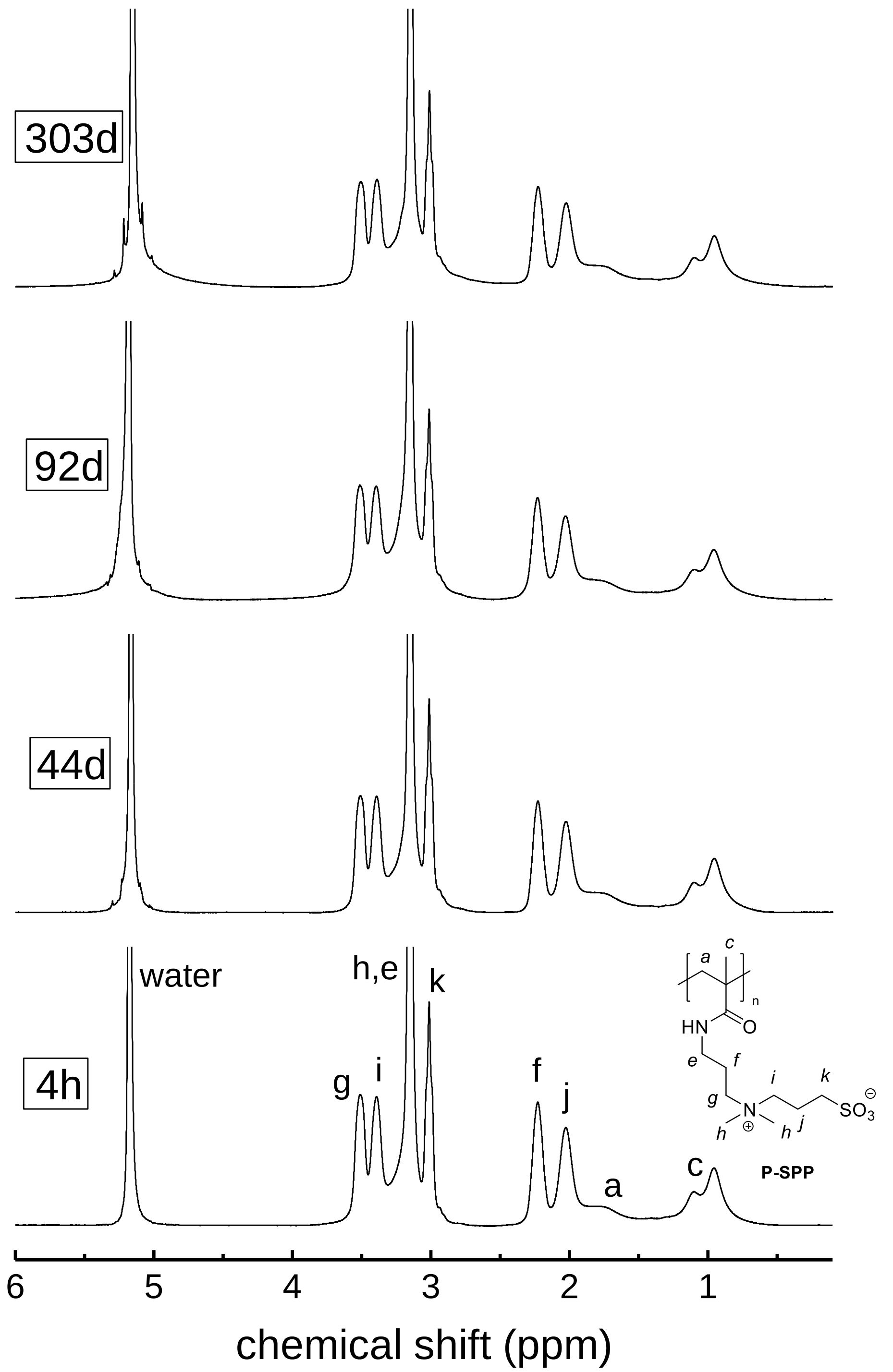


Figure S 85 $^{1}$H-NMR spectrum showing the degradation of **P-SPP** in 1 M hydrochloric acid in $D_2O$ (pH = 0) at room temperature over time.

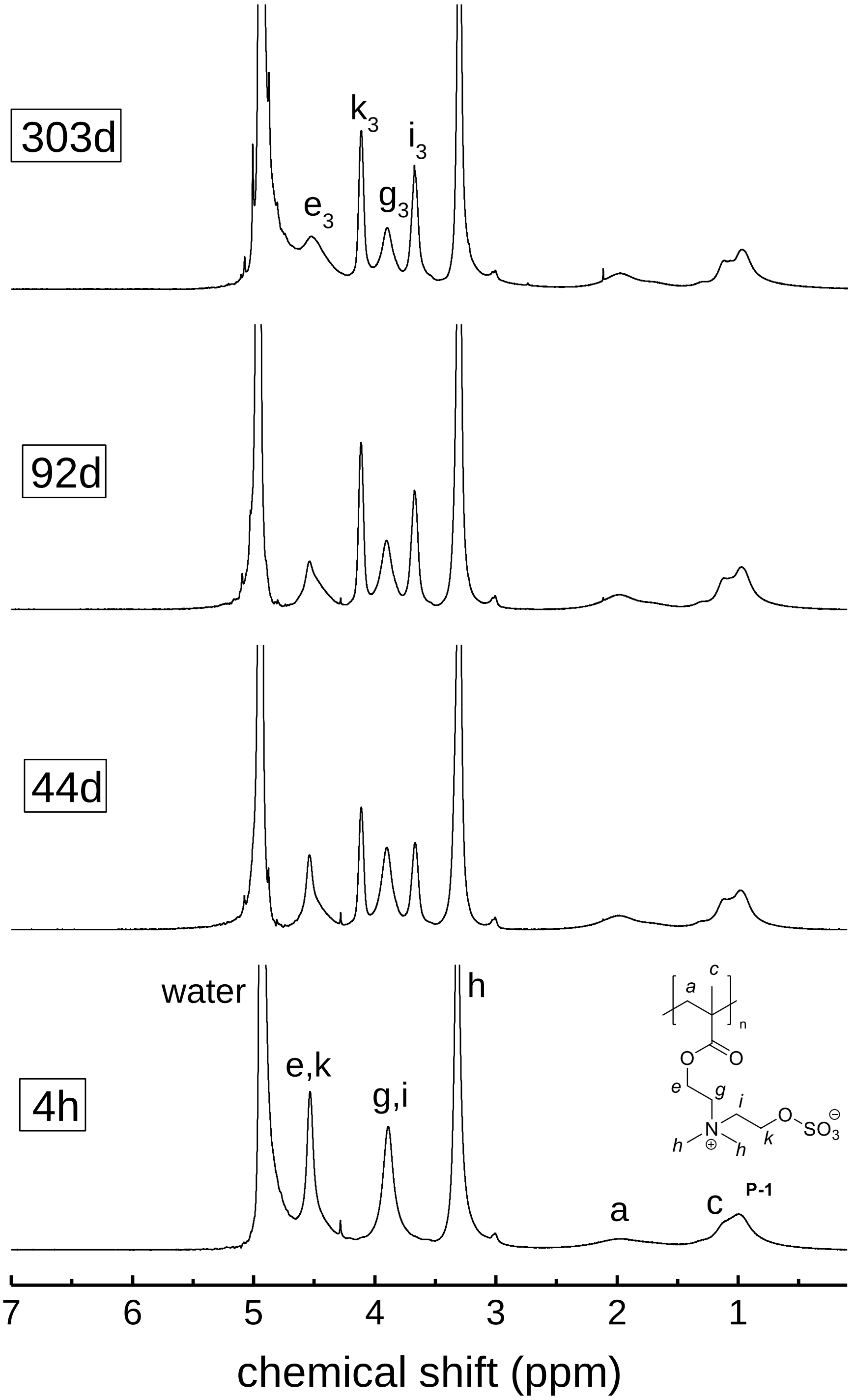


Figure S 86 $^{1}$H-NMR spectrum showing the degradation of **P-1** in 1 M hydrochloric acid in $D_2O$ (pH = 0) saturated with sodium chloride at room temperature over time.

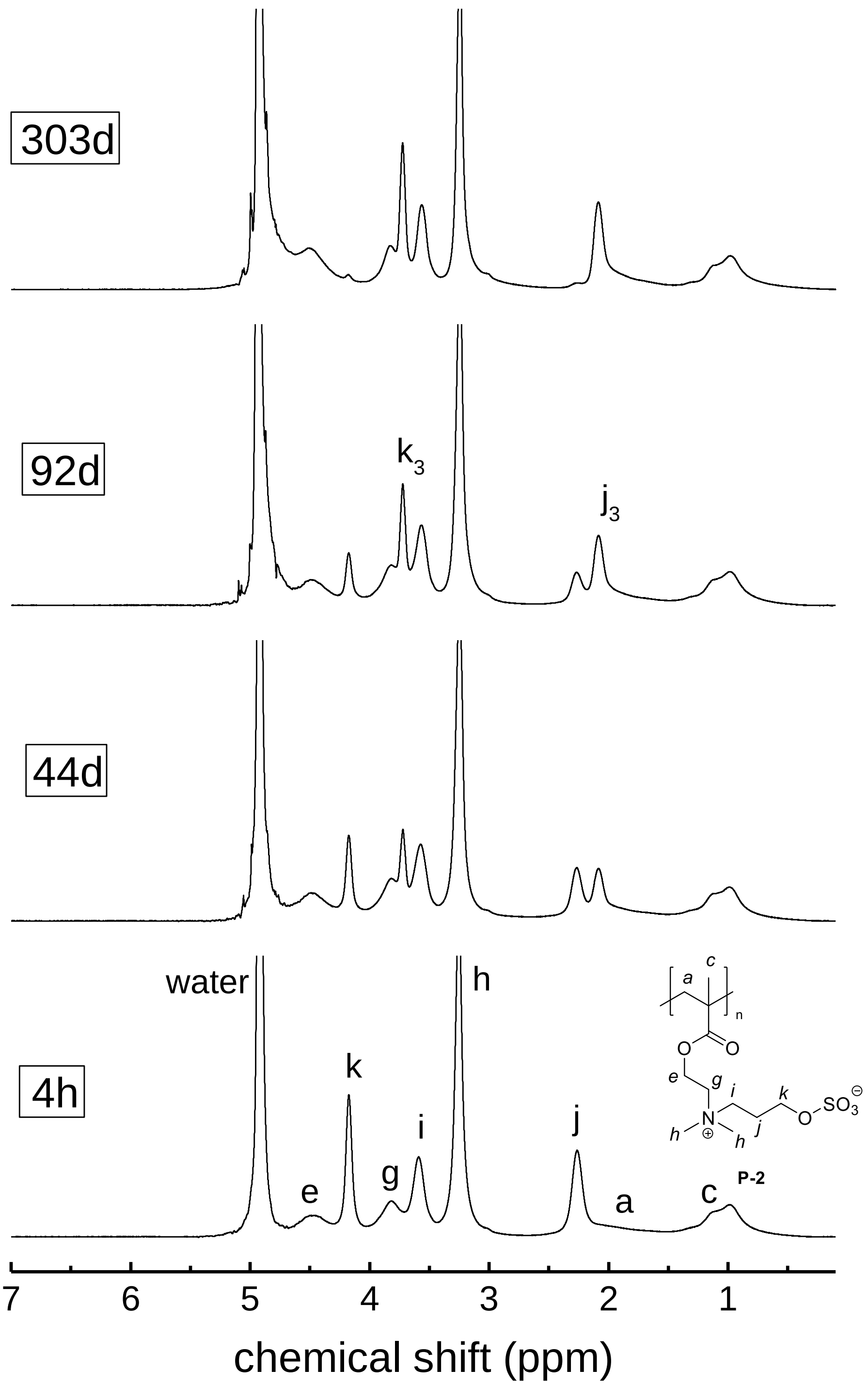


Figure S 87 $^{1}$H-NMR spectrum showing the degradation of **P-2** in 1 M hydrochloric acid in $D_2O$ (pH = 0) saturated with sodium chloride at room temperature over time.

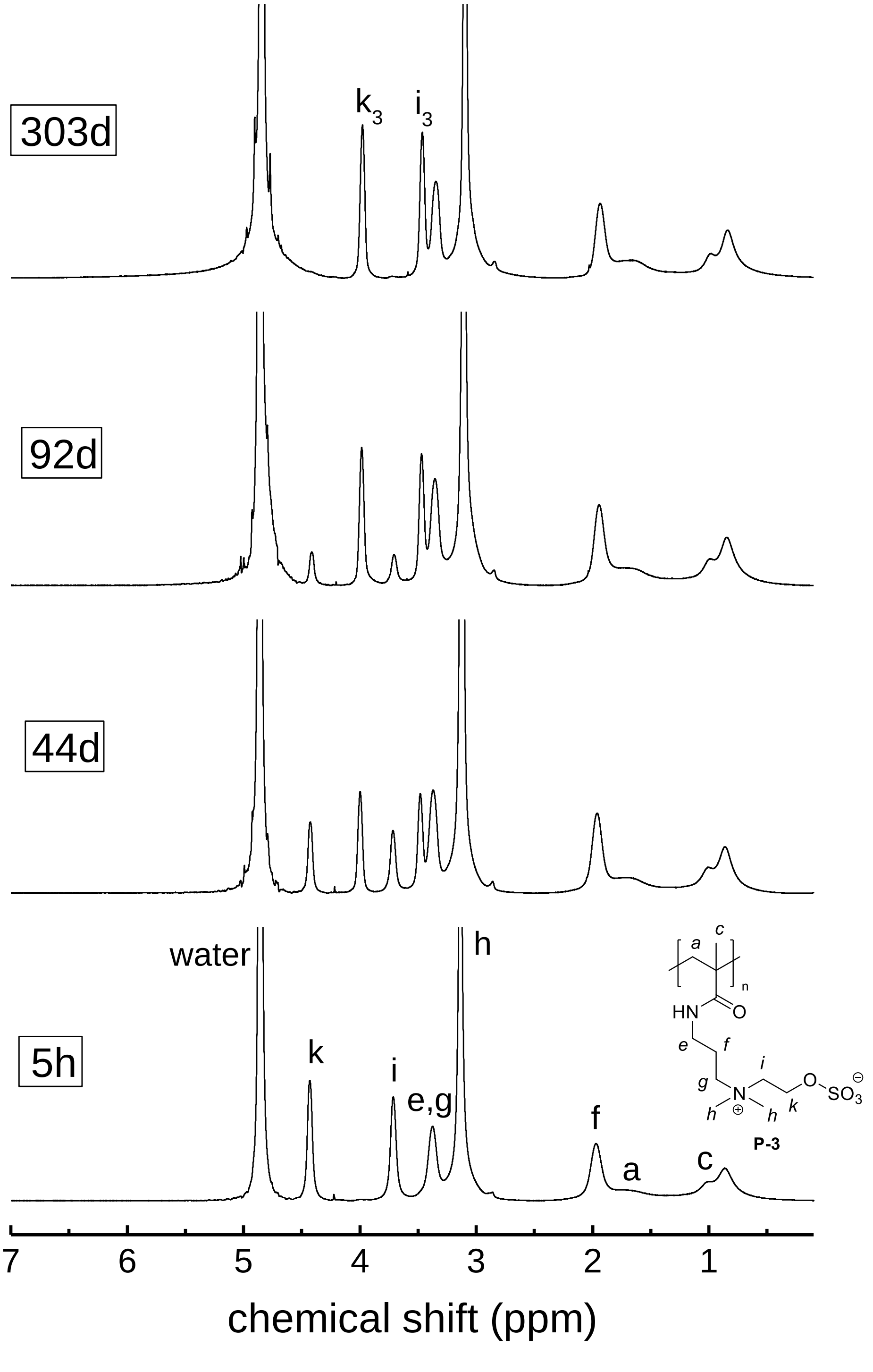


Figure S 88 $^{1}$H-NMR spectrum showing the degradation of **P-3** in 1 M hydrochloric acid in $D_2O$ (pH = 0) saturated with sodium chloride at room temperature over time.

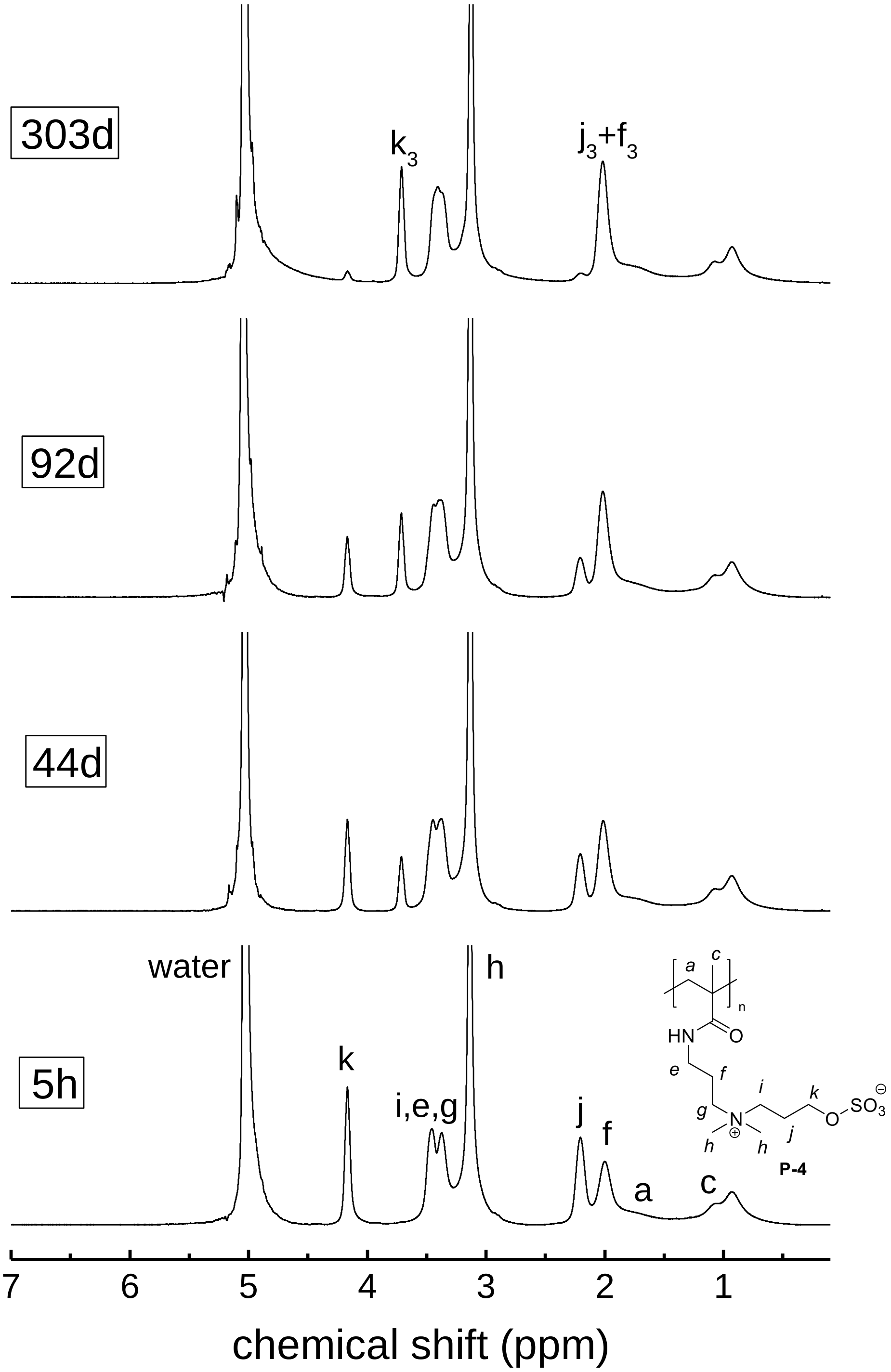


Figure S 89 $^{1}$H-NMR spectrum showing the degradation of **P-4** in 1 M hydrochloric acid in $D_2O$ (pH = 0) saturated with sodium chloride at room temperature over time.

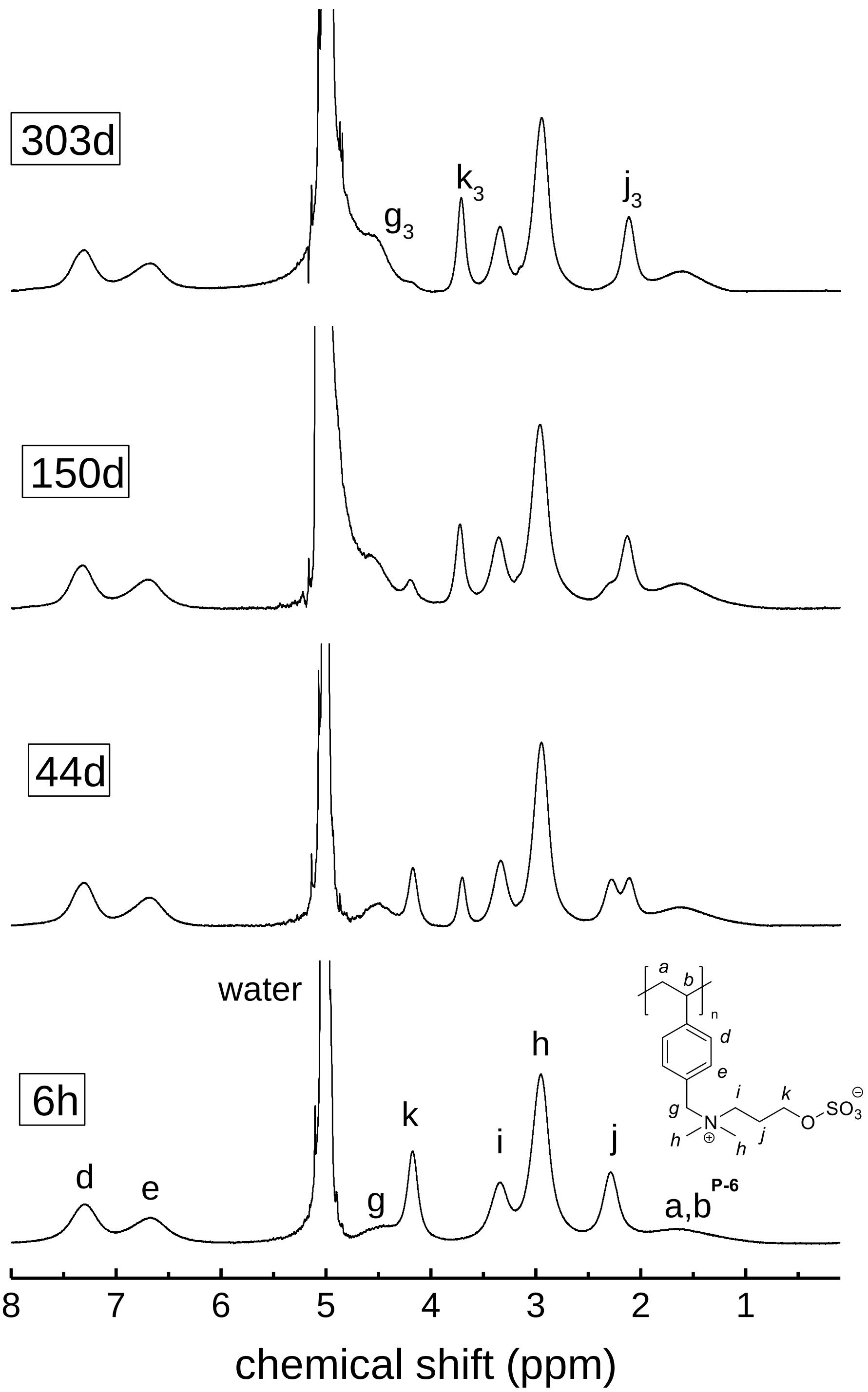


Figure S 90  ¹H-NMR spectrum showing the degradation of **P-6** in 1 M hydrochloric acid in $D_2O$ (pH = 0) saturated with sodium chloride at room temperature over time.

4.7. Polymer hydrolysis hydrogen carbonate buffer (pH=10)

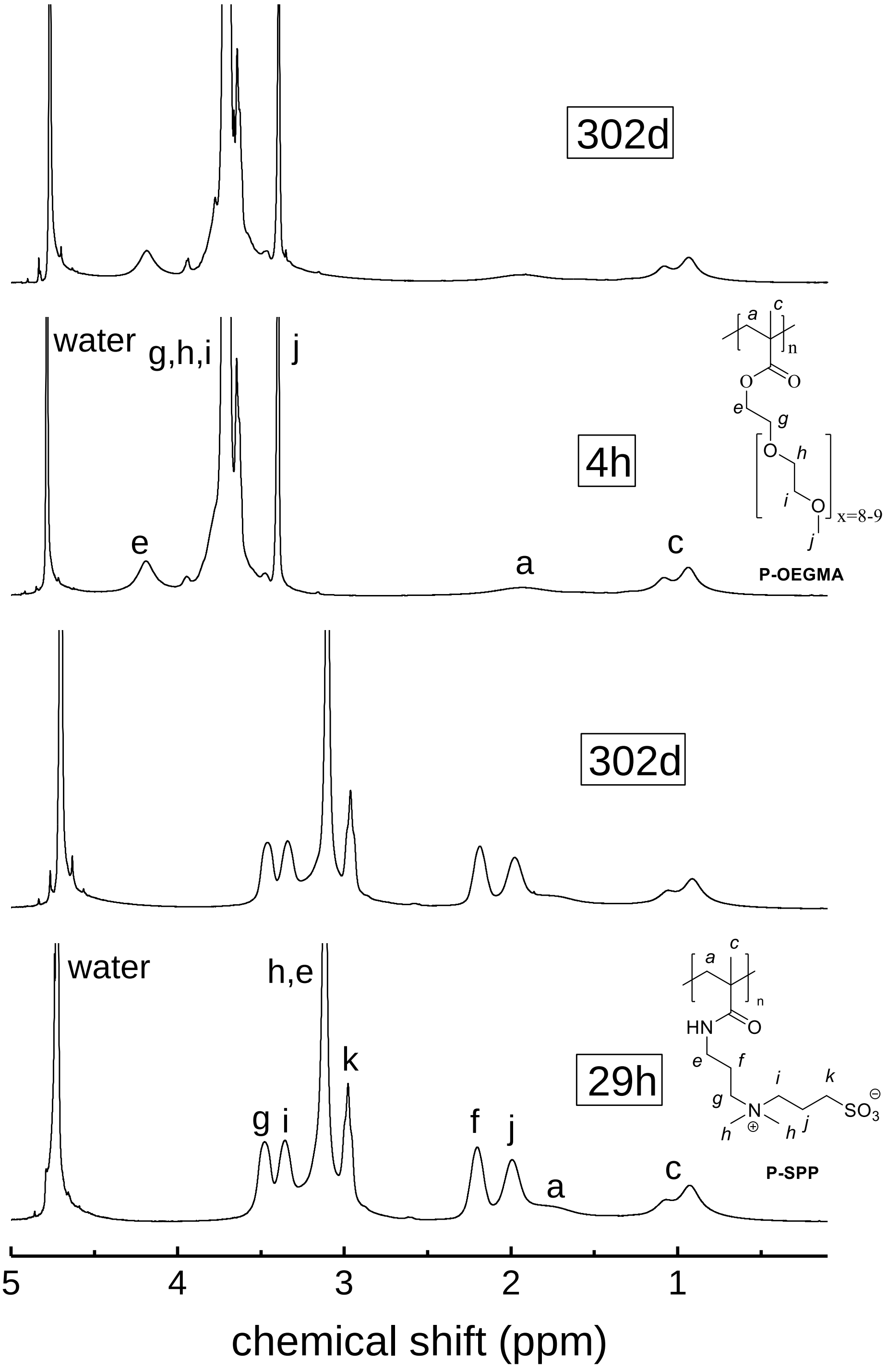


Figure S 91 $^{1}$H-NMR spectrum showing the degradation of **P-OEGMA** and **P-SPP** in carbonate buffer in (pH = 10) at room temperature over time.

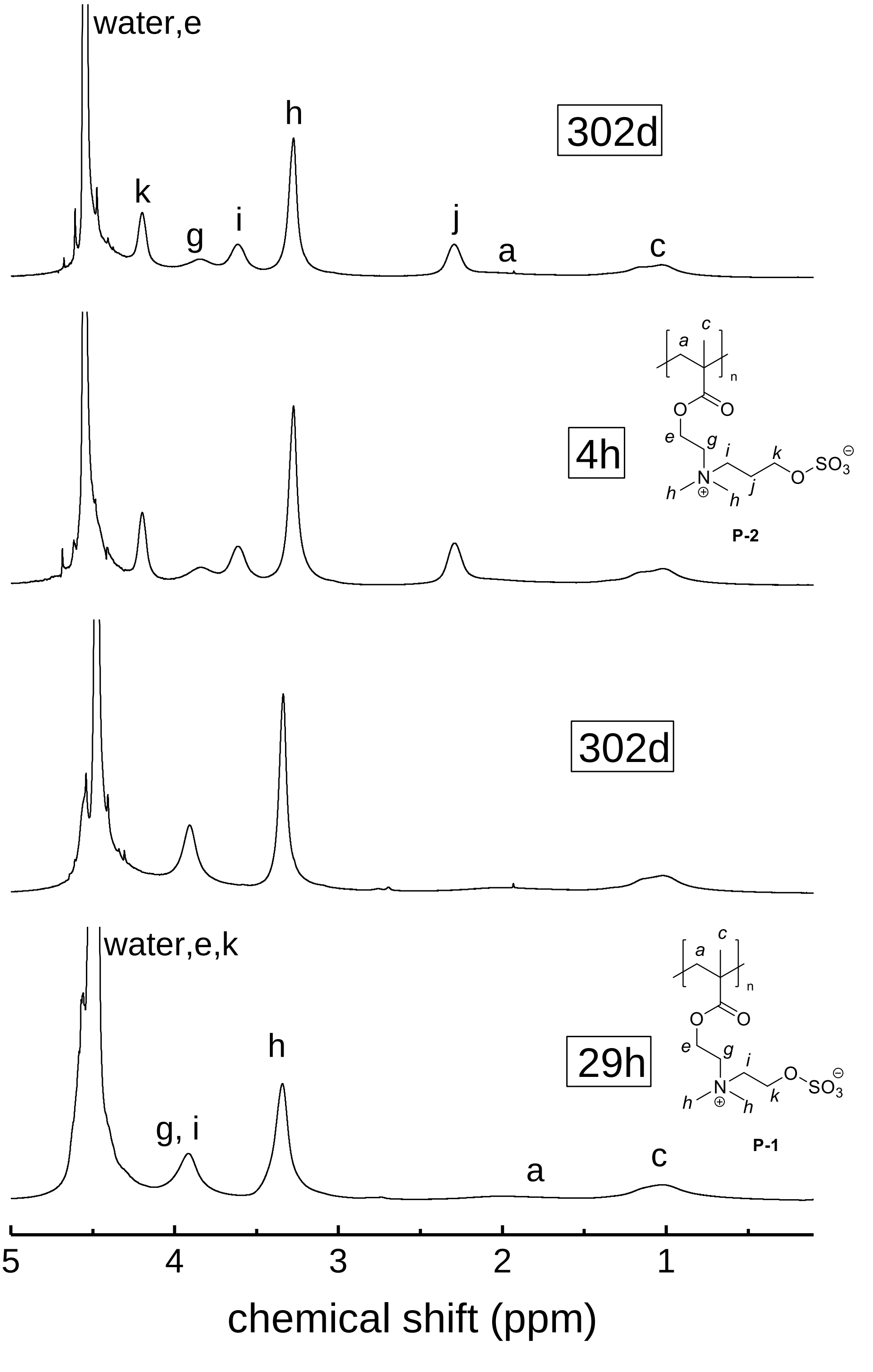


Figure S 92 $^{1}$H-NMR spectrum showing the degradation of **P-1** and **P-2** in carbonate buffer in $D_2O$ saturated with sodium chloride (pH = 10) at room temperature over time.

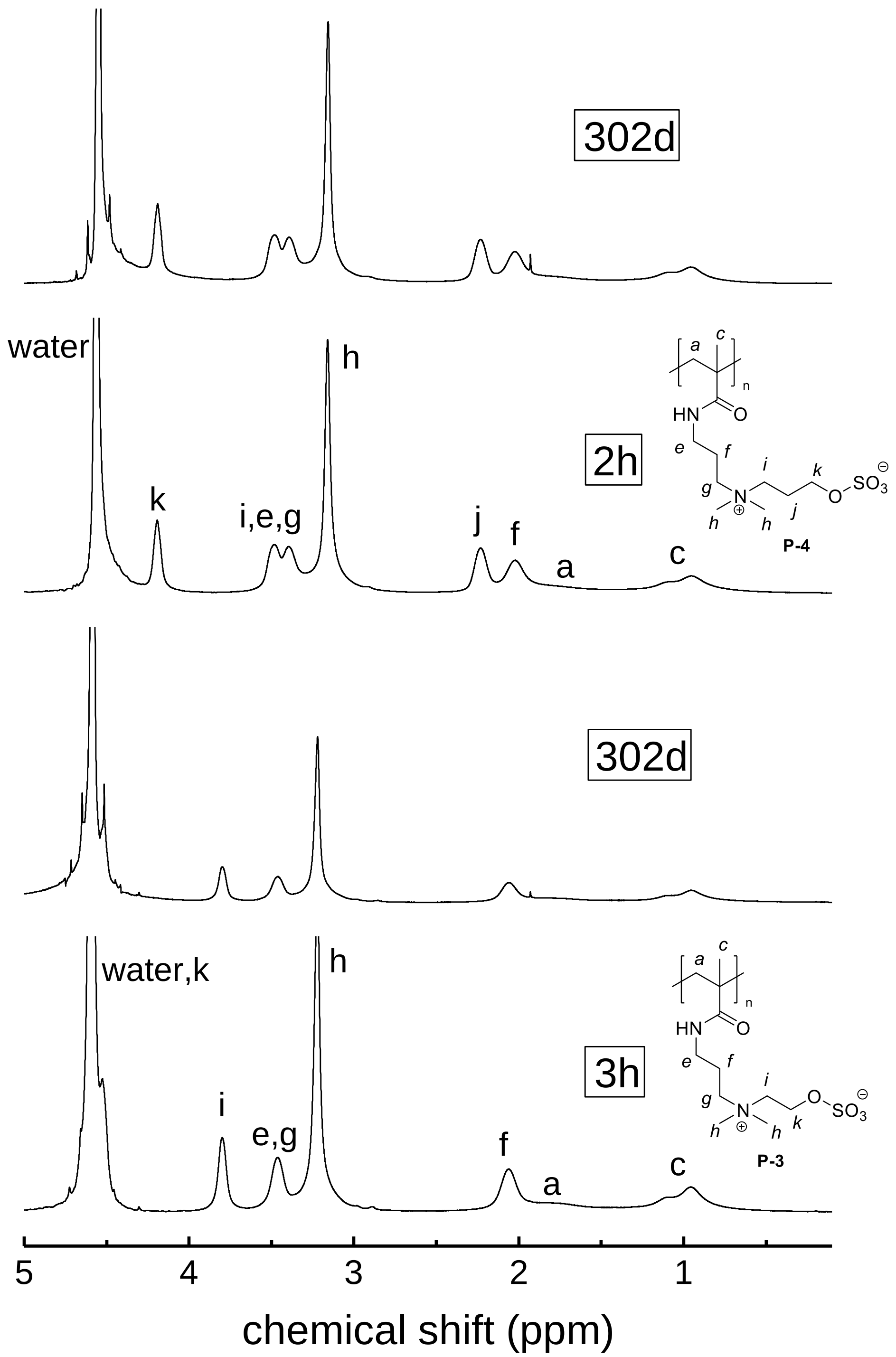


Figure S 93 $^{1}$H-NMR spectrum showing the degradation of **P-3** and **P-4** in carbonate buffer in $D_2O$ saturated with sodium chloride (pH = 10) at room temperature over time.

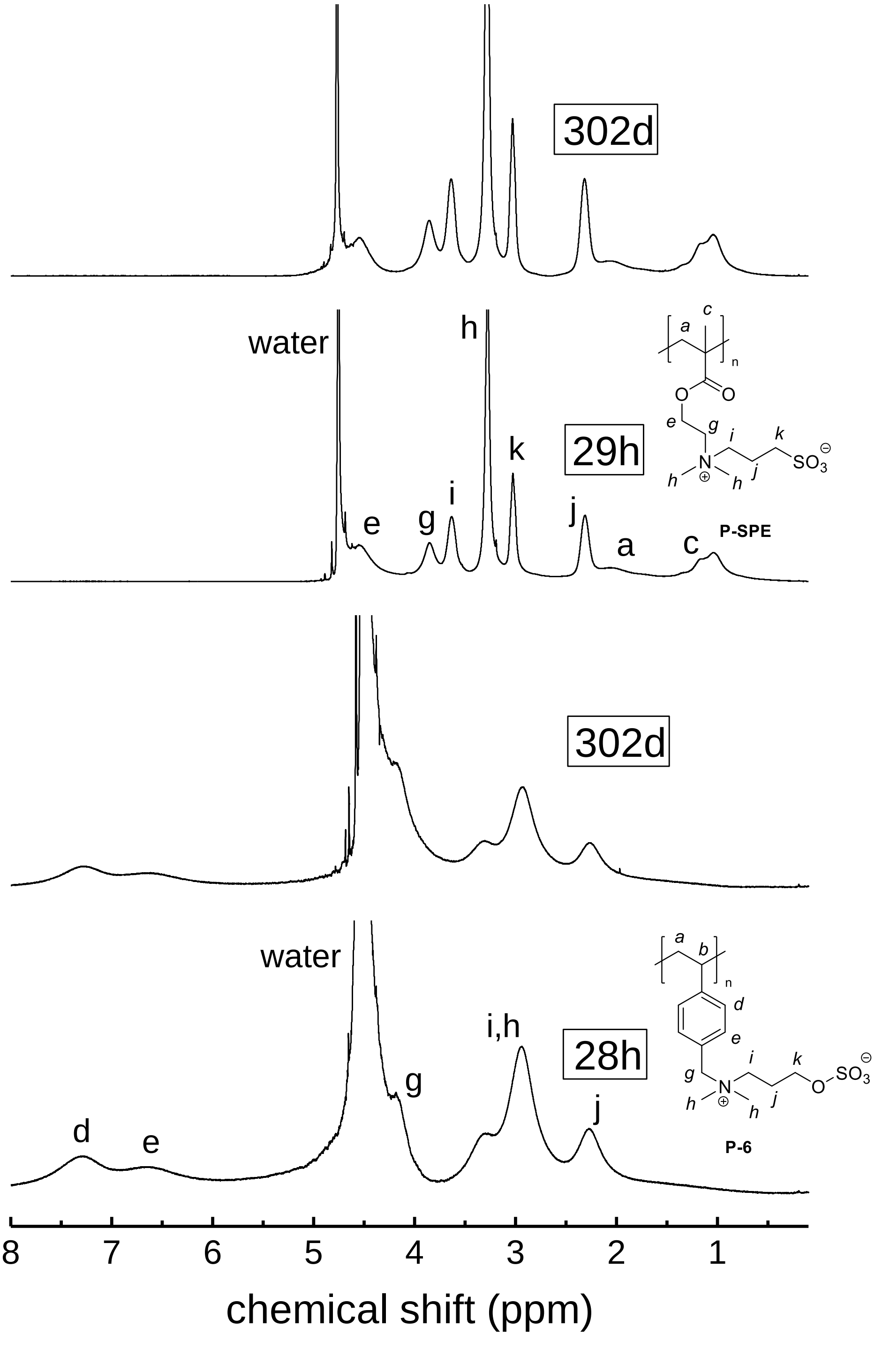


Figure S 94 $^{1}$H-NMR spectrum showing the degradation of **P-1** and **P-2** in carbonate buffer in $D_2O$ (pH = 10) (in case of P-6 saturated with sodium chloride) at room temperature over time.

4.8. Polymer hydrolysis in 1 M sodium hydroxide solution (pH=14)

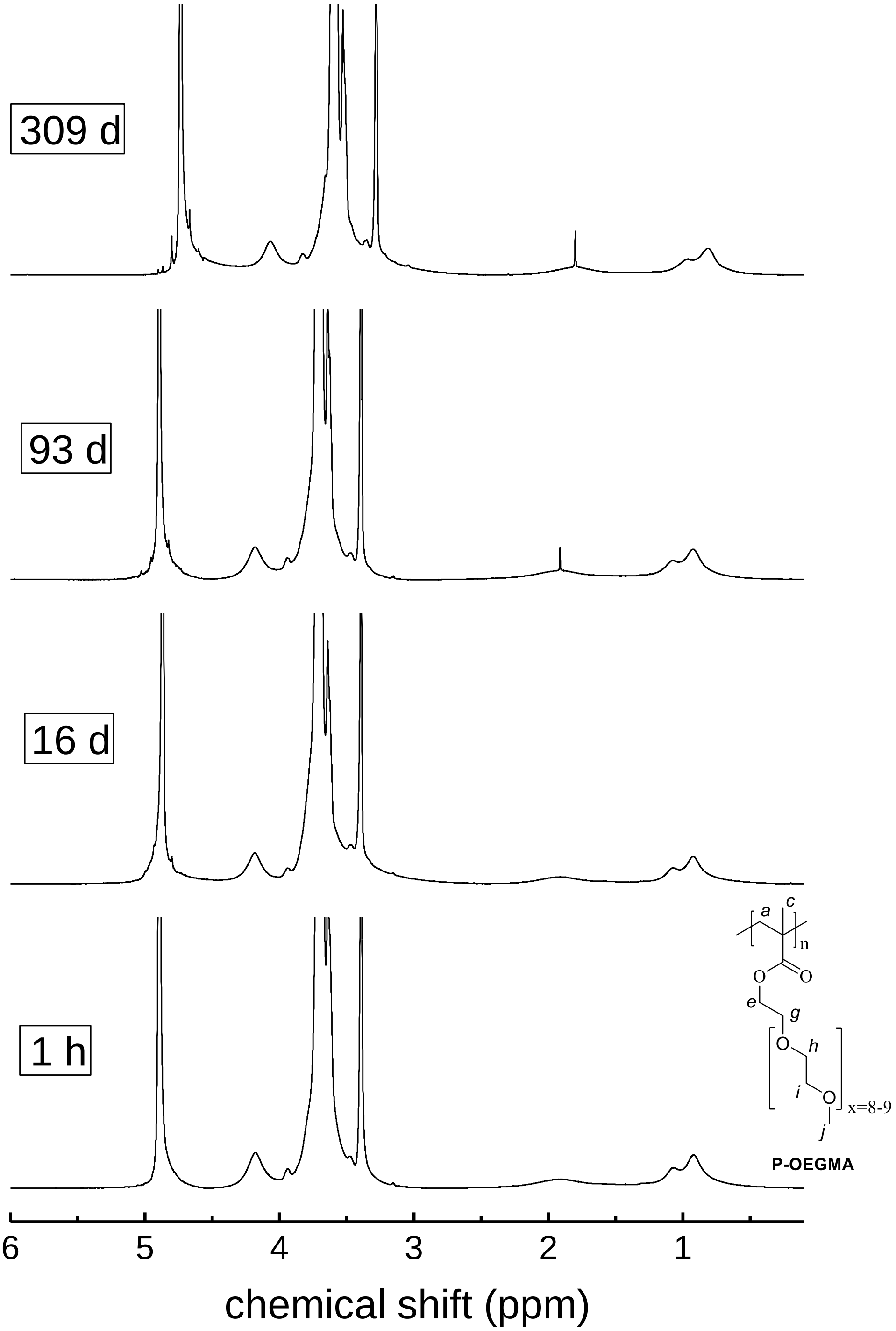


Figure S 95 $^{1}$H-NMR spectrum showing the degradation of **P-OEGMA** in sodium hydroxide in $D_2O$ (pH = 14) at room temperature over time.

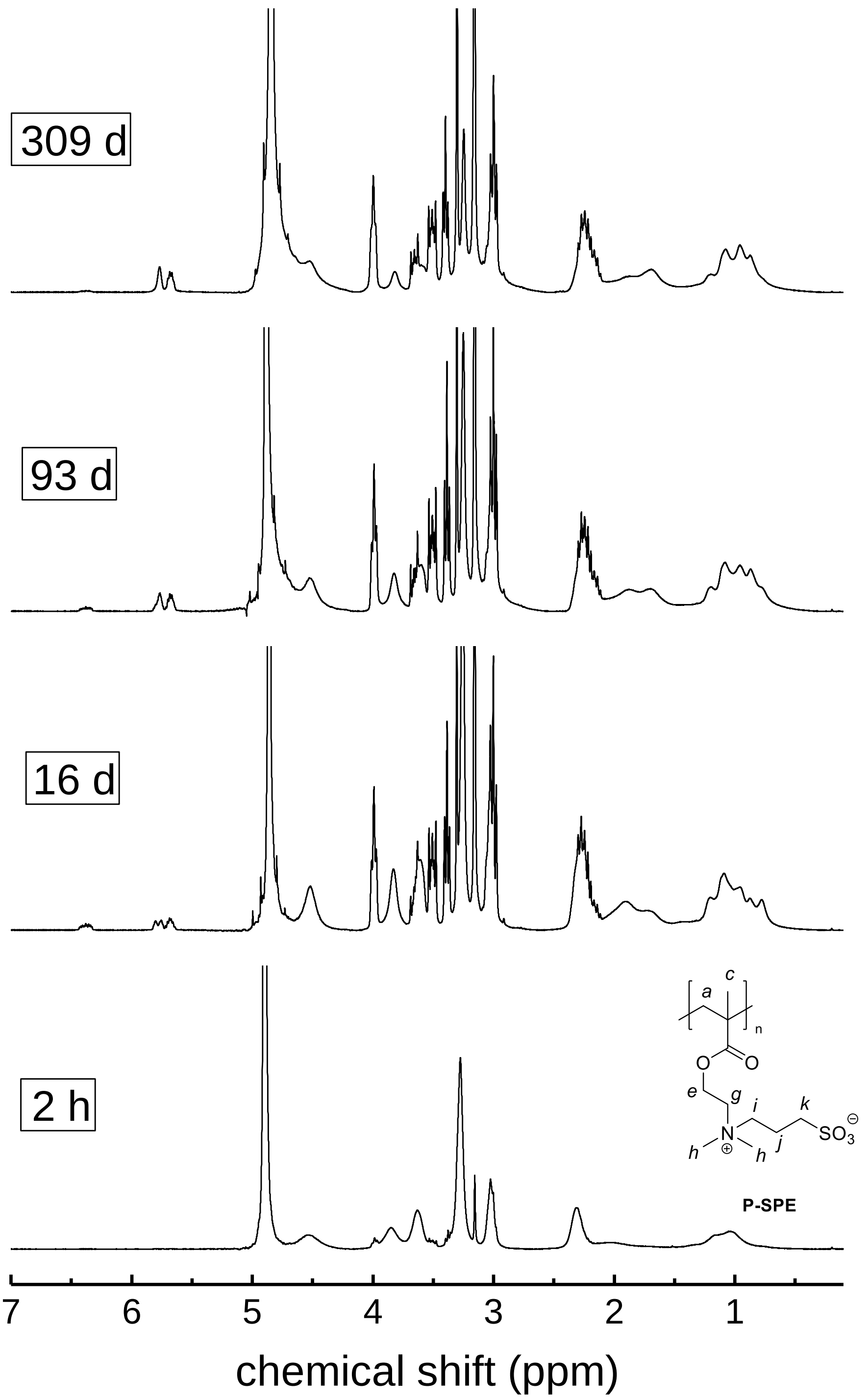


Figure S 96 $^{1}$H-NMR spectrum showing the degradation of **P-SPE** in sodium hydroxide in $D_2O$ (pH = 14) at room temperature over time.

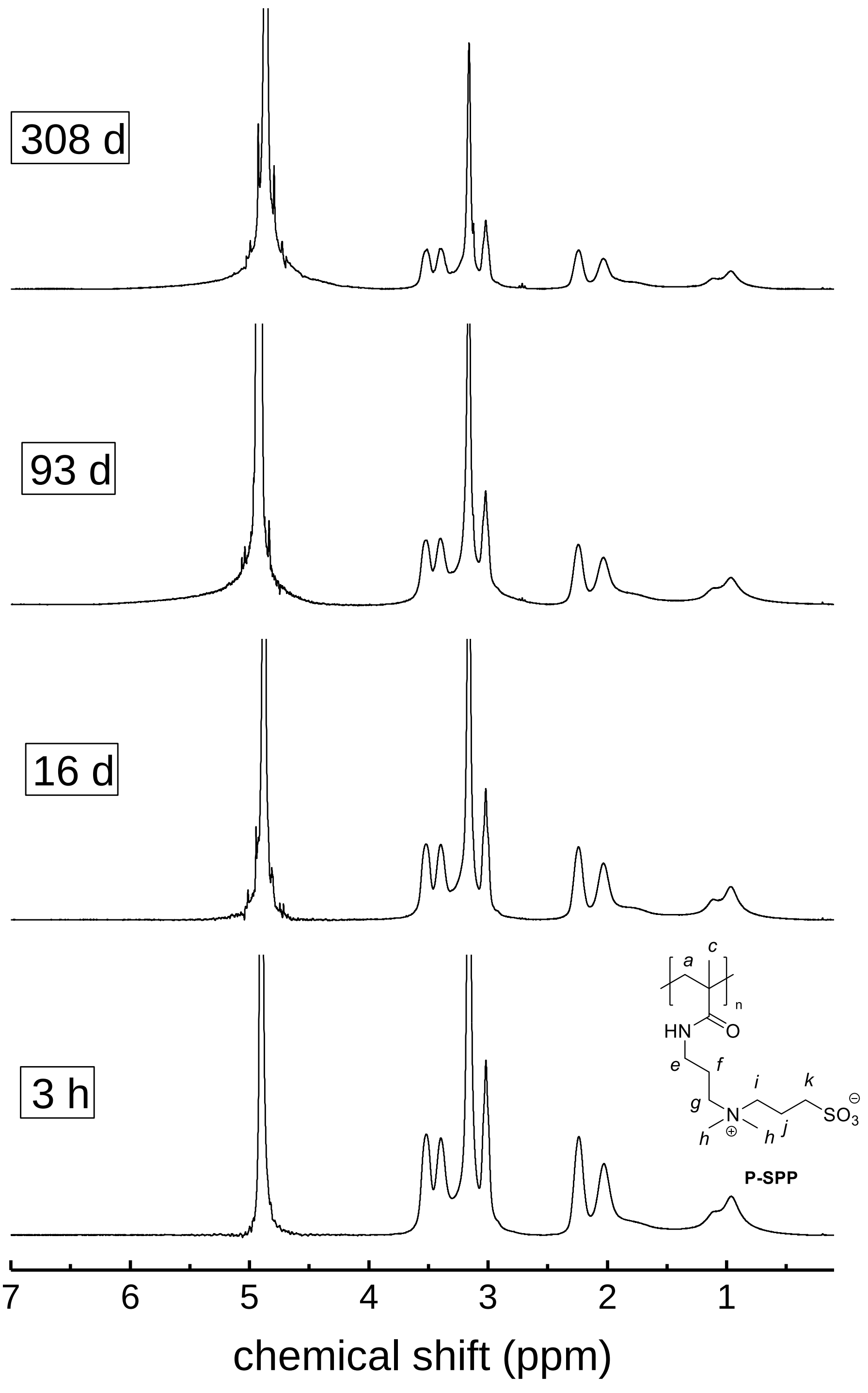


Figure S 97 $^{1}$H-NMR spectrum showing the degradation of **P-SPP** in sodium hydroxide in $D_2O$ (pH = 14) at room temperature over time.

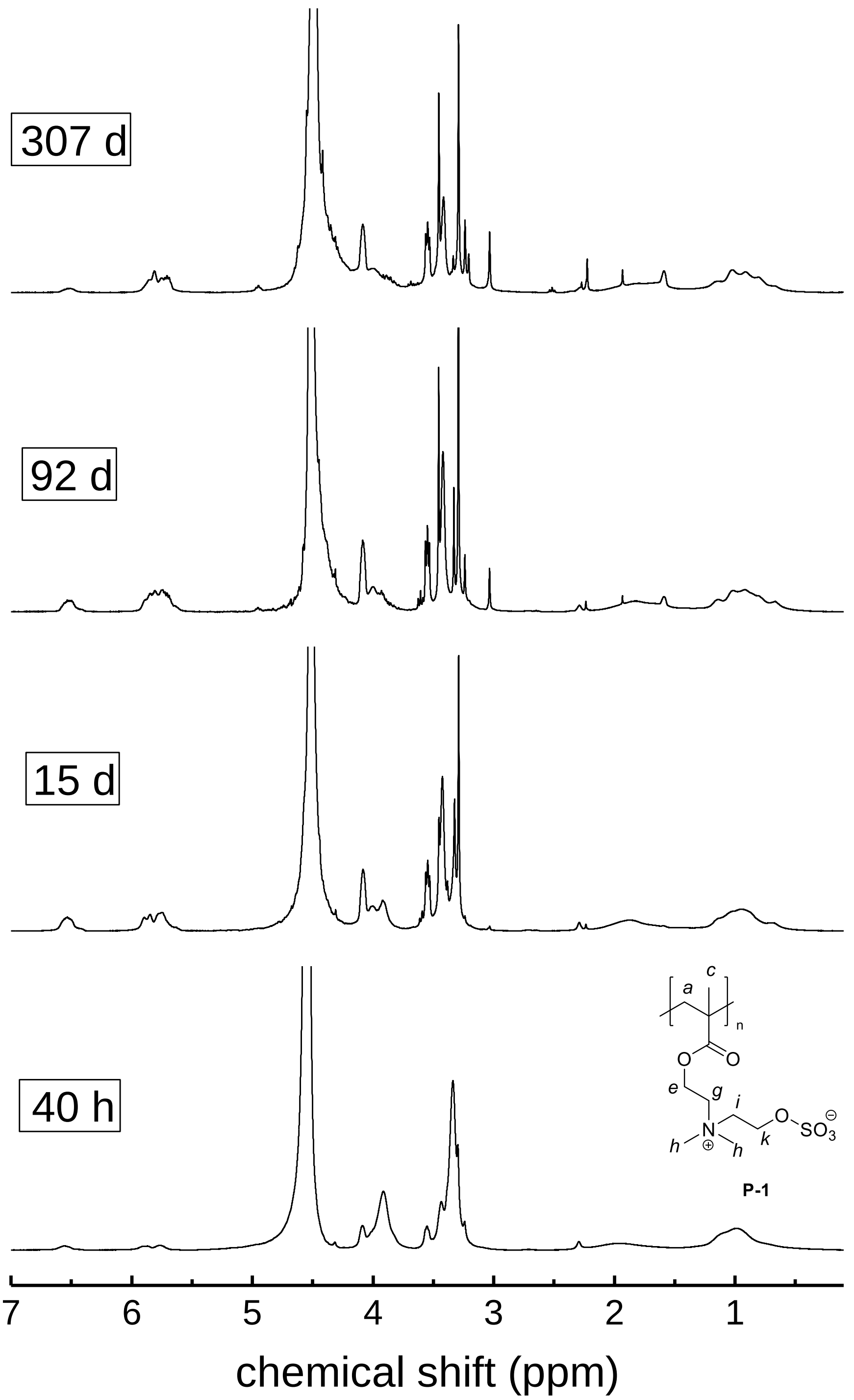


Figure S 98 $^{1}$H-NMR spectrum showing the degradation of **P-1** in sodium hydroxide in $D_2O$ saturated with sodium chloride (pH = 14) at room temperature over time.

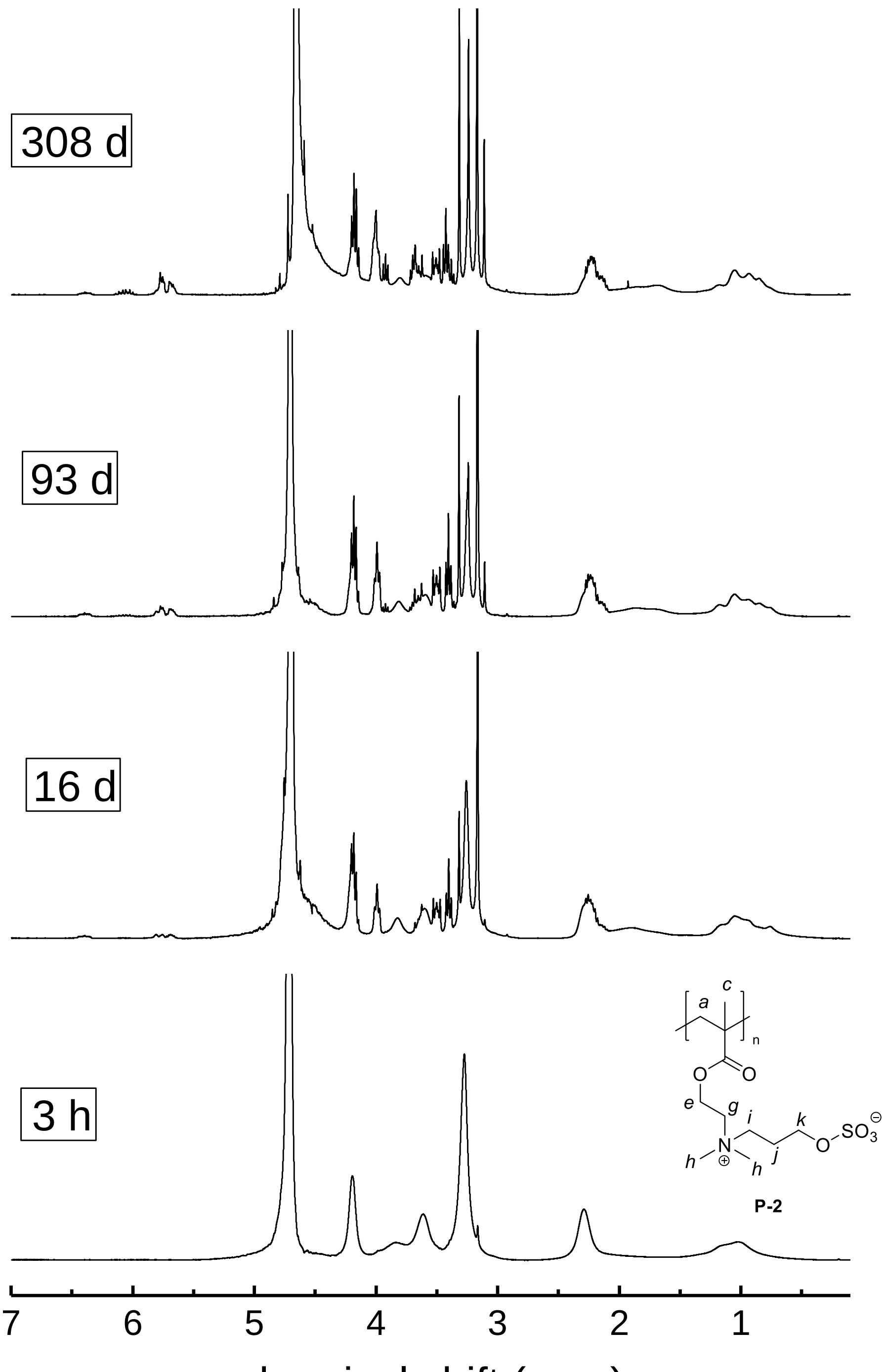


Figure S 99 $^{1}$H-NMR spectrum showing the degradation of **P-2** in sodium hydroxide in $D_2O$ saturated with sodium chloride (pH = 14) at room temperature over time.

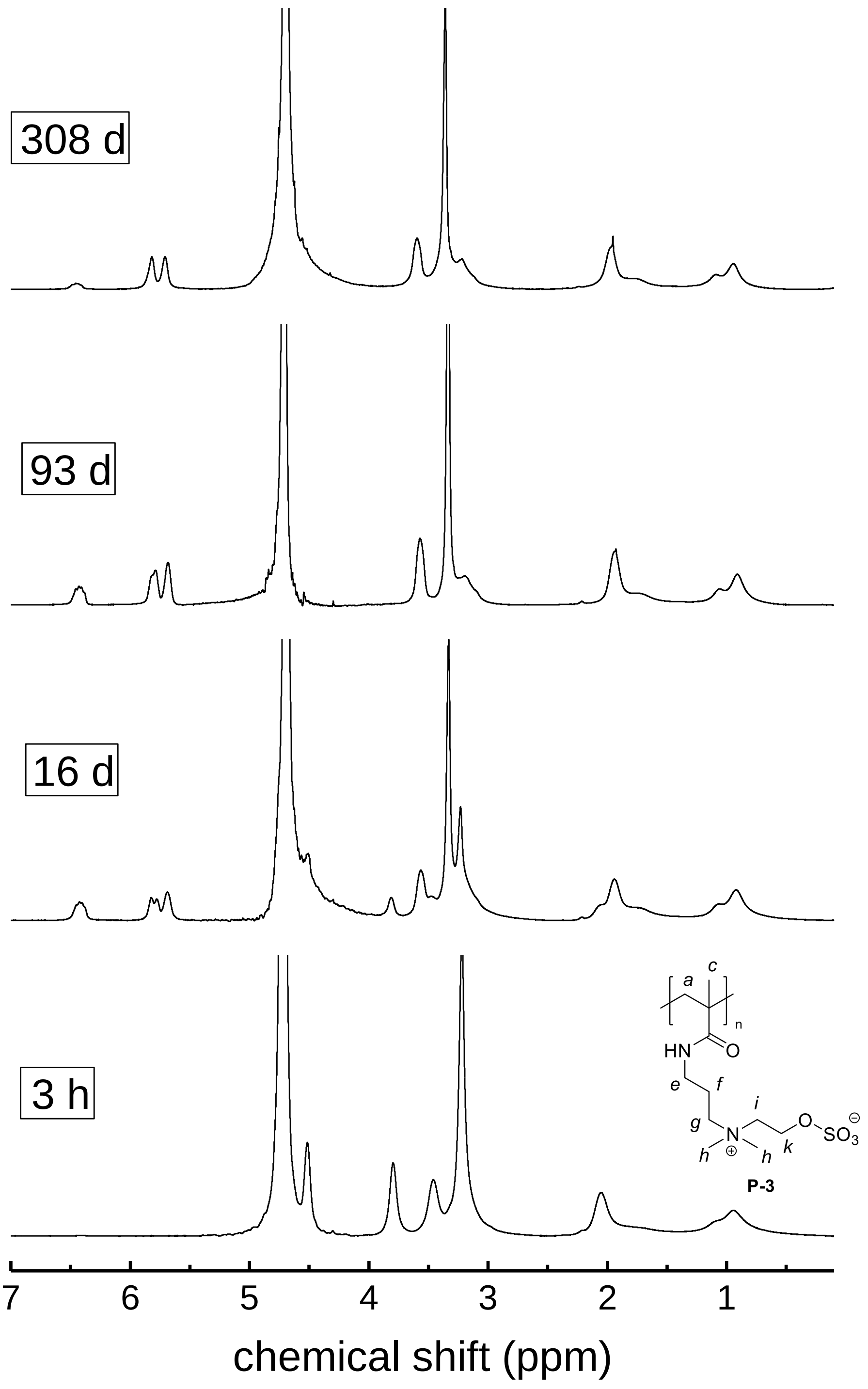


Figure S 100 $^{1}$H-NMR spectrum showing the degradation of **P-3** in sodium hydroxide in $D_2O$ saturated with sodium chloride (pH = 14) at room temperature over time.

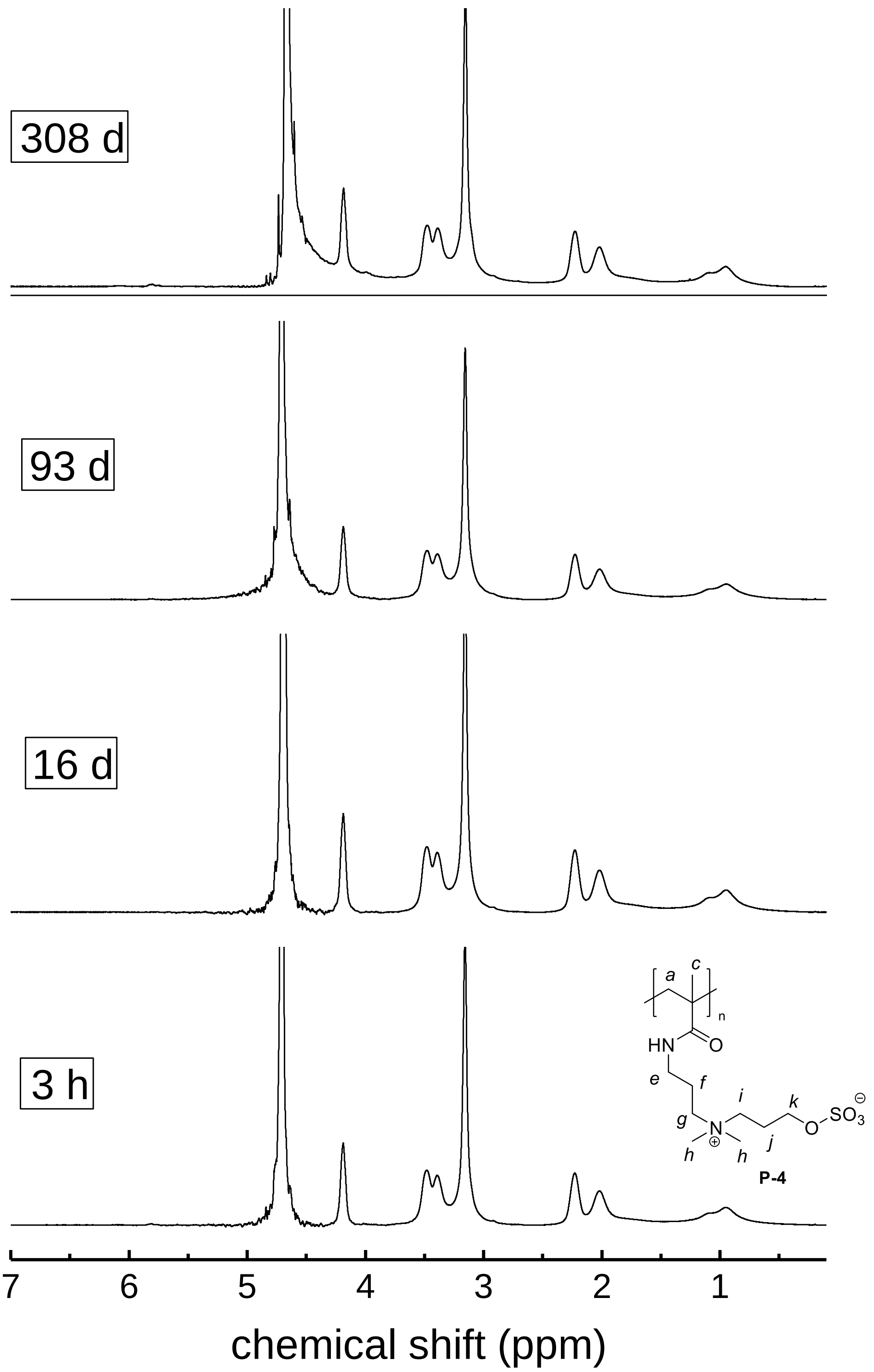


Figure S 101 $^{1}$H-NMR spectrum showing the degradation of **P-4** in sodium hydroxide in $D_2O$ saturated with sodium chloride (pH = 14) at room temperature over time.

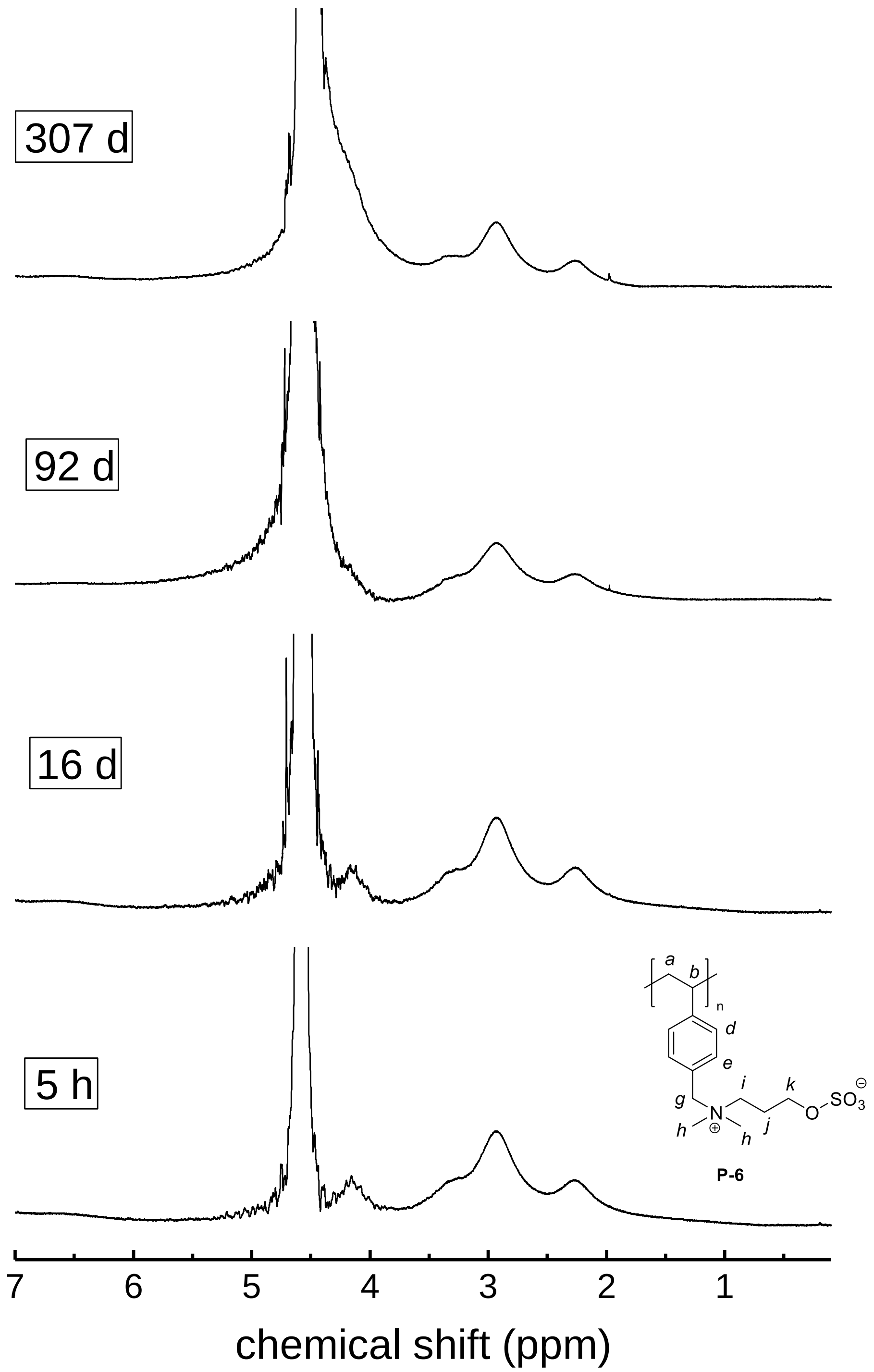


Figure S 102 $^{1}$H-NMR spectrum showing the degradation of **P-6** in sodium hydroxide in $D_2O$ saturated with sodium chloride (pH = 14) at room temperature over time.
